# Effect of fique fibers and its processing by-products on morphology, thermal and mechanical properties of epoxy based biocomposites

**DOI:** 10.1038/s41598-022-18934-x

**Published:** 2022-09-07

**Authors:** Nicolas Centeno-Mesa, Oscar Lombana-Toro, Juan P. Correa-Aguirre, Miguel A. Hidalgo-Salazar

**Affiliations:** Research Group for Manufacturing Technologies GITEM, Universidad Autónoma de Occidente, Cali, Colombia

**Keywords:** Materials science, Engineering, Mechanical engineering

## Abstract

This work examines the morphology, mechanical and thermal properties of biocomposites based on epoxy resin-EP and fique (Furcraea andina), a native crop of South America. The EP-fique biocomposites were prepared using fique powder-FP an industrial waste generated during fique processing, nonwoven fique fiber mats-NWF and unidirectional fique fiber mats-UF oriented at 0° and 90°. The addition of fique into EP matrix restricts EP macromolecule chains movement and enhance the thermal stability of EP. SEM images showed that fique form used (powder or fiber) and mat arrangement can generate changes in the biocomposites morphology. Mechanical characterization show that fique powder and fique fibers oriented at 90° acts as fillers for the epoxy matrix while the fique fibers oriented at 0° reinforce EP matrix increasing the tensile and flexural modulus up to 5700 and 1100% respectively and tensile and flexural strength up to 277% and 820% in comparison with neat EP. The obtained results can increase the interest in researching and developing products from fique Powders and other natural fibers processing byproducts thus reducing the abundance of waste in soil and landfills and environmental concerns and suggest that the EP-fique biocomposites are promising to be used in the automotive sector.

## Introduction

Fique Fibers are extracted from the fique plant (Furcraea andina), native to the Andean region of South America, which has characteristics very similar to Sisal and Henequen^1^. The Colombian Ministry of Agriculture and Rural Development stablished that during 2020 around 19,000 tons of fique plants were produced in the country^2^. Fique processing encompasses several stages: leave cutting, shredding, fermenting, drying, and packing. However, it has a low efficiency rate, since only 4% of the leaf (fibers) is used and marketed and the remaining 96% (powder and bagasse) is generally discarded in soil and ladfills, causing serious environmental concerns due to their high production and accumulation (around 100 Tons per year). Fibers are used for handicrafts and sacks manufacturing with rustic looms, using traditional technologies with very low performances. In principle, the challenge for the fique agricultural and industrial sectors consists of searching for alternatives to use the discarded byproducts and manufacturing products with high aggregated value with the fibers. Recent research has shown that the fique fibers can compete with other known natural fibers to produce reinforced biocomposites or natural fiber reinforced composites (NFRC) for technological applications. The interest in using natural fibers, such as fique, for the development of alternative materials has grown in recent years. Mainly, for the manufacture of new sustainable products with lower costs and lower CO_2_ emissions, recycling possibilities, biodegradability, improved performance of polymeric and cementitious matrices, lower weight, and high availability^3,4^.These characteristics could be considered an advantage over composite materials based on synthetic fibers such as glass or carbon, which present oil dependence and waste management concerns after their life cycle.

Several studies have shown that the mechanical properties of biocomposites are influenced by several factors such as fiber content^5,6^, the chemical treatments of the fibers^7^, geometry and orientation of the natural fibers^8^, and the difference in failure mechanisms between particles, short and long fibers reinforced biocomposites^9^.

Salman et al.^10^ studied the mechanical and morphological properties of woven kenaf oriented at 0°/90° and 45°/ − 45° and its biocomposites made with epoxy, polyester and vinyl ester resins. Their results show that fiber orientation 0°/90° induces the highest mechanical properties (tensile, flexural strengths, and modulus) in the biocomposites. Another study conducted on the effect of fiber content and orientation on the mechanical properties of epoxy-jute-Kevlar hybrid composites by Maharana et al.^11^revealed that 40% fiber loading with 30° fiber orientation enhances the hybrid composite tensile properties, whereas flexural properties were maximized for 40% fiber loading with 45° fiber orientation. Prabakaran et al.^12^ researched the effect of fiber orientation on the mechanical properties of Epoxy-Glass Fiber composites oriented at 0°/90°and non-woven mat composites. Their results show that spirograph non-woven, mat-based composites exhibit a quasi-isotropic fiber orientation and the better mechanical performance compared to woven laminated composites. In recent years, epoxy based biocomposites, using lignocellulosic biomass as wheat straw^13^, banana, kenaf^14^, bamboo^15^, sisal^16^, date stone flour^17^, have been reported and produced.

This paper focuses on whether fique fiber mats (non-woven and oriented at 0° and 90°) and powder can be used effectively for the manufacturing of epoxy-based biocomposites. In this study the Epoxy based biocomposites were manufactured using the resin film infusion process. The effect of the fique on the morphology, thermal, tensile, and flexural properties was evaluated, as well. These characterizations are important for developing alternative solutions for industry. We aim to evaluate the use of this natural fiber and its powdered processing residues as a reinforcement for Epoxy-based biocomposites to generate a positive impact on regional economies, technological development and expand the range of fique applications. The feasibility study, presented here, used to develop prototypes of car parts from the presented biocomposites, using a scalable process and the inclusion of a highly produced agroindustrial byproduct as a filler for biocomposites production supports the novelty of this article. Furthermore, the obtained results can increase the interest in researching and developing products from fique powders and other natural fibers processing byproducts, thus reducing the abundance of waste in soil and landfills and environmental concerns.

## Materials and methods

### Materials

The Epoxy Resin used (EP) was a general purposed and low modulus Epoxy system based on a low viscosity Epoxy resin type Bisphenol A, reference ‘‘R3610”, and a modified cycloaliphatic amine hardener, reference ‘‘E-1610”. The Epoxy resin and hardener were purchased from Sintepox (Bogota-Colombia). The Epoxy system was prepared by blending the epoxy resin and the hardener, using a 50:50 (%wt/wt) mixing ratio. The average measurement density of the cured EP was 1.11 ± 0.03 g/cm^3^. These measurements were performed following the ASTM D792-13 standard by means of the Archimedean method, using a multifunction solid densimeter DA-300 (Dahometer, China).

**Figure 1:**
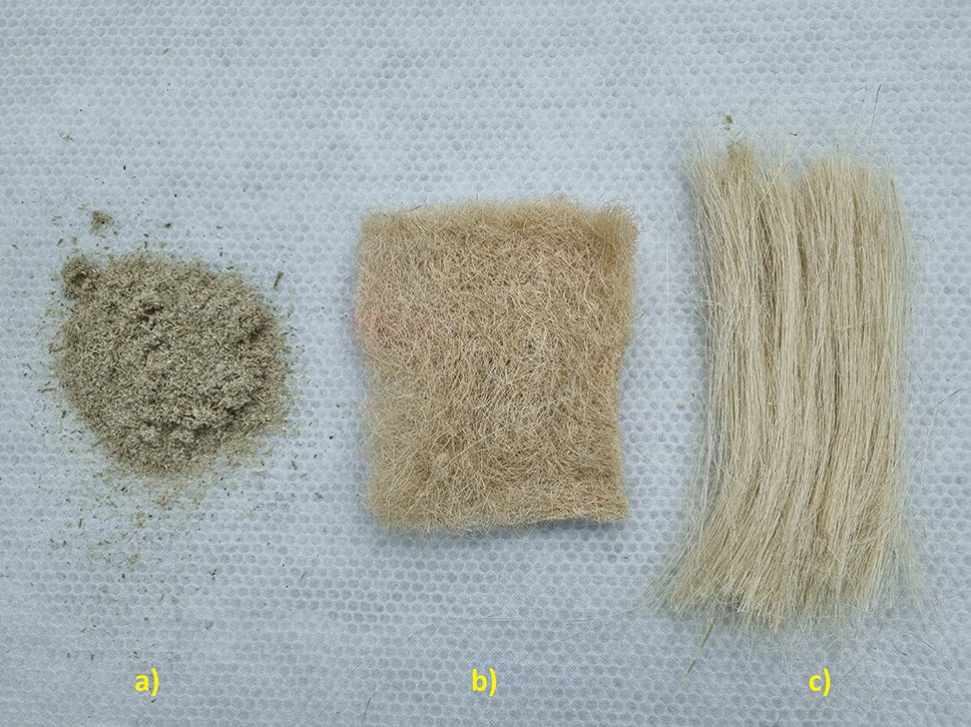
Raw fique: (**a**) powder (FP), (**b**) non-woven mat (NWF), (**c**) unidirectional mat (UF).

The fique powder (FP) and unidirectional fique (UF) mats were provided by “Empaques del Cauca” (Popayan-Colombia). FP is a processing waste generated during fique fibers processing and was only sieved through a 400 µm sieve. Non-woven industrial fique fiber mats (NWF) were supplied by ‘‘Packaging Company of Medellin” (Medellin-Colombia). UF and NWF mats were used as received and dried in an oven at 80 °C for 24 h. before biocomposites manufacturing (Fig. 1). The average density of fique was 0.78 ± 0.09 g/cm^3^. Experimental research and field studies on plants (either cultivated or wild), including the collection of plant material developed in this paper, comply with relevant institutional, national, and international guidelines and legislation.

### Biocomposites preparation

**Figure 2:**
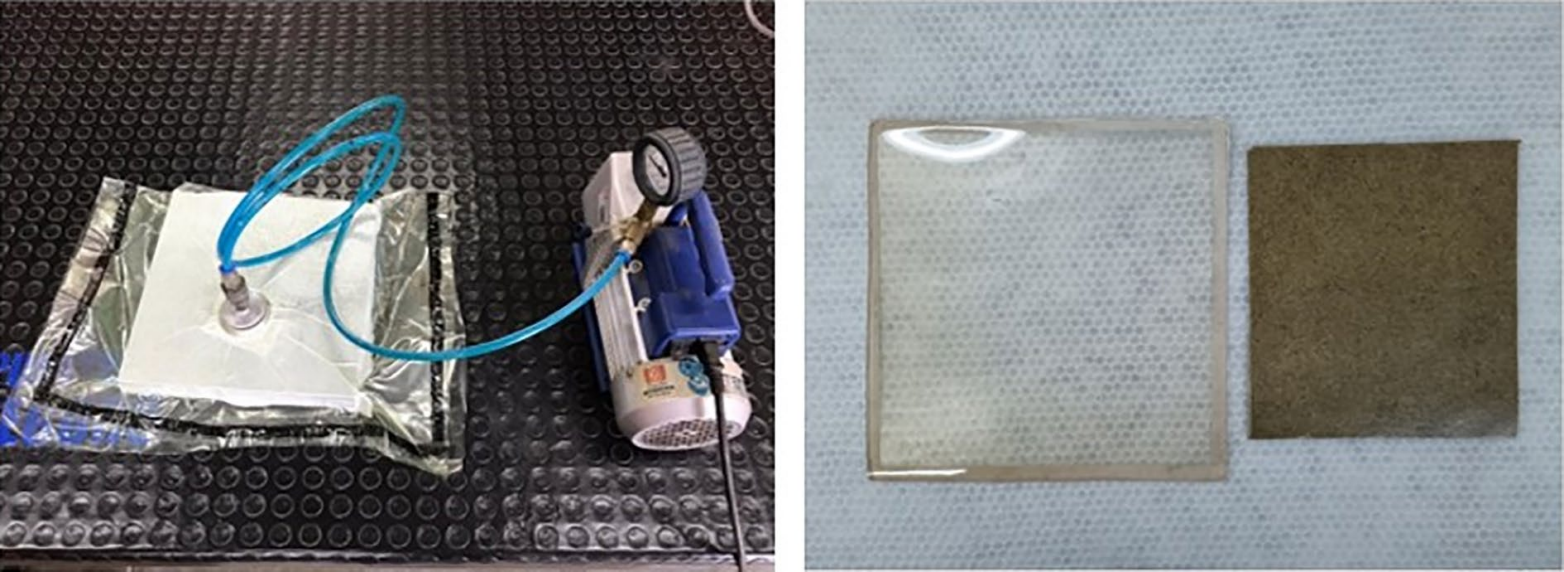
Resin film infusion process (left) and neat EP and EP-FP biocomposite sheets obtained with the resin film infusion process (right).

The neat EP and EP-fique biocomposites sheets were manufactured using the Resin Film Infusion technique in a Teflon mold (300 mm × 300 mm). For all biocomposites the EP-fique composition was 70/30 (% wt/wt) or 62/38 (% v/v) according to density measurements. For EP-fique mats the Teflon mold surface was first coated with a thin layer of EP, then fique matswere placed on the mold to obtain three different arrangements: EP-NWF using the non-woven fique and EP-UF using oriented fibers at 0° and 90° (see Fig. 4). After that, the fique mats were filled with more EP and were left to be absorbed. For the EP-FP biocomposite, the EP and the FP were manually mixed until a homogeneous mixture was obtained. This mixture was left for 5 min in a vacuum chamber to lose all the air bubbles produced during mixture for 5 min and then spread within the mold. Finally, the mold was placed inside a flexible film and left to cure under vacuum conditions for 24 h at room temperature (Fig. 2).

The obtained EP-fique biocomposites sheets show that the Resin Film Infusion technique enables the manufacture of homogeneous sheets with well-distributed fique fibers and powder that could be used for large scale automotive parts. By using the proposed method, natural fibers and processed waste can be utilized in industrial production.

When the neat EP and EP-fique biocomposites sheets were ready, they were removed from the molds and cut into different tensile and flexural test specimens using a water jet cutter (Protomax-Omax, Kent, USA). The water jet cutting procedure was selected due to the materials being sensitive to the high temperatures generated in other cutting methods.

### Product application

**Figure 3:**
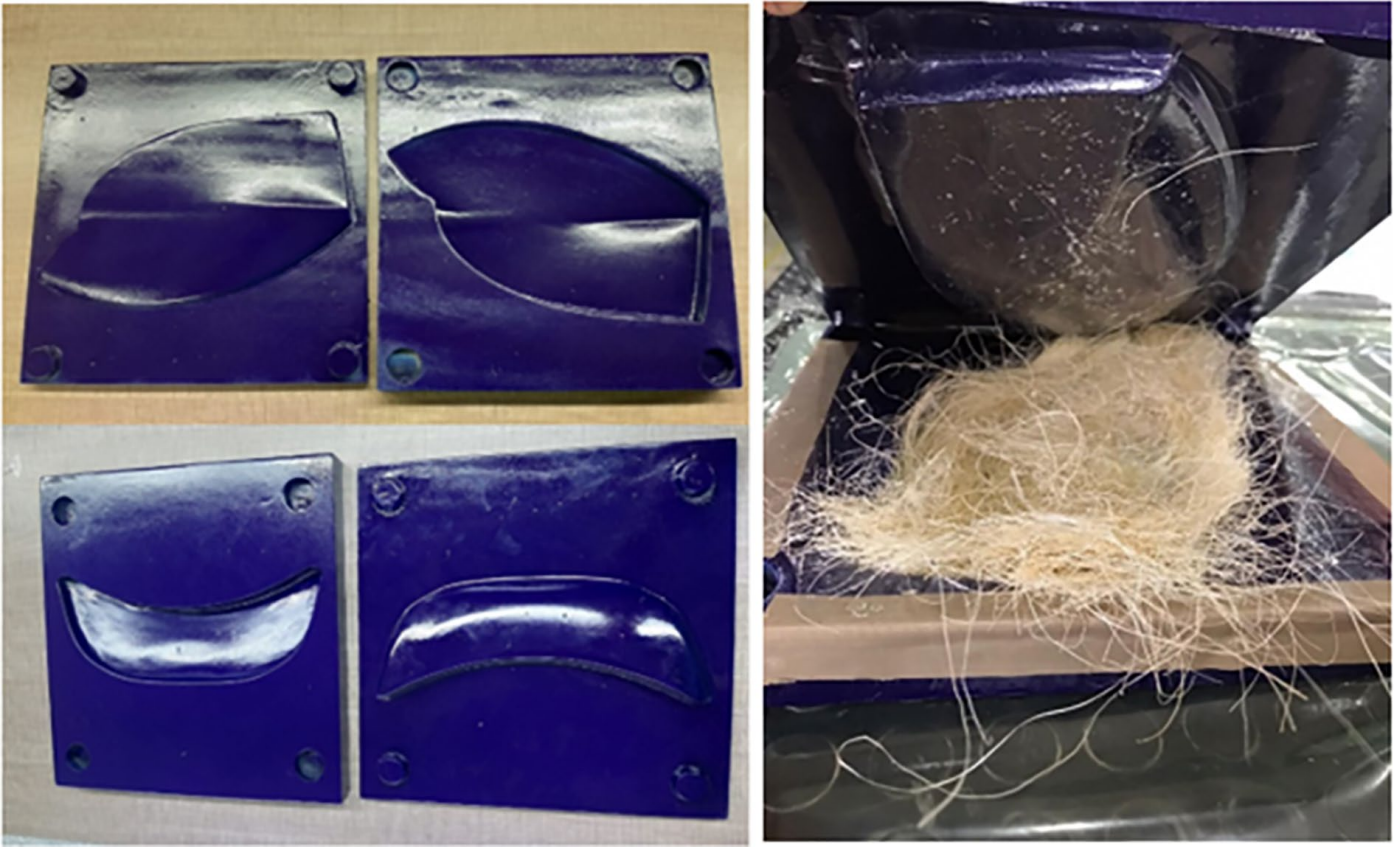
Molds and configuration used for manufacturing automotive parts with the resin film infusion process.

From the studied biocomposites, the EP-UF 0° was selected to assemble two automotive parts to observe the functionality of these materials in a real manufacturing process, such as resin film infusion. The EP-UF weight fraction was 70/30 (% wt/wt) or 62/38 (% v/v) and was selecteddue to its mechanical characterization results (see “Mechanical properties'' section). To obtain the prototype parts, two molds composed of two cavities (male and female) were manufactured on scale due to the resource limitations in the work area of the CNC router, where the wood cutting was performed. After the molds were prepared, the parts were fabricated, following the methodology explained in “Biocomposites preparation'' section. Figure 3 shows the molds used and the disposition of the mats and the resin within the mold before the curated process.

### Materials characterization

#### Morphology

A scanning electron microscope (SEM) Phenom PRO X (Thermo Fisher Scientific, USA) was used to scan the cross-sectional area, the distribution of fique fibers and powders and morphological features in the bulk of the samples. The samples were immersed in liquid nitrogen for 15 min to obtain a brittle fracture and were sputter-coated with gold to increase their electric conductivity. A voltage of 10 kV was applied and magnifications of 500 × and 2000 × were taken.

#### Thermal Properties

Neat Epoxy resin, Epoxy-fique based biocomposites, fique powder and fique fibers where thermally characterized using DSC and TGA (data available in the supplementary information). DSC tests of Neat Epoxy resin, Epoxy-fique based biocomposites were carried out using a TA Q2000 differential scanning calorimeter (Texas Instruments, Dallas, TX, USA) under the following conditions:

- Nitrogen atmosphere
- Scanning speed of 10 °C/min
- Sample weight 10 mg

The samples were first subjected to heating cycles ranging from 20 to 150 °C to erase the thermal history related to processing events. It was followed by cooling cycles bringing the temperature down from 150 to − 20 °C. Finally, the second heating cycles rise from − 20 to 150 °C. For this study, the cooling and second heating cycles were reported.

Thermogravimetric analysis (TGA) tests were performed using a TA Q500 thermogravimeter (Texas Instruments, Dallas, TX, USA) with a rising temperature starting at 25 to 600 °C at a heating rate of 10 °C/min. DSC and TGA samples were analyzed in aluminum crucibles under a N2 atmosphere.

#### Mechanical properties

Tensile tests and three-point bending flexural tests were performed in a universal testing machine INSTRON Model 3366 (INSTRON, Norwood, MA, USA) under the following conditions:

- Conditioning were performed at 23 °C and 50% RH for seven days
- Tensile tests were performed using an axial extensometer INSTRON model 2630 (gauge length 50 mm) (INSTRON, Norwood, MA, USA) and type I specimens (ASTM-D 638-14) using a crosshead speed of 5 mm/min.
- Flexural tests were performed on bars with a rectangular cross-section (12,5 mm * 3 mm), using a crosshead speed of 1.3 mm/min, a distance between support spans of 50 mm up to 5% strain following ASTM D 790-17 standard.
- The results were taken from an average of five samples.

**Figure 4:**
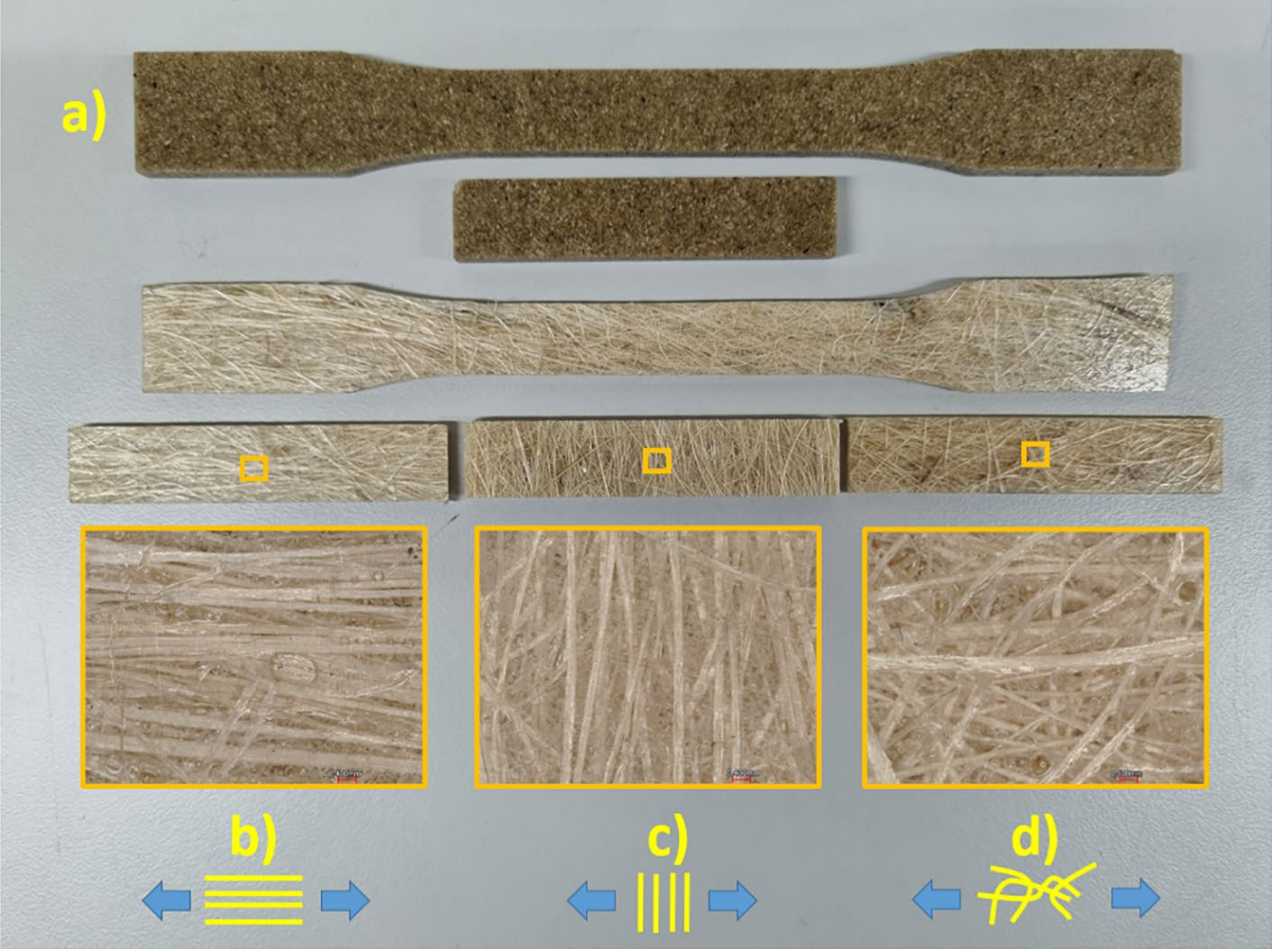
Tensile and flexural samples of (**a**) EP-FP (**b**) EP-UF 0° (**c**) EP-UF 90° and (**d**) EP-NWF biocomposites.

Figure 4 shows the specimens of EP-fique biocomposites used for the tensile and flexural tests. For EP-FP biocomposites (Fig. 4a), well-distributed fique powder particles were observed on the surface. In the biocomposites from fique mats, long fique fibers can be observed oriented at 0° (Fig. 4b), 90° (Fig. 4c) and in a two-dimensional random arrangement (Fig. 4d).

#### Statistical analysis

Tensile and flexural properties of the materials were subjected to analysis of variance (ANOVA), and the Tukey’s test was applied at the 0.05 level of significance. All statistical analyses were performed using Minitab Statistical Software Release 12 (Pennsylvania, USA).

## Results and discussion

### Mechanical properties

**Figure 5:**
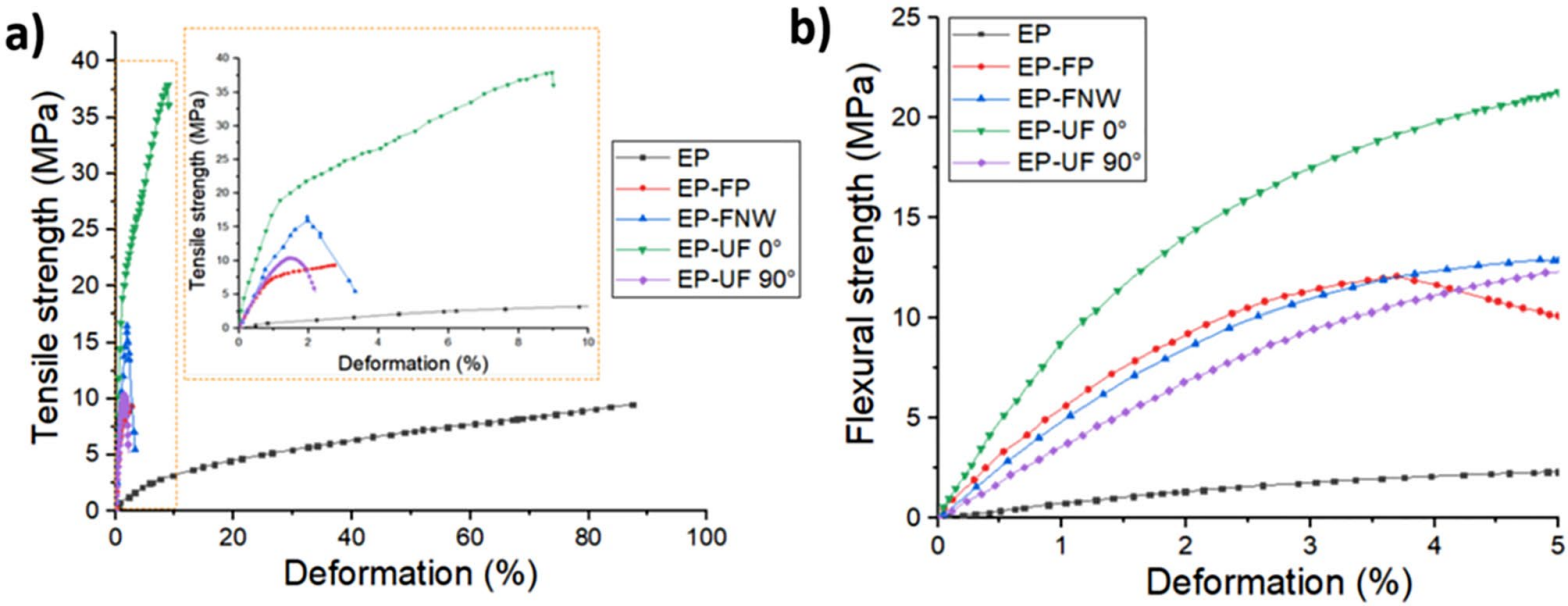
Average Tensile and flexural stress vs deformation of neat EP and their EP-fique biocomposites.

Figure 5 shows the influence of the addition of fique to the EP mechanical properties (data available in the supplementary information). These results are also summarized and presented in Table 1.

**Table 1:** Tensile and flexural properties of EP and EP-fique biocomposites.

Sample	Tensile and flexural properties*				
	Tensile properties			Flexural properties	
	Modulus (MPa)	Strength (MPa)	Deformation at break (%)	Modulus (MPa)	Strength (MPa)
Epoxy resin (EP)	34 ± 10a	9.7 ± 3.3a	90 ± 15.3a	83 ± 24a	2.3 ± 0.6a
Epoxy resin-fique Powder (EP-FP)	925 ± 200b	8.8 ± 0.6a	2.8 ± 1.1b	594 ± 85b	12.4 ± 1.2b
Epoxy resin-non woven fique fiber mat (EP-NWF)	1074 ± 198b	16.6 ± 1.7b	3.8 ± 0.8b	390 ± 65c	12.9 ± 0.2b
Epoxy Epoxy resin-unidirectional fique fiber mat oriented at 90° (EP-UF 90°)	1080 ± 308b	10.6 ± 1.2a	2.2 ± 0.3b	368 ± 12c	12.3 ± 0.8b
Epoxy resin-unidirectional fique fiber mat oriented at 0° (EP-UF 0°)	2000 ± 414c	36.6 ± 4.3c	9.0 ± 0.8c	1014 ± 159d	21.2 ± 4.6c

a–c Different letters in the same column indicate significant differences (*p* < 0.05).

*Average of five replications ± standard deviation.

Tensile tests show that fique powder and fibers incorporation generate significant increases (*p* < *0.05*) in tensile modulus (TM) values between 2600 and 5700% for EP-FP and EP-UF 0° (UF oriented parallel to the applied load) compared with the neat EP matrix. This stiffening effect is caused by the higher mechanical properties of fique and a decrease in the EP mobility due to fique fibers observed in DSC tests ("Thermal Properties'' section).

Tensile strength (TS) of EP-FP and EP-UF 90° (UF oriented perpendicularly to the applied load) was not significantly different from EP (*p* ≥ *0.05*). However, TS values of EP-NWF and EP-UF 0° increased 71 and 277% respectively, compared with EP. These results show that fique powder and the unidirectional fique mats oriented at 90° acts as a filler, while non-woven and unidirectional Fique mats oriented at 0° reinforced the EP matrix.

Previous studies performed on PP-Kenaf^18^ and HDPE-henequen^19^ biocomposites suggested that biocomposites with fibers oriented parallel to the applied load (0°) were able to contribute the same applied load and possessed a much longer fiber structure due to minimal fiber breakage. Thus, aiding in the strengthening of the biocomposite structure due to a homogeneous distribution of the load. On the other hand, 90° orientation biocomposites supports less tensile loads and resulted in a higher fibers breakage^18,19^. Therefore, the mechanical performance of EP-fique biocomposites is highly dependent on the structure and the fibers orientation angle.

**Figure 6:**
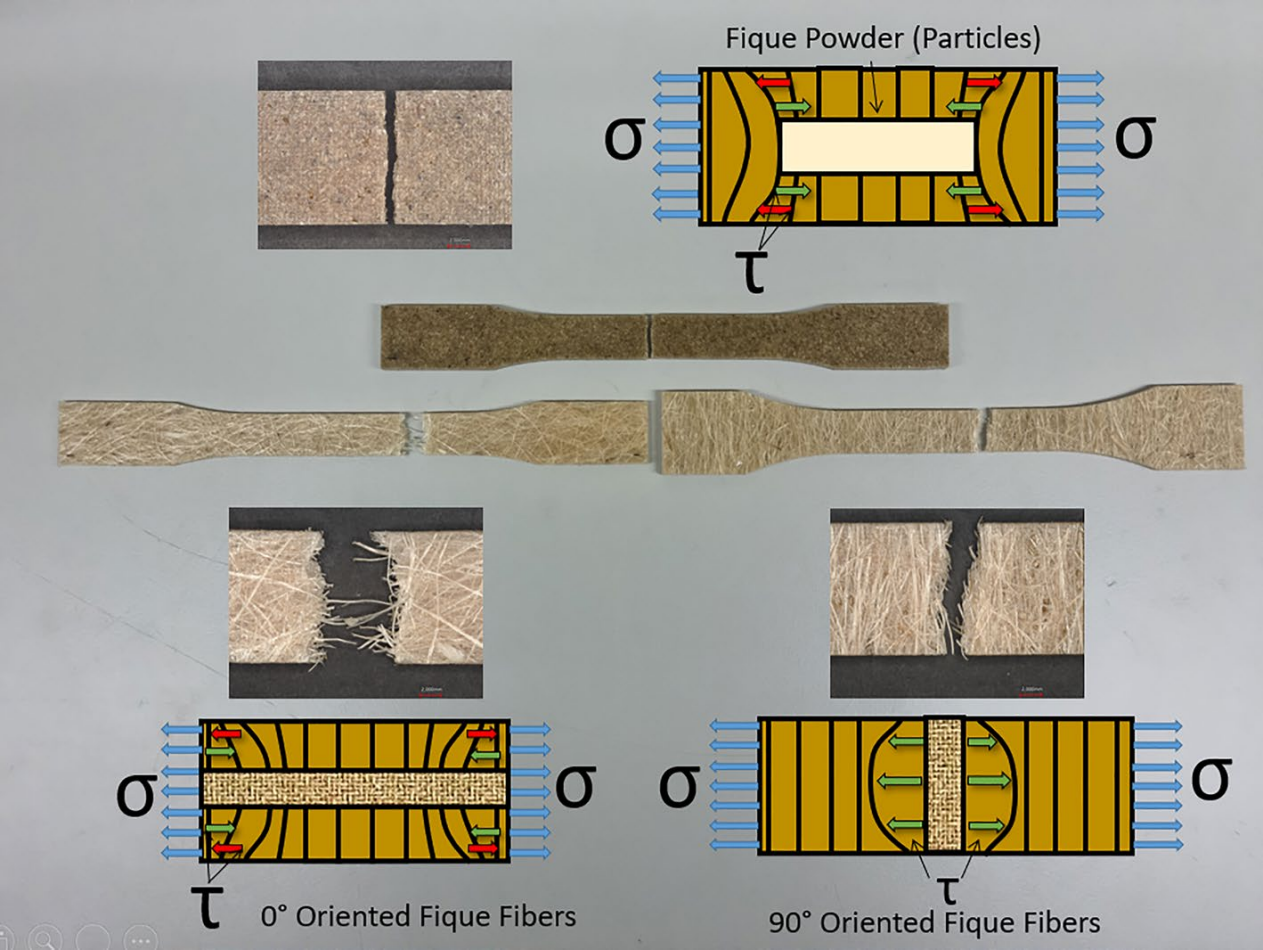
Fracture modes observed in EP-FP, EP-UF 0°, EP-UF 90°composites after tensile test.

Figure 6, shows that failure of the EP-UF 90° biocomposite material occurs and propagates along the fique fibers at 90° orientation angles. This also explains why the 0° aligned EP-UF biocomposite were able to maintain good tensile strength compared to the EP-FP and EP-UF 90° samples. Also, natural fibers with a higher cellulose content and a higher aspect ratio (L/D) may contribute to higher reinforcing efficacy, because the contact between the reinforcing elements and the matrix occur over a larger surface.

At the same time, fique incorporation decreases the deformation at break (ε_b_) of the EP matrix and could be related to the weak interfacial bonding between fique and EP as well as the stiffening effect of the interface discontinuities that affect the biocomposites deformation capacity (see "Morphology '' section). For the EP-FP samples the interfacial area between the matrix and fique particles increases due to higher superficial area, as a result εb values decreases from 90 to 2.8%. Regarding EP-fique mats biocomposites, εb decreases between 97 and 80% for EP-UF 90° and EP-UF 0° respectively in comparison with EP matrix (*p* < 0.05). This result could be related to a greater stress transfer between EP and fique fibers and fiber slippage within the matrix for EP-UF 0° orientation. Flexural test results show that flexural modulus values (FM) increase around 340 and 1100% for biocomposites EP-90° and EP-UF 0° respectively, compared with neat EP. The flexural strength (FS) increases around 430 and 820% for EP-90° and EP-UF 0° respectively in comparison with neat EP.

In general terms, the addition of fique fibers improved the mechanical properties of the epoxy matrix and generates a stiffening and reinforcing effect that could prove relevant for product applications such as automotive parts where rigidity and resistance are essential factors. The results showed that long fique fibers oriented at 0° generated the best mechanical properties among the analyzed samples, therefore the discussion about the fique fibers orientation is also crucial since it has effects on the performance and quality of the biocomposites.

Figure 6 shows the specimens and the fracture mechanisms of the EP-FP and EP-UF biocomposites after the tension tests. For the EP-FP and EP-UF 90° biocomposites, it should be noted that the fracture is generated by the simultaneous failure of the matrix and the dispersed fique particles or long fibers oriented at 90° within the matrix. In the case of EP-UF 0° samples, matrix failure occurs first, followed by the aligned fique fibers.

This difference in the fracture mechanisms between composites formulated with particles and oriented fibers has already been observed by other researchers^9,20^. These studies concluded that the fracture plane is obtained for the area with the minimum resistance of the interface between the matrix and the fibers or in the area with a lack of fibers or adhesion with the matrix.

Tensile test results show that unidirectional fique fibers oriented at 0° act as a reinforcement of the EP matrix, increasing the tensile strength almost 300% compared to the fique powder and fique fibers oriented at 90°, which did not generate significant differences in the tensile strength. The stress generated during the tension test causes shear stresses (τ) between the fiber and the matrix that generate an interface debonding. At the edge of the interface, the stress transfer from the matrix to the fibers depends on τ at the axial interface. For EP-FP and EP-UF 90° biocomposites, the interface between the particles, the oriented fibers and the matrix is easy to unbound during the test, but in the case of EP-UF 0° biocomposites the stress (τ) generated at the edge of the interface is higher due to the greater adhesion of EP and fique fibers, as well as a higher surface of contact between the reinforcing elements and the matrix. Thus, explaining the observed fracture mechanisms and the reinforcement observed in the biocomposites with unidirectional fique fibers.

### Thermal properties

**Figure 7:**
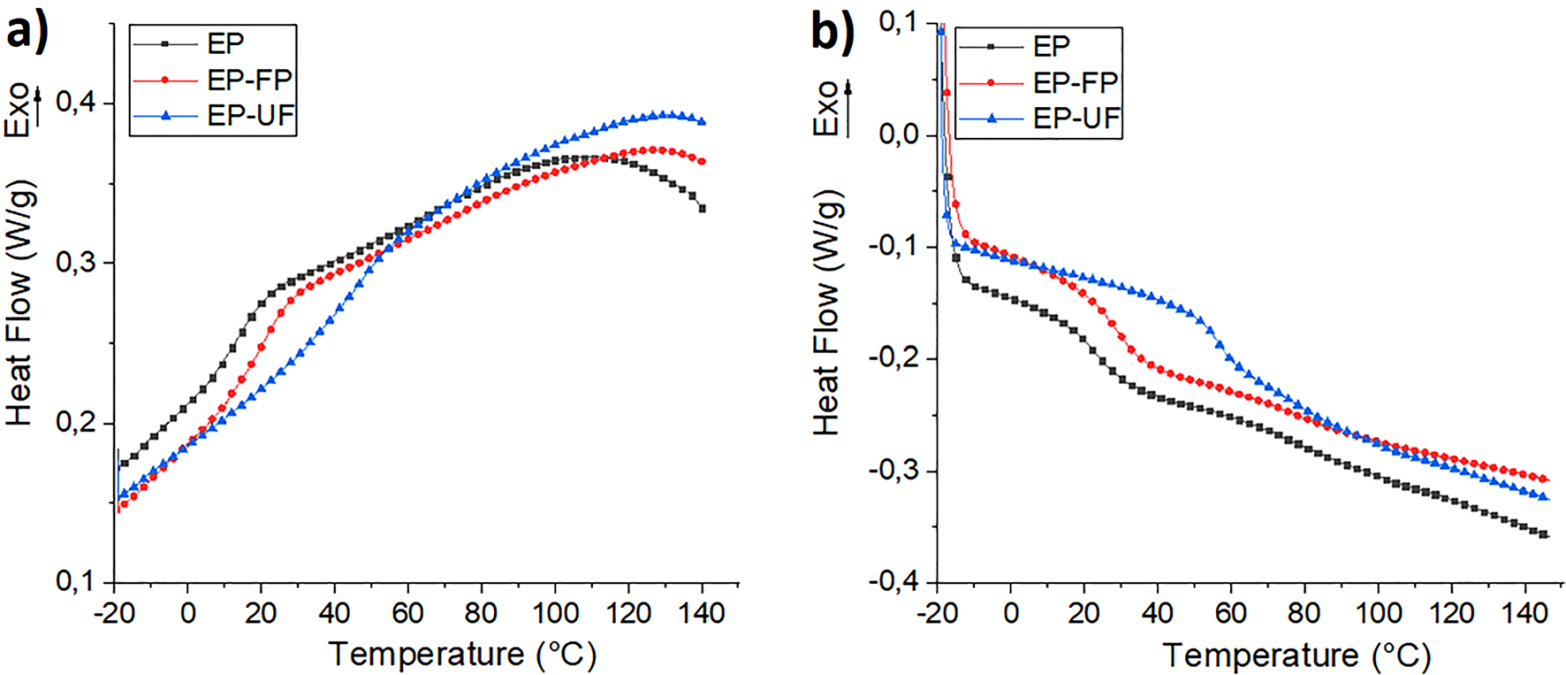
(**a**) Cooling and (**b**) Second Heating for neat EP and their fique biocomposites.

The cooling curves show the absence of crystallization exotherms, which indicates that the EP and the EP-fique biocomposites do not present crystallization during cooling (Fig. 7a.) The second heating cycle (Fig. 7b) shows the glass transition temperature (Tg) of the EP at 22 °C. The addition of fique powder and fique fibers increases this temperature to 29 and 57 °C respectively. This increase in Tg values indicates that the presence of fique affects the mobility of the EP chains. However, this effect is greater for EP-UF biocomposites. It may be related to a greater reinforcing effect of the fique fibers compared to fique powder, which restricts the movement of the EP macromolecule chains. Similar results were recently reported by Hidalgo and Correa for EP-NWF mat biocomposites^3^.

**Figure 8:**
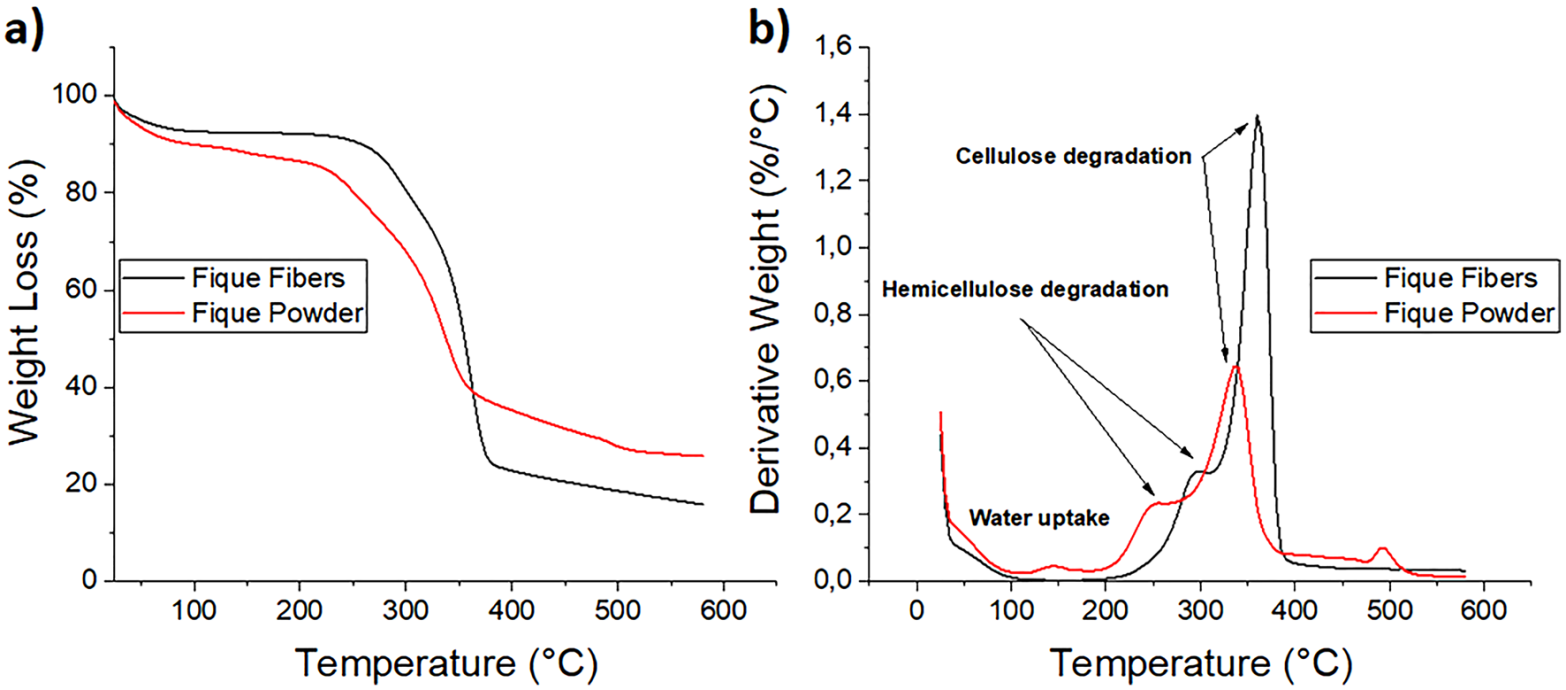
(**a**) TGA and (**b**) DTG of fique Fibers and fique powder.

**Figure 9:**
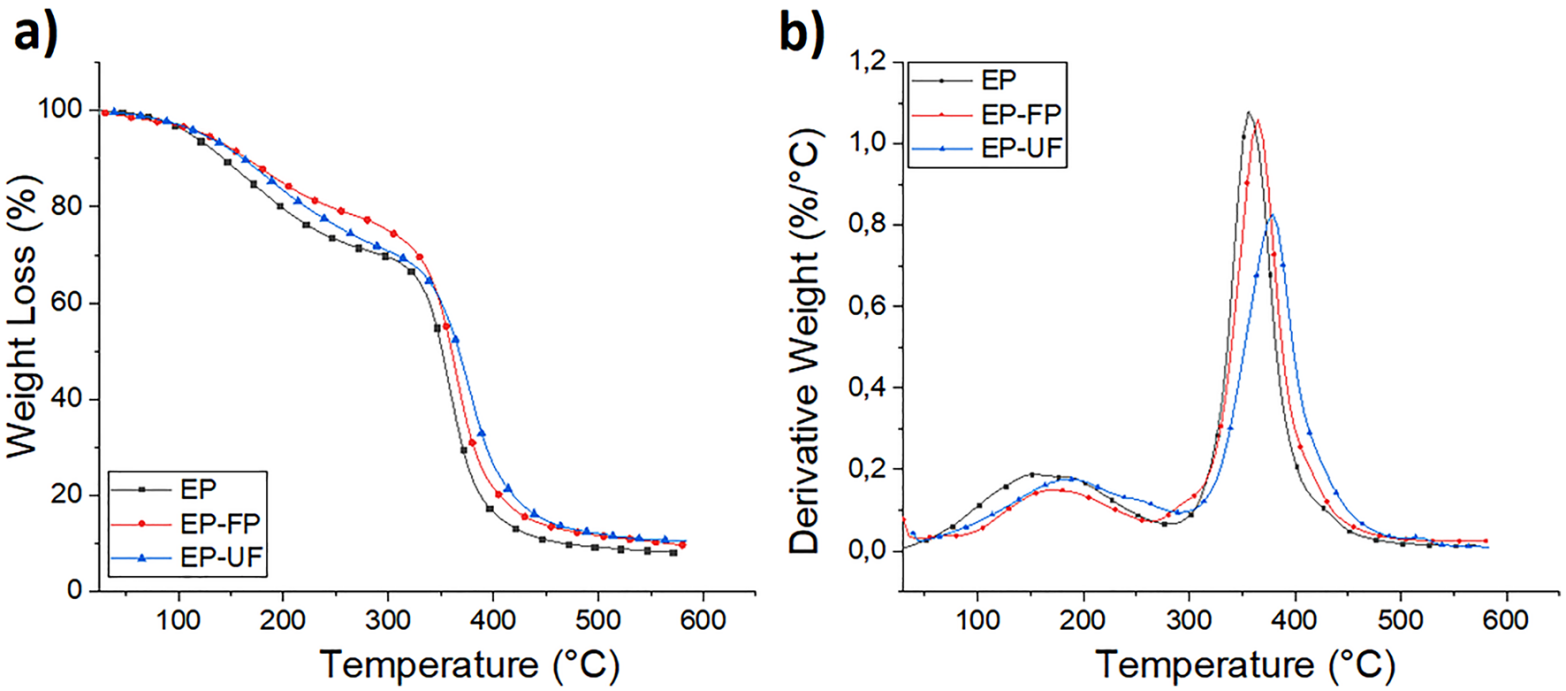
TGA of neat Epoxy resin and EP-fique Biocomposites.

Thermogravimetry (TG) and Derivative thermogravimetry over temperature (DTG) curves were used to determine the thermal stability of fique powder, fique fibers, EP and their fique biocomposites. The results are shown in Figs. 8 and 9. The thermal results from these tests are also summarized in Table 2.

**Table 2:** Thermal properties of fique, neat EP and their fique biocomposites.

Sample	DSC	TGA			
	Tg ½ Cp* (°C)	Degradation Stage	T_0_ (°C)	Tvmax(°C)	Residual char (%)
Fique Fibers (FF)	–	1	267	298	18,8
		2	343	361	
Fique Powder (FP)		1	227	256	22,8
		2	319	337	
Epoxy resin (EP)	22	1	102	151	8,4
		2	333	356	
Epoxy resin-Fique Powder (EP-FP)	29	1	109	169	10,8
		2	337	365	
Epoxy resin-unidirectional Fique fiber mat (EP-UF)	57	1	122	180	10,1
		2	341	370	

*Tg was determinate by the half step temperature.

For fique fibers and powder, three main sectors of mass loss were observed. The first zone located between 60 and 100° C is related to moisture evaporation, that was present in the surface of the sample. The other two were located between 250–350 °C and 350–600 °C and are related to hemicellulose and cellulose degradation respectively. The onset degradation temperatures (To) of these regions are higher for fique fibers compared to fique powder. This is related to the fique powder coming from the outer zone of the fique fibers and being produced on an industrial scale during the detangled process of the fibers. This fique powder may contain waxes and cellular content, which could reduce its thermal stability. The DTG curves show a first peak related to the maximum weight loss rate temperature (Tmax) of hemicellulose located at 256 °C for fique powder and 298 °C for fique fibers. The second peak is related to the Tmax of cellulose is higher for fique fibers.

The height of the peaks observed in the DTG curves are also related with the concentrations of several materials, as polymeric blends^21^ and lignocellulosic residues^22^. From the DTG curves, it is observed that the height of the peak related to cellulose decomposition is higher for fique fibers compared to fique powder, which could be related to a higher cellulose content in fique fibers.

Figure 9a shows that EP decomposition and their fique biocomposites occur in a two-step process Thus indicating that these materials have a similar thermal degradation behaviour. The first degradation step occurs between 90 and 200 °C, attributed to the decomposition of small molecules of the EP. The second degradation step, observed in the range of 250–500° C, shows the decomposition of themain polymeric chain^3,23^. For both degradation steps, To values of fique biocomposites were higher than To values of neat EP. The result has already been observed and reported for EP-NWF mat biocomposites^3^.

Tmax values of fique biocomposites were higher than those observed for neat EP (Fig. 8b). They show that fique powder and fique fibers improve the thermal stability of EP. These results have been reported for several biocomposites materials based on thermoplastic ^5,24,25^ and thermosetting matrices ^3,11^ and can be considered as an advantage in the performance and service temperature of these materials.

### Morphology

**Figure 10:**
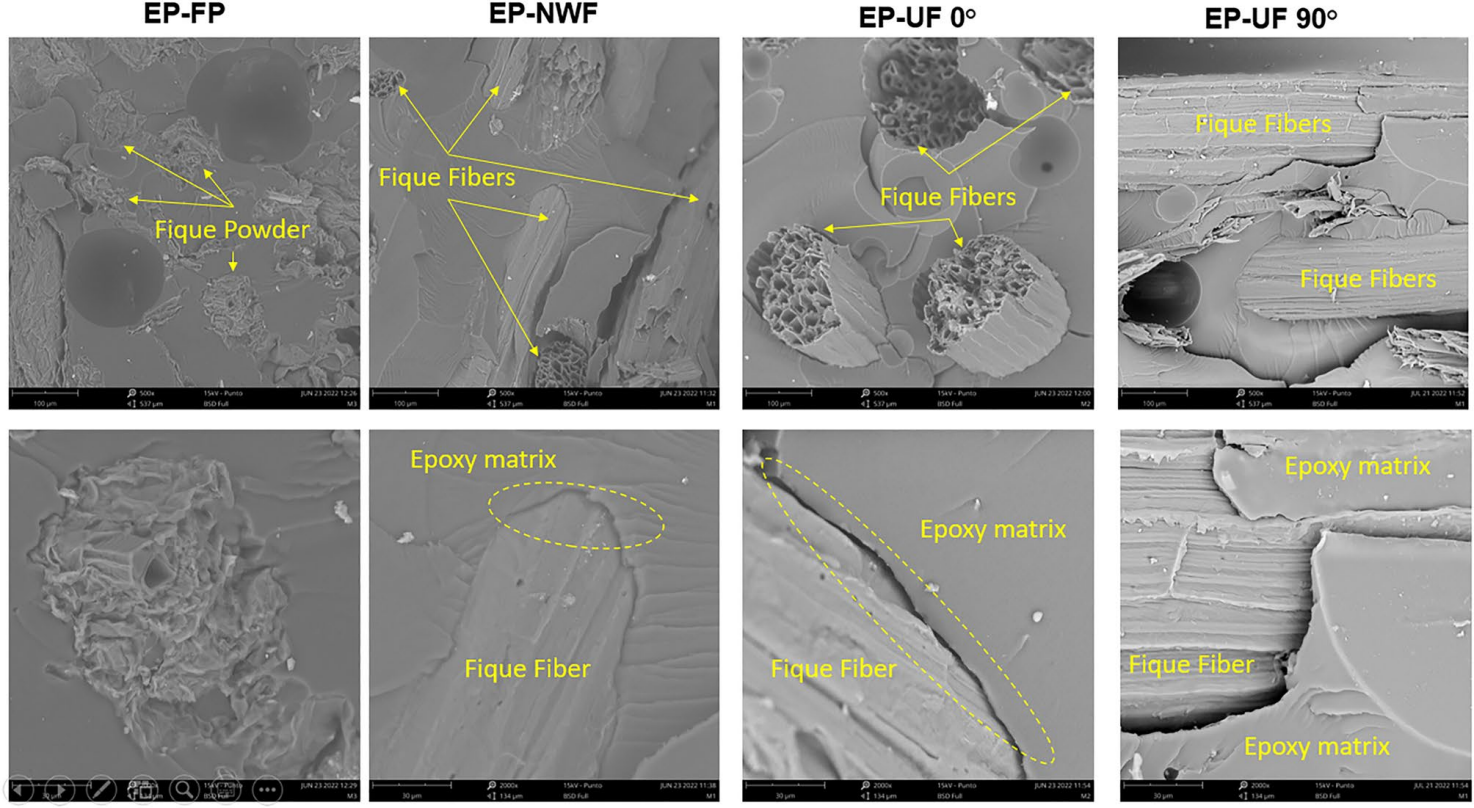
SEM micrographs of the cross-sectional area of EP-fique biocomposites.

SEM micrographs of the EP-fique biocomposites are shown in Fig. 10. In these images it can be observed that the fique type used (powder or fiber) and the fique mat arrangement generate changes in the biocomposites morphology.

For EP-FP biocomposites, semicircular and elongated fique powder particles were observed within EP matrix with diameters around 100 µm. The fique mat used and fiber orientation, generates changes in the direction and dispersion of the fibers within the matrix. For EP-UF 0° and EP-UF 90° biocomposites, the fibers are aligned perpendicular and parallel to the fracture plane respectively, while in the EP-NWF composites, the fibers are in a two-dimensional random arrangement. In addition, the interfacial spaces observed (yellow circles in Fig. 10) indicate a weak interfacial bonding between fique and Epoxy matrix and could be related to the observed decreasing in deformation at break ("Mechanical properties” section).

### Product application

**Figure 11:**
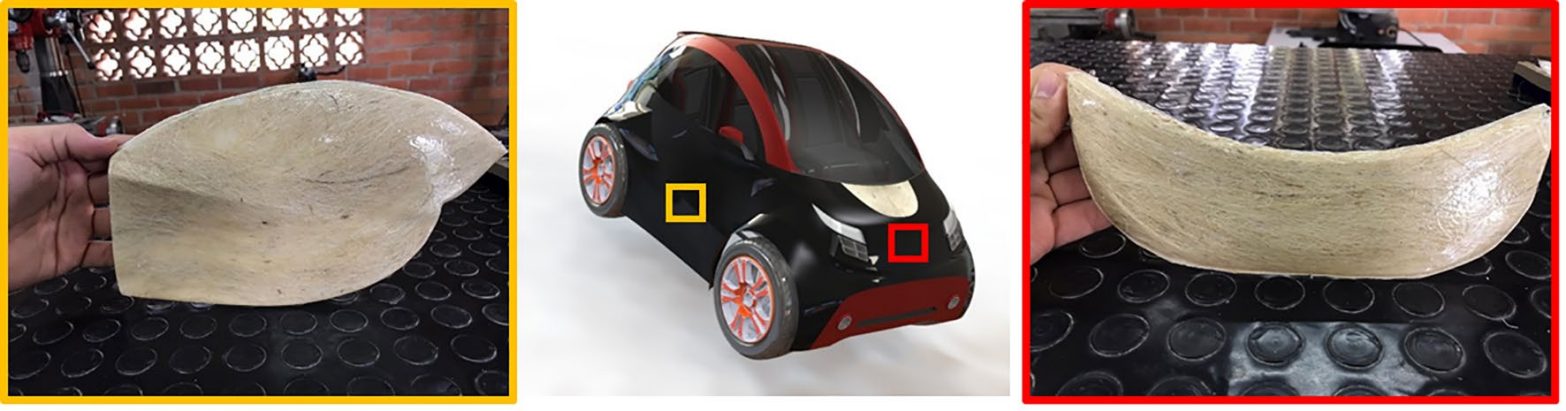
Automotive parts (door and hood) produced from EP-UF 0° biocomposites (scale 1:5).

The design process of the car parts was carried out using the SolidWorks surfaces module It allows the generation of sketches of the main lines and coatings with surfaces of complex geometries that would be impossible to generate otherwise. One of the benefits of the surface module is that a 3D modeling process can be carried out, in which the main perspectives of an object are used. In turn each is arranged in its corresponding plane, to generate a lineal system in space, whose coordinates are affected by each corresponding plane. In so doing, the side, top, and front views could be sketched separately and stitched to generate complex sketches. For this study, a complete scaled car was designed, and two parts (door and hood) were manufactured using in EP-UF 0° biocomposites (Fig. 11). This selection was due to higher modulus and resistance achieved during tensile and flexural tests. The fique fibers oriented parallel to the applied load during the mechanical tests and the stiffening effect of the fibers allowed to obtain a rigid biocomposite material with tensile and flexural strength (36.6 and 21.2 MPa, respectively) comparable to other automotive commercial products reported in the literature^18^. Also, the resin film infusion processing could generate a considerable pressure within the mold, which then allows for a homogeneous compaction of fique fibers and EP during the curing. In this way, it is possible to obtain scaled car parts with uniform thicknesses and with good homogeneity, which can be scaled for car parts production.

## Conclusions

This study aimed to research the use of natural fique fibers and its processing byproducts for the Epoxy Resin based biocomposites production, using resin film infusion, and the effects of the natural fiber form and orientation on the mechanical and thermal properties of these materials. Given the results presented in this paper, the following conclusion can be drawn:

Mechanical characterization indicated that aligned fique fibers at an orientation angle of 0° increase Tensile and flexural modulus up to 5700% and 1100% respectively in comparison with neat EP. Also, fique powder (a by-product from fique fibers processing) and fique fibers oriented at 90° acts as fillers for the Epoxy matrix while the fique fibers oriented at 0° reinforce EP matrix increasing the tensile strength up to 277% and flexural strength up to 820%. This behavior is related with a homogeneous distribution of the load due to fibers orientation and a higher aspect ratio (L/D) which may contribute to higher reinforcing efficacy for EP-UF 0° biocomposites. Thus, the mechanical performance of EP-fique biocomposites is highly dependent on the structure and the fibers orientation angle.

Thermal characterization shows that fique powder and fique fibers restrict the movement of the EP macromolecule chains and enhance the thermal stability of EP. These results can be considered an advantage for the performance and service temperature of fique based biocomposites. Tensile and flexural mechanical characterization showed that the incorporation of fique powder and fibers induced a stiffening effect of the EP matrix.

SEM images showed that the fique form used (powder or fiber) and the fiber orientation (0° or 90°) generated changes of biocomposites morphology and interfacial spaces, that indicate a weak interfacial bonding between fique and Epoxy matrix. Thus, they could generate a decrease in deformation at break, as observed in this paper.

The resin film infusion process method enables the production of biocomposites sheets and prototypes of automobile parts with uniform thicknesses and good homogeneity. These parts could be manufactured for full-scale parts production. These findings clearly show the possibility of considering EP-fique biocomposites as alternative materials for applications in the automotive sector as natural-fiber based products, and could increase the interest in researching and developing products from fique Powders and other natural fibers processing byproducts reducing their accumulation in soil and landfills, environmental concerns and generate a positive impact on regional economies, technological development and expand the range of fique applications.

## Supplementary Information

Supplementary Information.

## Data Availability

All data generated or analyzed during this study are included in this published article and its supplementary information files.
