# Supplementary material for: Effect of fique fibers and its processing by-products on morphology, thermal and mechanical properties of epoxy based biocomposites
Source: Sci Rep. 2022 Sep 7;12:15143. doi: 10.1038/s41598-022-18934-x (PMC9452679; doi:10.1038/s41598-022-18934-x)
Supplement: Supplementary file 1 — Supplementary Information. [file 41598_2022_18934_MOESM1_ESM.docx]

# Effect of Fique fibers and its processing by-products on morphology, thermal and mechanical properties of Epoxy based biocomposites

Nicolas Centeno-Mesa^1^, Oscar Lombana-Toro^1^, Juan Pablo Correa-Aguirre^1(*)^, Miguel Angel Hidalgo-Salazar^1^

^1^ Research Group for Manufacturing Technologies GITEM, Universidad Autónoma de Occidente, Cali, Colombia

**DATASET OF MECHANICAL PROPERTIES**

The following dataset corresponds to tensile and flexural tests average curves.

**Notes:**

-The results were taken as the average of five simples.

- Flexural tests were performed up to 5% strain following ASTM D 790-17 standard.

-EP: Epoxy Resin, EP-FP: Biocomposite Epoxy Resin-Fique Powder, EP-NWF: Biocomposite Epoxy Resin-nonwoven industrial fique fiber mats, EP-UF 0°: Biocomposite Epoxy Resin- unidirectional fique fiber mat parallel oriented to the applied load. EP-UF 90°: Biocomposite Epoxy Resin- unidirectional fique fiber mat perpendicularly oriented to the applied load

**TENSILE TESTS**

| **EP** | | **EP-FP** | | **EP-NWF** | | **EP-UF 0°** | | **EP-UF 90°** | |
| --- | --- | --- | --- | --- | --- | --- | --- | --- | --- |
| Deformation (%) | Tensile strength (MPa) | Deformation (%) | Tensile strength (MPa) | Deformation (%) | Tensile strength (MPa) | Deformation (%) | Tensile strength (MPa) | Deformation (%) | Tensile strength (MPa) |
| 0 | 0 | 0 | 0 | 0 | 0 | 0 | 0 | 0 | 0 |
| 0,46893 | 0,4842 | 0,02905 | 0,36 | 0,10292 | 0,91815 | 0,02054 | 1,23049 | 0,01122 | 0,12016 |
| 0,82063 | 0,77469 | 0,06968 | 0,8 | 0,24014 | 2,58363 | 0,07233 | 2,58363 | 0,02244 | 0,24032 |
| 2,22743 | 1,25844 | 0,12767 | 1,36 | 0,44597 | 4,82562 | 0,14485 | 4,42879 | 0,03365 | 0,36026 |
| 3,28253 | 1,64547 | 0,16825 | 1,72 | 0,68611 | 7,53737 | 0,27981 | 6,7654 | 0,04487 | 0,47795 |
| 4,5721 | 2,12927 | 0,23782 | 2,36 | 0,75472 | 8,71174 | 0,39405 | 8,61 | 0,05609 | 0,59497 |
| 5,86166 | 2,46775 | 0,27264 | 2,72 | 1,02916 | 10,59075 | 0,49792 | 10,20858 | 0,06731 | 0,71403 |
| 6,21336 | 2,56446 | 0,34218 | 3,32 | 1,2693 | 12,02135 | 0,60179 | 11,80716 | 0,07852 | 0,83216 |
| 7,62016 | 2,85444 | 0,41173 | 3,92 | 1,47513 | 13,72954 | 0,7784 | 14,38936 | 0,08974 | 0,94965 |
| 9,73036 | 3,19252 | 0,49281 | 4,52 | 1,61235 | 14,62633 | 0,93423 | 16,72569 | 0,10096 | 1,09315 |
| 13,13013 | 3,6753 | 0,59123 | 5,2 | 1,9554 | 15,92883 | 1,18394 | 18,93766 | 0,11218 | 1,24125 |
| 14,7714 | 3,91672 | 0,71283 | 6,08 | 1,9554 | 16,44128 | 1,47566 | 20,04136 | 0,12339 | 1,3492 |
| 16,76436 | 4,15797 | 0,77077 | 6,56 | 2,12693 | 15,05338 | 1,72569 | 21,02255 | 0,13461 | 1,39904 |
| 19,34349 | 4,44738 | 0,83433 | 6,8 | 2,33276 | 14,04982 | 1,92364 | 21,75831 | 0,14583 | 1,51706 |
| 21,33646 | 4,64018 | 0,91522 | 7,12 | 2,33276 | 13,51601 | 2,16334 | 22,37042 | 0,15705 | 1,63578 |
| 24,73623 | 5,02608 | 0,96149 | 7,36 | 3,15609 | 7,04626 | 2,37179 | 22,85987 | 0,16826 | 1,75457 |
| 26,49472 | 5,17056 | 1,07115 | 7,6 | 3,32762 | 5,44484 | 2,63232 | 23,59477 | 0,17948 | 1,87379 |
| 29,89449 | 5,45957 | 1,19805 | 7,76 |  |  | 2,87202 | 24,20688 | 0,1907 | 2,06413 |
| 32,59086 | 5,70048 | 1,29043 | 8 |  |  | 3,02829 | 24,82013 | 0,20192 | 2,16621 |
| 34,11489 | 5,79662 | 1,41154 | 8,12 |  |  | 3,28891 | 25,18579 | 0,21313 | 2,26804 |
| 36,22509 | 5,98937 | 1,56731 | 8,36 |  |  | 3,52858 | 25,92098 | 0,22435 | 2,36955 |
| 38,45252 | 6,18206 | 1,72877 | 8,48 |  |  | 3,7371 | 26,16428 | 0,23557 | 2,47399 |
| 40,79719 | 6,3747 | 1,89026 | 8,64 |  |  | 4,06026 | 26,65217 | 0,24679 | 2,57974 |
| 43,49355 | 6,61561 | 1,94795 | 8,72 |  |  | 4,21653 | 27,26541 | 0,258 | 2,68558 |
| 46,54162 | 6,8079 | 2,10362 | 8,8 |  |  | 4,46666 | 27,87738 | 0,26922 | 2,78789 |
| 48,88628 | 7,00054 | 2,22471 | 8,88 |  |  | 4,59168 | 28,36798 | 0,28044 | 2,88688 |
| 50,52755 | 7,09663 | 2,34003 | 8,96 |  |  | 5,05032 | 29,22324 | 0,29166 | 2,9852 |
| 51,93435 | 7,24128 | 2,43809 | 9,08 |  |  | 5,45666 | 30,69461 | 0,30287 | 3,0833 |
| 53,69285 | 7,28887 | 2,55344 | 9,2 |  |  | 5,79018 | 31,42851 | 0,31409 | 3,18075 |
| 56,15475 | 7,48145 | 2,65724 | 9,28 |  |  | 6,20705 | 32,5305 | 0,32531 | 3,28086 |
| 58,49941 | 7,62564 | 2,74372 | 9,32 |  |  | 6,6448 | 33,50913 | 0,33653 | 3,38087 |
| 60,72685 | 7,76989 |  |  |  |  | 7,01991 | 34,73477 | 0,34774 | 3,48101 |
| 63,07151 | 7,86564 |  |  |  |  | 7,33258 | 35,46896 | 0,35896 | 3,58234 |
| 65,06448 | 8,01 |  |  |  |  | 7,69743 | 36,07935 | 0,37018 | 3,68844 |
| 67,05744 | 8,15437 |  |  |  |  | 8,04139 | 36,81311 | 0,3814 | 3,79454 |
| 67,87808 | 8,20241 |  |  |  |  | 8,2395 | 36,93348 | 0,39261 | 3,89648 |
| 68,81594 | 8,2504 |  |  |  |  | 8,48966 | 37,42236 | 0,40383 | 3,99212 |
| 70,45721 | 8,34649 |  |  |  |  | 8,80243 | 37,78732 | 0,41505 | 4,08729 |
| 72,33294 | 8,44247 |  |  |  |  | 8,95883 | 37,90826 | 0,42627 | 4,18242 |
| 74,44314 | 8,63522 |  |  |  |  | 9,00103 | 36,06152 | 0,43748 | 4,27916 |
| 76,90504 | 8,77935 |  |  |  |  |  |  | 0,4487 | 4,44051 |
| 78,89801 | 8,92372 |  |  |  |  |  |  | 0,45992 | 4,54315 |
| 81,24267 | 9,06791 |  |  |  |  |  |  | 0,47114 | 4,64606 |
| 83,70457 | 9,26049 |  |  |  |  |  |  | 0,48235 | 4,74712 |
| 87,3388 | 9,50094 |  |  |  |  |  |  | 0,49357 | 4,84861 |
|  |  |  |  |  |  |  |  | 0,50479 | 4,9495 |
|  |  |  |  |  |  |  |  | 0,51601 | 5,04921 |
|  |  |  |  |  |  |  |  | 0,52722 | 5,14865 |
|  |  |  |  |  |  |  |  | 0,53844 | 5,24763 |
|  |  |  |  |  |  |  |  | 0,54966 | 5,34535 |
|  |  |  |  |  |  |  |  | 0,56088 | 5,44267 |
|  |  |  |  |  |  |  |  | 0,57209 | 5,54033 |
|  |  |  |  |  |  |  |  | 0,58331 | 5,63788 |
|  |  |  |  |  |  |  |  | 0,59453 | 5,73525 |
|  |  |  |  |  |  |  |  | 0,60575 | 5,83189 |
|  |  |  |  |  |  |  |  | 0,61696 | 5,92667 |
|  |  |  |  |  |  |  |  | 0,62818 | 6,02049 |
|  |  |  |  |  |  |  |  | 0,6394 | 6,11409 |
|  |  |  |  |  |  |  |  | 0,65062 | 6,2075 |
|  |  |  |  |  |  |  |  | 0,66183 | 6,2997 |
|  |  |  |  |  |  |  |  | 0,67305 | 6,39065 |
|  |  |  |  |  |  |  |  | 0,68427 | 6,47989 |
|  |  |  |  |  |  |  |  | 0,69549 | 6,56788 |
|  |  |  |  |  |  |  |  | 0,7067 | 6,65628 |
|  |  |  |  |  |  |  |  | 0,71792 | 6,74485 |
|  |  |  |  |  |  |  |  | 0,72914 | 6,83466 |
|  |  |  |  |  |  |  |  | 0,74036 | 6,92178 |
|  |  |  |  |  |  |  |  | 0,75157 | 7,00809 |
|  |  |  |  |  |  |  |  | 0,76279 | 7,09327 |
|  |  |  |  |  |  |  |  | 0,77401 | 7,17802 |
|  |  |  |  |  |  |  |  | 0,78523 | 7,26164 |
|  |  |  |  |  |  |  |  | 0,79644 | 7,34437 |
|  |  |  |  |  |  |  |  | 0,80766 | 7,42533 |
|  |  |  |  |  |  |  |  | 0,81888 | 7,50513 |
|  |  |  |  |  |  |  |  | 0,8301 | 7,58494 |
|  |  |  |  |  |  |  |  | 0,84131 | 7,66467 |
|  |  |  |  |  |  |  |  | 0,85253 | 7,74427 |
|  |  |  |  |  |  |  |  | 0,86375 | 7,82285 |
|  |  |  |  |  |  |  |  | 0,87497 | 7,90005 |
|  |  |  |  |  |  |  |  | 0,88618 | 7,97501 |
|  |  |  |  |  |  |  |  | 0,8974 | 8,04733 |
|  |  |  |  |  |  |  |  | 0,90862 | 8,11924 |
|  |  |  |  |  |  |  |  | 0,91984 | 8,1921 |
|  |  |  |  |  |  |  |  | 0,93105 | 8,26444 |
|  |  |  |  |  |  |  |  | 0,94227 | 8,33581 |
|  |  |  |  |  |  |  |  | 0,95349 | 8,4055 |
|  |  |  |  |  |  |  |  | 0,96471 | 8,47393 |
|  |  |  |  |  |  |  |  | 0,97592 | 8,54188 |
|  |  |  |  |  |  |  |  | 0,98714 | 8,60862 |
|  |  |  |  |  |  |  |  | 0,99836 | 8,6735 |
|  |  |  |  |  |  |  |  | 1,00958 | 8,73792 |
|  |  |  |  |  |  |  |  | 1,02079 | 8,80209 |
|  |  |  |  |  |  |  |  | 1,03201 | 8,86469 |
|  |  |  |  |  |  |  |  | 1,04323 | 8,92545 |
|  |  |  |  |  |  |  |  | 1,05445 | 8,98397 |
|  |  |  |  |  |  |  |  | 1,06566 | 9,04254 |
|  |  |  |  |  |  |  |  | 1,07688 | 9,09982 |
|  |  |  |  |  |  |  |  | 1,0881 | 9,1567 |
|  |  |  |  |  |  |  |  | 1,09932 | 9,2126 |
|  |  |  |  |  |  |  |  | 1,11053 | 9,26717 |
|  |  |  |  |  |  |  |  | 1,12175 | 9,32061 |
|  |  |  |  |  |  |  |  | 1,13297 | 9,37174 |
|  |  |  |  |  |  |  |  | 1,14419 | 9,42033 |
|  |  |  |  |  |  |  |  | 1,1554 | 9,46602 |
|  |  |  |  |  |  |  |  | 1,16662 | 9,51172 |
|  |  |  |  |  |  |  |  | 1,17784 | 9,55824 |
|  |  |  |  |  |  |  |  | 1,18906 | 9,60657 |
|  |  |  |  |  |  |  |  | 1,20027 | 9,65487 |
|  |  |  |  |  |  |  |  | 1,21149 | 9,70211 |
|  |  |  |  |  |  |  |  | 1,22271 | 9,74644 |
|  |  |  |  |  |  |  |  | 1,23393 | 9,79051 |
|  |  |  |  |  |  |  |  | 1,24514 | 9,83451 |
|  |  |  |  |  |  |  |  | 1,25636 | 9,87836 |
|  |  |  |  |  |  |  |  | 1,26758 | 9,92113 |
|  |  |  |  |  |  |  |  | 1,2788 | 9,96279 |
|  |  |  |  |  |  |  |  | 1,29001 | 10,00355 |
|  |  |  |  |  |  |  |  | 1,30123 | 10,04383 |
|  |  |  |  |  |  |  |  | 1,31245 | 10,08081 |
|  |  |  |  |  |  |  |  | 1,32367 | 10,11517 |
|  |  |  |  |  |  |  |  | 1,33489 | 10,14944 |
|  |  |  |  |  |  |  |  | 1,3461 | 10,18216 |
|  |  |  |  |  |  |  |  | 1,35732 | 10,2128 |
|  |  |  |  |  |  |  |  | 1,36854 | 10,24257 |
|  |  |  |  |  |  |  |  | 1,37976 | 10,2718 |
|  |  |  |  |  |  |  |  | 1,39097 | 10,30012 |
|  |  |  |  |  |  |  |  | 1,40219 | 10,32646 |
|  |  |  |  |  |  |  |  | 1,41341 | 10,3505 |
|  |  |  |  |  |  |  |  | 1,42463 | 10,37452 |
|  |  |  |  |  |  |  |  | 1,43584 | 10,39761 |
|  |  |  |  |  |  |  |  | 1,44706 | 10,41969 |
|  |  |  |  |  |  |  |  | 1,45828 | 10,41305 |
|  |  |  |  |  |  |  |  | 1,4695 | 10,40331 |
|  |  |  |  |  |  |  |  | 1,48071 | 10,39329 |
|  |  |  |  |  |  |  |  | 1,49193 | 10,38315 |
|  |  |  |  |  |  |  |  | 1,50315 | 10,37319 |
|  |  |  |  |  |  |  |  | 1,51437 | 10,36322 |
|  |  |  |  |  |  |  |  | 1,52558 | 10,35325 |
|  |  |  |  |  |  |  |  | 1,5368 | 10,34043 |
|  |  |  |  |  |  |  |  | 1,54802 | 10,32567 |
|  |  |  |  |  |  |  |  | 1,55924 | 10,31091 |
|  |  |  |  |  |  |  |  | 1,57045 | 10,29432 |
|  |  |  |  |  |  |  |  | 1,58167 | 10,27356 |
|  |  |  |  |  |  |  |  | 1,59289 | 10,25236 |
|  |  |  |  |  |  |  |  | 1,60411 | 10,23021 |
|  |  |  |  |  |  |  |  | 1,61532 | 10,20769 |
|  |  |  |  |  |  |  |  | 1,62654 | 10,18505 |
|  |  |  |  |  |  |  |  | 1,63776 | 10,1618 |
|  |  |  |  |  |  |  |  | 1,64898 | 10,13854 |
|  |  |  |  |  |  |  |  | 1,66019 | 10,11247 |
|  |  |  |  |  |  |  |  | 1,67141 | 10,08387 |
|  |  |  |  |  |  |  |  | 1,68263 | 10,05499 |
|  |  |  |  |  |  |  |  | 1,69385 | 10,02604 |
|  |  |  |  |  |  |  |  | 1,70506 | 9,9911 |
|  |  |  |  |  |  |  |  | 1,71628 | 9,93941 |
|  |  |  |  |  |  |  |  | 1,7275 | 9,88697 |
|  |  |  |  |  |  |  |  | 1,73872 | 9,83304 |
|  |  |  |  |  |  |  |  | 1,74993 | 9,77861 |
|  |  |  |  |  |  |  |  | 1,76115 | 9,72418 |
|  |  |  |  |  |  |  |  | 1,77237 | 9,66845 |
|  |  |  |  |  |  |  |  | 1,78359 | 9,60988 |
|  |  |  |  |  |  |  |  | 1,7948 | 9,55113 |
|  |  |  |  |  |  |  |  | 1,80602 | 9,49125 |
|  |  |  |  |  |  |  |  | 1,81724 | 9,42892 |
|  |  |  |  |  |  |  |  | 1,82846 | 9,36659 |
|  |  |  |  |  |  |  |  | 1,83967 | 9,30279 |
|  |  |  |  |  |  |  |  | 1,85089 | 9,23835 |
|  |  |  |  |  |  |  |  | 1,86211 | 9,17361 |
|  |  |  |  |  |  |  |  | 1,87333 | 9,10888 |
|  |  |  |  |  |  |  |  | 1,88454 | 9,04414 |
|  |  |  |  |  |  |  |  | 1,89576 | 8,97832 |
|  |  |  |  |  |  |  |  | 1,90698 | 8,91044 |
|  |  |  |  |  |  |  |  | 1,9182 | 8,80736 |
|  |  |  |  |  |  |  |  | 1,92941 | 8,70428 |
|  |  |  |  |  |  |  |  | 1,94063 | 8,59808 |
|  |  |  |  |  |  |  |  | 1,95185 | 8,47254 |
|  |  |  |  |  |  |  |  | 1,96307 | 8,34394 |
|  |  |  |  |  |  |  |  | 1,97428 | 8,21535 |
|  |  |  |  |  |  |  |  | 1,9855 | 8,07871 |
|  |  |  |  |  |  |  |  | 1,99672 | 7,93341 |
|  |  |  |  |  |  |  |  | 2,00794 | 7,78811 |
|  |  |  |  |  |  |  |  | 2,01915 | 7,64281 |
|  |  |  |  |  |  |  |  | 2,03037 | 7,49751 |
|  |  |  |  |  |  |  |  | 2,04159 | 7,35222 |
|  |  |  |  |  |  |  |  | 2,05281 | 7,20692 |
|  |  |  |  |  |  |  |  | 2,06402 | 7,06162 |
|  |  |  |  |  |  |  |  | 2,07524 | 6,91632 |
|  |  |  |  |  |  |  |  | 2,08646 | 6,77102 |
|  |  |  |  |  |  |  |  | 2,09768 | 6,62572 |
|  |  |  |  |  |  |  |  | 2,10889 | 6,48043 |
|  |  |  |  |  |  |  |  | 2,12011 | 6,33513 |
|  |  |  |  |  |  |  |  | 2,13133 | 6,18983 |
|  |  |  |  |  |  |  |  | 2,14255 | 6,04453 |
|  |  |  |  |  |  |  |  | 2,15376 | 5,89923 |
|  |  |  |  |  |  |  |  | 2,16498 | 5,75393 |
|  |  |  |  |  |  |  |  | 2,1762 | 5,60864 |
|  |  |  |  |  |  |  |  | 2,18742 | 5,46334 |
|  |  |  |  |  |  |  |  | 2,19863 | 5,31804 |
|  |  |  |  |  |  |  |  | 2,20985 | 5,17274 |

**FLEXURAL TESTS**

| **EP** | | **EP-FP** | | **EP-FNW** | | **EP-UF 0°** | | **EP-UF 90°** | |
| --- | --- | --- | --- | --- | --- | --- | --- | --- | --- |
| Deformation (%) | Tensile strength (MPa) | Deformation (%) | Tensile strength (MPa) | Deformation (%) | Tensile strength (MPa) | Deformation (%) | Tensile strength (MPa) | Deformation (%) | Tensile strength (MPa) |
| 0 | 0 | 0 | 0 | 0 | 0 | 0 | 0 | 0 | 0 |
| 0,08392 | 0,08621 | 0,04892 | 0,49219 | 0,05175 | 0,20206 | 0,04808 | 0,58027 | 0,01166 | 0,03147 |
| 0,2028 | 0,14009 | 0,11868 | 0,92969 | 0,31048 | 1,55876 | 0,09081 | 0,99488 | 0,02331 | 0,06294 |
| 0,3007 | 0,20474 | 0,29302 | 1,91406 | 0,56274 | 2,85773 | 0,14423 | 1,49245 | 0,03497 | 0,09441 |
| 0,41259 | 0,29095 | 0,38373 | 2,51563 | 0,81501 | 4,04124 | 0,21902 | 2,15594 | 0,04662 | 0,12587 |
| 0,5035 | 0,36638 | 0,53017 | 3,33594 | 1,06727 | 5,13814 | 0,27244 | 2,65352 | 0,05828 | 0,15734 |
| 0,62238 | 0,46336 | 0,71834 | 4,15625 | 1,33247 | 6,20619 | 0,34722 | 3,48257 | 0,06993 | 0,18881 |
| 0,69231 | 0,52802 | 0,86475 | 4,92188 | 1,58473 | 7,15876 | 0,41667 | 4,14597 | 0,08159 | 0,22028 |
| 0,88811 | 0,67888 | 1,03201 | 5,63281 | 1,83053 | 7,96701 | 0,53419 | 5,14129 | 0,09324 | 0,25175 |
| 1,02797 | 0,77586 | 1,21322 | 6,45313 | 2,07633 | 8,74639 | 0,63568 | 5,88801 | 0,1049 | 0,29301 |
| 1,12587 | 0,82974 | 1,40136 | 7,21875 | 2,34799 | 9,52577 | 0,74252 | 6,80037 | 0,11655 | 0,34779 |
| 1,27273 | 0,91595 | 1,58945 | 7,875 | 2,58085 | 10,10309 | 0,84402 | 7,54709 | 0,12821 | 0,40256 |
| 1,41259 | 1,00216 | 1,75664 | 8,47656 | 2,85252 | 10,68041 | 0,98291 | 8,70833 | 0,13986 | 0,45734 |
| 1,57343 | 1,10991 | 1,88894 | 8,80469 | 3,10479 | 11,17113 | 1,16987 | 9,87036 | 0,15152 | 0,51212 |
| 1,70629 | 1,19612 | 2,01433 | 9,24219 | 3,34411 | 11,54639 | 1,27671 | 10,36882 | 0,16317 | 0,5669 |
| 1,8042 | 1,25 | 2,16058 | 9,67969 | 3,58991 | 11,92165 | 1,44765 | 11,36503 | 0,17483 | 0,62168 |
| 1,90909 | 1,30388 | 2,36947 | 10,22656 | 3,84217 | 12,21031 | 1,63462 | 12,3615 | 0,18648 | 0,67646 |
| 1,99301 | 1,33621 | 2,49478 | 10,5 | 4,10091 | 12,41237 | 1,82692 | 13,27528 | 0,19814 | 0,73124 |
| 2,13986 | 1,42241 | 2,63403 | 10,82813 | 4,35317 | 12,61443 | 1,96581 | 13,93983 | 0,20979 | 0,77133 |
| 2,28671 | 1,49784 | 2,79411 | 11,10156 | 4,59897 | 12,75876 | 2,08333 | 14,43847 | 0,22145 | 0,80862 |
| 2,41958 | 1,57328 | 2,93327 | 11,26563 | 4,85123 | 12,90309 | 2,32372 | 15,35304 | 0,2331 | 0,84592 |
| 2,61538 | 1,64871 | 3,07246 | 11,48438 | 4,96119 | 12,87423 | 2,46261 | 15,85203 | 0,24476 | 0,88322 |
| 2,9021 | 1,74569 | 3,25338 | 11,70313 | 5 | 12,87423 | 2,59615 | 16,26815 | 0,25641 | 0,92051 |
| 3,04196 | 1,79957 | 3,46208 | 11,86719 |  |  | 2,72436 | 16,68418 | 0,26807 | 0,95781 |
| 3,18881 | 1,85345 | 3,58731 | 11,97656 |  |  | 2,87927 | 17,18343 | 0,27972 | 0,9951 |
| 3,36364 | 1,92888 | 3,69863 | 12,08594 |  |  | 3,01282 | 17,51677 | 0,29138 | 1,0324 |
| 3,57343 | 1,99353 | 3,83761 | 11,86719 |  |  | 3,19979 | 18,01655 | 0,30303 | 1,06939 |
| 3,75524 | 2,03664 | 4,0183 | 11,64844 |  |  | 3,37073 | 18,43329 | 0,31469 | 1,10552 |
| 3,97203 | 2,09052 | 4,12253 | 11,48438 |  |  | 3,54167 | 18,85003 | 0,32634 | 1,14166 |
| 4,23077 | 2,17672 | 4,4352 | 10,9375 |  |  | 3,69658 | 19,18372 | 0,338 | 1,17779 |
| 4,45455 | 2,21983 | 4,51164 | 10,82813 |  |  | 3,84081 | 19,43445 | 0,34965 | 1,21392 |
| 4,6014 | 2,25216 | 4,60196 | 10,66406 |  |  | 4,0438 | 19,85172 | 0,36131 | 1,25005 |
| 4,7972 | 2,28448 | 4,71314 | 10,5 |  |  | 4,18803 | 20,10245 | 0,37296 | 1,28618 |
| 4,90909 | 2,30603 | 4,85214 | 10,33594 |  |  | 4,33226 | 20,35318 | 0,38462 | 1,32231 |
| 4,99301 | 2,30603 | 4,92856 | 10,17188 |  |  | 4,40705 | 20,4372 | 0,39627 | 1,35844 |
|  |  | 4,99807 | 10,11719 |  |  | 4,53526 | 20,60489 | 0,40793 | 1,40329 |
|  |  |  |  |  |  | 4,65278 | 20,7724 | 0,41958 | 1,45224 |
|  |  |  |  |  |  | 4,72222 | 20,85633 | 0,43124 | 1,50119 |
|  |  |  |  |  |  | 4,77564 | 21,02278 | 0,44289 | 1,55014 |
|  |  |  |  |  |  | 4,82906 | 21,02366 | 0,45455 | 1,59909 |
|  |  |  |  |  |  | 4,88782 | 21,10741 | 0,4662 | 1,64804 |
|  |  |  |  |  |  | 4,9359 | 21,19099 | 0,47786 | 1,69699 |
|  |  |  |  |  |  | 5 | 21,27483 | 0,48951 | 1,74594 |
|  |  |  |  |  |  |  |  | 0,50117 | 1,79524 |
|  |  |  |  |  |  |  |  | 0,51282 | 1,84769 |
|  |  |  |  |  |  |  |  | 0,52448 | 1,90014 |
|  |  |  |  |  |  |  |  | 0,53613 | 1,95259 |
|  |  |  |  |  |  |  |  | 0,54779 | 2,00503 |
|  |  |  |  |  |  |  |  | 0,55944 | 2,05748 |
|  |  |  |  |  |  |  |  | 0,5711 | 2,10993 |
|  |  |  |  |  |  |  |  | 0,58275 | 2,16238 |
|  |  |  |  |  |  |  |  | 0,59441 | 2,21483 |
|  |  |  |  |  |  |  |  | 0,60606 | 2,25818 |
|  |  |  |  |  |  |  |  | 0,61772 | 2,29315 |
|  |  |  |  |  |  |  |  | 0,62937 | 2,32811 |
|  |  |  |  |  |  |  |  | 0,64103 | 2,36308 |
|  |  |  |  |  |  |  |  | 0,65268 | 2,39804 |
|  |  |  |  |  |  |  |  | 0,66434 | 2,43301 |
|  |  |  |  |  |  |  |  | 0,67599 | 2,46797 |
|  |  |  |  |  |  |  |  | 0,68765 | 2,50294 |
|  |  |  |  |  |  |  |  | 0,6993 | 2,5379 |
|  |  |  |  |  |  |  |  | 0,71096 | 2,57615 |
|  |  |  |  |  |  |  |  | 0,72261 | 2,61462 |
|  |  |  |  |  |  |  |  | 0,73427 | 2,65308 |
|  |  |  |  |  |  |  |  | 0,74592 | 2,69154 |
|  |  |  |  |  |  |  |  | 0,75758 | 2,73 |
|  |  |  |  |  |  |  |  | 0,76923 | 2,76846 |
|  |  |  |  |  |  |  |  | 0,78089 | 2,80692 |
|  |  |  |  |  |  |  |  | 0,79254 | 2,84538 |
|  |  |  |  |  |  |  |  | 0,8042 | 2,88762 |
|  |  |  |  |  |  |  |  | 0,81585 | 2,93657 |
|  |  |  |  |  |  |  |  | 0,82751 | 2,98552 |
|  |  |  |  |  |  |  |  | 0,83916 | 3,03448 |
|  |  |  |  |  |  |  |  | 0,85082 | 3,08343 |
|  |  |  |  |  |  |  |  | 0,86247 | 3,13238 |
|  |  |  |  |  |  |  |  | 0,87413 | 3,18133 |
|  |  |  |  |  |  |  |  | 0,88578 | 3,23028 |
|  |  |  |  |  |  |  |  | 0,89744 | 3,27923 |
|  |  |  |  |  |  |  |  | 0,90909 | 3,31818 |
|  |  |  |  |  |  |  |  | 0,92075 | 3,35431 |
|  |  |  |  |  |  |  |  | 0,9324 | 3,39044 |
|  |  |  |  |  |  |  |  | 0,94406 | 3,42657 |
|  |  |  |  |  |  |  |  | 0,95571 | 3,4627 |
|  |  |  |  |  |  |  |  | 0,96737 | 3,49883 |
|  |  |  |  |  |  |  |  | 0,97902 | 3,53497 |
|  |  |  |  |  |  |  |  | 0,99068 | 3,5711 |
|  |  |  |  |  |  |  |  | 1,00233 | 3,60723 |
|  |  |  |  |  |  |  |  | 1,01399 | 3,64336 |
|  |  |  |  |  |  |  |  | 1,02564 | 3,67949 |
|  |  |  |  |  |  |  |  | 1,0373 | 3,71562 |
|  |  |  |  |  |  |  |  | 1,04895 | 3,75175 |
|  |  |  |  |  |  |  |  | 1,06061 | 3,78788 |
|  |  |  |  |  |  |  |  | 1,07226 | 3,82401 |
|  |  |  |  |  |  |  |  | 1,08392 | 3,86014 |
|  |  |  |  |  |  |  |  | 1,09557 | 3,89627 |
|  |  |  |  |  |  |  |  | 1,10723 | 3,93601 |
|  |  |  |  |  |  |  |  | 1,11888 | 3,97797 |
|  |  |  |  |  |  |  |  | 1,13054 | 4,01993 |
|  |  |  |  |  |  |  |  | 1,14219 | 4,06189 |
|  |  |  |  |  |  |  |  | 1,15385 | 4,10385 |
|  |  |  |  |  |  |  |  | 1,1655 | 4,1458 |
|  |  |  |  |  |  |  |  | 1,17716 | 4,18776 |
|  |  |  |  |  |  |  |  | 1,18881 | 4,22972 |
|  |  |  |  |  |  |  |  | 1,20047 | 4,272 |
|  |  |  |  |  |  |  |  | 1,21212 | 4,32212 |
|  |  |  |  |  |  |  |  | 1,22378 | 4,37224 |
|  |  |  |  |  |  |  |  | 1,23543 | 4,42235 |
|  |  |  |  |  |  |  |  | 1,24709 | 4,47247 |
|  |  |  |  |  |  |  |  | 1,25874 | 4,52259 |
|  |  |  |  |  |  |  |  | 1,2704 | 4,5727 |
|  |  |  |  |  |  |  |  | 1,28205 | 4,62282 |
|  |  |  |  |  |  |  |  | 1,29371 | 4,67294 |
|  |  |  |  |  |  |  |  | 1,30536 | 4,71179 |
|  |  |  |  |  |  |  |  | 1,31702 | 4,73744 |
|  |  |  |  |  |  |  |  | 1,32867 | 4,76308 |
|  |  |  |  |  |  |  |  | 1,34033 | 4,78872 |
|  |  |  |  |  |  |  |  | 1,35198 | 4,81436 |
|  |  |  |  |  |  |  |  | 1,36364 | 4,84 |
|  |  |  |  |  |  |  |  | 1,37529 | 4,86564 |
|  |  |  |  |  |  |  |  | 1,38695 | 4,89128 |
|  |  |  |  |  |  |  |  | 1,3986 | 4,91692 |
|  |  |  |  |  |  |  |  | 1,41026 | 4,95385 |
|  |  |  |  |  |  |  |  | 1,42191 | 4,99231 |
|  |  |  |  |  |  |  |  | 1,43357 | 5,03077 |
|  |  |  |  |  |  |  |  | 1,44522 | 5,06923 |
|  |  |  |  |  |  |  |  | 1,45688 | 5,10769 |
|  |  |  |  |  |  |  |  | 1,46853 | 5,14615 |
|  |  |  |  |  |  |  |  | 1,48019 | 5,18462 |
|  |  |  |  |  |  |  |  | 1,49184 | 5,22308 |
|  |  |  |  |  |  |  |  | 1,5035 | 5,26224 |
|  |  |  |  |  |  |  |  | 1,51515 | 5,30303 |
|  |  |  |  |  |  |  |  | 1,52681 | 5,34382 |
|  |  |  |  |  |  |  |  | 1,53846 | 5,38462 |
|  |  |  |  |  |  |  |  | 1,55012 | 5,42541 |
|  |  |  |  |  |  |  |  | 1,56177 | 5,4662 |
|  |  |  |  |  |  |  |  | 1,57343 | 5,50699 |
|  |  |  |  |  |  |  |  | 1,58508 | 5,54779 |
|  |  |  |  |  |  |  |  | 1,59674 | 5,58858 |
|  |  |  |  |  |  |  |  | 1,60839 | 5,62182 |
|  |  |  |  |  |  |  |  | 1,62005 | 5,65212 |
|  |  |  |  |  |  |  |  | 1,6317 | 5,68242 |
|  |  |  |  |  |  |  |  | 1,64336 | 5,71273 |
|  |  |  |  |  |  |  |  | 1,65501 | 5,74303 |
|  |  |  |  |  |  |  |  | 1,66667 | 5,77333 |
|  |  |  |  |  |  |  |  | 1,67832 | 5,80364 |
|  |  |  |  |  |  |  |  | 1,68998 | 5,83394 |
|  |  |  |  |  |  |  |  | 1,70163 | 5,86506 |
|  |  |  |  |  |  |  |  | 1,71329 | 5,90119 |
|  |  |  |  |  |  |  |  | 1,72494 | 5,93732 |
|  |  |  |  |  |  |  |  | 1,7366 | 5,97345 |
|  |  |  |  |  |  |  |  | 1,74825 | 6,00958 |
|  |  |  |  |  |  |  |  | 1,75991 | 6,04571 |
|  |  |  |  |  |  |  |  | 1,77156 | 6,08184 |
|  |  |  |  |  |  |  |  | 1,78322 | 6,11797 |
|  |  |  |  |  |  |  |  | 1,79487 | 6,1541 |
|  |  |  |  |  |  |  |  | 1,80653 | 6,19154 |
|  |  |  |  |  |  |  |  | 1,81818 | 6,23 |
|  |  |  |  |  |  |  |  | 1,82984 | 6,26846 |
|  |  |  |  |  |  |  |  | 1,84149 | 6,30692 |
|  |  |  |  |  |  |  |  | 1,85315 | 6,34538 |
|  |  |  |  |  |  |  |  | 1,8648 | 6,38385 |
|  |  |  |  |  |  |  |  | 1,87646 | 6,42231 |
|  |  |  |  |  |  |  |  | 1,88811 | 6,46077 |
|  |  |  |  |  |  |  |  | 1,89977 | 6,49923 |
|  |  |  |  |  |  |  |  | 1,91142 | 6,54226 |
|  |  |  |  |  |  |  |  | 1,92308 | 6,58538 |
|  |  |  |  |  |  |  |  | 1,93473 | 6,62851 |
|  |  |  |  |  |  |  |  | 1,94639 | 6,67163 |
|  |  |  |  |  |  |  |  | 1,95804 | 6,71476 |
|  |  |  |  |  |  |  |  | 1,9697 | 6,75788 |
|  |  |  |  |  |  |  |  | 1,98135 | 6,801 |
|  |  |  |  |  |  |  |  | 1,99301 | 6,84413 |
|  |  |  |  |  |  |  |  | 2,00466 | 6,88072 |
|  |  |  |  |  |  |  |  | 2,01632 | 6,90753 |
|  |  |  |  |  |  |  |  | 2,02797 | 6,93434 |
|  |  |  |  |  |  |  |  | 2,03963 | 6,96114 |
|  |  |  |  |  |  |  |  | 2,05128 | 6,98795 |
|  |  |  |  |  |  |  |  | 2,06294 | 7,01476 |
|  |  |  |  |  |  |  |  | 2,07459 | 7,04156 |
|  |  |  |  |  |  |  |  | 2,08625 | 7,06837 |
|  |  |  |  |  |  |  |  | 2,0979 | 7,09517 |
|  |  |  |  |  |  |  |  | 2,10956 | 7,12676 |
|  |  |  |  |  |  |  |  | 2,12121 | 7,15939 |
|  |  |  |  |  |  |  |  | 2,13287 | 7,19203 |
|  |  |  |  |  |  |  |  | 2,14452 | 7,22466 |
|  |  |  |  |  |  |  |  | 2,15618 | 7,2573 |
|  |  |  |  |  |  |  |  | 2,16783 | 7,28993 |
|  |  |  |  |  |  |  |  | 2,17949 | 7,32256 |
|  |  |  |  |  |  |  |  | 2,19114 | 7,3552 |
|  |  |  |  |  |  |  |  | 2,2028 | 7,38867 |
|  |  |  |  |  |  |  |  | 2,21445 | 7,4248 |
|  |  |  |  |  |  |  |  | 2,22611 | 7,46093 |
|  |  |  |  |  |  |  |  | 2,23776 | 7,49706 |
|  |  |  |  |  |  |  |  | 2,24942 | 7,53319 |
|  |  |  |  |  |  |  |  | 2,26107 | 7,56932 |
|  |  |  |  |  |  |  |  | 2,27273 | 7,60545 |
|  |  |  |  |  |  |  |  | 2,28438 | 7,64159 |
|  |  |  |  |  |  |  |  | 2,29604 | 7,67772 |
|  |  |  |  |  |  |  |  | 2,30769 | 7,71077 |
|  |  |  |  |  |  |  |  | 2,31935 | 7,74224 |
|  |  |  |  |  |  |  |  | 2,331 | 7,77371 |
|  |  |  |  |  |  |  |  | 2,34266 | 7,80517 |
|  |  |  |  |  |  |  |  | 2,35431 | 7,83664 |
|  |  |  |  |  |  |  |  | 2,36597 | 7,86811 |
|  |  |  |  |  |  |  |  | 2,37762 | 7,89958 |
|  |  |  |  |  |  |  |  | 2,38928 | 7,93105 |
|  |  |  |  |  |  |  |  | 2,40093 | 7,96196 |
|  |  |  |  |  |  |  |  | 2,41259 | 7,98643 |
|  |  |  |  |  |  |  |  | 2,42424 | 8,01091 |
|  |  |  |  |  |  |  |  | 2,4359 | 8,03538 |
|  |  |  |  |  |  |  |  | 2,44755 | 8,05986 |
|  |  |  |  |  |  |  |  | 2,45921 | 8,08434 |
|  |  |  |  |  |  |  |  | 2,47086 | 8,10881 |
|  |  |  |  |  |  |  |  | 2,48252 | 8,13329 |
|  |  |  |  |  |  |  |  | 2,49417 | 8,15776 |
|  |  |  |  |  |  |  |  | 2,50583 | 8,18807 |
|  |  |  |  |  |  |  |  | 2,51748 | 8,2242 |
|  |  |  |  |  |  |  |  | 2,52914 | 8,26033 |
|  |  |  |  |  |  |  |  | 2,54079 | 8,29646 |
|  |  |  |  |  |  |  |  | 2,55245 | 8,33259 |
|  |  |  |  |  |  |  |  | 2,5641 | 8,36872 |
|  |  |  |  |  |  |  |  | 2,57576 | 8,40485 |
|  |  |  |  |  |  |  |  | 2,58741 | 8,44098 |
|  |  |  |  |  |  |  |  | 2,59907 | 8,47711 |
|  |  |  |  |  |  |  |  | 2,61072 | 8,51002 |
|  |  |  |  |  |  |  |  | 2,62238 | 8,54266 |
|  |  |  |  |  |  |  |  | 2,63403 | 8,57529 |
|  |  |  |  |  |  |  |  | 2,64569 | 8,60793 |
|  |  |  |  |  |  |  |  | 2,65734 | 8,64056 |
|  |  |  |  |  |  |  |  | 2,669 | 8,67319 |
|  |  |  |  |  |  |  |  | 2,68065 | 8,70583 |
|  |  |  |  |  |  |  |  | 2,69231 | 8,73846 |
|  |  |  |  |  |  |  |  | 2,70396 | 8,76555 |
|  |  |  |  |  |  |  |  | 2,71562 | 8,78186 |
|  |  |  |  |  |  |  |  | 2,72727 | 8,79818 |
|  |  |  |  |  |  |  |  | 2,73893 | 8,8145 |
|  |  |  |  |  |  |  |  | 2,75058 | 8,83082 |
|  |  |  |  |  |  |  |  | 2,76224 | 8,84713 |
|  |  |  |  |  |  |  |  | 2,77389 | 8,86345 |
|  |  |  |  |  |  |  |  | 2,78555 | 8,87977 |
|  |  |  |  |  |  |  |  | 2,7972 | 8,89608 |
|  |  |  |  |  |  |  |  | 2,80886 | 8,9248 |
|  |  |  |  |  |  |  |  | 2,82051 | 8,95744 |
|  |  |  |  |  |  |  |  | 2,83217 | 8,99007 |
|  |  |  |  |  |  |  |  | 2,84382 | 9,0227 |
|  |  |  |  |  |  |  |  | 2,85548 | 9,05534 |
|  |  |  |  |  |  |  |  | 2,86713 | 9,08797 |
|  |  |  |  |  |  |  |  | 2,87879 | 9,12061 |
|  |  |  |  |  |  |  |  | 2,89044 | 9,15324 |
|  |  |  |  |  |  |  |  | 2,9021 | 9,18483 |
|  |  |  |  |  |  |  |  | 2,91375 | 9,21163 |
|  |  |  |  |  |  |  |  | 2,92541 | 9,23844 |
|  |  |  |  |  |  |  |  | 2,93706 | 9,26524 |
|  |  |  |  |  |  |  |  | 2,94872 | 9,29205 |
|  |  |  |  |  |  |  |  | 2,96037 | 9,31886 |
|  |  |  |  |  |  |  |  | 2,97203 | 9,34566 |
|  |  |  |  |  |  |  |  | 2,98368 | 9,37247 |
|  |  |  |  |  |  |  |  | 2,99534 | 9,39928 |
|  |  |  |  |  |  |  |  | 3,00699 | 9,42329 |
|  |  |  |  |  |  |  |  | 3,01865 | 9,44543 |
|  |  |  |  |  |  |  |  | 3,0303 | 9,46758 |
|  |  |  |  |  |  |  |  | 3,04196 | 9,48972 |
|  |  |  |  |  |  |  |  | 3,05361 | 9,51186 |
|  |  |  |  |  |  |  |  | 3,06527 | 9,53401 |
|  |  |  |  |  |  |  |  | 3,07692 | 9,55615 |
|  |  |  |  |  |  |  |  | 3,08858 | 9,5783 |
|  |  |  |  |  |  |  |  | 3,10023 | 9,60035 |
|  |  |  |  |  |  |  |  | 3,11189 | 9,61783 |
|  |  |  |  |  |  |  |  | 3,12354 | 9,63531 |
|  |  |  |  |  |  |  |  | 3,1352 | 9,6528 |
|  |  |  |  |  |  |  |  | 3,14685 | 9,67028 |
|  |  |  |  |  |  |  |  | 3,15851 | 9,68776 |
|  |  |  |  |  |  |  |  | 3,17016 | 9,70524 |
|  |  |  |  |  |  |  |  | 3,18182 | 9,72273 |
|  |  |  |  |  |  |  |  | 3,19347 | 9,74021 |
|  |  |  |  |  |  |  |  | 3,20513 | 9,76282 |
|  |  |  |  |  |  |  |  | 3,21678 | 9,79196 |
|  |  |  |  |  |  |  |  | 3,22844 | 9,8211 |
|  |  |  |  |  |  |  |  | 3,24009 | 9,85023 |
|  |  |  |  |  |  |  |  | 3,25175 | 9,87937 |
|  |  |  |  |  |  |  |  | 3,2634 | 9,90851 |
|  |  |  |  |  |  |  |  | 3,27506 | 9,93765 |
|  |  |  |  |  |  |  |  | 3,28671 | 9,96678 |
|  |  |  |  |  |  |  |  | 3,29837 | 9,99592 |
|  |  |  |  |  |  |  |  | 3,31002 | 10,01403 |
|  |  |  |  |  |  |  |  | 3,32168 | 10,03035 |
|  |  |  |  |  |  |  |  | 3,33333 | 10,04667 |
|  |  |  |  |  |  |  |  | 3,34499 | 10,06298 |
|  |  |  |  |  |  |  |  | 3,35664 | 10,0793 |
|  |  |  |  |  |  |  |  | 3,3683 | 10,09562 |
|  |  |  |  |  |  |  |  | 3,37995 | 10,11193 |
|  |  |  |  |  |  |  |  | 3,39161 | 10,12825 |
|  |  |  |  |  |  |  |  | 3,40326 | 10,1449 |
|  |  |  |  |  |  |  |  | 3,41492 | 10,16238 |
|  |  |  |  |  |  |  |  | 3,42657 | 10,17986 |
|  |  |  |  |  |  |  |  | 3,43823 | 10,19734 |
|  |  |  |  |  |  |  |  | 3,44988 | 10,21483 |
|  |  |  |  |  |  |  |  | 3,46154 | 10,23231 |
|  |  |  |  |  |  |  |  | 3,47319 | 10,24979 |
|  |  |  |  |  |  |  |  | 3,48485 | 10,26727 |
|  |  |  |  |  |  |  |  | 3,4965 | 10,28476 |
|  |  |  |  |  |  |  |  | 3,50816 | 10,30632 |
|  |  |  |  |  |  |  |  | 3,51981 | 10,32963 |
|  |  |  |  |  |  |  |  | 3,53147 | 10,35294 |
|  |  |  |  |  |  |  |  | 3,54312 | 10,37625 |
|  |  |  |  |  |  |  |  | 3,55478 | 10,39956 |
|  |  |  |  |  |  |  |  | 3,56643 | 10,42287 |
|  |  |  |  |  |  |  |  | 3,57809 | 10,44618 |
|  |  |  |  |  |  |  |  | 3,58974 | 10,46949 |
|  |  |  |  |  |  |  |  | 3,6014 | 10,4928 |
|  |  |  |  |  |  |  |  | 3,61305 | 10,51611 |
|  |  |  |  |  |  |  |  | 3,62471 | 10,53942 |
|  |  |  |  |  |  |  |  | 3,63636 | 10,56273 |
|  |  |  |  |  |  |  |  | 3,64802 | 10,58604 |
|  |  |  |  |  |  |  |  | 3,65967 | 10,60935 |
|  |  |  |  |  |  |  |  | 3,67133 | 10,63266 |
|  |  |  |  |  |  |  |  | 3,68298 | 10,65597 |
|  |  |  |  |  |  |  |  | 3,69464 | 10,67928 |
|  |  |  |  |  |  |  |  | 3,70629 | 10,7007 |
|  |  |  |  |  |  |  |  | 3,71795 | 10,72051 |
|  |  |  |  |  |  |  |  | 3,7296 | 10,74033 |
|  |  |  |  |  |  |  |  | 3,74126 | 10,76014 |
|  |  |  |  |  |  |  |  | 3,75291 | 10,77995 |
|  |  |  |  |  |  |  |  | 3,76457 | 10,79977 |
|  |  |  |  |  |  |  |  | 3,77622 | 10,81958 |
|  |  |  |  |  |  |  |  | 3,78788 | 10,83939 |
|  |  |  |  |  |  |  |  | 3,79953 | 10,85921 |
|  |  |  |  |  |  |  |  | 3,81119 | 10,87007 |
|  |  |  |  |  |  |  |  | 3,82284 | 10,88056 |
|  |  |  |  |  |  |  |  | 3,8345 | 10,89105 |
|  |  |  |  |  |  |  |  | 3,84615 | 10,90154 |
|  |  |  |  |  |  |  |  | 3,85781 | 10,91203 |
|  |  |  |  |  |  |  |  | 3,86946 | 10,92252 |
|  |  |  |  |  |  |  |  | 3,88112 | 10,93301 |
|  |  |  |  |  |  |  |  | 3,89277 | 10,9435 |
|  |  |  |  |  |  |  |  | 3,90443 | 10,95841 |
|  |  |  |  |  |  |  |  | 3,91608 | 10,98056 |
|  |  |  |  |  |  |  |  | 3,92774 | 11,0027 |
|  |  |  |  |  |  |  |  | 3,93939 | 11,02485 |
|  |  |  |  |  |  |  |  | 3,95105 | 11,04699 |
|  |  |  |  |  |  |  |  | 3,9627 | 11,06914 |
|  |  |  |  |  |  |  |  | 3,97436 | 11,09128 |
|  |  |  |  |  |  |  |  | 3,98601 | 11,11343 |
|  |  |  |  |  |  |  |  | 3,99767 | 11,13557 |
|  |  |  |  |  |  |  |  | 4,00932 | 11,14932 |
|  |  |  |  |  |  |  |  | 4,02098 | 11,16098 |
|  |  |  |  |  |  |  |  | 4,03263 | 11,17263 |
|  |  |  |  |  |  |  |  | 4,04429 | 11,18429 |
|  |  |  |  |  |  |  |  | 4,05594 | 11,19594 |
|  |  |  |  |  |  |  |  | 4,0676 | 11,2076 |
|  |  |  |  |  |  |  |  | 4,07925 | 11,21925 |
|  |  |  |  |  |  |  |  | 4,09091 | 11,23091 |
|  |  |  |  |  |  |  |  | 4,10256 | 11,2441 |
|  |  |  |  |  |  |  |  | 4,11422 | 11,26275 |
|  |  |  |  |  |  |  |  | 4,12587 | 11,2814 |
|  |  |  |  |  |  |  |  | 4,13753 | 11,30005 |
|  |  |  |  |  |  |  |  | 4,14918 | 11,31869 |
|  |  |  |  |  |  |  |  | 4,16084 | 11,33734 |
|  |  |  |  |  |  |  |  | 4,17249 | 11,35599 |
|  |  |  |  |  |  |  |  | 4,18415 | 11,37464 |
|  |  |  |  |  |  |  |  | 4,1958 | 11,39329 |
|  |  |  |  |  |  |  |  | 4,20746 | 11,41193 |
|  |  |  |  |  |  |  |  | 4,21911 | 11,43058 |
|  |  |  |  |  |  |  |  | 4,23077 | 11,44923 |
|  |  |  |  |  |  |  |  | 4,24242 | 11,46788 |
|  |  |  |  |  |  |  |  | 4,25408 | 11,48653 |
|  |  |  |  |  |  |  |  | 4,26573 | 11,50517 |
|  |  |  |  |  |  |  |  | 4,27739 | 11,52382 |
|  |  |  |  |  |  |  |  | 4,28904 | 11,54247 |
|  |  |  |  |  |  |  |  | 4,3007 | 11,56105 |
|  |  |  |  |  |  |  |  | 4,31235 | 11,57853 |
|  |  |  |  |  |  |  |  | 4,32401 | 11,59601 |
|  |  |  |  |  |  |  |  | 4,33566 | 11,6135 |
|  |  |  |  |  |  |  |  | 4,34732 | 11,63098 |
|  |  |  |  |  |  |  |  | 4,35897 | 11,64846 |
|  |  |  |  |  |  |  |  | 4,37063 | 11,66594 |
|  |  |  |  |  |  |  |  | 4,38228 | 11,68343 |
|  |  |  |  |  |  |  |  | 4,39394 | 11,70091 |
|  |  |  |  |  |  |  |  | 4,40559 | 11,7128 |
|  |  |  |  |  |  |  |  | 4,41725 | 11,71862 |
|  |  |  |  |  |  |  |  | 4,4289 | 11,72445 |
|  |  |  |  |  |  |  |  | 4,44056 | 11,73028 |
|  |  |  |  |  |  |  |  | 4,45221 | 11,73611 |
|  |  |  |  |  |  |  |  | 4,46387 | 11,74193 |
|  |  |  |  |  |  |  |  | 4,47552 | 11,74776 |
|  |  |  |  |  |  |  |  | 4,48718 | 11,75359 |
|  |  |  |  |  |  |  |  | 4,49883 | 11,75942 |
|  |  |  |  |  |  |  |  | 4,51049 | 11,77783 |
|  |  |  |  |  |  |  |  | 4,52214 | 11,79765 |
|  |  |  |  |  |  |  |  | 4,5338 | 11,81746 |
|  |  |  |  |  |  |  |  | 4,54545 | 11,83727 |
|  |  |  |  |  |  |  |  | 4,55711 | 11,85709 |
|  |  |  |  |  |  |  |  | 4,56876 | 11,8769 |
|  |  |  |  |  |  |  |  | 4,58042 | 11,89671 |
|  |  |  |  |  |  |  |  | 4,59207 | 11,91653 |
|  |  |  |  |  |  |  |  | 4,60373 | 11,93448 |
|  |  |  |  |  |  |  |  | 4,61538 | 11,94846 |
|  |  |  |  |  |  |  |  | 4,62704 | 11,96245 |
|  |  |  |  |  |  |  |  | 4,63869 | 11,97643 |
|  |  |  |  |  |  |  |  | 4,65035 | 11,99042 |
|  |  |  |  |  |  |  |  | 4,662 | 12,00441 |
|  |  |  |  |  |  |  |  | 4,67366 | 12,01839 |
|  |  |  |  |  |  |  |  | 4,68531 | 12,03238 |
|  |  |  |  |  |  |  |  | 4,69697 | 12,04636 |
|  |  |  |  |  |  |  |  | 4,70862 | 12,05604 |
|  |  |  |  |  |  |  |  | 4,72028 | 12,0642 |
|  |  |  |  |  |  |  |  | 4,73193 | 12,07235 |
|  |  |  |  |  |  |  |  | 4,74359 | 12,08051 |
|  |  |  |  |  |  |  |  | 4,75524 | 12,08867 |
|  |  |  |  |  |  |  |  | 4,7669 | 12,09683 |
|  |  |  |  |  |  |  |  | 4,77855 | 12,10499 |
|  |  |  |  |  |  |  |  | 4,79021 | 12,11315 |
|  |  |  |  |  |  |  |  | 4,80186 | 12,12298 |
|  |  |  |  |  |  |  |  | 4,81352 | 12,14163 |
|  |  |  |  |  |  |  |  | 4,82517 | 12,16028 |
|  |  |  |  |  |  |  |  | 4,83683 | 12,17893 |
|  |  |  |  |  |  |  |  | 4,84848 | 12,19758 |
|  |  |  |  |  |  |  |  | 4,86014 | 12,21622 |
|  |  |  |  |  |  |  |  | 4,87179 | 12,23487 |
|  |  |  |  |  |  |  |  | 4,88345 | 12,25352 |
|  |  |  |  |  |  |  |  | 4,8951 | 12,27217 |
|  |  |  |  |  |  |  |  | 4,90676 | 12,28135 |
|  |  |  |  |  |  |  |  | 4,91841 | 12,28368 |
|  |  |  |  |  |  |  |  | 4,93007 | 12,28601 |
|  |  |  |  |  |  |  |  | 4,94172 | 12,28834 |
|  |  |  |  |  |  |  |  | 4,95338 | 12,29068 |
|  |  |  |  |  |  |  |  | 4,96503 | 12,29301 |
|  |  |  |  |  |  |  |  | 4,97669 | 12,29534 |
|  |  |  |  |  |  |  |  | 4,98834 | 12,29767 |
|  |  |  |  |  |  |  |  | 5 | 12,3 |

**DATASET OF THERMAL PROPERTIES**

The following dat**as**et corresponds to Differential scanning calorimetry (DSC) and Thermogravimetric Analysis (TGA) of Epoxy resin and their biocomposites.

**Notes:**

-EP: Epoxy Resin, EP-FP: Biocomposite Epoxy Resin-Fique Powder, EP-NWF: Biocomposite Epoxy Resin-nonwoven industrial fique fiber mats, EP-UF: Biocomposite Epoxy Resin- unidirectional fique fiber mat.

**Differential scanning calorimetry (DSC)**

| EP cooling step | | EP Second Heating Step | | EP-FP cooling step | | EP-FP Second Heating Step | | EP UF cooling step | | EP UF Second Heating Step | |
| --- | --- | --- | --- | --- | --- | --- | --- | --- | --- | --- | --- |
| Temperature | Heat Flow (W/g) | Temperature | Heat Flow (W/g) | Temperature | Heat Flow (W/g) | Temperature | Heat Flow (W/g) | Temperature | Heat Flow (W/g) | Temperature | Heat Flow (W/g) |
| 149,93 | -0,08898 | -19,03 | 0,1298 | 149,97 | -0,00793 | -19,01 | 0,1725 | 149,97 | -0,02278 | -19,09 | 0,0927 |
| 149,93 | -0,08899 | -19,03 | 0,1259 | 149,97 | -0,00794 | -19,01 | 0,1749 | 149,97 | -0,02277 | -19,09 | 0,08719 |
| 149,93 | -0,08899 | -19,03 | 0,1225 | 149,97 | -0,00795 | -19,01 | 0,1772 | 149,97 | -0,02275 | -19,09 | 0,08214 |
| 149,93 | -0,089 | -19,03 | 0,1195 | 149,96 | -0,00795 | -19 | 0,1794 | 149,96 | -0,02258 | -19,08 | 0,07774 |
| 149,93 | -0,08901 | -19,02 | 0,1168 | 149,96 | -0,00796 | -18,99 | 0,1814 | 149,96 | -0,02175 | -19,08 | 0,07352 |
| 149,92 | -0,08901 | -19,02 | 0,1144 | 149,95 | -0,00793 | -18,99 | 0,1829 | 149,95 | -0,0192 | -19,08 | 0,06949 |
| 149,92 | -0,08897 | -19,01 | 0,112 | 149,94 | -0,00783 | -18,98 | 0,1846 | 149,95 | -0,0136 | -19,07 | 0,06584 |
| 149,92 | -0,08874 | -19,01 | 0,1098 | 149,94 | -0,00757 | -18,97 | 0,1862 | 149,94 | -0,00403 | -19,07 | 0,06283 |
| 149,91 | -0,08783 | -19 | 0,1079 | 149,93 | -0,00711 | -18,96 | 0,1874 | 149,93 | 0,00823 | -19,06 | 0,05995 |
| 149,91 | -0,08573 | -18,99 | 0,1061 | 149,92 | -0,00639 | -18,95 | 0,1882 | 149,92 | 0,02 | -19,05 | 0,05722 |
| 149,9 | -0,08162 | -18,99 | 0,1039 | 149,91 | -0,00545 | -18,94 | 0,1887 | 149,92 | 0,03019 | -19,04 | 0,05429 |
| 149,89 | -0,0752 | -18,98 | 0,1017 | 149,9 | -0,00437 | -18,93 | 0,1889 | 149,91 | 0,03878 | -19,04 | 0,0508 |
| 149,89 | -0,0677 | -18,97 | 0,09951 | 149,88 | -0,00328 | -18,92 | 0,1889 | 149,89 | 0,04619 | -19,03 | 0,04751 |
| 149,88 | -0,06057 | -18,96 | 0,09741 | 149,87 | -0,00223 | -18,91 | 0,1889 | 149,88 | 0,05257 | -19,02 | 0,04463 |
| 149,87 | -0,05438 | -18,94 | 0,09542 | 149,86 | -0,00122 | -18,9 | 0,1887 | 149,87 | 0,05837 | -19,01 | 0,04137 |
| 149,86 | -0,04877 | -18,93 | 0,09296 | 149,84 | -3,12E-04 | -18,89 | 0,1883 | 149,86 | 0,06322 | -19 | 0,03809 |
| 149,85 | -0,04391 | -18,92 | 0,09023 | 149,83 | 4,75E-04 | -18,88 | 0,1878 | 149,84 | 0,06732 | -18,98 | 0,03508 |
| 149,84 | -0,03999 | -18,91 | 0,08771 | 149,81 | 0,00106 | -18,86 | 0,1873 | 149,83 | 0,07159 | -18,97 | 0,03176 |
| 149,83 | -0,0366 | -18,9 | 0,08496 | 149,79 | 0,00144 | -18,85 | 0,1868 | 149,81 | 0,07527 | -18,96 | 0,02816 |
| 149,81 | -0,03335 | -18,89 | 0,08259 | 149,78 | 0,00172 | -18,84 | 0,1863 | 149,8 | 0,0786 | -18,94 | 0,02486 |
| 149,8 | -0,03067 | -18,87 | 0,0801 | 149,76 | 0,00194 | -18,82 | 0,1848 | 149,78 | 0,08144 | -18,93 | 0,02165 |
| 149,78 | -0,02834 | -18,86 | 0,07757 | 149,74 | 0,00214 | -18,81 | 0,1837 | 149,76 | 0,08377 | -18,91 | 0,0186 |
| 149,77 | -0,02609 | -18,84 | 0,07525 | 149,72 | 0,00236 | -18,79 | 0,1829 | 149,75 | 0,0861 | -18,9 | 0,01544 |
| 149,75 | -0,02403 | -18,83 | 0,07274 | 149,7 | 0,00267 | -18,77 | 0,1825 | 149,73 | 0,08884 | -18,88 | 0,0127 |
| 149,74 | -0,02197 | -18,81 | 0,07039 | 149,68 | 0,00312 | -18,76 | 0,1823 | 149,71 | 0,0919 | -18,87 | 0,00987 |
| 149,72 | -0,02027 | -18,79 | 0,06819 | 149,66 | 0,00374 | -18,74 | 0,1823 | 149,69 | 0,09493 | -18,85 | 0,00707 |
| 149,7 | -0,01859 | -18,78 | 0,06624 | 149,64 | 0,00457 | -18,72 | 0,1822 | 149,67 | 0,09882 | -18,83 | 0,00443 |
| 149,68 | -0,01695 | -18,76 | 0,06392 | 149,62 | 0,00568 | -18,71 | 0,182 | 149,65 | 0,1024 | -18,82 | 0,00201 |
| 149,67 | -0,01512 | -18,74 | 0,0616 | 149,6 | 0,00704 | -18,69 | 0,1815 | 149,64 | 0,1059 | -18,8 | -3,18E-04 |
| 149,65 | -0,01304 | -18,72 | 0,05942 | 149,58 | 0,00877 | -18,67 | 0,1806 | 149,62 | 0,1089 | -18,78 | -0,00219 |
| 149,63 | -0,01104 | -18,7 | 0,05725 | 149,56 | 0,01108 | -18,65 | 0,1791 | 149,59 | 0,1118 | -18,76 | -0,00422 |
| 149,61 | -0,0086 | -18,69 | 0,05549 | 149,54 | 0,01373 | -18,63 | 0,1769 | 149,57 | 0,1155 | -18,74 | -0,00634 |
| 149,59 | -0,00619 | -18,66 | 0,05347 | 149,52 | 0,01688 | -18,61 | 0,1741 | 149,55 | 0,1198 | -18,72 | -0,00798 |
| 149,57 | -0,00381 | -18,65 | 0,05147 | 149,5 | 0,02016 | -18,59 | 0,1703 | 149,53 | 0,1242 | -18,7 | -0,00961 |
| 149,55 | -0,00112 | -18,63 | 0,04965 | 149,47 | 0,02353 | -18,57 | 0,1662 | 149,51 | 0,1282 | -18,68 | -0,01153 |
| 149,53 | 0,00145 | -18,61 | 0,04771 | 149,45 | 0,02687 | -18,55 | 0,162 | 149,49 | 0,132 | -18,66 | -0,01369 |
| 149,51 | 0,00379 | -18,59 | 0,04559 | 149,43 | 0,03029 | -18,53 | 0,1577 | 149,46 | 0,1355 | -18,64 | -0,01591 |
| 149,48 | 0,00628 | -18,57 | 0,04354 | 149,4 | 0,03348 | -18,51 | 0,1533 | 149,44 | 0,1393 | -18,62 | -0,01805 |
| 149,46 | 0,00916 | -18,54 | 0,04139 | 149,38 | 0,03629 | -18,49 | 0,1494 | 149,42 | 0,1432 | -18,6 | -0,0192 |
| 149,44 | 0,01189 | -18,52 | 0,03954 | 149,36 | 0,03907 | -18,47 | 0,1459 | 149,4 | 0,147 | -18,58 | -0,021 |
| 149,42 | 0,01499 | -18,5 | 0,03768 | 149,33 | 0,04186 | -18,45 | 0,1428 | 149,37 | 0,1508 | -18,55 | -0,02284 |
| 149,4 | 0,01799 | -18,48 | 0,0356 | 149,31 | 0,04484 | -18,43 | 0,1395 | 149,35 | 0,1543 | -18,53 | -0,02451 |
| 149,37 | 0,02031 | -18,46 | 0,03328 | 149,28 | 0,04795 | -18,41 | 0,136 | 149,32 | 0,1581 | -18,51 | -0,02669 |
| 149,35 | 0,02266 | -18,43 | 0,03113 | 149,26 | 0,05043 | -18,38 | 0,133 | 149,3 | 0,1616 | -18,49 | -0,02875 |
| 149,33 | 0,02526 | -18,41 | 0,02934 | 149,24 | 0,05306 | -18,36 | 0,1303 | 149,27 | 0,165 | -18,46 | -0,03029 |
| 149,3 | 0,02758 | -18,39 | 0,02739 | 149,21 | 0,05586 | -18,34 | 0,1276 | 149,25 | 0,1685 | -18,44 | -0,03181 |
| 149,28 | 0,03036 | -18,36 | 0,02505 | 149,18 | 0,05904 | -18,32 | 0,1245 | 149,23 | 0,1713 | -18,42 | -0,03316 |
| 149,25 | 0,03323 | -18,34 | 0,02276 | 149,16 | 0,06246 | -18,29 | 0,1209 | 149,2 | 0,1738 | -18,39 | -0,03437 |
| 149,23 | 0,03577 | -18,32 | 0,02052 | 149,13 | 0,06576 | -18,27 | 0,1178 | 149,17 | 0,1773 | -18,37 | -0,03614 |
| 149,2 | 0,03867 | -18,3 | 0,01841 | 149,11 | 0,06851 | -18,25 | 0,1149 | 149,15 | 0,18 | -18,34 | -0,03758 |
| 149,18 | 0,04095 | -18,27 | 0,01677 | 149,08 | 0,07088 | -18,23 | 0,1125 | 149,12 | 0,1828 | -18,32 | -0,03925 |
| 149,15 | 0,04306 | -18,25 | 0,01458 | 149,06 | 0,07358 | -18,2 | 0,1102 | 149,1 | 0,1856 | -18,3 | -0,04088 |
| 149,13 | 0,04556 | -18,22 | 0,01256 | 149,03 | 0,07662 | -18,18 | 0,1077 | 149,07 | 0,1881 | -18,27 | -0,04246 |
| 149,1 | 0,0477 | -18,2 | 0,01044 | 149 | 0,07989 | -18,15 | 0,1051 | 149,04 | 0,1917 | -18,24 | -0,04382 |
| 149,08 | 0,05021 | -18,17 | 0,00891 | 148,98 | 0,08308 | -18,13 | 0,1022 | 149,02 | 0,1953 | -18,22 | -0,04486 |
| 149,05 | 0,05298 | -18,15 | 0,00703 | 148,95 | 0,08663 | -18,1 | 0,09911 | 148,99 | 0,1988 | -18,19 | -0,04625 |
| 149,02 | 0,05516 | -18,12 | 0,00507 | 148,92 | 0,08964 | -18,08 | 0,09614 | 148,96 | 0,2022 | -18,17 | -0,04759 |
| 149 | 0,0575 | -18,1 | 0,00294 | 148,9 | 0,09268 | -18,06 | 0,09352 | 148,94 | 0,2051 | -18,14 | -0,04902 |
| 148,97 | 0,06032 | -18,07 | 0,0013 | 148,87 | 0,09613 | -18,03 | 0,09115 | 148,91 | 0,2078 | -18,12 | -0,05033 |
| 148,94 | 0,06293 | -18,05 | -2,77E-04 | 148,84 | 0,09896 | -18,01 | 0,08874 | 148,88 | 0,211 | -18,09 | -0,05131 |
| 148,92 | 0,06567 | -18,02 | -0,00204 | 148,82 | 0,1017 | -17,98 | 0,08628 | 148,86 | 0,214 | -18,06 | -0,0523 |
| 148,89 | 0,06847 | -18 | -0,00403 | 148,79 | 0,105 | -17,95 | 0,08341 | 148,83 | 0,2167 | -18,04 | -0,05344 |
| 148,86 | 0,0713 | -17,97 | -0,0063 | 148,76 | 0,1079 | -17,93 | 0,08073 | 148,8 | 0,2189 | -18,01 | -0,05479 |
| 148,84 | 0,07386 | -17,94 | -0,00839 | 148,73 | 0,1109 | -17,9 | 0,07815 | 148,77 | 0,2215 | -17,98 | -0,05597 |
| 148,81 | 0,07622 | -17,92 | -0,00988 | 148,71 | 0,1139 | -17,88 | 0,07572 | 148,74 | 0,2247 | -17,95 | -0,05721 |
| 148,78 | 0,07874 | -17,89 | -0,01172 | 148,68 | 0,117 | -17,85 | 0,07323 | 148,71 | 0,2285 | -17,93 | -0,05825 |
| 148,75 | 0,08108 | -17,87 | -0,0134 | 148,65 | 0,1204 | -17,83 | 0,071 | 148,69 | 0,2323 | -17,9 | -0,05911 |
| 148,73 | 0,08371 | -17,84 | -0,01553 | 148,62 | 0,1235 | -17,8 | 0,06829 | 148,66 | 0,2353 | -17,87 | -0,06009 |
| 148,7 | 0,08683 | -17,81 | -0,01755 | 148,59 | 0,1265 | -17,77 | 0,06598 | 148,63 | 0,2377 | -17,85 | -0,06119 |
| 148,67 | 0,08949 | -17,78 | -0,01865 | 148,57 | 0,1294 | -17,75 | 0,06317 | 148,6 | 0,2395 | -17,82 | -0,06219 |
| 148,64 | 0,09222 | -17,76 | -0,01974 | 148,54 | 0,1324 | -17,72 | 0,06061 | 148,57 | 0,2416 | -17,79 | -0,06351 |
| 148,62 | 0,09471 | -17,73 | -0,02125 | 148,51 | 0,1355 | -17,69 | 0,05837 | 148,54 | 0,2443 | -17,76 | -0,06474 |
| 148,59 | 0,09686 | -17,7 | -0,02297 | 148,48 | 0,1382 | -17,67 | 0,05619 | 148,51 | 0,2471 | -17,74 | -0,06569 |
| 148,56 | 0,09915 | -17,68 | -0,02538 | 148,45 | 0,1409 | -17,64 | 0,05406 | 148,49 | 0,2491 | -17,71 | -0,06641 |
| 148,53 | 0,1017 | -17,65 | -0,02724 | 148,42 | 0,1436 | -17,62 | 0,0516 | 148,46 | 0,252 | -17,68 | -0,06731 |
| 148,5 | 0,1039 | -17,62 | -0,02882 | 148,4 | 0,1463 | -17,59 | 0,04924 | 148,43 | 0,2543 | -17,65 | -0,06801 |
| 148,47 | 0,1063 | -17,59 | -0,03016 | 148,37 | 0,1493 | -17,56 | 0,04682 | 148,4 | 0,257 | -17,62 | -0,06857 |
| 148,44 | 0,109 | -17,57 | -0,03165 | 148,34 | 0,1522 | -17,53 | 0,04478 | 148,37 | 0,2598 | -17,59 | -0,06931 |
| 148,42 | 0,1116 | -17,54 | -0,03354 | 148,31 | 0,1551 | -17,5 | 0,04284 | 148,34 | 0,2619 | -17,57 | -0,06988 |
| 148,39 | 0,1141 | -17,51 | -0,03527 | 148,28 | 0,1578 | -17,48 | 0,04055 | 148,31 | 0,2638 | -17,54 | -0,0705 |
| 148,36 | 0,1166 | -17,48 | -0,03685 | 148,25 | 0,1606 | -17,45 | 0,03831 | 148,28 | 0,2661 | -17,51 | -0,07147 |
| 148,33 | 0,119 | -17,45 | -0,038 | 148,22 | 0,1633 | -17,42 | 0,03635 | 148,25 | 0,2683 | -17,48 | -0,07243 |
| 148,3 | 0,1212 | -17,42 | -0,03915 | 148,19 | 0,1663 | -17,4 | 0,03364 | 148,22 | 0,2711 | -17,45 | -0,07329 |
| 148,27 | 0,1239 | -17,4 | -0,04051 | 148,16 | 0,1693 | -17,37 | 0,0317 | 148,19 | 0,2732 | -17,42 | -0,07403 |
| 148,24 | 0,1262 | -17,37 | -0,04221 | 148,13 | 0,1721 | -17,34 | 0,02979 | 148,16 | 0,2751 | -17,39 | -0,07456 |
| 148,21 | 0,1283 | -17,34 | -0,04394 | 148,11 | 0,1748 | -17,31 | 0,02816 | 148,13 | 0,2772 | -17,36 | -0,07504 |
| 148,18 | 0,1306 | -17,31 | -0,04566 | 148,08 | 0,1773 | -17,28 | 0,02632 | 148,1 | 0,2793 | -17,33 | -0,07576 |
| 148,15 | 0,1329 | -17,28 | -0,04721 | 148,05 | 0,1793 | -17,25 | 0,02405 | 148,07 | 0,2817 | -17,3 | -0,0767 |
| 148,12 | 0,1355 | -17,25 | -0,04855 | 148,02 | 0,1815 | -17,23 | 0,02165 | 148,04 | 0,2841 | -17,27 | -0,07763 |
| 148,09 | 0,1377 | -17,22 | -0,04982 | 147,99 | 0,1839 | -17,2 | 0,01935 | 148,01 | 0,2857 | -17,24 | -0,0785 |
| 148,07 | 0,1396 | -17,19 | -0,05108 | 147,96 | 0,1863 | -17,17 | 0,01759 | 147,98 | 0,2871 | -17,21 | -0,07929 |
| 148,04 | 0,1417 | -17,17 | -0,05225 | 147,93 | 0,1893 | -17,14 | 0,01639 | 147,95 | 0,2892 | -17,18 | -0,07994 |
| 148,01 | 0,1439 | -17,14 | -0,05381 | 147,9 | 0,192 | -17,11 | 0,01461 | 147,92 | 0,2911 | -17,15 | -0,08052 |
| 147,98 | 0,1467 | -17,11 | -0,05523 | 147,87 | 0,1948 | -17,08 | 0,01314 | 147,89 | 0,2932 | -17,12 | -0,08107 |
| 147,95 | 0,1492 | -17,08 | -0,05648 | 147,84 | 0,1975 | -17,05 | 0,01127 | 147,86 | 0,2949 | -17,09 | -0,08157 |
| 147,92 | 0,1513 | -17,05 | -0,05723 | 147,81 | 0,1995 | -17,03 | 0,00921 | 147,83 | 0,2965 | -17,06 | -0,08198 |
| 147,89 | 0,1535 | -17,02 | -0,05796 | 147,78 | 0,2016 | -17 | 0,00737 | 147,8 | 0,2982 | -17,03 | -0,08232 |
| 147,86 | 0,1557 | -16,99 | -0,05882 | 147,75 | 0,2039 | -16,97 | 0,00547 | 147,77 | 0,3 | -17 | -0,08269 |
| 147,83 | 0,158 | -16,96 | -0,06034 | 147,72 | 0,2059 | -16,94 | 0,00359 | 147,74 | 0,3019 | -16,97 | -0,08313 |
| 147,8 | 0,1601 | -16,93 | -0,0619 | 147,69 | 0,2083 | -16,91 | 0,00231 | 147,71 | 0,3038 | -16,94 | -0,08354 |
| 147,77 | 0,1621 | -16,9 | -0,0635 | 147,66 | 0,2108 | -16,88 | 0,00101 | 147,67 | 0,306 | -16,91 | -0,08391 |
| 147,74 | 0,1641 | -16,87 | -0,06435 | 147,63 | 0,2133 | -16,85 | -6,10E-04 | 147,64 | 0,3078 | -16,88 | -0,08425 |
| 147,71 | 0,1662 | -16,84 | -0,06534 | 147,6 | 0,2155 | -16,82 | -0,00257 | 147,61 | 0,3098 | -16,85 | -0,08454 |
| 147,68 | 0,1681 | -16,81 | -0,06631 | 147,57 | 0,2172 | -16,79 | -0,00466 | 147,58 | 0,311 | -16,82 | -0,08494 |
| 147,65 | 0,17 | -16,78 | -0,06772 | 147,54 | 0,2195 | -16,76 | -0,00651 | 147,55 | 0,3122 | -16,79 | -0,0853 |
| 147,61 | 0,1718 | -16,75 | -0,06912 | 147,51 | 0,2213 | -16,73 | -0,00758 | 147,52 | 0,3136 | -16,76 | -0,08568 |
| 147,58 | 0,1735 | -16,72 | -0,07024 | 147,48 | 0,2233 | -16,7 | -0,00866 | 147,49 | 0,3145 | -16,73 | -0,08613 |
| 147,55 | 0,1754 | -16,69 | -0,07118 | 147,45 | 0,2254 | -16,67 | -0,00966 | 147,46 | 0,3157 | -16,7 | -0,08653 |
| 147,52 | 0,1773 | -16,66 | -0,07222 | 147,42 | 0,2277 | -16,65 | -0,01104 | 147,43 | 0,3175 | -16,67 | -0,08691 |
| 147,49 | 0,1794 | -16,63 | -0,07326 | 147,39 | 0,2299 | -16,62 | -0,01299 | 147,4 | 0,3191 | -16,63 | -0,08739 |
| 147,46 | 0,1816 | -16,6 | -0,07418 | 147,35 | 0,2318 | -16,59 | -0,01477 | 147,37 | 0,3211 | -16,6 | -0,08772 |
| 147,43 | 0,1833 | -16,57 | -0,07493 | 147,32 | 0,2335 | -16,56 | -0,01614 | 147,33 | 0,3225 | -16,57 | -0,08806 |
| 147,4 | 0,1852 | -16,54 | -0,07549 | 147,29 | 0,2351 | -16,53 | -0,01695 | 147,3 | 0,3234 | -16,54 | -0,08836 |
| 147,37 | 0,1875 | -16,51 | -0,0762 | 147,26 | 0,2369 | -16,5 | -0,01805 | 147,27 | 0,3245 | -16,51 | -0,08867 |
| 147,34 | 0,1895 | -16,48 | -0,07746 | 147,23 | 0,2391 | -16,46 | -0,01917 | 147,24 | 0,326 | -16,48 | -0,08901 |
| 147,31 | 0,1912 | -16,45 | -0,07886 | 147,2 | 0,2413 | -16,44 | -0,02064 | 147,21 | 0,3274 | -16,45 | -0,08936 |
| 147,28 | 0,193 | -16,42 | -0,07991 | 147,17 | 0,2437 | -16,41 | -0,02216 | 147,18 | 0,3288 | -16,42 | -0,08966 |
| 147,25 | 0,1943 | -16,39 | -0,08065 | 147,14 | 0,2456 | -16,38 | -0,02339 | 147,14 | 0,33 | -16,39 | -0,0899 |
| 147,22 | 0,1955 | -16,36 | -0,08108 | 147,11 | 0,2472 | -16,35 | -0,02436 | 147,11 | 0,3309 | -16,36 | -0,09007 |
| 147,19 | 0,1975 | -16,33 | -0,08187 | 147,08 | 0,249 | -16,31 | -0,02568 | 147,08 | 0,3318 | -16,32 | -0,09027 |
| 147,15 | 0,1997 | -16,3 | -0,08335 | 147,05 | 0,2503 | -16,28 | -0,02687 | 147,05 | 0,3331 | -16,29 | -0,0905 |
| 147,12 | 0,2015 | -16,27 | -0,08477 | 147,02 | 0,2518 | -16,25 | -0,02787 | 147,02 | 0,3344 | -16,26 | -0,09077 |
| 147,09 | 0,2035 | -16,24 | -0,08586 | 146,98 | 0,2538 | -16,22 | -0,02902 | 146,99 | 0,3358 | -16,23 | -0,09105 |
| 147,06 | 0,205 | -16,2 | -0,08648 | 146,95 | 0,2553 | -16,19 | -0,03047 | 146,96 | 0,3375 | -16,2 | -0,09134 |
| 147,03 | 0,2062 | -16,17 | -0,08683 | 146,92 | 0,2572 | -16,16 | -0,03184 | 146,92 | 0,3386 | -16,17 | -0,09163 |
| 147 | 0,208 | -16,14 | -0,08735 | 146,89 | 0,2589 | -16,13 | -0,03329 | 146,89 | 0,3395 | -16,14 | -0,092 |
| 146,97 | 0,2097 | -16,11 | -0,08809 | 146,86 | 0,2603 | -16,1 | -0,03465 | 146,86 | 0,3407 | -16,1 | -0,09238 |
| 146,94 | 0,2113 | -16,08 | -0,08891 | 146,83 | 0,2618 | -16,07 | -0,03552 | 146,83 | 0,3417 | -16,07 | -0,09273 |
| 146,91 | 0,213 | -16,05 | -0,08963 | 146,8 | 0,2635 | -16,04 | -0,03637 | 146,8 | 0,3427 | -16,04 | -0,09299 |
| 146,87 | 0,2144 | -16,02 | -0,09033 | 146,77 | 0,2654 | -16,01 | -0,03693 | 146,77 | 0,3434 | -16,01 | -0,09316 |
| 146,84 | 0,2159 | -15,99 | -0,0911 | 146,73 | 0,2671 | -15,98 | -0,03743 | 146,73 | 0,344 | -15,98 | -0,09329 |
| 146,81 | 0,2171 | -15,96 | -0,09186 | 146,7 | 0,2688 | -15,95 | -0,03825 | 146,7 | 0,3447 | -15,95 | -0,09344 |
| 146,78 | 0,2182 | -15,92 | -0,09274 | 146,67 | 0,2697 | -15,92 | -0,03953 | 146,67 | 0,3456 | -15,92 | -0,0936 |
| 146,75 | 0,2202 | -15,89 | -0,09357 | 146,64 | 0,2706 | -15,89 | -0,04079 | 146,64 | 0,3464 | -15,88 | -0,09377 |
| 146,72 | 0,2223 | -15,86 | -0,09445 | 146,61 | 0,272 | -15,85 | -0,04192 | 146,6 | 0,3471 | -15,85 | -0,09391 |
| 146,69 | 0,224 | -15,83 | -0,09519 | 146,58 | 0,2742 | -15,82 | -0,04296 | 146,57 | 0,3475 | -15,82 | -0,09407 |
| 146,66 | 0,2251 | -15,8 | -0,09582 | 146,55 | 0,2761 | -15,79 | -0,04368 | 146,54 | 0,3484 | -15,79 | -0,09421 |
| 146,62 | 0,2259 | -15,77 | -0,09646 | 146,52 | 0,2777 | -15,76 | -0,04441 | 146,51 | 0,3499 | -15,76 | -0,09434 |
| 146,59 | 0,227 | -15,74 | -0,09697 | 146,48 | 0,2785 | -15,73 | -0,0452 | 146,48 | 0,3514 | -15,72 | -0,09448 |
| 146,56 | 0,2286 | -15,71 | -0,09758 | 146,45 | 0,2793 | -15,7 | -0,046 | 146,45 | 0,3527 | -15,69 | -0,0946 |
| 146,53 | 0,2305 | -15,68 | -0,0981 | 146,42 | 0,2803 | -15,67 | -0,04683 | 146,41 | 0,3534 | -15,66 | -0,09477 |
| 146,5 | 0,2319 | -15,64 | -0,09853 | 146,39 | 0,2821 | -15,64 | -0,0476 | 146,38 | 0,3539 | -15,63 | -0,09496 |
| 146,47 | 0,233 | -15,61 | -0,09908 | 146,36 | 0,2837 | -15,61 | -0,04855 | 146,35 | 0,3543 | -15,6 | -0,09514 |
| 146,44 | 0,2343 | -15,58 | -0,09976 | 146,33 | 0,2849 | -15,58 | -0,04987 | 146,32 | 0,355 | -15,57 | -0,09531 |
| 146,4 | 0,2361 | -15,55 | -0,1005 | 146,29 | 0,2862 | -15,54 | -0,05098 | 146,28 | 0,3558 | -15,53 | -0,09546 |
| 146,37 | 0,238 | -15,52 | -0,1012 | 146,26 | 0,2874 | -15,51 | -0,05181 | 146,25 | 0,3569 | -15,5 | -0,09556 |
| 146,34 | 0,239 | -15,49 | -0,1019 | 146,23 | 0,2884 | -15,48 | -0,05245 | 146,22 | 0,3579 | -15,47 | -0,09567 |
| 146,31 | 0,2398 | -15,45 | -0,1025 | 146,2 | 0,2896 | -15,45 | -0,05295 | 146,19 | 0,3588 | -15,44 | -0,09578 |
| 146,28 | 0,2405 | -15,42 | -0,103 | 146,17 | 0,2909 | -15,42 | -0,05363 | 146,16 | 0,3592 | -15,41 | -0,09592 |
| 146,25 | 0,2416 | -15,39 | -0,1035 | 146,14 | 0,292 | -15,39 | -0,05468 | 146,12 | 0,3596 | -15,37 | -0,09605 |
| 146,21 | 0,243 | -15,36 | -0,104 | 146,1 | 0,2935 | -15,36 | -0,0557 | 146,09 | 0,3599 | -15,34 | -0,09616 |
| 146,18 | 0,2445 | -15,33 | -0,1045 | 146,07 | 0,2946 | -15,32 | -0,05636 | 146,06 | 0,3606 | -15,31 | -0,09626 |
| 146,15 | 0,2457 | -15,3 | -0,105 | 146,04 | 0,2957 | -15,29 | -0,05689 | 146,03 | 0,3613 | -15,28 | -0,09634 |
| 146,12 | 0,2468 | -15,27 | -0,1055 | 146,01 | 0,2966 | -15,26 | -0,05721 | 146 | 0,362 | -15,24 | -0,09643 |
| 146,09 | 0,2478 | -15,23 | -0,1059 | 145,98 | 0,2977 | -15,23 | -0,05744 | 145,96 | 0,3626 | -15,21 | -0,09653 |
| 146,06 | 0,249 | -15,2 | -0,1065 | 145,94 | 0,2991 | -15,2 | -0,05803 | 145,93 | 0,3632 | -15,18 | -0,09663 |
| 146,02 | 0,2501 | -15,17 | -0,1071 | 145,91 | 0,3 | -15,17 | -0,05876 | 145,9 | 0,3637 | -15,15 | -0,09673 |
| 145,99 | 0,2511 | -15,14 | -0,1077 | 145,88 | 0,3012 | -15,13 | -0,05942 | 145,87 | 0,3643 | -15,12 | -0,0968 |
| 145,96 | 0,2518 | -15,11 | -0,1081 | 145,85 | 0,3021 | -15,1 | -0,06012 | 145,83 | 0,3649 | -15,09 | -0,09688 |
| 145,93 | 0,2527 | -15,08 | -0,1085 | 145,82 | 0,303 | -15,07 | -0,06087 | 145,8 | 0,3655 | -15,05 | -0,09696 |
| 145,9 | 0,2542 | -15,04 | -0,1089 | 145,79 | 0,3039 | -15,04 | -0,06158 | 145,77 | 0,3661 | -15,02 | -0,09706 |
| 145,86 | 0,2557 | -15,01 | -0,1093 | 145,75 | 0,3046 | -15,01 | -0,06224 | 145,73 | 0,3666 | -14,99 | -0,09718 |
| 145,83 | 0,2571 | -14,98 | -0,1099 | 145,72 | 0,3055 | -14,98 | -0,06284 | 145,7 | 0,3672 | -14,95 | -0,09726 |
| 145,8 | 0,2581 | -14,95 | -0,1104 | 145,69 | 0,3066 | -14,95 | -0,06335 | 145,67 | 0,3678 | -14,92 | -0,09731 |
| 145,77 | 0,2588 | -14,91 | -0,1108 | 145,66 | 0,3078 | -14,91 | -0,0638 | 145,64 | 0,3683 | -14,89 | -0,09736 |
| 145,74 | 0,2598 | -14,88 | -0,1113 | 145,63 | 0,3088 | -14,88 | -0,06419 | 145,61 | 0,3688 | -14,86 | -0,09741 |
| 145,7 | 0,2607 | -14,85 | -0,1117 | 145,59 | 0,3095 | -14,85 | -0,06465 | 145,57 | 0,3694 | -14,83 | -0,09747 |
| 145,67 | 0,2619 | -14,82 | -0,112 | 145,56 | 0,3102 | -14,82 | -0,06537 | 145,54 | 0,3699 | -14,79 | -0,09751 |
| 145,64 | 0,2629 | -14,79 | -0,1123 | 145,53 | 0,3112 | -14,79 | -0,06634 | 145,51 | 0,3706 | -14,76 | -0,09757 |
| 145,61 | 0,2641 | -14,75 | -0,1126 | 145,5 | 0,3125 | -14,75 | -0,0672 | 145,48 | 0,371 | -14,73 | -0,09762 |
| 145,58 | 0,2655 | -14,72 | -0,1129 | 145,47 | 0,3138 | -14,72 | -0,06778 | 145,44 | 0,3713 | -14,7 | -0,0977 |
| 145,54 | 0,2669 | -14,69 | -0,1132 | 145,43 | 0,3149 | -14,69 | -0,06802 | 145,41 | 0,3716 | -14,66 | -0,09776 |
| 145,51 | 0,2679 | -14,66 | -0,1135 | 145,4 | 0,3154 | -14,66 | -0,06812 | 145,38 | 0,3718 | -14,63 | -0,09781 |
| 145,48 | 0,2684 | -14,63 | -0,1138 | 145,37 | 0,3161 | -14,63 | -0,06834 | 145,35 | 0,3721 | -14,6 | -0,09785 |
| 145,45 | 0,2688 | -14,59 | -0,1142 | 145,34 | 0,3168 | -14,59 | -0,06865 | 145,31 | 0,3723 | -14,57 | -0,09789 |
| 145,41 | 0,2693 | -14,56 | -0,1146 | 145,31 | 0,3176 | -14,56 | -0,06916 | 145,28 | 0,3725 | -14,53 | -0,09794 |
| 145,38 | 0,2702 | -14,53 | -0,115 | 145,27 | 0,3185 | -14,53 | -0,0698 | 145,25 | 0,3728 | -14,5 | -0,09799 |
| 145,35 | 0,2713 | -14,5 | -0,1153 | 145,24 | 0,3193 | -14,5 | -0,07032 | 145,22 | 0,3732 | -14,47 | -0,09803 |
| 145,32 | 0,2723 | -14,47 | -0,1156 | 145,21 | 0,32 | -14,47 | -0,07073 | 145,18 | 0,3736 | -14,44 | -0,09808 |
| 145,29 | 0,273 | -14,43 | -0,116 | 145,18 | 0,3208 | -14,43 | -0,07111 | 145,15 | 0,3741 | -14,4 | -0,0981 |
| 145,25 | 0,2735 | -14,4 | -0,1165 | 145,14 | 0,3215 | -14,4 | -0,07149 | 145,12 | 0,3745 | -14,37 | -0,09813 |
| 145,22 | 0,2742 | -14,37 | -0,1169 | 145,11 | 0,3221 | -14,37 | -0,072 | 145,08 | 0,3749 | -14,34 | -0,09818 |
| 145,19 | 0,2751 | -14,34 | -0,1172 | 145,08 | 0,3227 | -14,34 | -0,07259 | 145,05 | 0,3752 | -14,31 | -0,09823 |
| 145,16 | 0,276 | -14,31 | -0,1175 | 145,05 | 0,3232 | -14,3 | -0,07313 | 145,02 | 0,3756 | -14,28 | -0,09829 |
| 145,13 | 0,2768 | -14,27 | -0,1178 | 145,01 | 0,3237 | -14,27 | -0,07368 | 144,99 | 0,3761 | -14,24 | -0,09834 |
| 145,09 | 0,2777 | -14,24 | -0,118 | 144,98 | 0,3242 | -14,24 | -0,07409 | 144,95 | 0,3765 | -14,21 | -0,09838 |
| 145,06 | 0,2787 | -14,21 | -0,1183 | 144,95 | 0,3249 | -14,21 | -0,07448 | 144,92 | 0,3768 | -14,18 | -0,09841 |
| 145,03 | 0,2798 | -14,18 | -0,1186 | 144,92 | 0,3258 | -14,18 | -0,07486 | 144,89 | 0,377 | -14,14 | -0,09845 |
| 145 | 0,2809 | -14,14 | -0,1188 | 144,89 | 0,3268 | -14,15 | -0,07526 | 144,86 | 0,3772 | -14,11 | -0,09848 |
| 144,96 | 0,2816 | -14,11 | -0,1191 | 144,85 | 0,3276 | -14,11 | -0,07562 | 144,82 | 0,3773 | -14,08 | -0,09853 |
| 144,93 | 0,2821 | -14,08 | -0,1194 | 144,82 | 0,3284 | -14,08 | -0,07592 | 144,79 | 0,3776 | -14,05 | -0,09856 |
| 144,9 | 0,2828 | -14,05 | -0,1197 | 144,79 | 0,329 | -14,05 | -0,07619 | 144,76 | 0,3778 | -14,01 | -0,09859 |
| 144,87 | 0,2836 | -14,01 | -0,1199 | 144,76 | 0,3295 | -14,02 | -0,07645 | 144,72 | 0,3782 | -13,98 | -0,09861 |
| 144,84 | 0,2845 | -13,98 | -0,1201 | 144,72 | 0,3299 | -13,99 | -0,07675 | 144,69 | 0,3785 | -13,95 | -0,09864 |
| 144,8 | 0,2853 | -13,95 | -0,1204 | 144,69 | 0,3303 | -13,95 | -0,07708 | 144,66 | 0,3788 | -13,92 | -0,09867 |
| 144,77 | 0,2859 | -13,92 | -0,1206 | 144,66 | 0,331 | -13,92 | -0,07732 | 144,63 | 0,379 | -13,88 | -0,0987 |
| 144,74 | 0,2866 | -13,89 | -0,1209 | 144,63 | 0,3317 | -13,89 | -0,07759 | 144,59 | 0,3792 | -13,85 | -0,09873 |
| 144,71 | 0,2871 | -13,85 | -0,1212 | 144,59 | 0,3324 | -13,86 | -0,07794 | 144,56 | 0,3794 | -13,82 | -0,09876 |
| 144,67 | 0,2875 | -13,82 | -0,1215 | 144,56 | 0,333 | -13,82 | -0,07832 | 144,53 | 0,3796 | -13,79 | -0,09879 |
| 144,64 | 0,288 | -13,79 | -0,1218 | 144,53 | 0,3337 | -13,79 | -0,07868 | 144,5 | 0,3799 | -13,75 | -0,09881 |
| 144,61 | 0,2885 | -13,75 | -0,1221 | 144,5 | 0,3343 | -13,76 | -0,079 | 144,46 | 0,3801 | -13,72 | -0,09883 |
| 144,58 | 0,2893 | -13,72 | -0,1223 | 144,46 | 0,3348 | -13,73 | -0,07925 | 144,43 | 0,3804 | -13,69 | -0,09886 |
| 144,54 | 0,29 | -13,69 | -0,1225 | 144,43 | 0,3353 | -13,69 | -0,07956 | 144,4 | 0,3806 | -13,65 | -0,09889 |
| 144,51 | 0,2907 | -13,66 | -0,1226 | 144,4 | 0,3358 | -13,66 | -0,07989 | 144,37 | 0,3807 | -13,62 | -0,09892 |
| 144,48 | 0,2912 | -13,63 | -0,1228 | 144,37 | 0,3364 | -13,63 | -0,08019 | 144,33 | 0,3809 | -13,59 | -0,09895 |
| 144,45 | 0,2918 | -13,59 | -0,123 | 144,33 | 0,337 | -13,6 | -0,08046 | 144,3 | 0,3809 | -13,56 | -0,09898 |
| 144,41 | 0,2924 | -13,56 | -0,1232 | 144,3 | 0,3374 | -13,56 | -0,08068 | 144,27 | 0,381 | -13,52 | -0,09901 |
| 144,38 | 0,2931 | -13,53 | -0,1234 | 144,27 | 0,3377 | -13,53 | -0,08092 | 144,23 | 0,3812 | -13,49 | -0,09903 |
| 144,35 | 0,2939 | -13,5 | -0,1236 | 144,24 | 0,338 | -13,5 | -0,08118 | 144,2 | 0,3815 | -13,46 | -0,09906 |
| 144,32 | 0,2949 | -13,46 | -0,1238 | 144,2 | 0,3385 | -13,47 | -0,08141 | 144,17 | 0,3818 | -13,43 | -0,09909 |
| 144,28 | 0,2957 | -13,43 | -0,1239 | 144,17 | 0,3391 | -13,43 | -0,08172 | 144,13 | 0,382 | -13,39 | -0,09912 |
| 144,25 | 0,2962 | -13,4 | -0,1241 | 144,14 | 0,3398 | -13,4 | -0,082 | 144,1 | 0,3821 | -13,36 | -0,09915 |
| 144,22 | 0,2967 | -13,37 | -0,1243 | 144,11 | 0,3403 | -13,37 | -0,08227 | 144,07 | 0,3823 | -13,33 | -0,09918 |
| 144,19 | 0,2971 | -13,33 | -0,1245 | 144,07 | 0,3409 | -13,34 | -0,08252 | 144,04 | 0,3824 | -13,3 | -0,09921 |
| 144,15 | 0,2976 | -13,3 | -0,1246 | 144,04 | 0,3415 | -13,3 | -0,08272 | 144 | 0,3827 | -13,26 | -0,09924 |
| 144,12 | 0,2982 | -13,27 | -0,1248 | 144,01 | 0,342 | -13,27 | -0,08293 | 143,97 | 0,3829 | -13,23 | -0,09927 |
| 144,09 | 0,2989 | -13,24 | -0,1251 | 143,97 | 0,3423 | -13,24 | -0,08314 | 143,94 | 0,383 | -13,2 | -0,09929 |
| 144,06 | 0,2995 | -13,2 | -0,1252 | 143,94 | 0,3427 | -13,2 | -0,08334 | 143,9 | 0,3831 | -13,16 | -0,09932 |
| 144,02 | 0,3 | -13,17 | -0,1254 | 143,91 | 0,343 | -13,17 | -0,08358 | 143,87 | 0,3832 | -13,13 | -0,09935 |
| 143,99 | 0,3005 | -13,14 | -0,1255 | 143,88 | 0,3433 | -13,14 | -0,08382 | 143,84 | 0,3833 | -13,1 | -0,09938 |
| 143,96 | 0,3011 | -13,1 | -0,1257 | 143,85 | 0,3437 | -13,11 | -0,0841 | 143,8 | 0,3834 | -13,07 | -0,09941 |
| 143,93 | 0,3018 | -13,07 | -0,1259 | 143,81 | 0,344 | -13,08 | -0,0844 | 143,77 | 0,3835 | -13,03 | -0,09944 |
| 143,89 | 0,3023 | -13,04 | -0,126 | 143,78 | 0,3444 | -13,04 | -0,08468 | 143,74 | 0,3836 | -13 | -0,09947 |
| 143,86 | 0,3028 | -13,01 | -0,1262 | 143,75 | 0,3447 | -13,01 | -0,08493 | 143,71 | 0,3837 | -12,97 | -0,0995 |
| 143,83 | 0,3031 | -12,97 | -0,1263 | 143,71 | 0,345 | -12,98 | -0,08514 | 143,67 | 0,3837 | -12,93 | -0,09953 |
| 143,79 | 0,3034 | -12,94 | -0,1265 | 143,68 | 0,3453 | -12,95 | -0,08528 | 143,64 | 0,3838 | -12,9 | -0,09956 |
| 143,76 | 0,3038 | -12,91 | -0,1265 | 143,65 | 0,3456 | -12,91 | -0,08542 | 143,61 | 0,3839 | -12,87 | -0,09959 |
| 143,73 | 0,3041 | -12,88 | -0,1266 | 143,62 | 0,346 | -12,88 | -0,08555 | 143,57 | 0,384 | -12,84 | -0,09962 |
| 143,7 | 0,3046 | -12,84 | -0,1268 | 143,58 | 0,3464 | -12,85 | -0,08569 | 143,54 | 0,3842 | -12,8 | -0,09964 |
| 143,66 | 0,3052 | -12,81 | -0,127 | 143,55 | 0,3467 | -12,81 | -0,08589 | 143,51 | 0,3843 | -12,77 | -0,09967 |
| 143,63 | 0,306 | -12,78 | -0,1272 | 143,52 | 0,3472 | -12,78 | -0,0861 | 143,48 | 0,3844 | -12,74 | -0,0997 |
| 143,6 | 0,3066 | -12,75 | -0,1274 | 143,49 | 0,3477 | -12,75 | -0,0863 | 143,44 | 0,3845 | -12,7 | -0,09973 |
| 143,57 | 0,307 | -12,71 | -0,1275 | 143,45 | 0,3482 | -12,72 | -0,08655 | 143,41 | 0,3845 | -12,67 | -0,09976 |
| 143,53 | 0,3074 | -12,68 | -0,1276 | 143,42 | 0,3486 | -12,68 | -0,08678 | 143,38 | 0,3846 | -12,64 | -0,09979 |
| 143,5 | 0,3078 | -12,65 | -0,1278 | 143,39 | 0,3489 | -12,65 | -0,08703 | 143,34 | 0,3847 | -12,61 | -0,09982 |
| 143,47 | 0,3081 | -12,61 | -0,1279 | 143,35 | 0,349 | -12,62 | -0,08729 | 143,31 | 0,3848 | -12,57 | -0,09986 |
| 143,44 | 0,3085 | -12,58 | -0,128 | 143,32 | 0,3493 | -12,59 | -0,08749 | 143,28 | 0,3849 | -12,54 | -0,09989 |
| 143,4 | 0,3088 | -12,55 | -0,1281 | 143,29 | 0,3497 | -12,55 | -0,08764 | 143,24 | 0,3849 | -12,51 | -0,09992 |
| 143,37 | 0,3091 | -12,52 | -0,1282 | 143,25 | 0,3501 | -12,52 | -0,08779 | 143,21 | 0,385 | -12,47 | -0,09995 |
| 143,34 | 0,3095 | -12,48 | -0,1283 | 143,22 | 0,3504 | -12,49 | -0,08792 | 143,18 | 0,3851 | -12,44 | -0,09998 |
| 143,3 | 0,3099 | -12,45 | -0,1285 | 143,19 | 0,3506 | -12,46 | -0,08811 | 143,14 | 0,3851 | -12,41 | -0,1 |
| 143,27 | 0,3104 | -12,42 | -0,1286 | 143,16 | 0,3509 | -12,42 | -0,08833 | 143,11 | 0,3852 | -12,38 | -0,1 |
| 143,24 | 0,3109 | -12,38 | -0,1287 | 143,12 | 0,3512 | -12,39 | -0,08853 | 143,08 | 0,3853 | -12,34 | -0,1001 |
| 143,21 | 0,3115 | -12,35 | -0,1289 | 143,09 | 0,3515 | -12,36 | -0,08872 | 143,05 | 0,3853 | -12,31 | -0,1001 |
| 143,17 | 0,3119 | -12,32 | -0,1291 | 143,06 | 0,3517 | -12,32 | -0,08885 | 143,01 | 0,3854 | -12,28 | -0,1001 |
| 143,14 | 0,3124 | -12,28 | -0,1293 | 143,03 | 0,3519 | -12,29 | -0,08896 | 142,98 | 0,3855 | -12,24 | -0,1002 |
| 143,11 | 0,3128 | -12,25 | -0,1295 | 142,99 | 0,3522 | -12,26 | -0,08909 | 142,95 | 0,3855 | -12,21 | -0,1002 |
| 143,08 | 0,3132 | -12,22 | -0,1295 | 142,96 | 0,3524 | -12,23 | -0,08924 | 142,91 | 0,3856 | -12,18 | -0,1002 |
| 143,04 | 0,3136 | -12,19 | -0,1296 | 142,93 | 0,3527 | -12,19 | -0,08939 | 142,88 | 0,3856 | -12,14 | -0,1003 |
| 143,01 | 0,3139 | -12,16 | -0,1296 | 142,89 | 0,353 | -12,16 | -0,08955 | 142,85 | 0,3856 | -12,11 | -0,1003 |
| 142,98 | 0,3142 | -12,12 | -0,1297 | 142,86 | 0,3533 | -12,13 | -0,08971 | 142,81 | 0,3857 | -12,08 | -0,1003 |
| 142,94 | 0,3146 | -12,09 | -0,1298 | 142,83 | 0,3534 | -12,09 | -0,0899 | 142,78 | 0,3857 | -12,05 | -0,1004 |
| 142,91 | 0,3149 | -12,06 | -0,1299 | 142,8 | 0,3536 | -12,06 | -0,09008 | 142,75 | 0,3857 | -12,01 | -0,1004 |
| 142,88 | 0,3153 | -12,02 | -0,13 | 142,76 | 0,3539 | -12,03 | -0,09026 | 142,72 | 0,3858 | -11,98 | -0,1005 |
| 142,85 | 0,3158 | -11,99 | -0,1302 | 142,73 | 0,3541 | -11,99 | -0,09041 | 142,68 | 0,3858 | -11,95 | -0,1005 |
| 142,81 | 0,3162 | -11,96 | -0,1303 | 142,7 | 0,3545 | -11,96 | -0,09053 | 142,65 | 0,3859 | -11,91 | -0,1005 |
| 142,78 | 0,3165 | -11,92 | -0,1304 | 142,66 | 0,3547 | -11,93 | -0,09065 | 142,62 | 0,386 | -11,88 | -0,1006 |
| 142,75 | 0,3168 | -11,89 | -0,1305 | 142,63 | 0,3549 | -11,9 | -0,09077 | 142,58 | 0,386 | -11,85 | -0,1006 |
| 142,71 | 0,317 | -11,86 | -0,1305 | 142,6 | 0,3551 | -11,86 | -0,0909 | 142,55 | 0,3861 | -11,81 | -0,1006 |
| 142,68 | 0,3172 | -11,83 | -0,1306 | 142,56 | 0,3552 | -11,83 | -0,09107 | 142,52 | 0,3861 | -11,78 | -0,1007 |
| 142,65 | 0,3175 | -11,79 | -0,1307 | 142,53 | 0,3555 | -11,8 | -0,09122 | 142,48 | 0,3861 | -11,75 | -0,1007 |
| 142,62 | 0,3178 | -11,76 | -0,1308 | 142,5 | 0,3558 | -11,77 | -0,09135 | 142,45 | 0,3861 | -11,72 | -0,1007 |
| 142,58 | 0,3181 | -11,73 | -0,1309 | 142,47 | 0,3561 | -11,73 | -0,09149 | 142,42 | 0,3862 | -11,68 | -0,1008 |
| 142,55 | 0,3184 | -11,69 | -0,131 | 142,43 | 0,3562 | -11,7 | -0,0916 | 142,39 | 0,3862 | -11,65 | -0,1008 |
| 142,52 | 0,3187 | -11,66 | -0,1312 | 142,4 | 0,3564 | -11,67 | -0,09172 | 142,35 | 0,3863 | -11,62 | -0,1008 |
| 142,48 | 0,319 | -11,63 | -0,1314 | 142,37 | 0,3565 | -11,63 | -0,09187 | 142,32 | 0,3863 | -11,58 | -0,1009 |
| 142,45 | 0,3193 | -11,6 | -0,1315 | 142,33 | 0,3567 | -11,6 | -0,09199 | 142,29 | 0,3863 | -11,55 | -0,1009 |
| 142,42 | 0,3196 | -11,56 | -0,1316 | 142,3 | 0,3568 | -11,57 | -0,09211 | 142,25 | 0,3864 | -11,52 | -0,1009 |
| 142,39 | 0,3199 | -11,53 | -0,1317 | 142,27 | 0,357 | -11,54 | -0,09223 | 142,22 | 0,3864 | -11,49 | -0,101 |
| 142,35 | 0,3203 | -11,5 | -0,1318 | 142,24 | 0,3571 | -11,5 | -0,09235 | 142,19 | 0,3864 | -11,45 | -0,101 |
| 142,32 | 0,3206 | -11,46 | -0,1319 | 142,2 | 0,3573 | -11,47 | -0,09247 | 142,15 | 0,3864 | -11,42 | -0,101 |
| 142,29 | 0,321 | -11,43 | -0,132 | 142,17 | 0,3576 | -11,44 | -0,09257 | 142,12 | 0,3865 | -11,38 | -0,1011 |
| 142,25 | 0,3214 | -11,4 | -0,1321 | 142,14 | 0,3578 | -11,4 | -0,09265 | 142,09 | 0,3865 | -11,35 | -0,1011 |
| 142,22 | 0,3217 | -11,37 | -0,1322 | 142,1 | 0,358 | -11,37 | -0,09274 | 142,06 | 0,3866 | -11,32 | -0,1012 |
| 142,19 | 0,3219 | -11,33 | -0,1323 | 142,07 | 0,3581 | -11,34 | -0,09286 | 142,02 | 0,3866 | -11,29 | -0,1012 |
| 142,15 | 0,3221 | -11,3 | -0,1323 | 142,04 | 0,3583 | -11,3 | -0,09299 | 141,99 | 0,3866 | -11,25 | -0,1012 |
| 142,12 | 0,3223 | -11,27 | -0,1324 | 142 | 0,3584 | -11,27 | -0,09311 | 141,95 | 0,3867 | -11,22 | -0,1013 |
| 142,09 | 0,3226 | -11,23 | -0,1324 | 141,97 | 0,3586 | -11,24 | -0,0932 | 141,92 | 0,3867 | -11,19 | -0,1013 |
| 142,06 | 0,3229 | -11,2 | -0,1325 | 141,94 | 0,3587 | -11,21 | -0,09327 | 141,89 | 0,3867 | -11,15 | -0,1013 |
| 142,02 | 0,3231 | -11,17 | -0,1325 | 141,91 | 0,3588 | -11,17 | -0,09334 | 141,86 | 0,3868 | -11,12 | -0,1014 |
| 141,99 | 0,3233 | -11,13 | -0,1326 | 141,87 | 0,3589 | -11,14 | -0,09345 | 141,82 | 0,3868 | -11,09 | -0,1014 |
| 141,96 | 0,3235 | -11,1 | -0,1326 | 141,84 | 0,359 | -11,11 | -0,09356 | 141,79 | 0,3869 | -11,05 | -0,1014 |
| 141,92 | 0,3237 | -11,07 | -0,1327 | 141,81 | 0,3592 | -11,07 | -0,0937 | 141,76 | 0,3869 | -11,02 | -0,1015 |
| 141,89 | 0,3239 | -11,04 | -0,1327 | 141,77 | 0,3594 | -11,04 | -0,09382 | 141,72 | 0,3869 | -10,99 | -0,1015 |
| 141,86 | 0,3241 | -11 | -0,1328 | 141,74 | 0,3595 | -11,01 | -0,0939 | 141,69 | 0,387 | -10,95 | -0,1015 |
| 141,83 | 0,3244 | -10,97 | -0,1329 | 141,71 | 0,3596 | -10,98 | -0,09395 | 141,66 | 0,387 | -10,92 | -0,1016 |
| 141,79 | 0,3247 | -10,94 | -0,133 | 141,68 | 0,3597 | -10,94 | -0,09401 | 141,62 | 0,387 | -10,89 | -0,1016 |
| 141,76 | 0,325 | -10,9 | -0,1331 | 141,64 | 0,3599 | -10,91 | -0,09407 | 141,59 | 0,3871 | -10,86 | -0,1017 |
| 141,73 | 0,3253 | -10,87 | -0,1331 | 141,61 | 0,36 | -10,87 | -0,09411 | 141,56 | 0,3871 | -10,82 | -0,1017 |
| 141,69 | 0,3255 | -10,84 | -0,1332 | 141,58 | 0,36 | -10,84 | -0,09416 | 141,52 | 0,3871 | -10,79 | -0,1017 |
| 141,66 | 0,3257 | -10,8 | -0,1332 | 141,54 | 0,3601 | -10,81 | -0,09421 | 141,49 | 0,3871 | -10,76 | -0,1018 |
| 141,63 | 0,3259 | -10,77 | -0,1333 | 141,51 | 0,3602 | -10,78 | -0,09427 | 141,46 | 0,3872 | -10,72 | -0,1018 |
| 141,6 | 0,3261 | -10,74 | -0,1334 | 141,48 | 0,3604 | -10,74 | -0,09437 | 141,42 | 0,3872 | -10,69 | -0,1018 |
| 141,56 | 0,3263 | -10,71 | -0,1335 | 141,44 | 0,3605 | -10,71 | -0,09446 | 141,39 | 0,3872 | -10,66 | -0,1019 |
| 141,53 | 0,3265 | -10,67 | -0,1336 | 141,41 | 0,3606 | -10,68 | -0,09454 | 141,36 | 0,3873 | -10,62 | -0,1019 |
| 141,5 | 0,3267 | -10,64 | -0,1336 | 141,38 | 0,3607 | -10,65 | -0,09461 | 141,33 | 0,3873 | -10,59 | -0,1019 |
| 141,46 | 0,327 | -10,61 | -0,1337 | 141,34 | 0,3609 | -10,61 | -0,09466 | 141,29 | 0,3873 | -10,56 | -0,102 |
| 141,43 | 0,3272 | -10,57 | -0,1337 | 141,31 | 0,361 | -10,58 | -0,09472 | 141,26 | 0,3873 | -10,52 | -0,102 |
| 141,4 | 0,3276 | -10,54 | -0,1338 | 141,28 | 0,3611 | -10,55 | -0,09478 | 141,23 | 0,3874 | -10,49 | -0,102 |
| 141,37 | 0,3279 | -10,51 | -0,1339 | 141,24 | 0,3612 | -10,51 | -0,09483 | 141,19 | 0,3874 | -10,46 | -0,1021 |
| 141,33 | 0,3281 | -10,47 | -0,1339 | 141,21 | 0,3613 | -10,48 | -0,09488 | 141,16 | 0,3874 | -10,43 | -0,1021 |
| 141,3 | 0,3283 | -10,44 | -0,134 | 141,18 | 0,3614 | -10,45 | -0,09491 | 141,13 | 0,3875 | -10,39 | -0,1021 |
| 141,27 | 0,3284 | -10,41 | -0,134 | 141,15 | 0,3615 | -10,41 | -0,09496 | 141,09 | 0,3875 | -10,36 | -0,1022 |
| 141,23 | 0,3285 | -10,37 | -0,134 | 141,11 | 0,3616 | -10,38 | -0,09502 | 141,06 | 0,3875 | -10,33 | -0,1022 |
| 141,2 | 0,3287 | -10,34 | -0,1341 | 141,08 | 0,3617 | -10,35 | -0,09511 | 141,03 | 0,3876 | -10,29 | -0,1023 |
| 141,17 | 0,3288 | -10,31 | -0,1341 | 141,05 | 0,3617 | -10,32 | -0,09519 | 140,99 | 0,3876 | -10,26 | -0,1023 |
| 141,13 | 0,3289 | -10,28 | -0,1342 | 141,01 | 0,3618 | -10,28 | -0,09525 | 140,96 | 0,3876 | -10,23 | -0,1023 |
| 141,1 | 0,3291 | -10,24 | -0,1342 | 140,98 | 0,3619 | -10,25 | -0,0953 | 140,93 | 0,3877 | -10,19 | -0,1023 |
| 141,07 | 0,3292 | -10,21 | -0,1343 | 140,95 | 0,362 | -10,22 | -0,09535 | 140,89 | 0,3877 | -10,16 | -0,1024 |
| 141,04 | 0,3295 | -10,18 | -0,1343 | 140,92 | 0,3621 | -10,18 | -0,09541 | 140,86 | 0,3877 | -10,13 | -0,1024 |
| 141 | 0,3297 | -10,14 | -0,1344 | 140,88 | 0,3621 | -10,15 | -0,09546 | 140,83 | 0,3878 | -10,1 | -0,1024 |
| 140,97 | 0,33 | -10,11 | -0,1344 | 140,85 | 0,3622 | -10,12 | -0,0955 | 140,8 | 0,3878 | -10,06 | -0,1025 |
| 140,94 | 0,3302 | -10,08 | -0,1344 | 140,82 | 0,3623 | -10,08 | -0,09555 | 140,76 | 0,3878 | -10,03 | -0,1025 |
| 140,9 | 0,3304 | -10,04 | -0,1345 | 140,78 | 0,3623 | -10,05 | -0,0956 | 140,73 | 0,3879 | -10 | -0,1026 |
| 140,87 | 0,3306 | -10,01 | -0,1345 | 140,75 | 0,3624 | -10,02 | -0,09565 | 140,7 | 0,3879 | -9,96 | -0,1026 |
| 140,84 | 0,3308 | -9,98 | -0,1345 | 140,72 | 0,3624 | -9,99 | -0,09571 | 140,66 | 0,3879 | -9,93 | -0,1026 |
| 140,8 | 0,331 | -9,95 | -0,1345 | 140,68 | 0,3625 | -9,95 | -0,09576 | 140,63 | 0,3879 | -9,9 | -0,1027 |
| 140,77 | 0,3311 | -9,91 | -0,1346 | 140,65 | 0,3626 | -9,92 | -0,09581 | 140,6 | 0,388 | -9,86 | -0,1027 |
| 140,74 | 0,3313 | -9,88 | -0,1346 | 140,62 | 0,3626 | -9,89 | -0,09584 | 140,56 | 0,388 | -9,83 | -0,1027 |
| 140,71 | 0,3315 | -9,85 | -0,1347 | 140,58 | 0,3627 | -9,85 | -0,09587 | 140,53 | 0,388 | -9,8 | -0,1028 |
| 140,67 | 0,3317 | -9,81 | -0,1348 | 140,55 | 0,3628 | -9,82 | -0,0959 | 140,5 | 0,3881 | -9,76 | -0,1028 |
| 140,64 | 0,3319 | -9,78 | -0,1349 | 140,52 | 0,3629 | -9,79 | -0,09593 | 140,46 | 0,3881 | -9,73 | -0,1028 |
| 140,61 | 0,332 | -9,75 | -0,1349 | 140,49 | 0,3629 | -9,75 | -0,09595 | 140,43 | 0,3881 | -9,7 | -0,1029 |
| 140,57 | 0,3321 | -9,71 | -0,1349 | 140,45 | 0,363 | -9,72 | -0,096 | 140,4 | 0,3881 | -9,66 | -0,1029 |
| 140,54 | 0,3322 | -9,68 | -0,1349 | 140,42 | 0,3631 | -9,69 | -0,09606 | 140,36 | 0,3882 | -9,63 | -0,1029 |
| 140,51 | 0,3323 | -9,65 | -0,135 | 140,39 | 0,3631 | -9,65 | -0,09612 | 140,33 | 0,3882 | -9,6 | -0,103 |
| 140,47 | 0,3325 | -9,61 | -0,135 | 140,35 | 0,3632 | -9,62 | -0,09616 | 140,3 | 0,3882 | -9,56 | -0,103 |
| 140,44 | 0,3326 | -9,58 | -0,135 | 140,32 | 0,3632 | -9,59 | -0,09619 | 140,27 | 0,3883 | -9,53 | -0,103 |
| 140,41 | 0,3328 | -9,55 | -0,1351 | 140,29 | 0,3632 | -9,55 | -0,09624 | 140,23 | 0,3883 | -9,5 | -0,1031 |
| 140,37 | 0,333 | -9,51 | -0,1351 | 140,25 | 0,3633 | -9,52 | -0,09629 | 140,2 | 0,3883 | -9,46 | -0,1031 |
| 140,34 | 0,3332 | -9,48 | -0,1352 | 140,22 | 0,3634 | -9,49 | -0,09633 | 140,17 | 0,3884 | -9,43 | -0,1032 |
| 140,31 | 0,3333 | -9,45 | -0,1352 | 140,19 | 0,3634 | -9,45 | -0,09637 | 140,13 | 0,3884 | -9,4 | -0,1032 |
| 140,28 | 0,3335 | -9,42 | -0,1353 | 140,15 | 0,3635 | -9,42 | -0,09641 | 140,1 | 0,3884 | -9,37 | -0,1032 |
| 140,24 | 0,3336 | -9,38 | -0,1353 | 140,12 | 0,3636 | -9,39 | -0,09645 | 140,07 | 0,3885 | -9,33 | -0,1033 |
| 140,21 | 0,3337 | -9,35 | -0,1353 | 140,09 | 0,3636 | -9,36 | -0,09651 | 140,03 | 0,3885 | -9,3 | -0,1033 |
| 140,18 | 0,3338 | -9,32 | -0,1353 | 140,05 | 0,3636 | -9,32 | -0,09656 | 140 | 0,3885 | -9,27 | -0,1033 |
| 140,14 | 0,3339 | -9,28 | -0,1354 | 140,02 | 0,3636 | -9,29 | -0,0966 | 139,96 | 0,3885 | -9,23 | -0,1034 |
| 140,11 | 0,3341 | -9,25 | -0,1354 | 139,99 | 0,3637 | -9,26 | -0,09664 | 139,93 | 0,3886 | -9,2 | -0,1034 |
| 140,08 | 0,3342 | -9,22 | -0,1354 | 139,95 | 0,3637 | -9,22 | -0,09669 | 139,9 | 0,3886 | -9,17 | -0,1034 |
| 140,04 | 0,3343 | -9,18 | -0,1355 | 139,92 | 0,3638 | -9,19 | -0,09672 | 139,87 | 0,3886 | -9,13 | -0,1035 |
| 140,01 | 0,3344 | -9,15 | -0,1355 | 139,89 | 0,3638 | -9,16 | -0,09675 | 139,83 | 0,3887 | -9,1 | -0,1035 |
| 139,98 | 0,3345 | -9,12 | -0,1355 | 139,86 | 0,3638 | -9,12 | -0,09678 | 139,8 | 0,3887 | -9,07 | -0,1035 |
| 139,95 | 0,3347 | -9,08 | -0,1355 | 139,82 | 0,3639 | -9,09 | -0,09681 | 139,77 | 0,3887 | -9,03 | -0,1036 |
| 139,91 | 0,3348 | -9,05 | -0,1356 | 139,79 | 0,3639 | -9,06 | -0,09689 | 139,73 | 0,3887 | -9 | -0,1036 |
| 139,88 | 0,3349 | -9,02 | -0,1356 | 139,76 | 0,364 | -9,02 | -0,09697 | 139,7 | 0,3888 | -8,97 | -0,1036 |
| 139,85 | 0,335 | -8,98 | -0,1356 | 139,72 | 0,364 | -8,99 | -0,09703 | 139,67 | 0,3888 | -8,93 | -0,1037 |
| 139,81 | 0,3351 | -8,95 | -0,1357 | 139,69 | 0,3641 | -8,96 | -0,09708 | 139,63 | 0,3888 | -8,9 | -0,1037 |
| 139,78 | 0,3352 | -8,92 | -0,1357 | 139,66 | 0,3641 | -8,92 | -0,09711 | 139,6 | 0,3888 | -8,87 | -0,1037 |
| 139,75 | 0,3354 | -8,89 | -0,1357 | 139,62 | 0,3641 | -8,89 | -0,09714 | 139,57 | 0,3889 | -8,84 | -0,1038 |
| 139,71 | 0,3356 | -8,85 | -0,1358 | 139,59 | 0,3642 | -8,86 | -0,09717 | 139,53 | 0,3889 | -8,8 | -0,1038 |
| 139,68 | 0,3357 | -8,82 | -0,1358 | 139,56 | 0,3642 | -8,82 | -0,0972 | 139,5 | 0,3889 | -8,77 | -0,1038 |
| 139,65 | 0,3359 | -8,79 | -0,1358 | 139,52 | 0,3642 | -8,79 | -0,09723 | 139,47 | 0,389 | -8,74 | -0,1039 |
| 139,62 | 0,336 | -8,75 | -0,1359 | 139,49 | 0,3643 | -8,76 | -0,09728 | 139,43 | 0,389 | -8,7 | -0,1039 |
| 139,58 | 0,3361 | -8,72 | -0,1359 | 139,46 | 0,3643 | -8,73 | -0,09734 | 139,4 | 0,389 | -8,67 | -0,1039 |
| 139,55 | 0,3362 | -8,69 | -0,1359 | 139,42 | 0,3643 | -8,69 | -0,09739 | 139,37 | 0,389 | -8,64 | -0,104 |
| 139,52 | 0,3363 | -8,65 | -0,136 | 139,39 | 0,3644 | -8,66 | -0,09742 | 139,34 | 0,3891 | -8,6 | -0,104 |
| 139,48 | 0,3365 | -8,62 | -0,136 | 139,36 | 0,3644 | -8,63 | -0,09745 | 139,3 | 0,3891 | -8,57 | -0,104 |
| 139,45 | 0,3367 | -8,59 | -0,136 | 139,33 | 0,3644 | -8,59 | -0,09748 | 139,27 | 0,3891 | -8,54 | -0,1041 |
| 139,42 | 0,3368 | -8,55 | -0,1361 | 139,29 | 0,3645 | -8,56 | -0,09751 | 139,24 | 0,3892 | -8,5 | -0,1041 |
| 139,38 | 0,3369 | -8,52 | -0,1361 | 139,26 | 0,3645 | -8,53 | -0,09754 | 139,2 | 0,3892 | -8,47 | -0,1041 |
| 139,35 | 0,3369 | -8,49 | -0,1362 | 139,22 | 0,3645 | -8,49 | -0,09757 | 139,17 | 0,3892 | -8,44 | -0,1042 |
| 139,32 | 0,337 | -8,45 | -0,1362 | 139,19 | 0,3646 | -8,46 | -0,09761 | 139,14 | 0,3892 | -8,4 | -0,1042 |
| 139,28 | 0,3371 | -8,42 | -0,1363 | 139,16 | 0,3646 | -8,43 | -0,09764 | 139,1 | 0,3893 | -8,37 | -0,1043 |
| 139,25 | 0,3372 | -8,39 | -0,1363 | 139,13 | 0,3647 | -8,39 | -0,09767 | 139,07 | 0,3893 | -8,34 | -0,1043 |
| 139,22 | 0,3373 | -8,35 | -0,1363 | 139,09 | 0,3647 | -8,36 | -0,09771 | 139,04 | 0,3893 | -8,3 | -0,1043 |
| 139,18 | 0,3375 | -8,32 | -0,1363 | 139,06 | 0,3647 | -8,33 | -0,09774 | 139 | 0,3893 | -8,27 | -0,1043 |
| 139,15 | 0,3376 | -8,29 | -0,1364 | 139,03 | 0,3647 | -8,3 | -0,09778 | 138,97 | 0,3894 | -8,24 | -0,1044 |
| 139,12 | 0,3378 | -8,25 | -0,1364 | 138,99 | 0,3648 | -8,26 | -0,09782 | 138,94 | 0,3894 | -8,2 | -0,1044 |
| 139,09 | 0,3379 | -8,22 | -0,1364 | 138,96 | 0,3648 | -8,23 | -0,09785 | 138,9 | 0,3894 | -8,17 | -0,1044 |
| 139,05 | 0,338 | -8,19 | -0,1365 | 138,93 | 0,3648 | -8,19 | -0,09789 | 138,87 | 0,3894 | -8,14 | -0,1045 |
| 139,02 | 0,3381 | -8,16 | -0,1365 | 138,89 | 0,3649 | -8,16 | -0,09792 | 138,84 | 0,3895 | -8,1 | -0,1045 |
| 138,99 | 0,3381 | -8,12 | -0,1365 | 138,86 | 0,3649 | -8,13 | -0,09795 | 138,81 | 0,3895 | -8,07 | -0,1045 |
| 138,95 | 0,3382 | -8,09 | -0,1366 | 138,83 | 0,3649 | -8,1 | -0,09799 | 138,77 | 0,3895 | -8,04 | -0,1046 |
| 138,92 | 0,3383 | -8,06 | -0,1366 | 138,79 | 0,3649 | -8,06 | -0,09805 | 138,74 | 0,3895 | -8 | -0,1046 |
| 138,89 | 0,3384 | -8,02 | -0,1366 | 138,76 | 0,365 | -8,03 | -0,0981 | 138,7 | 0,3896 | -7,97 | -0,1046 |
| 138,85 | 0,3385 | -7,99 | -0,1367 | 138,73 | 0,365 | -8 | -0,09813 | 138,67 | 0,3896 | -7,94 | -0,1047 |
| 138,82 | 0,3386 | -7,96 | -0,1367 | 138,7 | 0,365 | -7,96 | -0,09817 | 138,64 | 0,3896 | -7,91 | -0,1047 |
| 138,79 | 0,3387 | -7,92 | -0,1367 | 138,66 | 0,3651 | -7,93 | -0,0982 | 138,61 | 0,3896 | -7,87 | -0,1047 |
| 138,75 | 0,3388 | -7,89 | -0,1368 | 138,63 | 0,3651 | -7,9 | -0,09823 | 138,57 | 0,3897 | -7,84 | -0,1048 |
| 138,72 | 0,3389 | -7,86 | -0,1368 | 138,59 | 0,3651 | -7,86 | -0,09826 | 138,54 | 0,3897 | -7,81 | -0,1048 |
| 138,69 | 0,339 | -7,82 | -0,1368 | 138,56 | 0,3651 | -7,83 | -0,0983 | 138,51 | 0,3897 | -7,77 | -0,1048 |
| 138,65 | 0,339 | -7,79 | -0,1369 | 138,53 | 0,3652 | -7,8 | -0,09834 | 138,47 | 0,3898 | -7,74 | -0,1049 |
| 138,62 | 0,3391 | -7,76 | -0,1369 | 138,5 | 0,3652 | -7,77 | -0,09838 | 138,44 | 0,3898 | -7,71 | -0,1049 |
| 138,59 | 0,3392 | -7,72 | -0,1369 | 138,46 | 0,3652 | -7,73 | -0,09842 | 138,4 | 0,3898 | -7,67 | -0,1049 |
| 138,56 | 0,3393 | -7,69 | -0,137 | 138,43 | 0,3653 | -7,7 | -0,09845 | 138,37 | 0,3898 | -7,64 | -0,105 |
| 138,52 | 0,3393 | -7,66 | -0,137 | 138,4 | 0,3653 | -7,66 | -0,09848 | 138,34 | 0,3898 | -7,61 | -0,105 |
| 138,49 | 0,3394 | -7,62 | -0,137 | 138,36 | 0,3653 | -7,63 | -0,09852 | 138,31 | 0,3899 | -7,57 | -0,105 |
| 138,46 | 0,3395 | -7,59 | -0,1371 | 138,33 | 0,3654 | -7,6 | -0,09855 | 138,27 | 0,3899 | -7,54 | -0,1051 |
| 138,42 | 0,3396 | -7,56 | -0,1371 | 138,3 | 0,3654 | -7,57 | -0,09859 | 138,24 | 0,3899 | -7,51 | -0,1051 |
| 138,39 | 0,3397 | -7,52 | -0,1371 | 138,26 | 0,3654 | -7,53 | -0,09862 | 138,21 | 0,3899 | -7,47 | -0,1051 |
| 138,36 | 0,3397 | -7,49 | -0,1372 | 138,23 | 0,3655 | -7,5 | -0,09865 | 138,17 | 0,39 | -7,44 | -0,1052 |
| 138,32 | 0,3398 | -7,46 | -0,1372 | 138,2 | 0,3655 | -7,47 | -0,0987 | 138,14 | 0,39 | -7,41 | -0,1052 |
| 138,29 | 0,3399 | -7,42 | -0,1372 | 138,16 | 0,3655 | -7,43 | -0,09876 | 138,11 | 0,39 | -7,37 | -0,1052 |
| 138,26 | 0,34 | -7,39 | -0,1373 | 138,13 | 0,3655 | -7,4 | -0,09882 | 138,07 | 0,39 | -7,34 | -0,1053 |
| 138,22 | 0,3401 | -7,36 | -0,1373 | 138,1 | 0,3656 | -7,37 | -0,09886 | 138,04 | 0,3901 | -7,31 | -0,1053 |
| 138,19 | 0,3402 | -7,32 | -0,1373 | 138,06 | 0,3656 | -7,33 | -0,09889 | 138,01 | 0,3901 | -7,27 | -0,1053 |
| 138,16 | 0,3403 | -7,29 | -0,1374 | 138,03 | 0,3656 | -7,3 | -0,09893 | 137,97 | 0,3901 | -7,24 | -0,1054 |
| 138,12 | 0,3404 | -7,26 | -0,1374 | 138 | 0,3656 | -7,27 | -0,09896 | 137,94 | 0,3901 | -7,21 | -0,1054 |
| 138,09 | 0,3405 | -7,23 | -0,1374 | 137,96 | 0,3657 | -7,23 | -0,09899 | 137,91 | 0,3901 | -7,17 | -0,1054 |
| 138,06 | 0,3406 | -7,19 | -0,1375 | 137,93 | 0,3657 | -7,2 | -0,09903 | 137,87 | 0,3902 | -7,14 | -0,1055 |
| 138,03 | 0,3407 | -7,16 | -0,1375 | 137,9 | 0,3657 | -7,17 | -0,09907 | 137,84 | 0,3902 | -7,11 | -0,1055 |
| 137,99 | 0,3408 | -7,13 | -0,1375 | 137,87 | 0,3658 | -7,13 | -0,09912 | 137,81 | 0,3902 | -7,07 | -0,1055 |
| 137,96 | 0,3409 | -7,09 | -0,1376 | 137,83 | 0,3658 | -7,1 | -0,09915 | 137,77 | 0,3902 | -7,04 | -0,1056 |
| 137,93 | 0,3409 | -7,06 | -0,1376 | 137,8 | 0,3658 | -7,07 | -0,09919 | 137,74 | 0,3903 | -7,01 | -0,1056 |
| 137,89 | 0,341 | -7,03 | -0,1376 | 137,76 | 0,3659 | -7,03 | -0,09922 | 137,71 | 0,3903 | -6,97 | -0,1056 |
| 137,86 | 0,3411 | -6,99 | -0,1377 | 137,73 | 0,3659 | -7 | -0,09926 | 137,68 | 0,3903 | -6,94 | -0,1057 |
| 137,83 | 0,3412 | -6,96 | -0,1377 | 137,7 | 0,3659 | -6,97 | -0,0993 | 137,64 | 0,3903 | -6,91 | -0,1057 |
| 137,79 | 0,3413 | -6,93 | -0,1377 | 137,67 | 0,3659 | -6,93 | -0,09933 | 137,61 | 0,3904 | -6,87 | -0,1057 |
| 137,76 | 0,3413 | -6,89 | -0,1378 | 137,63 | 0,366 | -6,9 | -0,09937 | 137,57 | 0,3904 | -6,84 | -0,1058 |
| 137,73 | 0,3414 | -6,86 | -0,1378 | 137,6 | 0,366 | -6,87 | -0,09941 | 137,54 | 0,3904 | -6,81 | -0,1058 |
| 137,69 | 0,3415 | -6,83 | -0,1378 | 137,57 | 0,366 | -6,83 | -0,09945 | 137,51 | 0,3904 | -6,77 | -0,1058 |
| 137,66 | 0,3416 | -6,79 | -0,1379 | 137,53 | 0,3661 | -6,8 | -0,09948 | 137,47 | 0,3904 | -6,74 | -0,1059 |
| 137,63 | 0,3417 | -6,76 | -0,1379 | 137,5 | 0,3661 | -6,77 | -0,09952 | 137,44 | 0,3905 | -6,71 | -0,1059 |
| 137,6 | 0,3418 | -6,73 | -0,138 | 137,47 | 0,3661 | -6,73 | -0,09955 | 137,41 | 0,3905 | -6,67 | -0,1059 |
| 137,56 | 0,3418 | -6,7 | -0,138 | 137,43 | 0,3661 | -6,7 | -0,09959 | 137,37 | 0,3905 | -6,64 | -0,106 |
| 137,53 | 0,3419 | -6,66 | -0,138 | 137,4 | 0,3662 | -6,67 | -0,09962 | 137,34 | 0,3905 | -6,61 | -0,106 |
| 137,5 | 0,3419 | -6,63 | -0,1381 | 137,37 | 0,3662 | -6,63 | -0,09966 | 137,31 | 0,3906 | -6,57 | -0,106 |
| 137,46 | 0,342 | -6,59 | -0,1381 | 137,33 | 0,3662 | -6,6 | -0,0997 | 137,28 | 0,3906 | -6,54 | -0,1061 |
| 137,43 | 0,342 | -6,56 | -0,1381 | 137,3 | 0,3663 | -6,57 | -0,09973 | 137,24 | 0,3906 | -6,51 | -0,1061 |
| 137,4 | 0,3421 | -6,53 | -0,1382 | 137,27 | 0,3663 | -6,54 | -0,09977 | 137,21 | 0,3906 | -6,47 | -0,1061 |
| 137,36 | 0,3422 | -6,5 | -0,1382 | 137,23 | 0,3663 | -6,5 | -0,09981 | 137,18 | 0,3906 | -6,44 | -0,1062 |
| 137,33 | 0,3423 | -6,46 | -0,1383 | 137,2 | 0,3664 | -6,47 | -0,09986 | 137,14 | 0,3907 | -6,41 | -0,1062 |
| 137,3 | 0,3423 | -6,43 | -0,1383 | 137,17 | 0,3664 | -6,43 | -0,0999 | 137,11 | 0,3907 | -6,38 | -0,1062 |
| 137,26 | 0,3424 | -6,39 | -0,1383 | 137,13 | 0,3664 | -6,4 | -0,09994 | 137,08 | 0,3907 | -6,34 | -0,1062 |
| 137,23 | 0,3425 | -6,36 | -0,1384 | 137,1 | 0,3664 | -6,37 | -0,09997 | 137,04 | 0,3907 | -6,31 | -0,1063 |
| 137,2 | 0,3425 | -6,33 | -0,1384 | 137,07 | 0,3665 | -6,34 | -0,1 | 137,01 | 0,3907 | -6,28 | -0,1063 |
| 137,16 | 0,3426 | -6,3 | -0,1384 | 137,03 | 0,3665 | -6,3 | -0,1 | 136,98 | 0,3908 | -6,24 | -0,1063 |
| 137,13 | 0,3426 | -6,26 | -0,1385 | 137 | 0,3665 | -6,27 | -0,1001 | 136,94 | 0,3908 | -6,21 | -0,1064 |
| 137,1 | 0,3427 | -6,23 | -0,1385 | 136,97 | 0,3666 | -6,24 | -0,1001 | 136,91 | 0,3908 | -6,18 | -0,1064 |
| 137,06 | 0,3428 | -6,2 | -0,1385 | 136,93 | 0,3666 | -6,2 | -0,1002 | 136,88 | 0,3908 | -6,14 | -0,1064 |
| 137,03 | 0,3428 | -6,16 | -0,1386 | 136,9 | 0,3666 | -6,17 | -0,1002 | 136,84 | 0,3908 | -6,11 | -0,1065 |
| 137 | 0,3429 | -6,13 | -0,1386 | 136,87 | 0,3666 | -6,14 | -0,1002 | 136,81 | 0,3909 | -6,08 | -0,1065 |
| 136,97 | 0,3429 | -6,1 | -0,1386 | 136,84 | 0,3667 | -6,1 | -0,1003 | 136,78 | 0,3909 | -6,04 | -0,1065 |
| 136,93 | 0,343 | -6,06 | -0,1387 | 136,8 | 0,3667 | -6,07 | -0,1003 | 136,75 | 0,3909 | -6,01 | -0,1066 |
| 136,9 | 0,343 | -6,03 | -0,1387 | 136,77 | 0,3667 | -6,04 | -0,1003 | 136,71 | 0,3909 | -5,98 | -0,1066 |
| 136,86 | 0,3431 | -6 | -0,1387 | 136,73 | 0,3668 | -6 | -0,1004 | 136,68 | 0,3909 | -5,94 | -0,1066 |
| 136,83 | 0,3432 | -5,96 | -0,1388 | 136,7 | 0,3668 | -5,97 | -0,1004 | 136,64 | 0,391 | -5,91 | -0,1067 |
| 136,8 | 0,3433 | -5,93 | -0,1388 | 136,67 | 0,3668 | -5,94 | -0,1005 | 136,61 | 0,391 | -5,88 | -0,1067 |
| 136,77 | 0,3433 | -5,9 | -0,1389 | 136,64 | 0,3668 | -5,9 | -0,1005 | 136,58 | 0,391 | -5,84 | -0,1067 |
| 136,73 | 0,3434 | -5,86 | -0,1389 | 136,6 | 0,3669 | -5,87 | -0,1005 | 136,54 | 0,391 | -5,81 | -0,1068 |
| 136,7 | 0,3434 | -5,83 | -0,1389 | 136,57 | 0,3669 | -5,84 | -0,1006 | 136,51 | 0,391 | -5,78 | -0,1068 |
| 136,67 | 0,3435 | -5,8 | -0,139 | 136,54 | 0,3669 | -5,8 | -0,1006 | 136,48 | 0,3911 | -5,74 | -0,1068 |
| 136,63 | 0,3435 | -5,76 | -0,139 | 136,5 | 0,367 | -5,77 | -0,1006 | 136,45 | 0,3911 | -5,71 | -0,1069 |
| 136,6 | 0,3436 | -5,73 | -0,139 | 136,47 | 0,367 | -5,74 | -0,1007 | 136,41 | 0,3911 | -5,68 | -0,1069 |
| 136,57 | 0,3437 | -5,7 | -0,1391 | 136,44 | 0,367 | -5,7 | -0,1007 | 136,38 | 0,3911 | -5,64 | -0,1069 |
| 136,53 | 0,3438 | -5,66 | -0,1391 | 136,4 | 0,367 | -5,67 | -0,1008 | 136,34 | 0,3911 | -5,61 | -0,107 |
| 136,5 | 0,3439 | -5,63 | -0,1391 | 136,37 | 0,3671 | -5,64 | -0,1008 | 136,31 | 0,3911 | -5,58 | -0,107 |
| 136,47 | 0,3439 | -5,6 | -0,1392 | 136,34 | 0,3671 | -5,61 | -0,1008 | 136,28 | 0,3912 | -5,54 | -0,107 |
| 136,43 | 0,344 | -5,56 | -0,1392 | 136,3 | 0,3671 | -5,57 | -0,1009 | 136,25 | 0,3912 | -5,51 | -0,107 |
| 136,4 | 0,344 | -5,53 | -0,1392 | 136,27 | 0,3672 | -5,54 | -0,1009 | 136,21 | 0,3912 | -5,48 | -0,1071 |
| 136,37 | 0,3441 | -5,5 | -0,1393 | 136,24 | 0,3672 | -5,51 | -0,1009 | 136,18 | 0,3912 | -5,44 | -0,1071 |
| 136,33 | 0,3442 | -5,46 | -0,1393 | 136,2 | 0,3672 | -5,47 | -0,101 | 136,15 | 0,3912 | -5,41 | -0,1071 |
| 136,3 | 0,3442 | -5,43 | -0,1394 | 136,17 | 0,3672 | -5,44 | -0,101 | 136,11 | 0,3913 | -5,38 | -0,1072 |
| 136,27 | 0,3442 | -5,4 | -0,1394 | 136,14 | 0,3673 | -5,41 | -0,1011 | 136,08 | 0,3913 | -5,34 | -0,1072 |
| 136,24 | 0,3443 | -5,36 | -0,1394 | 136,1 | 0,3673 | -5,37 | -0,1011 | 136,05 | 0,3913 | -5,31 | -0,1072 |
| 136,2 | 0,3443 | -5,33 | -0,1395 | 136,07 | 0,3673 | -5,34 | -0,1011 | 136,01 | 0,3913 | -5,28 | -0,1073 |
| 136,17 | 0,3444 | -5,3 | -0,1395 | 136,04 | 0,3674 | -5,31 | -0,1012 | 135,98 | 0,3913 | -5,24 | -0,1073 |
| 136,13 | 0,3444 | -5,27 | -0,1396 | 136 | 0,3674 | -5,27 | -0,1012 | 135,95 | 0,3913 | -5,21 | -0,1073 |
| 136,1 | 0,3445 | -5,23 | -0,1396 | 135,97 | 0,3674 | -5,24 | -0,1013 | 135,91 | 0,3914 | -5,18 | -0,1074 |
| 136,07 | 0,3446 | -5,2 | -0,1396 | 135,94 | 0,3674 | -5,21 | -0,1013 | 135,88 | 0,3914 | -5,14 | -0,1074 |
| 136,04 | 0,3446 | -5,16 | -0,1397 | 135,9 | 0,3675 | -5,17 | -0,1014 | 135,85 | 0,3914 | -5,11 | -0,1074 |
| 136 | 0,3446 | -5,13 | -0,1397 | 135,87 | 0,3675 | -5,14 | -0,1014 | 135,81 | 0,3914 | -5,08 | -0,1075 |
| 135,97 | 0,3447 | -5,1 | -0,1397 | 135,84 | 0,3675 | -5,11 | -0,1014 | 135,78 | 0,3914 | -5,04 | -0,1075 |
| 135,94 | 0,3447 | -5,07 | -0,1398 | 135,8 | 0,3675 | -5,07 | -0,1015 | 135,75 | 0,3914 | -5,01 | -0,1075 |
| 135,9 | 0,3448 | -5,03 | -0,1398 | 135,77 | 0,3676 | -5,04 | -0,1015 | 135,71 | 0,3915 | -4,98 | -0,1076 |
| 135,87 | 0,3448 | -5 | -0,1398 | 135,74 | 0,3676 | -5,01 | -0,1016 | 135,68 | 0,3915 | -4,94 | -0,1076 |
| 135,84 | 0,3448 | -4,97 | -0,1399 | 135,7 | 0,3676 | -4,97 | -0,1016 | 135,65 | 0,3915 | -4,91 | -0,1076 |
| 135,8 | 0,3449 | -4,93 | -0,1399 | 135,67 | 0,3677 | -4,94 | -0,1016 | 135,62 | 0,3915 | -4,88 | -0,1076 |
| 135,77 | 0,3449 | -4,9 | -0,14 | 135,64 | 0,3677 | -4,91 | -0,1017 | 135,58 | 0,3915 | -4,85 | -0,1077 |
| 135,74 | 0,345 | -4,87 | -0,14 | 135,6 | 0,3677 | -4,87 | -0,1017 | 135,55 | 0,3915 | -4,81 | -0,1077 |
| 135,7 | 0,3451 | -4,83 | -0,14 | 135,57 | 0,3677 | -4,84 | -0,1017 | 135,51 | 0,3916 | -4,78 | -0,1077 |
| 135,67 | 0,3451 | -4,8 | -0,1401 | 135,54 | 0,3678 | -4,81 | -0,1018 | 135,48 | 0,3916 | -4,74 | -0,1078 |
| 135,64 | 0,3452 | -4,77 | -0,1401 | 135,5 | 0,3678 | -4,77 | -0,1018 | 135,45 | 0,3916 | -4,71 | -0,1078 |
| 135,6 | 0,3452 | -4,73 | -0,1401 | 135,47 | 0,3678 | -4,74 | -0,1019 | 135,41 | 0,3916 | -4,68 | -0,1078 |
| 135,57 | 0,3453 | -4,7 | -0,1402 | 135,44 | 0,3678 | -4,71 | -0,102 | 135,38 | 0,3916 | -4,64 | -0,1079 |
| 135,54 | 0,3453 | -4,67 | -0,1402 | 135,41 | 0,3679 | -4,67 | -0,102 | 135,35 | 0,3916 | -4,61 | -0,1079 |
| 135,5 | 0,3454 | -4,63 | -0,1403 | 135,37 | 0,3679 | -4,64 | -0,1021 | 135,31 | 0,3917 | -4,58 | -0,1079 |
| 135,47 | 0,3454 | -4,6 | -0,1403 | 135,34 | 0,3679 | -4,61 | -0,1021 | 135,28 | 0,3917 | -4,54 | -0,1079 |
| 135,44 | 0,3454 | -4,57 | -0,1403 | 135,3 | 0,3679 | -4,57 | -0,1022 | 135,25 | 0,3917 | -4,51 | -0,108 |
| 135,4 | 0,3455 | -4,53 | -0,1404 | 135,27 | 0,368 | -4,54 | -0,1022 | 135,21 | 0,3917 | -4,48 | -0,108 |
| 135,37 | 0,3456 | -4,5 | -0,1404 | 135,24 | 0,368 | -4,51 | -0,1022 | 135,18 | 0,3917 | -4,44 | -0,108 |
| 135,34 | 0,3456 | -4,47 | -0,1405 | 135,2 | 0,368 | -4,47 | -0,1023 | 135,15 | 0,3917 | -4,41 | -0,1081 |
| 135,31 | 0,3457 | -4,43 | -0,1405 | 135,17 | 0,368 | -4,44 | -0,1023 | 135,12 | 0,3917 | -4,38 | -0,1081 |
| 135,27 | 0,3457 | -4,4 | -0,1405 | 135,14 | 0,3681 | -4,41 | -0,1024 | 135,08 | 0,3918 | -4,34 | -0,1081 |
| 135,24 | 0,3457 | -4,37 | -0,1406 | 135,11 | 0,3681 | -4,37 | -0,1024 | 135,05 | 0,3918 | -4,31 | -0,1082 |
| 135,2 | 0,3458 | -4,33 | -0,1406 | 135,07 | 0,3681 | -4,34 | -0,1024 | 135,01 | 0,3918 | -4,28 | -0,1082 |
| 135,17 | 0,3458 | -4,3 | -0,1406 | 135,04 | 0,3681 | -4,31 | -0,1025 | 134,98 | 0,3918 | -4,24 | -0,1082 |
| 135,14 | 0,3459 | -4,27 | -0,1407 | 135,01 | 0,3682 | -4,27 | -0,1025 | 134,95 | 0,3918 | -4,21 | -0,1083 |
| 135,11 | 0,346 | -4,23 | -0,1407 | 134,97 | 0,3682 | -4,24 | -0,1025 | 134,92 | 0,3918 | -4,18 | -0,1083 |
| 135,07 | 0,3461 | -4,2 | -0,1408 | 134,94 | 0,3682 | -4,21 | -0,1026 | 134,88 | 0,3918 | -4,15 | -0,1083 |
| 135,04 | 0,3461 | -4,17 | -0,1408 | 134,91 | 0,3682 | -4,17 | -0,1026 | 134,85 | 0,3918 | -4,11 | -0,1083 |
| 135,01 | 0,3462 | -4,14 | -0,1408 | 134,87 | 0,3683 | -4,14 | -0,1027 | 134,82 | 0,3918 | -4,08 | -0,1084 |
| 134,97 | 0,3462 | -4,1 | -0,1409 | 134,84 | 0,3683 | -4,11 | -0,1027 | 134,78 | 0,3919 | -4,05 | -0,1084 |
| 134,94 | 0,3463 | -4,07 | -0,1409 | 134,81 | 0,3683 | -4,08 | -0,1028 | 134,75 | 0,3919 | -4,01 | -0,1084 |
| 134,91 | 0,3463 | -4,03 | -0,1409 | 134,77 | 0,3683 | -4,04 | -0,1028 | 134,72 | 0,3919 | -3,98 | -0,1085 |
| 134,87 | 0,3464 | -4 | -0,141 | 134,74 | 0,3684 | -4,01 | -0,1028 | 134,68 | 0,3919 | -3,95 | -0,1085 |
| 134,84 | 0,3464 | -3,97 | -0,141 | 134,71 | 0,3684 | -3,97 | -0,1029 | 134,65 | 0,3919 | -3,91 | -0,1085 |
| 134,81 | 0,3464 | -3,93 | -0,1411 | 134,67 | 0,3684 | -3,94 | -0,1029 | 134,62 | 0,3919 | -3,88 | -0,1085 |
| 134,77 | 0,3465 | -3,9 | -0,1411 | 134,64 | 0,3684 | -3,91 | -0,103 | 134,58 | 0,3919 | -3,85 | -0,1086 |
| 134,74 | 0,3465 | -3,87 | -0,1411 | 134,61 | 0,3685 | -3,88 | -0,103 | 134,55 | 0,392 | -3,81 | -0,1086 |
| 134,71 | 0,3466 | -3,83 | -0,1412 | 134,57 | 0,3685 | -3,84 | -0,103 | 134,52 | 0,392 | -3,78 | -0,1086 |
| 134,67 | 0,3466 | -3,8 | -0,1412 | 134,54 | 0,3685 | -3,81 | -0,1031 | 134,48 | 0,392 | -3,75 | -0,1087 |
| 134,64 | 0,3466 | -3,77 | -0,1412 | 134,51 | 0,3685 | -3,77 | -0,1031 | 134,45 | 0,392 | -3,71 | -0,1087 |
| 134,61 | 0,3467 | -3,73 | -0,1413 | 134,47 | 0,3686 | -3,74 | -0,1032 | 134,42 | 0,392 | -3,68 | -0,1087 |
| 134,58 | 0,3467 | -3,7 | -0,1413 | 134,44 | 0,3686 | -3,71 | -0,1032 | 134,38 | 0,392 | -3,65 | -0,1088 |
| 134,54 | 0,3468 | -3,67 | -0,1414 | 134,41 | 0,3686 | -3,68 | -0,1032 | 134,35 | 0,392 | -3,61 | -0,1088 |
| 134,51 | 0,3468 | -3,64 | -0,1414 | 134,37 | 0,3686 | -3,64 | -0,1033 | 134,32 | 0,392 | -3,58 | -0,1088 |
| 134,48 | 0,3468 | -3,6 | -0,1414 | 134,34 | 0,3686 | -3,61 | -0,1033 | 134,28 | 0,392 | -3,55 | -0,1088 |
| 134,44 | 0,3469 | -3,57 | -0,1415 | 134,31 | 0,3687 | -3,58 | -0,1034 | 134,25 | 0,3921 | -3,51 | -0,1089 |
| 134,41 | 0,3469 | -3,54 | -0,1415 | 134,27 | 0,3687 | -3,54 | -0,1034 | 134,22 | 0,3921 | -3,48 | -0,1089 |
| 134,38 | 0,347 | -3,5 | -0,1416 | 134,24 | 0,3687 | -3,51 | -0,1035 | 134,18 | 0,3921 | -3,45 | -0,1089 |
| 134,34 | 0,347 | -3,47 | -0,1416 | 134,21 | 0,3687 | -3,48 | -0,1035 | 134,15 | 0,3921 | -3,41 | -0,109 |
| 134,31 | 0,3471 | -3,44 | -0,1416 | 134,17 | 0,3688 | -3,44 | -0,1035 | 134,12 | 0,3921 | -3,38 | -0,109 |
| 134,28 | 0,3472 | -3,4 | -0,1417 | 134,14 | 0,3688 | -3,41 | -0,1036 | 134,08 | 0,3921 | -3,35 | -0,109 |
| 134,24 | 0,3472 | -3,37 | -0,1417 | 134,11 | 0,3688 | -3,38 | -0,1036 | 134,05 | 0,3921 | -3,31 | -0,1091 |
| 134,21 | 0,3473 | -3,34 | -0,1418 | 134,07 | 0,3688 | -3,34 | -0,1037 | 134,02 | 0,3921 | -3,28 | -0,1091 |
| 134,18 | 0,3473 | -3,3 | -0,1418 | 134,04 | 0,3688 | -3,31 | -0,1037 | 133,98 | 0,3921 | -3,25 | -0,1091 |
| 134,14 | 0,3474 | -3,27 | -0,1418 | 134,01 | 0,3689 | -3,28 | -0,1038 | 133,95 | 0,3921 | -3,21 | -0,1091 |
| 134,11 | 0,3474 | -3,24 | -0,1419 | 133,97 | 0,3689 | -3,24 | -0,1038 | 133,92 | 0,3922 | -3,18 | -0,1092 |
| 134,08 | 0,3475 | -3,2 | -0,1419 | 133,94 | 0,3689 | -3,21 | -0,1038 | 133,88 | 0,3922 | -3,15 | -0,1092 |
| 134,04 | 0,3475 | -3,17 | -0,1419 | 133,91 | 0,3689 | -3,18 | -0,1039 | 133,85 | 0,3922 | -3,11 | -0,1092 |
| 134,01 | 0,3476 | -3,14 | -0,142 | 133,87 | 0,369 | -3,14 | -0,1039 | 133,82 | 0,3922 | -3,08 | -0,1093 |
| 133,98 | 0,3476 | -3,1 | -0,142 | 133,84 | 0,369 | -3,11 | -0,104 | 133,78 | 0,3922 | -3,05 | -0,1093 |
| 133,94 | 0,3476 | -3,07 | -0,1421 | 133,81 | 0,369 | -3,08 | -0,104 | 133,75 | 0,3922 | -3,01 | -0,1093 |
| 133,91 | 0,3477 | -3,04 | -0,1421 | 133,77 | 0,369 | -3,04 | -0,104 | 133,72 | 0,3922 | -2,98 | -0,1093 |
| 133,88 | 0,3477 | -3 | -0,1421 | 133,74 | 0,369 | -3,01 | -0,1041 | 133,68 | 0,3922 | -2,95 | -0,1094 |
| 133,84 | 0,3478 | -2,97 | -0,1422 | 133,71 | 0,3691 | -2,98 | -0,1041 | 133,65 | 0,3922 | -2,91 | -0,1094 |
| 133,81 | 0,3478 | -2,94 | -0,1422 | 133,67 | 0,3691 | -2,94 | -0,1042 | 133,62 | 0,3922 | -2,88 | -0,1094 |
| 133,78 | 0,3479 | -2,9 | -0,1423 | 133,64 | 0,3691 | -2,91 | -0,1042 | 133,58 | 0,3923 | -2,85 | -0,1095 |
| 133,74 | 0,3479 | -2,87 | -0,1423 | 133,61 | 0,3691 | -2,88 | -0,1042 | 133,55 | 0,3923 | -2,81 | -0,1095 |
| 133,71 | 0,3479 | -2,84 | -0,1423 | 133,58 | 0,3691 | -2,84 | -0,1043 | 133,52 | 0,3923 | -2,78 | -0,1095 |
| 133,68 | 0,348 | -2,8 | -0,1424 | 133,54 | 0,3692 | -2,81 | -0,1043 | 133,48 | 0,3923 | -2,75 | -0,1095 |
| 133,65 | 0,348 | -2,77 | -0,1424 | 133,51 | 0,3692 | -2,78 | -0,1044 | 133,45 | 0,3923 | -2,71 | -0,1096 |
| 133,61 | 0,3481 | -2,74 | -0,1425 | 133,47 | 0,3692 | -2,74 | -0,1044 | 133,42 | 0,3923 | -2,68 | -0,1096 |
| 133,58 | 0,3481 | -2,7 | -0,1425 | 133,44 | 0,3692 | -2,71 | -0,1045 | 133,38 | 0,3923 | -2,65 | -0,1096 |
| 133,55 | 0,3481 | -2,67 | -0,1426 | 133,41 | 0,3692 | -2,68 | -0,1045 | 133,35 | 0,3923 | -2,61 | -0,1097 |
| 133,51 | 0,3482 | -2,64 | -0,1426 | 133,37 | 0,3693 | -2,64 | -0,1045 | 133,32 | 0,3923 | -2,58 | -0,1097 |
| 133,48 | 0,3482 | -2,6 | -0,1426 | 133,34 | 0,3693 | -2,61 | -0,1046 | 133,28 | 0,3923 | -2,55 | -0,1097 |
| 133,44 | 0,3483 | -2,57 | -0,1427 | 133,31 | 0,3693 | -2,58 | -0,1046 | 133,25 | 0,3923 | -2,51 | -0,1097 |
| 133,41 | 0,3484 | -2,54 | -0,1427 | 133,28 | 0,3693 | -2,54 | -0,1047 | 133,22 | 0,3923 | -2,48 | -0,1098 |
| 133,38 | 0,3484 | -2,5 | -0,1427 | 133,24 | 0,3693 | -2,51 | -0,1047 | 133,18 | 0,3923 | -2,45 | -0,1098 |
| 133,35 | 0,3485 | -2,47 | -0,1428 | 133,21 | 0,3693 | -2,48 | -0,1048 | 133,15 | 0,3923 | -2,41 | -0,1098 |
| 133,31 | 0,3485 | -2,44 | -0,1428 | 133,17 | 0,3694 | -2,44 | -0,1048 | 133,12 | 0,3923 | -2,38 | -0,1099 |
| 133,28 | 0,3485 | -2,4 | -0,1429 | 133,14 | 0,3694 | -2,41 | -0,1048 | 133,08 | 0,3923 | -2,35 | -0,1099 |
| 133,25 | 0,3486 | -2,37 | -0,1429 | 133,11 | 0,3694 | -2,38 | -0,1049 | 133,05 | 0,3924 | -2,31 | -0,1099 |
| 133,21 | 0,3486 | -2,34 | -0,1429 | 133,08 | 0,3694 | -2,34 | -0,1049 | 133,02 | 0,3924 | -2,28 | -0,1099 |
| 133,18 | 0,3487 | -2,3 | -0,143 | 133,04 | 0,3694 | -2,31 | -0,105 | 132,98 | 0,3924 | -2,25 | -0,11 |
| 133,15 | 0,3487 | -2,27 | -0,143 | 133,01 | 0,3695 | -2,28 | -0,105 | 132,95 | 0,3924 | -2,21 | -0,11 |
| 133,11 | 0,3488 | -2,24 | -0,143 | 132,98 | 0,3695 | -2,24 | -0,105 | 132,92 | 0,3924 | -2,18 | -0,11 |
| 133,08 | 0,3488 | -2,2 | -0,1431 | 132,94 | 0,3695 | -2,21 | -0,1051 | 132,89 | 0,3924 | -2,15 | -0,1101 |
| 133,05 | 0,3489 | -2,17 | -0,1432 | 132,91 | 0,3695 | -2,18 | -0,1051 | 132,85 | 0,3924 | -2,11 | -0,1101 |
| 133,01 | 0,3489 | -2,14 | -0,1432 | 132,88 | 0,3695 | -2,15 | -0,1052 | 132,82 | 0,3924 | -2,08 | -0,1101 |
| 132,98 | 0,3489 | -2,1 | -0,1432 | 132,84 | 0,3695 | -2,11 | -0,1052 | 132,79 | 0,3924 | -2,05 | -0,1101 |
| 132,95 | 0,349 | -2,07 | -0,1433 | 132,81 | 0,3696 | -2,08 | -0,1053 | 132,75 | 0,3924 | -2,01 | -0,1102 |
| 132,91 | 0,3491 | -2,04 | -0,1433 | 132,78 | 0,3696 | -2,04 | -0,1053 | 132,72 | 0,3924 | -1,98 | -0,1102 |
| 132,88 | 0,3491 | -2 | -0,1434 | 132,74 | 0,3696 | -2,01 | -0,1053 | 132,69 | 0,3924 | -1,95 | -0,1102 |
| 132,85 | 0,3492 | -1,97 | -0,1434 | 132,71 | 0,3696 | -1,98 | -0,1054 | 132,65 | 0,3924 | -1,91 | -0,1103 |
| 132,81 | 0,3492 | -1,94 | -0,1435 | 132,68 | 0,3696 | -1,94 | -0,1054 | 132,62 | 0,3924 | -1,88 | -0,1103 |
| 132,78 | 0,3492 | -1,9 | -0,1435 | 132,64 | 0,3696 | -1,91 | -0,1055 | 132,59 | 0,3924 | -1,85 | -0,1103 |
| 132,75 | 0,3493 | -1,87 | -0,1435 | 132,61 | 0,3697 | -1,88 | -0,1055 | 132,55 | 0,3924 | -1,81 | -0,1103 |
| 132,71 | 0,3493 | -1,84 | -0,1436 | 132,58 | 0,3697 | -1,85 | -0,1056 | 132,52 | 0,3925 | -1,78 | -0,1104 |
| 132,68 | 0,3494 | -1,8 | -0,1436 | 132,54 | 0,3697 | -1,81 | -0,1056 | 132,49 | 0,3925 | -1,75 | -0,1104 |
| 132,65 | 0,3494 | -1,77 | -0,1436 | 132,51 | 0,3697 | -1,78 | -0,1056 | 132,45 | 0,3925 | -1,71 | -0,1104 |
| 132,61 | 0,3494 | -1,74 | -0,1437 | 132,48 | 0,3697 | -1,75 | -0,1057 | 132,42 | 0,3925 | -1,68 | -0,1105 |
| 132,58 | 0,3495 | -1,71 | -0,1437 | 132,44 | 0,3697 | -1,71 | -0,1057 | 132,39 | 0,3925 | -1,65 | -0,1105 |
| 132,55 | 0,3495 | -1,67 | -0,1438 | 132,41 | 0,3698 | -1,68 | -0,1058 | 132,35 | 0,3925 | -1,61 | -0,1105 |
| 132,51 | 0,3496 | -1,64 | -0,1438 | 132,38 | 0,3698 | -1,65 | -0,1058 | 132,32 | 0,3925 | -1,58 | -0,1105 |
| 132,48 | 0,3496 | -1,61 | -0,1438 | 132,34 | 0,3698 | -1,61 | -0,1059 | 132,29 | 0,3925 | -1,55 | -0,1106 |
| 132,45 | 0,3497 | -1,57 | -0,1439 | 132,31 | 0,3698 | -1,58 | -0,1059 | 132,25 | 0,3925 | -1,51 | -0,1106 |
| 132,41 | 0,3497 | -1,54 | -0,1439 | 132,28 | 0,3698 | -1,55 | -0,1059 | 132,22 | 0,3925 | -1,48 | -0,1106 |
| 132,38 | 0,3498 | -1,51 | -0,144 | 132,24 | 0,3698 | -1,51 | -0,106 | 132,19 | 0,3925 | -1,45 | -0,1106 |
| 132,35 | 0,3498 | -1,47 | -0,144 | 132,21 | 0,3699 | -1,48 | -0,106 | 132,15 | 0,3925 | -1,41 | -0,1107 |
| 132,32 | 0,3499 | -1,44 | -0,144 | 132,18 | 0,3699 | -1,45 | -0,1061 | 132,12 | 0,3925 | -1,38 | -0,1107 |
| 132,28 | 0,3499 | -1,41 | -0,1441 | 132,14 | 0,3699 | -1,41 | -0,1061 | 132,09 | 0,3925 | -1,35 | -0,1107 |
| 132,25 | 0,3499 | -1,37 | -0,1441 | 132,11 | 0,3699 | -1,38 | -0,1062 | 132,05 | 0,3925 | -1,31 | -0,1108 |
| 132,22 | 0,35 | -1,34 | -0,1442 | 132,08 | 0,3699 | -1,35 | -0,1062 | 132,02 | 0,3925 | -1,28 | -0,1108 |
| 132,18 | 0,35 | -1,31 | -0,1442 | 132,04 | 0,3699 | -1,31 | -0,1062 | 131,99 | 0,3925 | -1,25 | -0,1108 |
| 132,15 | 0,3501 | -1,27 | -0,1443 | 132,01 | 0,3699 | -1,28 | -0,1063 | 131,95 | 0,3925 | -1,21 | -0,1108 |
| 132,12 | 0,3501 | -1,24 | -0,1443 | 131,98 | 0,3699 | -1,25 | -0,1063 | 131,92 | 0,3925 | -1,18 | -0,1109 |
| 132,08 | 0,3501 | -1,21 | -0,1443 | 131,94 | 0,37 | -1,21 | -0,1064 | 131,89 | 0,3925 | -1,15 | -0,1109 |
| 132,05 | 0,3502 | -1,17 | -0,1444 | 131,91 | 0,37 | -1,18 | -0,1064 | 131,85 | 0,3925 | -1,11 | -0,1109 |
| 132,02 | 0,3502 | -1,14 | -0,1444 | 131,88 | 0,37 | -1,15 | -0,1065 | 131,82 | 0,3925 | -1,08 | -0,1109 |
| 131,98 | 0,3503 | -1,11 | -0,1445 | 131,84 | 0,37 | -1,11 | -0,1065 | 131,79 | 0,3925 | -1,05 | -0,111 |
| 131,95 | 0,3503 | -1,07 | -0,1445 | 131,81 | 0,37 | -1,08 | -0,1065 | 131,75 | 0,3925 | -1,01 | -0,111 |
| 131,91 | 0,3504 | -1,04 | -0,1445 | 131,78 | 0,37 | -1,05 | -0,1066 | 131,72 | 0,3925 | -0,98 | -0,111 |
| 131,88 | 0,3504 | -1,01 | -0,1446 | 131,74 | 0,37 | -1,01 | -0,1066 | 131,69 | 0,3925 | -0,95 | -0,1111 |
| 131,85 | 0,3504 | -0,97 | -0,1446 | 131,71 | 0,3701 | -0,98 | -0,1067 | 131,65 | 0,3925 | -0,91 | -0,1111 |
| 131,82 | 0,3505 | -0,94 | -0,1447 | 131,68 | 0,3701 | -0,95 | -0,1067 | 131,62 | 0,3925 | -0,88 | -0,1111 |
| 131,78 | 0,3505 | -0,91 | -0,1447 | 131,64 | 0,3701 | -0,91 | -0,1068 | 131,59 | 0,3925 | -0,85 | -0,1112 |
| 131,75 | 0,3506 | -0,87 | -0,1447 | 131,61 | 0,3701 | -0,88 | -0,1068 | 131,55 | 0,3925 | -0,81 | -0,1112 |
| 131,72 | 0,3506 | -0,84 | -0,1448 | 131,58 | 0,3701 | -0,85 | -0,1069 | 131,52 | 0,3925 | -0,78 | -0,1112 |
| 131,68 | 0,3507 | -0,81 | -0,1448 | 131,54 | 0,3701 | -0,81 | -0,1069 | 131,49 | 0,3926 | -0,75 | -0,1112 |
| 131,65 | 0,3507 | -0,77 | -0,1449 | 131,51 | 0,3701 | -0,78 | -0,1069 | 131,45 | 0,3926 | -0,72 | -0,1113 |
| 131,62 | 0,3507 | -0,74 | -0,1449 | 131,48 | 0,3701 | -0,75 | -0,107 | 131,42 | 0,3926 | -0,68 | -0,1113 |
| 131,58 | 0,3508 | -0,71 | -0,1449 | 131,44 | 0,3702 | -0,71 | -0,107 | 131,39 | 0,3926 | -0,65 | -0,1113 |
| 131,55 | 0,3508 | -0,67 | -0,145 | 131,41 | 0,3702 | -0,68 | -0,1071 | 131,35 | 0,3926 | -0,61 | -0,1114 |
| 131,52 | 0,3509 | -0,64 | -0,145 | 131,38 | 0,3702 | -0,65 | -0,1071 | 131,32 | 0,3926 | -0,58 | -0,1114 |
| 131,48 | 0,3509 | -0,61 | -0,1451 | 131,34 | 0,3702 | -0,61 | -0,1071 | 131,29 | 0,3926 | -0,55 | -0,1114 |
| 131,45 | 0,351 | -0,57 | -0,1451 | 131,31 | 0,3702 | -0,58 | -0,1072 | 131,25 | 0,3926 | -0,51 | -0,1114 |
| 131,42 | 0,351 | -0,54 | -0,1451 | 131,28 | 0,3702 | -0,55 | -0,1072 | 131,22 | 0,3926 | -0,48 | -0,1115 |
| 131,38 | 0,3511 | -0,51 | -0,1452 | 131,24 | 0,3702 | -0,51 | -0,1073 | 131,19 | 0,3926 | -0,45 | -0,1115 |
| 131,35 | 0,3511 | -0,47 | -0,1452 | 131,21 | 0,3702 | -0,48 | -0,1073 | 131,16 | 0,3926 | -0,41 | -0,1115 |
| 131,32 | 0,3511 | -0,44 | -0,1453 | 131,18 | 0,3702 | -0,45 | -0,1074 | 131,12 | 0,3926 | -0,38 | -0,1115 |
| 131,28 | 0,3512 | -0,41 | -0,1453 | 131,14 | 0,3703 | -0,41 | -0,1074 | 131,09 | 0,3926 | -0,35 | -0,1116 |
| 131,25 | 0,3512 | -0,37 | -0,1453 | 131,11 | 0,3703 | -0,38 | -0,1074 | 131,05 | 0,3926 | -0,32 | -0,1116 |
| 131,22 | 0,3513 | -0,34 | -0,1454 | 131,08 | 0,3703 | -0,35 | -0,1075 | 131,02 | 0,3926 | -0,28 | -0,1116 |
| 131,19 | 0,3513 | -0,31 | -0,1454 | 131,04 | 0,3703 | -0,31 | -0,1075 | 130,99 | 0,3926 | -0,25 | -0,1116 |
| 131,15 | 0,3514 | -0,27 | -0,1455 | 131,01 | 0,3703 | -0,28 | -0,1076 | 130,95 | 0,3926 | -0,21 | -0,1117 |
| 131,12 | 0,3514 | -0,24 | -0,1455 | 130,98 | 0,3703 | -0,25 | -0,1076 | 130,92 | 0,3926 | -0,18 | -0,1117 |
| 131,09 | 0,3514 | -0,21 | -0,1456 | 130,95 | 0,3703 | -0,22 | -0,1077 | 130,89 | 0,3926 | -0,15 | -0,1117 |
| 131,05 | 0,3515 | -0,17 | -0,1456 | 130,91 | 0,3703 | -0,18 | -0,1077 | 130,85 | 0,3926 | -0,12 | -0,1118 |
| 131,02 | 0,3515 | -0,14 | -0,1456 | 130,88 | 0,3704 | -0,15 | -0,1078 | 130,82 | 0,3926 | -0,08 | -0,1118 |
| 130,99 | 0,3516 | -0,11 | -0,1457 | 130,84 | 0,3704 | -0,11 | -0,1078 | 130,79 | 0,3926 | -0,05 | -0,1118 |
| 130,95 | 0,3516 | -0,07 | -0,1457 | 130,81 | 0,3704 | -0,08 | -0,1078 | 130,75 | 0,3926 | -0,01 | -0,1118 |
| 130,92 | 0,3517 | -0,04 | -0,1458 | 130,78 | 0,3704 | -0,05 | -0,1079 | 130,72 | 0,3926 | 0,02 | -0,1119 |
| 130,89 | 0,3518 | -0,01 | -0,1458 | 130,74 | 0,3704 | -0,01 | -0,1079 | 130,69 | 0,3926 | 0,05 | -0,1119 |
| 130,85 | 0,3518 | 0,03 | -0,1458 | 130,71 | 0,3704 | 0,02 | -0,108 | 130,65 | 0,3926 | 0,09 | -0,1119 |
| 130,82 | 0,3519 | 0,06 | -0,1459 | 130,68 | 0,3704 | 0,05 | -0,108 | 130,62 | 0,3926 | 0,12 | -0,112 |
| 130,79 | 0,3519 | 0,09 | -0,1459 | 130,64 | 0,3704 | 0,08 | -0,1081 | 130,59 | 0,3926 | 0,15 | -0,112 |
| 130,75 | 0,3519 | 0,13 | -0,146 | 130,61 | 0,3704 | 0,12 | -0,1081 | 130,55 | 0,3926 | 0,19 | -0,112 |
| 130,72 | 0,352 | 0,16 | -0,146 | 130,58 | 0,3704 | 0,15 | -0,1081 | 130,52 | 0,3926 | 0,22 | -0,112 |
| 130,69 | 0,352 | 0,19 | -0,1461 | 130,55 | 0,3705 | 0,19 | -0,1082 | 130,49 | 0,3926 | 0,25 | -0,1121 |
| 130,65 | 0,3521 | 0,23 | -0,1461 | 130,51 | 0,3705 | 0,22 | -0,1082 | 130,46 | 0,3926 | 0,29 | -0,1121 |
| 130,62 | 0,3521 | 0,26 | -0,1461 | 130,48 | 0,3705 | 0,25 | -0,1083 | 130,42 | 0,3926 | 0,32 | -0,1121 |
| 130,59 | 0,3522 | 0,29 | -0,1462 | 130,45 | 0,3705 | 0,28 | -0,1083 | 130,39 | 0,3926 | 0,35 | -0,1121 |
| 130,55 | 0,3522 | 0,33 | -0,1462 | 130,41 | 0,3705 | 0,32 | -0,1084 | 130,35 | 0,3926 | 0,38 | -0,1122 |
| 130,52 | 0,3522 | 0,36 | -0,1463 | 130,38 | 0,3705 | 0,35 | -0,1084 | 130,32 | 0,3926 | 0,42 | -0,1122 |
| 130,49 | 0,3523 | 0,39 | -0,1463 | 130,35 | 0,3705 | 0,39 | -0,1084 | 130,29 | 0,3926 | 0,45 | -0,1122 |
| 130,45 | 0,3523 | 0,43 | -0,1463 | 130,31 | 0,3705 | 0,42 | -0,1085 | 130,26 | 0,3926 | 0,49 | -0,1122 |
| 130,42 | 0,3524 | 0,46 | -0,1464 | 130,28 | 0,3705 | 0,45 | -0,1085 | 130,22 | 0,3925 | 0,52 | -0,1123 |
| 130,39 | 0,3524 | 0,49 | -0,1464 | 130,25 | 0,3705 | 0,48 | -0,1086 | 130,19 | 0,3925 | 0,55 | -0,1123 |
| 130,35 | 0,3525 | 0,52 | -0,1465 | 130,21 | 0,3706 | 0,52 | -0,1086 | 130,16 | 0,3925 | 0,58 | -0,1123 |
| 130,32 | 0,3525 | 0,56 | -0,1465 | 130,18 | 0,3706 | 0,55 | -0,1087 | 130,12 | 0,3925 | 0,62 | -0,1123 |
| 130,29 | 0,3525 | 0,59 | -0,1465 | 130,15 | 0,3706 | 0,58 | -0,1087 | 130,09 | 0,3925 | 0,65 | -0,1124 |
| 130,25 | 0,3526 | 0,63 | -0,1466 | 130,11 | 0,3706 | 0,62 | -0,1087 | 130,06 | 0,3925 | 0,69 | -0,1124 |
| 130,22 | 0,3526 | 0,66 | -0,1466 | 130,08 | 0,3706 | 0,65 | -0,1088 | 130,02 | 0,3925 | 0,72 | -0,1124 |
| 130,19 | 0,3527 | 0,69 | -0,1467 | 130,05 | 0,3706 | 0,68 | -0,1088 | 129,99 | 0,3925 | 0,75 | -0,1125 |
| 130,15 | 0,3527 | 0,73 | -0,1467 | 130,01 | 0,3706 | 0,72 | -0,1089 | 129,96 | 0,3925 | 0,79 | -0,1125 |
| 130,12 | 0,3527 | 0,76 | -0,1468 | 129,98 | 0,3706 | 0,75 | -0,1089 | 129,92 | 0,3925 | 0,82 | -0,1125 |
| 130,09 | 0,3528 | 0,79 | -0,1468 | 129,95 | 0,3706 | 0,78 | -0,109 | 129,89 | 0,3925 | 0,85 | -0,1125 |
| 130,05 | 0,3528 | 0,82 | -0,1468 | 129,91 | 0,3706 | 0,82 | -0,109 | 129,86 | 0,3925 | 0,89 | -0,1126 |
| 130,02 | 0,3529 | 0,86 | -0,1469 | 129,88 | 0,3706 | 0,85 | -0,1091 | 129,82 | 0,3925 | 0,92 | -0,1126 |
| 129,99 | 0,3529 | 0,89 | -0,1469 | 129,85 | 0,3706 | 0,88 | -0,1091 | 129,79 | 0,3925 | 0,95 | -0,1126 |
| 129,95 | 0,353 | 0,92 | -0,147 | 129,81 | 0,3707 | 0,92 | -0,1091 | 129,76 | 0,3925 | 0,98 | -0,1126 |
| 129,92 | 0,353 | 0,96 | -0,147 | 129,78 | 0,3707 | 0,95 | -0,1092 | 129,72 | 0,3925 | 1,02 | -0,1127 |
| 129,89 | 0,353 | 0,99 | -0,147 | 129,75 | 0,3707 | 0,98 | -0,1092 | 129,69 | 0,3925 | 1,05 | -0,1127 |
| 129,86 | 0,3531 | 1,02 | -0,1471 | 129,71 | 0,3707 | 1,02 | -0,1093 | 129,66 | 0,3925 | 1,08 | -0,1127 |
| 129,82 | 0,3531 | 1,06 | -0,1471 | 129,68 | 0,3707 | 1,05 | -0,1093 | 129,62 | 0,3925 | 1,12 | -0,1128 |
| 129,79 | 0,3532 | 1,09 | -0,1472 | 129,65 | 0,3707 | 1,08 | -0,1094 | 129,59 | 0,3925 | 1,15 | -0,1128 |
| 129,75 | 0,3532 | 1,12 | -0,1472 | 129,61 | 0,3707 | 1,12 | -0,1094 | 129,56 | 0,3925 | 1,19 | -0,1128 |
| 129,72 | 0,3533 | 1,16 | -0,1472 | 129,58 | 0,3707 | 1,15 | -0,1095 | 129,52 | 0,3925 | 1,22 | -0,1128 |
| 129,69 | 0,3533 | 1,19 | -0,1473 | 129,55 | 0,3707 | 1,18 | -0,1095 | 129,49 | 0,3925 | 1,25 | -0,1129 |
| 129,66 | 0,3533 | 1,22 | -0,1473 | 129,51 | 0,3707 | 1,22 | -0,1095 | 129,46 | 0,3925 | 1,28 | -0,1129 |
| 129,62 | 0,3534 | 1,26 | -0,1474 | 129,48 | 0,3707 | 1,25 | -0,1096 | 129,42 | 0,3925 | 1,32 | -0,1129 |
| 129,59 | 0,3534 | 1,29 | -0,1474 | 129,45 | 0,3707 | 1,28 | -0,1096 | 129,39 | 0,3925 | 1,35 | -0,1129 |
| 129,56 | 0,3535 | 1,32 | -0,1475 | 129,41 | 0,3707 | 1,32 | -0,1097 | 129,36 | 0,3925 | 1,38 | -0,113 |
| 129,52 | 0,3535 | 1,36 | -0,1475 | 129,38 | 0,3707 | 1,35 | -0,1097 | 129,32 | 0,3925 | 1,42 | -0,113 |
| 129,49 | 0,3535 | 1,39 | -0,1475 | 129,35 | 0,3708 | 1,38 | -0,1098 | 129,29 | 0,3925 | 1,45 | -0,113 |
| 129,46 | 0,3536 | 1,42 | -0,1476 | 129,31 | 0,3708 | 1,42 | -0,1098 | 129,26 | 0,3925 | 1,48 | -0,113 |
| 129,42 | 0,3536 | 1,46 | -0,1476 | 129,28 | 0,3708 | 1,45 | -0,1099 | 129,22 | 0,3925 | 1,52 | -0,1131 |
| 129,39 | 0,3537 | 1,49 | -0,1477 | 129,25 | 0,3708 | 1,48 | -0,1099 | 129,19 | 0,3925 | 1,55 | -0,1131 |
| 129,36 | 0,3537 | 1,52 | -0,1477 | 129,21 | 0,3708 | 1,52 | -0,1099 | 129,16 | 0,3925 | 1,59 | -0,1131 |
| 129,32 | 0,3538 | 1,56 | -0,1477 | 129,18 | 0,3708 | 1,55 | -0,11 | 129,12 | 0,3925 | 1,62 | -0,1131 |
| 129,29 | 0,3538 | 1,59 | -0,1478 | 129,15 | 0,3708 | 1,58 | -0,11 | 129,09 | 0,3925 | 1,65 | -0,1132 |
| 129,26 | 0,3539 | 1,62 | -0,1478 | 129,11 | 0,3708 | 1,62 | -0,1101 | 129,06 | 0,3925 | 1,68 | -0,1132 |
| 129,22 | 0,3539 | 1,66 | -0,1479 | 129,08 | 0,3708 | 1,65 | -0,1101 | 129,02 | 0,3925 | 1,72 | -0,1132 |
| 129,19 | 0,3539 | 1,69 | -0,1479 | 129,05 | 0,3708 | 1,68 | -0,1102 | 128,99 | 0,3925 | 1,75 | -0,1133 |
| 129,16 | 0,354 | 1,72 | -0,148 | 129,01 | 0,3708 | 1,71 | -0,1102 | 128,96 | 0,3925 | 1,78 | -0,1133 |
| 129,12 | 0,354 | 1,76 | -0,148 | 128,98 | 0,3708 | 1,75 | -0,1103 | 128,92 | 0,3925 | 1,82 | -0,1133 |
| 129,09 | 0,3541 | 1,79 | -0,1481 | 128,95 | 0,3708 | 1,78 | -0,1103 | 128,89 | 0,3925 | 1,85 | -0,1133 |
| 129,06 | 0,3541 | 1,82 | -0,1481 | 128,91 | 0,3708 | 1,82 | -0,1104 | 128,86 | 0,3925 | 1,88 | -0,1134 |
| 129,02 | 0,3541 | 1,86 | -0,1481 | 128,88 | 0,3708 | 1,85 | -0,1104 | 128,82 | 0,3925 | 1,92 | -0,1134 |
| 128,99 | 0,3542 | 1,89 | -0,1482 | 128,85 | 0,3708 | 1,88 | -0,1104 | 128,79 | 0,3925 | 1,95 | -0,1134 |
| 128,96 | 0,3542 | 1,92 | -0,1482 | 128,81 | 0,3708 | 1,92 | -0,1105 | 128,76 | 0,3925 | 1,98 | -0,1134 |
| 128,92 | 0,3543 | 1,96 | -0,1483 | 128,78 | 0,3708 | 1,95 | -0,1105 | 128,72 | 0,3925 | 2,02 | -0,1134 |
| 128,89 | 0,3543 | 1,99 | -0,1483 | 128,75 | 0,3709 | 1,98 | -0,1106 | 128,69 | 0,3925 | 2,05 | -0,1135 |
| 128,86 | 0,3544 | 2,02 | -0,1484 | 128,71 | 0,3709 | 2,02 | -0,1106 | 128,66 | 0,3925 | 2,08 | -0,1135 |
| 128,82 | 0,3544 | 2,06 | -0,1484 | 128,68 | 0,3709 | 2,05 | -0,1107 | 128,62 | 0,3925 | 2,12 | -0,1135 |
| 128,79 | 0,3544 | 2,09 | -0,1484 | 128,65 | 0,3709 | 2,08 | -0,1107 | 128,59 | 0,3924 | 2,15 | -0,1136 |
| 128,76 | 0,3545 | 2,12 | -0,1485 | 128,62 | 0,3709 | 2,12 | -0,1108 | 128,56 | 0,3924 | 2,18 | -0,1136 |
| 128,73 | 0,3545 | 2,16 | -0,1485 | 128,58 | 0,3709 | 2,15 | -0,1108 | 128,52 | 0,3924 | 2,22 | -0,1136 |
| 128,69 | 0,3546 | 2,19 | -0,1486 | 128,55 | 0,3709 | 2,18 | -0,1109 | 128,49 | 0,3924 | 2,25 | -0,1136 |
| 128,66 | 0,3546 | 2,22 | -0,1486 | 128,52 | 0,3709 | 2,22 | -0,1109 | 128,46 | 0,3924 | 2,28 | -0,1137 |
| 128,63 | 0,3547 | 2,26 | -0,1487 | 128,48 | 0,3709 | 2,25 | -0,1109 | 128,42 | 0,3924 | 2,32 | -0,1137 |
| 128,59 | 0,3547 | 2,29 | -0,1487 | 128,45 | 0,3709 | 2,28 | -0,111 | 128,39 | 0,3924 | 2,35 | -0,1137 |
| 128,56 | 0,3547 | 2,32 | -0,1487 | 128,41 | 0,3709 | 2,32 | -0,111 | 128,36 | 0,3924 | 2,38 | -0,1137 |
| 128,52 | 0,3548 | 2,36 | -0,1488 | 128,38 | 0,3709 | 2,35 | -0,1111 | 128,32 | 0,3924 | 2,42 | -0,1138 |
| 128,49 | 0,3548 | 2,39 | -0,1488 | 128,35 | 0,3709 | 2,38 | -0,1111 | 128,29 | 0,3924 | 2,45 | -0,1138 |
| 128,46 | 0,3549 | 2,42 | -0,1489 | 128,31 | 0,3709 | 2,42 | -0,1112 | 128,26 | 0,3924 | 2,48 | -0,1138 |
| 128,43 | 0,3549 | 2,46 | -0,1489 | 128,28 | 0,3709 | 2,45 | -0,1112 | 128,22 | 0,3924 | 2,52 | -0,1138 |
| 128,39 | 0,3549 | 2,49 | -0,149 | 128,25 | 0,3709 | 2,48 | -0,1113 | 128,19 | 0,3924 | 2,55 | -0,1139 |
| 128,36 | 0,355 | 2,52 | -0,149 | 128,22 | 0,3709 | 2,52 | -0,1113 | 128,16 | 0,3924 | 2,58 | -0,1139 |
| 128,33 | 0,355 | 2,56 | -0,149 | 128,18 | 0,3709 | 2,55 | -0,1114 | 128,12 | 0,3924 | 2,62 | -0,1139 |
| 128,29 | 0,3551 | 2,59 | -0,1491 | 128,15 | 0,3709 | 2,58 | -0,1114 | 128,09 | 0,3924 | 2,65 | -0,1139 |
| 128,26 | 0,3551 | 2,62 | -0,1491 | 128,12 | 0,3709 | 2,62 | -0,1114 | 128,06 | 0,3924 | 2,68 | -0,114 |
| 128,23 | 0,3552 | 2,66 | -0,1492 | 128,08 | 0,3709 | 2,65 | -0,1115 | 128,03 | 0,3924 | 2,72 | -0,114 |
| 128,19 | 0,3552 | 2,69 | -0,1492 | 128,05 | 0,3709 | 2,68 | -0,1115 | 127,99 | 0,3924 | 2,75 | -0,114 |
| 128,16 | 0,3552 | 2,72 | -0,1493 | 128,01 | 0,3709 | 2,71 | -0,1116 | 127,96 | 0,3924 | 2,78 | -0,114 |
| 128,13 | 0,3553 | 2,76 | -0,1493 | 127,98 | 0,3709 | 2,75 | -0,1116 | 127,92 | 0,3924 | 2,82 | -0,1141 |
| 128,09 | 0,3553 | 2,79 | -0,1493 | 127,95 | 0,3709 | 2,78 | -0,1117 | 127,89 | 0,3923 | 2,85 | -0,1141 |
| 128,06 | 0,3554 | 2,82 | -0,1494 | 127,92 | 0,3709 | 2,81 | -0,1117 | 127,86 | 0,3923 | 2,88 | -0,1141 |
| 128,03 | 0,3554 | 2,86 | -0,1494 | 127,88 | 0,3709 | 2,85 | -0,1118 | 127,82 | 0,3923 | 2,92 | -0,1142 |
| 127,99 | 0,3554 | 2,89 | -0,1495 | 127,85 | 0,3709 | 2,88 | -0,1118 | 127,79 | 0,3923 | 2,95 | -0,1142 |
| 127,96 | 0,3555 | 2,92 | -0,1495 | 127,81 | 0,3709 | 2,92 | -0,1118 | 127,76 | 0,3923 | 2,98 | -0,1142 |
| 127,93 | 0,3555 | 2,96 | -0,1496 | 127,78 | 0,371 | 2,95 | -0,1119 | 127,73 | 0,3923 | 3,02 | -0,1142 |
| 127,89 | 0,3556 | 2,99 | -0,1496 | 127,75 | 0,371 | 2,98 | -0,1119 | 127,69 | 0,3923 | 3,05 | -0,1143 |
| 127,86 | 0,3556 | 3,02 | -0,1497 | 127,72 | 0,371 | 3,01 | -0,112 | 127,66 | 0,3923 | 3,08 | -0,1143 |
| 127,83 | 0,3556 | 3,06 | -0,1497 | 127,68 | 0,371 | 3,05 | -0,112 | 127,62 | 0,3923 | 3,12 | -0,1143 |
| 127,79 | 0,3557 | 3,09 | -0,1498 | 127,65 | 0,371 | 3,08 | -0,1121 | 127,59 | 0,3923 | 3,15 | -0,1143 |
| 127,76 | 0,3557 | 3,12 | -0,1498 | 127,61 | 0,371 | 3,12 | -0,1121 | 127,56 | 0,3923 | 3,18 | -0,1143 |
| 127,73 | 0,3558 | 3,15 | -0,1498 | 127,58 | 0,371 | 3,15 | -0,1122 | 127,52 | 0,3923 | 3,22 | -0,1144 |
| 127,69 | 0,3558 | 3,19 | -0,1499 | 127,55 | 0,371 | 3,18 | -0,1122 | 127,49 | 0,3923 | 3,25 | -0,1144 |
| 127,66 | 0,3558 | 3,22 | -0,1499 | 127,52 | 0,371 | 3,21 | -0,1123 | 127,46 | 0,3923 | 3,28 | -0,1144 |
| 127,63 | 0,3559 | 3,26 | -0,15 | 127,48 | 0,371 | 3,25 | -0,1123 | 127,42 | 0,3923 | 3,32 | -0,1145 |
| 127,59 | 0,3559 | 3,29 | -0,15 | 127,45 | 0,371 | 3,28 | -0,1124 | 127,39 | 0,3922 | 3,35 | -0,1145 |
| 127,56 | 0,356 | 3,32 | -0,1501 | 127,42 | 0,371 | 3,31 | -0,1124 | 127,36 | 0,3922 | 3,38 | -0,1145 |
| 127,53 | 0,356 | 3,36 | -0,1501 | 127,38 | 0,371 | 3,35 | -0,1125 | 127,32 | 0,3922 | 3,42 | -0,1145 |
| 127,49 | 0,356 | 3,39 | -0,1501 | 127,35 | 0,371 | 3,38 | -0,1125 | 127,29 | 0,3922 | 3,45 | -0,1146 |
| 127,46 | 0,3561 | 3,42 | -0,1502 | 127,32 | 0,371 | 3,41 | -0,1126 | 127,26 | 0,3922 | 3,48 | -0,1146 |
| 127,43 | 0,3561 | 3,45 | -0,1502 | 127,28 | 0,371 | 3,45 | -0,1126 | 127,23 | 0,3922 | 3,52 | -0,1146 |
| 127,4 | 0,3562 | 3,49 | -0,1503 | 127,25 | 0,371 | 3,48 | -0,1126 | 127,19 | 0,3922 | 3,55 | -0,1146 |
| 127,36 | 0,3562 | 3,52 | -0,1503 | 127,21 | 0,371 | 3,51 | -0,1127 | 127,16 | 0,3922 | 3,58 | -0,1147 |
| 127,33 | 0,3562 | 3,55 | -0,1504 | 127,18 | 0,371 | 3,55 | -0,1127 | 127,12 | 0,3922 | 3,62 | -0,1147 |
| 127,3 | 0,3563 | 3,59 | -0,1504 | 127,15 | 0,371 | 3,58 | -0,1128 | 127,09 | 0,3922 | 3,65 | -0,1147 |
| 127,26 | 0,3563 | 3,62 | -0,1505 | 127,12 | 0,371 | 3,61 | -0,1128 | 127,06 | 0,3922 | 3,68 | -0,1147 |
| 127,23 | 0,3564 | 3,65 | -0,1505 | 127,08 | 0,371 | 3,65 | -0,1129 | 127,03 | 0,3922 | 3,72 | -0,1148 |
| 127,2 | 0,3564 | 3,69 | -0,1505 | 127,05 | 0,371 | 3,68 | -0,1129 | 126,99 | 0,3922 | 3,75 | -0,1148 |
| 127,16 | 0,3565 | 3,72 | -0,1506 | 127,02 | 0,371 | 3,71 | -0,113 | 126,96 | 0,3922 | 3,78 | -0,1148 |
| 127,13 | 0,3565 | 3,76 | -0,1506 | 126,98 | 0,371 | 3,75 | -0,113 | 126,93 | 0,3922 | 3,82 | -0,1148 |
| 127,1 | 0,3565 | 3,79 | -0,1507 | 126,95 | 0,371 | 3,78 | -0,1131 | 126,89 | 0,3921 | 3,85 | -0,1149 |
| 127,06 | 0,3566 | 3,82 | -0,1507 | 126,92 | 0,371 | 3,81 | -0,1131 | 126,86 | 0,3921 | 3,88 | -0,1149 |
| 127,03 | 0,3566 | 3,85 | -0,1508 | 126,88 | 0,371 | 3,85 | -0,1132 | 126,83 | 0,3921 | 3,92 | -0,1149 |
| 127 | 0,3566 | 3,89 | -0,1508 | 126,85 | 0,371 | 3,88 | -0,1132 | 126,79 | 0,3921 | 3,95 | -0,1149 |
| 126,96 | 0,3567 | 3,92 | -0,1509 | 126,82 | 0,371 | 3,91 | -0,1132 | 126,76 | 0,3921 | 3,98 | -0,115 |
| 126,93 | 0,3567 | 3,95 | -0,1509 | 126,78 | 0,371 | 3,95 | -0,1133 | 126,73 | 0,3921 | 4,02 | -0,115 |
| 126,9 | 0,3568 | 3,99 | -0,1509 | 126,75 | 0,371 | 3,98 | -0,1133 | 126,69 | 0,3921 | 4,05 | -0,115 |
| 126,86 | 0,3568 | 4,02 | -0,151 | 126,72 | 0,371 | 4,01 | -0,1134 | 126,66 | 0,3921 | 4,08 | -0,115 |
| 126,83 | 0,3568 | 4,06 | -0,151 | 126,68 | 0,371 | 4,05 | -0,1134 | 126,63 | 0,3921 | 4,12 | -0,1151 |
| 126,8 | 0,3569 | 4,09 | -0,1511 | 126,65 | 0,371 | 4,08 | -0,1135 | 126,59 | 0,3921 | 4,15 | -0,1151 |
| 126,76 | 0,3569 | 4,12 | -0,1511 | 126,62 | 0,371 | 4,11 | -0,1135 | 126,56 | 0,3921 | 4,18 | -0,1151 |
| 126,73 | 0,357 | 4,16 | -0,1512 | 126,58 | 0,371 | 4,15 | -0,1136 | 126,53 | 0,3921 | 4,22 | -0,1151 |
| 126,7 | 0,357 | 4,19 | -0,1512 | 126,55 | 0,371 | 4,18 | -0,1136 | 126,49 | 0,392 | 4,25 | -0,1152 |
| 126,66 | 0,357 | 4,22 | -0,1513 | 126,52 | 0,371 | 4,21 | -0,1137 | 126,46 | 0,392 | 4,28 | -0,1152 |
| 126,63 | 0,3571 | 4,25 | -0,1513 | 126,48 | 0,371 | 4,25 | -0,1137 | 126,43 | 0,392 | 4,32 | -0,1152 |
| 126,6 | 0,3571 | 4,29 | -0,1513 | 126,45 | 0,371 | 4,28 | -0,1138 | 126,39 | 0,392 | 4,35 | -0,1152 |
| 126,56 | 0,3572 | 4,32 | -0,1514 | 126,42 | 0,371 | 4,31 | -0,1138 | 126,36 | 0,392 | 4,39 | -0,1153 |
| 126,53 | 0,3572 | 4,36 | -0,1514 | 126,38 | 0,371 | 4,35 | -0,1138 | 126,32 | 0,392 | 4,42 | -0,1153 |
| 126,5 | 0,3572 | 4,39 | -0,1515 | 126,35 | 0,371 | 4,38 | -0,1139 | 126,29 | 0,392 | 4,45 | -0,1153 |
| 126,46 | 0,3573 | 4,42 | -0,1515 | 126,32 | 0,371 | 4,41 | -0,1139 | 126,26 | 0,392 | 4,48 | -0,1153 |
| 126,43 | 0,3573 | 4,45 | -0,1516 | 126,28 | 0,371 | 4,45 | -0,114 | 126,23 | 0,392 | 4,52 | -0,1154 |
| 126,4 | 0,3573 | 4,49 | -0,1516 | 126,25 | 0,371 | 4,48 | -0,114 | 126,19 | 0,392 | 4,55 | -0,1154 |
| 126,36 | 0,3574 | 4,52 | -0,1517 | 126,22 | 0,371 | 4,51 | -0,1141 | 126,16 | 0,392 | 4,58 | -0,1154 |
| 126,33 | 0,3574 | 4,55 | -0,1517 | 126,18 | 0,3709 | 4,55 | -0,1141 | 126,13 | 0,3919 | 4,62 | -0,1154 |
| 126,3 | 0,3575 | 4,59 | -0,1518 | 126,15 | 0,3709 | 4,58 | -0,1142 | 126,09 | 0,3919 | 4,65 | -0,1155 |
| 126,26 | 0,3575 | 4,62 | -0,1518 | 126,12 | 0,3709 | 4,61 | -0,1142 | 126,06 | 0,3919 | 4,69 | -0,1155 |
| 126,23 | 0,3575 | 4,65 | -0,1518 | 126,08 | 0,3709 | 4,65 | -0,1143 | 126,03 | 0,3919 | 4,72 | -0,1155 |
| 126,2 | 0,3576 | 4,69 | -0,1519 | 126,05 | 0,3709 | 4,68 | -0,1143 | 125,99 | 0,3919 | 4,75 | -0,1155 |
| 126,17 | 0,3576 | 4,72 | -0,1519 | 126,02 | 0,3709 | 4,71 | -0,1144 | 125,96 | 0,3919 | 4,78 | -0,1156 |
| 126,13 | 0,3576 | 4,75 | -0,152 | 125,98 | 0,3709 | 4,75 | -0,1144 | 125,93 | 0,3919 | 4,82 | -0,1156 |
| 126,1 | 0,3577 | 4,79 | -0,152 | 125,95 | 0,3709 | 4,78 | -0,1145 | 125,89 | 0,3919 | 4,85 | -0,1156 |
| 126,06 | 0,3577 | 4,82 | -0,1521 | 125,92 | 0,3709 | 4,81 | -0,1145 | 125,86 | 0,3918 | 4,88 | -0,1156 |
| 126,03 | 0,3578 | 4,85 | -0,1522 | 125,88 | 0,3709 | 4,85 | -0,1146 | 125,83 | 0,3918 | 4,92 | -0,1157 |
| 126 | 0,3578 | 4,89 | -0,1522 | 125,85 | 0,3709 | 4,88 | -0,1146 | 125,79 | 0,3918 | 4,95 | -0,1157 |
| 125,96 | 0,3578 | 4,92 | -0,1523 | 125,82 | 0,3709 | 4,91 | -0,1147 | 125,76 | 0,3918 | 4,98 | -0,1157 |
| 125,93 | 0,3579 | 4,95 | -0,1524 | 125,78 | 0,3709 | 4,95 | -0,1147 | 125,73 | 0,3918 | 5,02 | -0,1157 |
| 125,9 | 0,3579 | 4,99 | -0,1524 | 125,75 | 0,3709 | 4,98 | -0,1148 | 125,69 | 0,3918 | 5,05 | -0,1158 |
| 125,86 | 0,3579 | 5,02 | -0,1525 | 125,72 | 0,3709 | 5,01 | -0,1148 | 125,66 | 0,3918 | 5,09 | -0,1158 |
| 125,83 | 0,358 | 5,05 | -0,1525 | 125,68 | 0,3709 | 5,05 | -0,1148 | 125,63 | 0,3918 | 5,12 | -0,1158 |
| 125,8 | 0,358 | 5,09 | -0,1526 | 125,65 | 0,3709 | 5,08 | -0,1149 | 125,59 | 0,3918 | 5,15 | -0,1158 |
| 125,77 | 0,358 | 5,12 | -0,1526 | 125,62 | 0,3709 | 5,11 | -0,1149 | 125,56 | 0,3918 | 5,18 | -0,1159 |
| 125,73 | 0,3581 | 5,15 | -0,1526 | 125,58 | 0,3709 | 5,15 | -0,115 | 125,53 | 0,3917 | 5,22 | -0,1159 |
| 125,7 | 0,3581 | 5,19 | -0,1527 | 125,55 | 0,3709 | 5,18 | -0,115 | 125,49 | 0,3917 | 5,25 | -0,1159 |
| 125,66 | 0,3582 | 5,22 | -0,1527 | 125,52 | 0,3709 | 5,21 | -0,1151 | 125,46 | 0,3917 | 5,28 | -0,1159 |
| 125,63 | 0,3582 | 5,25 | -0,1528 | 125,48 | 0,3709 | 5,25 | -0,1151 | 125,43 | 0,3917 | 5,32 | -0,116 |
| 125,6 | 0,3582 | 5,29 | -0,1528 | 125,45 | 0,3709 | 5,28 | -0,1152 | 125,39 | 0,3917 | 5,35 | -0,116 |
| 125,57 | 0,3583 | 5,32 | -0,1529 | 125,42 | 0,3709 | 5,31 | -0,1152 | 125,36 | 0,3917 | 5,38 | -0,116 |
| 125,53 | 0,3583 | 5,35 | -0,1529 | 125,38 | 0,3709 | 5,35 | -0,1153 | 125,33 | 0,3917 | 5,42 | -0,116 |
| 125,5 | 0,3583 | 5,39 | -0,153 | 125,35 | 0,3709 | 5,38 | -0,1153 | 125,29 | 0,3917 | 5,45 | -0,1161 |
| 125,47 | 0,3584 | 5,42 | -0,153 | 125,32 | 0,3709 | 5,41 | -0,1154 | 125,26 | 0,3917 | 5,48 | -0,1161 |
| 125,43 | 0,3584 | 5,45 | -0,153 | 125,28 | 0,3708 | 5,45 | -0,1154 | 125,23 | 0,3916 | 5,52 | -0,1161 |
| 125,4 | 0,3584 | 5,49 | -0,1531 | 125,25 | 0,3708 | 5,48 | -0,1155 | 125,19 | 0,3916 | 5,55 | -0,1161 |
| 125,37 | 0,3585 | 5,52 | -0,1531 | 125,22 | 0,3708 | 5,51 | -0,1155 | 125,16 | 0,3916 | 5,58 | -0,1162 |
| 125,33 | 0,3585 | 5,55 | -0,1532 | 125,18 | 0,3708 | 5,55 | -0,1156 | 125,13 | 0,3916 | 5,62 | -0,1162 |
| 125,3 | 0,3585 | 5,59 | -0,1532 | 125,15 | 0,3708 | 5,58 | -0,1156 | 125,09 | 0,3916 | 5,65 | -0,1162 |
| 125,27 | 0,3586 | 5,62 | -0,1533 | 125,12 | 0,3708 | 5,61 | -0,1157 | 125,06 | 0,3916 | 5,68 | -0,1162 |
| 125,23 | 0,3586 | 5,65 | -0,1533 | 125,08 | 0,3708 | 5,65 | -0,1157 | 125,03 | 0,3916 | 5,72 | -0,1163 |
| 125,2 | 0,3586 | 5,69 | -0,1534 | 125,05 | 0,3708 | 5,68 | -0,1158 | 124,99 | 0,3915 | 5,75 | -0,1163 |
| 125,17 | 0,3587 | 5,72 | -0,1534 | 125,02 | 0,3708 | 5,71 | -0,1158 | 124,96 | 0,3915 | 5,78 | -0,1163 |
| 125,13 | 0,3587 | 5,75 | -0,1535 | 124,98 | 0,3708 | 5,75 | -0,1159 | 124,93 | 0,3915 | 5,82 | -0,1163 |
| 125,1 | 0,3587 | 5,79 | -0,1535 | 124,95 | 0,3708 | 5,78 | -0,1159 | 124,89 | 0,3915 | 5,85 | -0,1164 |
| 125,07 | 0,3588 | 5,82 | -0,1536 | 124,92 | 0,3708 | 5,81 | -0,116 | 124,86 | 0,3915 | 5,88 | -0,1164 |
| 125,03 | 0,3588 | 5,85 | -0,1536 | 124,88 | 0,3708 | 5,85 | -0,116 | 124,83 | 0,3915 | 5,92 | -0,1164 |
| 125 | 0,3588 | 5,89 | -0,1536 | 124,85 | 0,3708 | 5,88 | -0,116 | 124,79 | 0,3915 | 5,95 | -0,1164 |
| 124,97 | 0,3589 | 5,92 | -0,1537 | 124,82 | 0,3708 | 5,91 | -0,1161 | 124,76 | 0,3915 | 5,99 | -0,1165 |
| 124,93 | 0,3589 | 5,95 | -0,1537 | 124,78 | 0,3708 | 5,95 | -0,1162 | 124,73 | 0,3914 | 6,02 | -0,1165 |
| 124,9 | 0,359 | 5,99 | -0,1538 | 124,75 | 0,3708 | 5,98 | -0,1163 | 124,69 | 0,3914 | 6,05 | -0,1165 |
| 124,87 | 0,359 | 6,02 | -0,1539 | 124,72 | 0,3707 | 6,01 | -0,1163 | 124,66 | 0,3914 | 6,08 | -0,1165 |
| 124,83 | 0,359 | 6,05 | -0,1539 | 124,69 | 0,3707 | 6,05 | -0,1164 | 124,63 | 0,3914 | 6,12 | -0,1166 |
| 124,8 | 0,3591 | 6,09 | -0,154 | 124,65 | 0,3707 | 6,08 | -0,1164 | 124,59 | 0,3914 | 6,15 | -0,1166 |
| 124,77 | 0,3591 | 6,12 | -0,154 | 124,62 | 0,3707 | 6,11 | -0,1165 | 124,56 | 0,3914 | 6,18 | -0,1166 |
| 124,73 | 0,3591 | 6,15 | -0,1541 | 124,59 | 0,3707 | 6,15 | -0,1165 | 124,53 | 0,3914 | 6,22 | -0,1166 |
| 124,7 | 0,3591 | 6,19 | -0,1541 | 124,55 | 0,3707 | 6,18 | -0,1166 | 124,49 | 0,3913 | 6,25 | -0,1167 |
| 124,67 | 0,3592 | 6,22 | -0,1541 | 124,52 | 0,3707 | 6,21 | -0,1166 | 124,46 | 0,3913 | 6,28 | -0,1167 |
| 124,63 | 0,3592 | 6,25 | -0,1542 | 124,49 | 0,3707 | 6,25 | -0,1167 | 124,43 | 0,3913 | 6,32 | -0,1167 |
| 124,6 | 0,3592 | 6,29 | -0,1542 | 124,45 | 0,3707 | 6,28 | -0,1167 | 124,39 | 0,3913 | 6,35 | -0,1167 |
| 124,57 | 0,3593 | 6,32 | -0,1543 | 124,42 | 0,3707 | 6,31 | -0,1168 | 124,36 | 0,3913 | 6,39 | -0,1168 |
| 124,53 | 0,3593 | 6,35 | -0,1543 | 124,39 | 0,3707 | 6,35 | -0,1168 | 124,33 | 0,3913 | 6,42 | -0,1168 |
| 124,5 | 0,3593 | 6,39 | -0,1544 | 124,35 | 0,3707 | 6,38 | -0,1169 | 124,29 | 0,3913 | 6,45 | -0,1168 |
| 124,47 | 0,3594 | 6,42 | -0,1544 | 124,32 | 0,3707 | 6,41 | -0,1169 | 124,26 | 0,3912 | 6,48 | -0,1168 |
| 124,43 | 0,3594 | 6,45 | -0,1545 | 124,29 | 0,3707 | 6,44 | -0,117 | 124,23 | 0,3912 | 6,52 | -0,1169 |
| 124,4 | 0,3595 | 6,49 | -0,1545 | 124,25 | 0,3706 | 6,48 | -0,117 | 124,19 | 0,3912 | 6,55 | -0,1169 |
| 124,37 | 0,3595 | 6,52 | -0,1546 | 124,22 | 0,3706 | 6,51 | -0,1171 | 124,16 | 0,3912 | 6,59 | -0,1169 |
| 124,33 | 0,3595 | 6,55 | -0,1546 | 124,19 | 0,3706 | 6,54 | -0,1171 | 124,13 | 0,3912 | 6,62 | -0,1169 |
| 124,3 | 0,3595 | 6,59 | -0,1547 | 124,15 | 0,3706 | 6,58 | -0,1171 | 124,09 | 0,3912 | 6,65 | -0,117 |
| 124,27 | 0,3596 | 6,62 | -0,1547 | 124,12 | 0,3706 | 6,61 | -0,1172 | 124,06 | 0,3912 | 6,69 | -0,117 |
| 124,23 | 0,3596 | 6,65 | -0,1548 | 124,09 | 0,3706 | 6,64 | -0,1172 | 124,03 | 0,3911 | 6,72 | -0,117 |
| 124,2 | 0,3596 | 6,69 | -0,1548 | 124,05 | 0,3706 | 6,68 | -0,1173 | 123,99 | 0,3911 | 6,75 | -0,117 |
| 124,17 | 0,3597 | 6,72 | -0,1549 | 124,02 | 0,3706 | 6,71 | -0,1173 | 123,96 | 0,3911 | 6,78 | -0,1171 |
| 124,13 | 0,3597 | 6,75 | -0,155 | 123,99 | 0,3706 | 6,74 | -0,1174 | 123,93 | 0,3911 | 6,82 | -0,1171 |
| 124,1 | 0,3597 | 6,79 | -0,155 | 123,95 | 0,3706 | 6,78 | -0,1174 | 123,89 | 0,3911 | 6,85 | -0,1171 |
| 124,07 | 0,3598 | 6,82 | -0,155 | 123,92 | 0,3706 | 6,81 | -0,1175 | 123,86 | 0,3911 | 6,88 | -0,1171 |
| 124,03 | 0,3598 | 6,85 | -0,1551 | 123,89 | 0,3706 | 6,85 | -0,1175 | 123,83 | 0,3911 | 6,92 | -0,1172 |
| 124 | 0,3598 | 6,89 | -0,1551 | 123,85 | 0,3706 | 6,88 | -0,1176 | 123,79 | 0,391 | 6,95 | -0,1172 |
| 123,97 | 0,3599 | 6,92 | -0,1552 | 123,82 | 0,3705 | 6,91 | -0,1176 | 123,76 | 0,391 | 6,98 | -0,1172 |
| 123,93 | 0,3599 | 6,95 | -0,1552 | 123,79 | 0,3705 | 6,95 | -0,1177 | 123,73 | 0,391 | 7,02 | -0,1172 |
| 123,9 | 0,3599 | 6,99 | -0,1553 | 123,75 | 0,3705 | 6,98 | -0,1178 | 123,69 | 0,391 | 7,05 | -0,1173 |
| 123,87 | 0,36 | 7,02 | -0,1553 | 123,72 | 0,3705 | 7,01 | -0,1178 | 123,66 | 0,391 | 7,09 | -0,1173 |
| 123,83 | 0,36 | 7,05 | -0,1554 | 123,69 | 0,3705 | 7,04 | -0,1179 | 123,63 | 0,391 | 7,12 | -0,1173 |
| 123,8 | 0,36 | 7,09 | -0,1554 | 123,65 | 0,3705 | 7,08 | -0,1179 | 123,59 | 0,391 | 7,15 | -0,1173 |
| 123,77 | 0,36 | 7,12 | -0,1555 | 123,62 | 0,3705 | 7,11 | -0,118 | 123,56 | 0,3909 | 7,19 | -0,1174 |
| 123,73 | 0,3601 | 7,15 | -0,1555 | 123,59 | 0,3705 | 7,15 | -0,118 | 123,53 | 0,3909 | 7,22 | -0,1174 |
| 123,7 | 0,3601 | 7,19 | -0,1556 | 123,55 | 0,3705 | 7,18 | -0,1181 | 123,5 | 0,3909 | 7,25 | -0,1174 |
| 123,67 | 0,3601 | 7,22 | -0,1556 | 123,52 | 0,3705 | 7,21 | -0,1181 | 123,46 | 0,3909 | 7,28 | -0,1174 |
| 123,63 | 0,3602 | 7,25 | -0,1557 | 123,49 | 0,3705 | 7,25 | -0,1182 | 123,43 | 0,3909 | 7,32 | -0,1175 |
| 123,6 | 0,3602 | 7,28 | -0,1558 | 123,45 | 0,3705 | 7,28 | -0,1182 | 123,39 | 0,3909 | 7,35 | -0,1175 |
| 123,57 | 0,3602 | 7,32 | -0,1558 | 123,42 | 0,3704 | 7,31 | -0,1183 | 123,36 | 0,3908 | 7,38 | -0,1175 |
| 123,53 | 0,3603 | 7,35 | -0,1559 | 123,39 | 0,3704 | 7,34 | -0,1183 | 123,33 | 0,3908 | 7,42 | -0,1175 |
| 123,5 | 0,3603 | 7,39 | -0,1559 | 123,35 | 0,3704 | 7,38 | -0,1184 | 123,29 | 0,3908 | 7,45 | -0,1176 |
| 123,47 | 0,3603 | 7,42 | -0,156 | 123,32 | 0,3704 | 7,41 | -0,1184 | 123,26 | 0,3908 | 7,48 | -0,1176 |
| 123,43 | 0,3603 | 7,45 | -0,156 | 123,29 | 0,3704 | 7,44 | -0,1185 | 123,23 | 0,3908 | 7,52 | -0,1176 |
| 123,4 | 0,3604 | 7,49 | -0,1561 | 123,25 | 0,3704 | 7,48 | -0,1185 | 123,2 | 0,3908 | 7,55 | -0,1176 |
| 123,37 | 0,3604 | 7,52 | -0,1561 | 123,22 | 0,3704 | 7,51 | -0,1186 | 123,16 | 0,3908 | 7,59 | -0,1177 |
| 123,33 | 0,3604 | 7,55 | -0,1562 | 123,19 | 0,3704 | 7,54 | -0,1186 | 123,13 | 0,3907 | 7,62 | -0,1177 |
| 123,3 | 0,3605 | 7,59 | -0,1562 | 123,15 | 0,3704 | 7,58 | -0,1187 | 123,1 | 0,3907 | 7,65 | -0,1177 |
| 123,27 | 0,3605 | 7,62 | -0,1563 | 123,12 | 0,3704 | 7,61 | -0,1187 | 123,06 | 0,3907 | 7,68 | -0,1177 |
| 123,24 | 0,3605 | 7,65 | -0,1563 | 123,09 | 0,3704 | 7,65 | -0,1188 | 123,03 | 0,3907 | 7,72 | -0,1178 |
| 123,2 | 0,3606 | 7,68 | -0,1564 | 123,05 | 0,3703 | 7,68 | -0,1188 | 122,99 | 0,3907 | 7,75 | -0,1178 |
| 123,17 | 0,3606 | 7,72 | -0,1564 | 123,02 | 0,3703 | 7,71 | -0,1189 | 122,96 | 0,3907 | 7,78 | -0,1178 |
| 123,13 | 0,3606 | 7,75 | -0,1565 | 122,99 | 0,3703 | 7,74 | -0,1189 | 122,93 | 0,3906 | 7,82 | -0,1178 |
| 123,1 | 0,3606 | 7,79 | -0,1565 | 122,95 | 0,3703 | 7,78 | -0,119 | 122,89 | 0,3906 | 7,85 | -0,1179 |
| 123,07 | 0,3607 | 7,82 | -0,1566 | 122,92 | 0,3703 | 7,81 | -0,119 | 122,86 | 0,3906 | 7,89 | -0,1179 |
| 123,04 | 0,3607 | 7,85 | -0,1566 | 122,89 | 0,3703 | 7,84 | -0,1191 | 122,83 | 0,3906 | 7,92 | -0,1179 |
| 123 | 0,3607 | 7,89 | -0,1567 | 122,85 | 0,3703 | 7,88 | -0,1191 | 122,79 | 0,3906 | 7,95 | -0,1179 |
| 122,97 | 0,3608 | 7,92 | -0,1568 | 122,82 | 0,3703 | 7,91 | -0,1192 | 122,76 | 0,3906 | 7,98 | -0,118 |
| 122,94 | 0,3608 | 7,95 | -0,1568 | 122,79 | 0,3703 | 7,94 | -0,1192 | 122,73 | 0,3905 | 8,02 | -0,118 |
| 122,9 | 0,3608 | 7,99 | -0,1568 | 122,75 | 0,3702 | 7,98 | -0,1193 | 122,7 | 0,3905 | 8,05 | -0,118 |
| 122,87 | 0,3609 | 8,02 | -0,1569 | 122,72 | 0,3702 | 8,01 | -0,1193 | 122,66 | 0,3905 | 8,09 | -0,118 |
| 122,84 | 0,3609 | 8,05 | -0,1569 | 122,69 | 0,3702 | 8,04 | -0,1194 | 122,63 | 0,3905 | 8,12 | -0,1181 |
| 122,8 | 0,3609 | 8,08 | -0,157 | 122,65 | 0,3702 | 8,08 | -0,1194 | 122,6 | 0,3905 | 8,15 | -0,1181 |
| 122,77 | 0,3609 | 8,12 | -0,157 | 122,62 | 0,3702 | 8,11 | -0,1195 | 122,56 | 0,3905 | 8,19 | -0,1181 |
| 122,74 | 0,361 | 8,15 | -0,1571 | 122,59 | 0,3702 | 8,14 | -0,1195 | 122,53 | 0,3905 | 8,22 | -0,1181 |
| 122,7 | 0,361 | 8,19 | -0,1572 | 122,55 | 0,3702 | 8,18 | -0,1196 | 122,49 | 0,3904 | 8,25 | -0,1182 |
| 122,67 | 0,361 | 8,22 | -0,1572 | 122,52 | 0,3702 | 8,21 | -0,1196 | 122,46 | 0,3904 | 8,28 | -0,1182 |
| 122,64 | 0,3611 | 8,25 | -0,1573 | 122,49 | 0,3702 | 8,24 | -0,1197 | 122,43 | 0,3904 | 8,32 | -0,1182 |
| 122,6 | 0,3611 | 8,29 | -0,1573 | 122,45 | 0,3702 | 8,28 | -0,1197 | 122,4 | 0,3904 | 8,35 | -0,1182 |
| 122,57 | 0,3611 | 8,32 | -0,1574 | 122,42 | 0,3701 | 8,31 | -0,1198 | 122,36 | 0,3904 | 8,39 | -0,1183 |
| 122,54 | 0,3611 | 8,35 | -0,1574 | 122,39 | 0,3701 | 8,34 | -0,1198 | 122,33 | 0,3903 | 8,42 | -0,1183 |
| 122,5 | 0,3612 | 8,39 | -0,1575 | 122,35 | 0,3701 | 8,38 | -0,1199 | 122,3 | 0,3903 | 8,45 | -0,1183 |
| 122,47 | 0,3612 | 8,42 | -0,1575 | 122,32 | 0,3701 | 8,41 | -0,12 | 122,26 | 0,3903 | 8,49 | -0,1183 |
| 122,44 | 0,3612 | 8,45 | -0,1576 | 122,29 | 0,3701 | 8,44 | -0,12 | 122,23 | 0,3903 | 8,52 | -0,1183 |
| 122,4 | 0,3613 | 8,48 | -0,1577 | 122,25 | 0,3701 | 8,48 | -0,1201 | 122,2 | 0,3903 | 8,55 | -0,1184 |
| 122,37 | 0,3613 | 8,52 | -0,1577 | 122,22 | 0,3701 | 8,51 | -0,1201 | 122,16 | 0,3903 | 8,58 | -0,1184 |
| 122,34 | 0,3613 | 8,55 | -0,1578 | 122,19 | 0,3701 | 8,54 | -0,1202 | 122,13 | 0,3903 | 8,62 | -0,1184 |
| 122,3 | 0,3613 | 8,59 | -0,1578 | 122,15 | 0,3701 | 8,58 | -0,1202 | 122,1 | 0,3902 | 8,65 | -0,1185 |
| 122,27 | 0,3614 | 8,62 | -0,1579 | 122,12 | 0,37 | 8,61 | -0,1203 | 122,06 | 0,3902 | 8,68 | -0,1185 |
| 122,24 | 0,3614 | 8,65 | -0,1579 | 122,09 | 0,37 | 8,64 | -0,1203 | 122,03 | 0,3902 | 8,72 | -0,1185 |
| 122,2 | 0,3614 | 8,68 | -0,158 | 122,05 | 0,37 | 8,68 | -0,1204 | 122 | 0,3902 | 8,75 | -0,1185 |
| 122,17 | 0,3614 | 8,72 | -0,158 | 122,02 | 0,37 | 8,71 | -0,1204 | 121,96 | 0,3902 | 8,78 | -0,1186 |
| 122,14 | 0,3615 | 8,75 | -0,1581 | 121,99 | 0,37 | 8,74 | -0,1205 | 121,93 | 0,3902 | 8,82 | -0,1186 |
| 122,1 | 0,3615 | 8,78 | -0,1581 | 121,95 | 0,37 | 8,78 | -0,1205 | 121,89 | 0,3902 | 8,85 | -0,1186 |
| 122,07 | 0,3615 | 8,82 | -0,1582 | 121,92 | 0,37 | 8,81 | -0,1206 | 121,86 | 0,3901 | 8,88 | -0,1186 |
| 122,04 | 0,3616 | 8,85 | -0,1582 | 121,89 | 0,37 | 8,84 | -0,1207 | 121,83 | 0,3901 | 8,92 | -0,1187 |
| 122 | 0,3616 | 8,88 | -0,1583 | 121,85 | 0,37 | 8,88 | -0,1207 | 121,8 | 0,3901 | 8,95 | -0,1187 |
| 121,97 | 0,3616 | 8,92 | -0,1584 | 121,82 | 0,3699 | 8,91 | -0,1208 | 121,76 | 0,3901 | 8,98 | -0,1187 |
| 121,94 | 0,3616 | 8,95 | -0,1584 | 121,79 | 0,3699 | 8,94 | -0,1208 | 121,73 | 0,3901 | 9,02 | -0,1187 |
| 121,9 | 0,3617 | 8,98 | -0,1585 | 121,75 | 0,3699 | 8,98 | -0,1209 | 121,7 | 0,39 | 9,05 | -0,1188 |
| 121,87 | 0,3617 | 9,02 | -0,1585 | 121,72 | 0,3699 | 9,01 | -0,1209 | 121,66 | 0,39 | 9,08 | -0,1188 |
| 121,84 | 0,3617 | 9,05 | -0,1586 | 121,69 | 0,3699 | 9,04 | -0,121 | 121,63 | 0,39 | 9,12 | -0,1188 |
| 121,8 | 0,3617 | 9,08 | -0,1586 | 121,65 | 0,3699 | 9,08 | -0,121 | 121,6 | 0,39 | 9,15 | -0,1188 |
| 121,77 | 0,3618 | 9,12 | -0,1587 | 121,62 | 0,3699 | 9,11 | -0,1211 | 121,56 | 0,39 | 9,18 | -0,1189 |
| 121,74 | 0,3618 | 9,15 | -0,1588 | 121,59 | 0,3699 | 9,14 | -0,1211 | 121,53 | 0,39 | 9,22 | -0,1189 |
| 121,7 | 0,3618 | 9,18 | -0,1588 | 121,55 | 0,3699 | 9,18 | -0,1212 | 121,5 | 0,39 | 9,25 | -0,1189 |
| 121,67 | 0,3618 | 9,22 | -0,1589 | 121,52 | 0,3699 | 9,21 | -0,1213 | 121,46 | 0,3899 | 9,28 | -0,1189 |
| 121,64 | 0,3619 | 9,25 | -0,1589 | 121,49 | 0,3698 | 9,24 | -0,1214 | 121,43 | 0,3899 | 9,32 | -0,119 |
| 121,6 | 0,3619 | 9,28 | -0,159 | 121,45 | 0,3698 | 9,28 | -0,1215 | 121,4 | 0,3899 | 9,35 | -0,119 |
| 121,57 | 0,3619 | 9,32 | -0,159 | 121,42 | 0,3698 | 9,31 | -0,1215 | 121,36 | 0,3899 | 9,38 | -0,119 |
| 121,54 | 0,362 | 9,35 | -0,1591 | 121,39 | 0,3698 | 9,34 | -0,1216 | 121,33 | 0,3899 | 9,42 | -0,119 |
| 121,51 | 0,362 | 9,38 | -0,1591 | 121,35 | 0,3698 | 9,38 | -0,1216 | 121,3 | 0,3898 | 9,45 | -0,1191 |
| 121,47 | 0,362 | 9,42 | -0,1592 | 121,32 | 0,3698 | 9,41 | -0,1217 | 121,26 | 0,3898 | 9,48 | -0,1191 |
| 121,44 | 0,362 | 9,45 | -0,1592 | 121,29 | 0,3698 | 9,44 | -0,1217 | 121,23 | 0,3898 | 9,52 | -0,1191 |
| 121,4 | 0,3621 | 9,48 | -0,1593 | 121,25 | 0,3698 | 9,48 | -0,1218 | 121,2 | 0,3898 | 9,55 | -0,1191 |
| 121,37 | 0,3621 | 9,52 | -0,1593 | 121,22 | 0,3697 | 9,51 | -0,1218 | 121,16 | 0,3898 | 9,58 | -0,1192 |
| 121,34 | 0,3621 | 9,55 | -0,1594 | 121,19 | 0,3697 | 9,54 | -0,1219 | 121,13 | 0,3898 | 9,62 | -0,1192 |
| 121,31 | 0,3621 | 9,58 | -0,1594 | 121,15 | 0,3697 | 9,58 | -0,1219 | 121,1 | 0,3897 | 9,65 | -0,1192 |
| 121,27 | 0,3622 | 9,62 | -0,1595 | 121,12 | 0,3697 | 9,61 | -0,122 | 121,06 | 0,3897 | 9,68 | -0,1192 |
| 121,24 | 0,3622 | 9,65 | -0,1595 | 121,09 | 0,3697 | 9,64 | -0,122 | 121,03 | 0,3897 | 9,72 | -0,1193 |
| 121,2 | 0,3622 | 9,68 | -0,1596 | 121,06 | 0,3697 | 9,68 | -0,1221 | 121 | 0,3897 | 9,75 | -0,1193 |
| 121,17 | 0,3622 | 9,72 | -0,1597 | 121,02 | 0,3697 | 9,71 | -0,1222 | 120,96 | 0,3897 | 9,78 | -0,1193 |
| 121,14 | 0,3623 | 9,75 | -0,1598 | 120,99 | 0,3697 | 9,74 | -0,1222 | 120,93 | 0,3897 | 9,82 | -0,1193 |
| 121,1 | 0,3623 | 9,78 | -0,1598 | 120,96 | 0,3696 | 9,78 | -0,1223 | 120,9 | 0,3896 | 9,85 | -0,1194 |
| 121,07 | 0,3623 | 9,82 | -0,1599 | 120,92 | 0,3696 | 9,81 | -0,1223 | 120,86 | 0,3896 | 9,88 | -0,1194 |
| 121,04 | 0,3623 | 9,85 | -0,1599 | 120,89 | 0,3696 | 9,84 | -0,1224 | 120,83 | 0,3896 | 9,92 | -0,1194 |
| 121 | 0,3624 | 9,88 | -0,16 | 120,86 | 0,3696 | 9,88 | -0,1224 | 120,8 | 0,3896 | 9,95 | -0,1194 |
| 120,97 | 0,3624 | 9,92 | -0,16 | 120,82 | 0,3696 | 9,91 | -0,1225 | 120,76 | 0,3896 | 9,98 | -0,1195 |
| 120,94 | 0,3624 | 9,95 | -0,1601 | 120,79 | 0,3696 | 9,94 | -0,1225 | 120,73 | 0,3895 | 10,02 | -0,1195 |
| 120,91 | 0,3624 | 9,98 | -0,1601 | 120,75 | 0,3696 | 9,97 | -0,1226 | 120,7 | 0,3895 | 10,05 | -0,1195 |
| 120,87 | 0,3625 | 10,02 | -0,1602 | 120,72 | 0,3696 | 10,01 | -0,1226 | 120,66 | 0,3895 | 10,08 | -0,1195 |
| 120,84 | 0,3625 | 10,05 | -0,1603 | 120,69 | 0,3695 | 10,04 | -0,1227 | 120,63 | 0,3895 | 10,12 | -0,1196 |
| 120,81 | 0,3625 | 10,08 | -0,1604 | 120,66 | 0,3695 | 10,08 | -0,1228 | 120,6 | 0,3895 | 10,15 | -0,1196 |
| 120,77 | 0,3625 | 10,12 | -0,1604 | 120,62 | 0,3695 | 10,11 | -0,1228 | 120,56 | 0,3895 | 10,18 | -0,1196 |
| 120,74 | 0,3626 | 10,15 | -0,1605 | 120,59 | 0,3695 | 10,14 | -0,1229 | 120,53 | 0,3894 | 10,22 | -0,1196 |
| 120,71 | 0,3626 | 10,18 | -0,1605 | 120,56 | 0,3695 | 10,18 | -0,1229 | 120,5 | 0,3894 | 10,25 | -0,1197 |
| 120,67 | 0,3626 | 10,22 | -0,1606 | 120,52 | 0,3695 | 10,21 | -0,123 | 120,46 | 0,3894 | 10,28 | -0,1197 |
| 120,64 | 0,3626 | 10,25 | -0,1606 | 120,49 | 0,3695 | 10,24 | -0,123 | 120,43 | 0,3894 | 10,32 | -0,1197 |
| 120,6 | 0,3627 | 10,28 | -0,1607 | 120,46 | 0,3695 | 10,28 | -0,1231 | 120,4 | 0,3894 | 10,35 | -0,1197 |
| 120,57 | 0,3627 | 10,32 | -0,1608 | 120,42 | 0,3694 | 10,31 | -0,1231 | 120,36 | 0,3893 | 10,38 | -0,1198 |
| 120,54 | 0,3627 | 10,35 | -0,1608 | 120,39 | 0,3694 | 10,34 | -0,1232 | 120,33 | 0,3893 | 10,42 | -0,1198 |
| 120,51 | 0,3627 | 10,38 | -0,1609 | 120,36 | 0,3694 | 10,38 | -0,1232 | 120,3 | 0,3893 | 10,45 | -0,1198 |
| 120,47 | 0,3627 | 10,42 | -0,1609 | 120,32 | 0,3694 | 10,41 | -0,1233 | 120,26 | 0,3893 | 10,48 | -0,1198 |
| 120,44 | 0,3628 | 10,45 | -0,161 | 120,29 | 0,3694 | 10,44 | -0,1233 | 120,23 | 0,3893 | 10,52 | -0,1199 |
| 120,41 | 0,3628 | 10,48 | -0,161 | 120,26 | 0,3694 | 10,48 | -0,1234 | 120,2 | 0,3893 | 10,55 | -0,1199 |
| 120,37 | 0,3628 | 10,52 | -0,1611 | 120,22 | 0,3694 | 10,51 | -0,1234 | 120,16 | 0,3892 | 10,58 | -0,1199 |
| 120,34 | 0,3628 | 10,55 | -0,1611 | 120,19 | 0,3693 | 10,54 | -0,1235 | 120,13 | 0,3892 | 10,62 | -0,12 |
| 120,31 | 0,3629 | 10,58 | -0,1612 | 120,16 | 0,3693 | 10,57 | -0,1236 | 120,1 | 0,3892 | 10,65 | -0,12 |
| 120,27 | 0,3629 | 10,62 | -0,1612 | 120,12 | 0,3693 | 10,61 | -0,1236 | 120,06 | 0,3892 | 10,68 | -0,12 |
| 120,24 | 0,3629 | 10,65 | -0,1613 | 120,09 | 0,3693 | 10,64 | -0,1237 | 120,03 | 0,3892 | 10,72 | -0,12 |
| 120,21 | 0,3629 | 10,68 | -0,1614 | 120,06 | 0,3693 | 10,67 | -0,1237 | 120 | 0,3891 | 10,75 | -0,1201 |
| 120,17 | 0,363 | 10,72 | -0,1614 | 120,02 | 0,3693 | 10,71 | -0,1238 | 119,96 | 0,3891 | 10,78 | -0,1201 |
| 120,14 | 0,363 | 10,75 | -0,1615 | 119,99 | 0,3693 | 10,74 | -0,1238 | 119,93 | 0,3891 | 10,82 | -0,1201 |
| 120,11 | 0,363 | 10,78 | -0,1615 | 119,96 | 0,3693 | 10,77 | -0,1239 | 119,9 | 0,3891 | 10,85 | -0,1201 |
| 120,07 | 0,363 | 10,82 | -0,1616 | 119,92 | 0,3692 | 10,81 | -0,1239 | 119,86 | 0,3891 | 10,88 | -0,1202 |
| 120,04 | 0,3631 | 10,85 | -0,1616 | 119,89 | 0,3692 | 10,84 | -0,124 | 119,83 | 0,3891 | 10,92 | -0,1202 |
| 120,01 | 0,3631 | 10,88 | -0,1617 | 119,85 | 0,3692 | 10,87 | -0,124 | 119,8 | 0,389 | 10,95 | -0,1202 |
| 119,97 | 0,3631 | 10,92 | -0,1617 | 119,82 | 0,3692 | 10,91 | -0,1241 | 119,76 | 0,389 | 10,98 | -0,1202 |
| 119,94 | 0,3631 | 10,95 | -0,1618 | 119,79 | 0,3692 | 10,94 | -0,1241 | 119,73 | 0,389 | 11,02 | -0,1203 |
| 119,91 | 0,3631 | 10,98 | -0,1618 | 119,76 | 0,3692 | 10,97 | -0,1242 | 119,7 | 0,389 | 11,05 | -0,1203 |
| 119,87 | 0,3632 | 11,02 | -0,1619 | 119,72 | 0,3692 | 11,01 | -0,1243 | 119,66 | 0,3889 | 11,08 | -0,1203 |
| 119,84 | 0,3632 | 11,05 | -0,1619 | 119,69 | 0,3691 | 11,04 | -0,1244 | 119,63 | 0,3889 | 11,12 | -0,1203 |
| 119,81 | 0,3632 | 11,08 | -0,162 | 119,66 | 0,3691 | 11,07 | -0,1244 | 119,6 | 0,3889 | 11,15 | -0,1204 |
| 119,77 | 0,3632 | 11,12 | -0,1621 | 119,62 | 0,3691 | 11,11 | -0,1245 | 119,56 | 0,3889 | 11,18 | -0,1204 |
| 119,74 | 0,3633 | 11,15 | -0,1622 | 119,59 | 0,3691 | 11,14 | -0,1245 | 119,53 | 0,3889 | 11,22 | -0,1204 |
| 119,71 | 0,3633 | 11,18 | -0,1622 | 119,56 | 0,3691 | 11,17 | -0,1246 | 119,5 | 0,3889 | 11,25 | -0,1204 |
| 119,67 | 0,3633 | 11,22 | -0,1623 | 119,52 | 0,3691 | 11,21 | -0,1247 | 119,46 | 0,3888 | 11,28 | -0,1205 |
| 119,64 | 0,3633 | 11,25 | -0,1623 | 119,49 | 0,369 | 11,24 | -0,1247 | 119,43 | 0,3888 | 11,32 | -0,1205 |
| 119,61 | 0,3633 | 11,28 | -0,1624 | 119,46 | 0,369 | 11,27 | -0,1248 | 119,4 | 0,3888 | 11,35 | -0,1205 |
| 119,57 | 0,3634 | 11,32 | -0,1625 | 119,42 | 0,369 | 11,31 | -0,1249 | 119,36 | 0,3888 | 11,38 | -0,1205 |
| 119,54 | 0,3634 | 11,35 | -0,1625 | 119,39 | 0,369 | 11,34 | -0,1249 | 119,33 | 0,3888 | 11,42 | -0,1206 |
| 119,51 | 0,3634 | 11,38 | -0,1626 | 119,36 | 0,369 | 11,37 | -0,125 | 119,3 | 0,3887 | 11,45 | -0,1206 |
| 119,47 | 0,3634 | 11,42 | -0,1626 | 119,32 | 0,369 | 11,41 | -0,125 | 119,26 | 0,3887 | 11,48 | -0,1206 |
| 119,44 | 0,3634 | 11,45 | -0,1627 | 119,29 | 0,369 | 11,44 | -0,1251 | 119,23 | 0,3887 | 11,52 | -0,1206 |
| 119,41 | 0,3635 | 11,48 | -0,1627 | 119,26 | 0,369 | 11,48 | -0,1251 | 119,2 | 0,3887 | 11,55 | -0,1207 |
| 119,37 | 0,3635 | 11,52 | -0,1628 | 119,22 | 0,3689 | 11,51 | -0,1252 | 119,16 | 0,3887 | 11,58 | -0,1207 |
| 119,34 | 0,3635 | 11,55 | -0,1629 | 119,19 | 0,3689 | 11,54 | -0,1252 | 119,13 | 0,3887 | 11,62 | -0,1207 |
| 119,31 | 0,3635 | 11,58 | -0,1629 | 119,16 | 0,3689 | 11,57 | -0,1253 | 119,1 | 0,3886 | 11,65 | -0,1207 |
| 119,27 | 0,3636 | 11,62 | -0,163 | 119,12 | 0,3689 | 11,61 | -0,1254 | 119,06 | 0,3886 | 11,68 | -0,1208 |
| 119,24 | 0,3636 | 11,65 | -0,163 | 119,09 | 0,3689 | 11,64 | -0,1254 | 119,03 | 0,3886 | 11,72 | -0,1208 |
| 119,21 | 0,3636 | 11,68 | -0,1631 | 119,06 | 0,3689 | 11,67 | -0,1255 | 119 | 0,3886 | 11,75 | -0,1208 |
| 119,17 | 0,3636 | 11,72 | -0,1631 | 119,02 | 0,3689 | 11,71 | -0,1256 | 118,96 | 0,3885 | 11,78 | -0,1208 |
| 119,14 | 0,3636 | 11,75 | -0,1632 | 118,99 | 0,3688 | 11,74 | -0,1256 | 118,93 | 0,3885 | 11,82 | -0,1209 |
| 119,11 | 0,3637 | 11,78 | -0,1633 | 118,96 | 0,3688 | 11,78 | -0,1257 | 118,9 | 0,3885 | 11,85 | -0,1209 |
| 119,07 | 0,3637 | 11,82 | -0,1633 | 118,92 | 0,3688 | 11,81 | -0,1258 | 118,86 | 0,3885 | 11,88 | -0,1209 |
| 119,04 | 0,3637 | 11,85 | -0,1634 | 118,89 | 0,3688 | 11,84 | -0,1258 | 118,83 | 0,3885 | 11,92 | -0,121 |
| 119,01 | 0,3637 | 11,88 | -0,1635 | 118,86 | 0,3688 | 11,87 | -0,1259 | 118,8 | 0,3884 | 11,95 | -0,121 |
| 118,98 | 0,3637 | 11,91 | -0,1636 | 118,82 | 0,3688 | 11,91 | -0,1259 | 118,76 | 0,3884 | 11,98 | -0,121 |
| 118,94 | 0,3638 | 11,95 | -0,1636 | 118,79 | 0,3687 | 11,94 | -0,126 | 118,73 | 0,3884 | 12,02 | -0,121 |
| 118,91 | 0,3638 | 11,98 | -0,1637 | 118,76 | 0,3687 | 11,97 | -0,126 | 118,7 | 0,3884 | 12,05 | -0,1211 |
| 118,87 | 0,3638 | 12,02 | -0,1637 | 118,72 | 0,3687 | 12,01 | -0,1261 | 118,66 | 0,3884 | 12,08 | -0,1211 |
| 118,84 | 0,3638 | 12,05 | -0,1638 | 118,69 | 0,3687 | 12,04 | -0,1262 | 118,63 | 0,3884 | 12,12 | -0,1211 |
| 118,81 | 0,3638 | 12,08 | -0,1638 | 118,66 | 0,3687 | 12,07 | -0,1262 | 118,6 | 0,3883 | 12,15 | -0,1211 |
| 118,77 | 0,3639 | 12,12 | -0,1639 | 118,62 | 0,3687 | 12,11 | -0,1263 | 118,56 | 0,3883 | 12,18 | -0,1212 |
| 118,74 | 0,3639 | 12,15 | -0,164 | 118,59 | 0,3687 | 12,14 | -0,1263 | 118,53 | 0,3883 | 12,22 | -0,1212 |
| 118,71 | 0,3639 | 12,18 | -0,164 | 118,56 | 0,3686 | 12,17 | -0,1264 | 118,5 | 0,3883 | 12,25 | -0,1212 |
| 118,67 | 0,3639 | 12,22 | -0,1641 | 118,52 | 0,3686 | 12,21 | -0,1264 | 118,46 | 0,3883 | 12,28 | -0,1212 |
| 118,64 | 0,3639 | 12,25 | -0,1641 | 118,49 | 0,3686 | 12,24 | -0,1265 | 118,43 | 0,3882 | 12,32 | -0,1213 |
| 118,61 | 0,364 | 12,28 | -0,1642 | 118,46 | 0,3686 | 12,27 | -0,1266 | 118,4 | 0,3882 | 12,35 | -0,1213 |
| 118,58 | 0,364 | 12,32 | -0,1643 | 118,42 | 0,3686 | 12,31 | -0,1266 | 118,36 | 0,3882 | 12,38 | -0,1213 |
| 118,54 | 0,364 | 12,35 | -0,1643 | 118,39 | 0,3686 | 12,34 | -0,1267 | 118,33 | 0,3882 | 12,42 | -0,1213 |
| 118,51 | 0,364 | 12,38 | -0,1644 | 118,36 | 0,3685 | 12,37 | -0,1267 | 118,3 | 0,3881 | 12,45 | -0,1214 |
| 118,48 | 0,364 | 12,41 | -0,1644 | 118,33 | 0,3685 | 12,41 | -0,1268 | 118,26 | 0,3881 | 12,49 | -0,1214 |
| 118,44 | 0,3641 | 12,45 | -0,1645 | 118,29 | 0,3685 | 12,44 | -0,1269 | 118,23 | 0,3881 | 12,52 | -0,1214 |
| 118,41 | 0,3641 | 12,48 | -0,1646 | 118,26 | 0,3685 | 12,48 | -0,1269 | 118,2 | 0,3881 | 12,55 | -0,1214 |
| 118,37 | 0,3641 | 12,51 | -0,1647 | 118,22 | 0,3685 | 12,51 | -0,127 | 118,16 | 0,3881 | 12,58 | -0,1215 |
| 118,34 | 0,3641 | 12,55 | -0,1648 | 118,19 | 0,3684 | 12,54 | -0,127 | 118,13 | 0,388 | 12,62 | -0,1215 |
| 118,31 | 0,3641 | 12,58 | -0,1648 | 118,16 | 0,3684 | 12,57 | -0,1271 | 118,1 | 0,388 | 12,65 | -0,1215 |
| 118,28 | 0,3642 | 12,62 | -0,1649 | 118,12 | 0,3684 | 12,61 | -0,1271 | 118,06 | 0,388 | 12,69 | -0,1215 |
| 118,24 | 0,3642 | 12,65 | -0,1649 | 118,09 | 0,3684 | 12,64 | -0,1272 | 118,03 | 0,388 | 12,72 | -0,1216 |
| 118,21 | 0,3642 | 12,68 | -0,165 | 118,06 | 0,3684 | 12,67 | -0,1273 | 118 | 0,388 | 12,75 | -0,1216 |
| 118,18 | 0,3642 | 12,72 | -0,1651 | 118,02 | 0,3684 | 12,71 | -0,1273 | 117,96 | 0,3879 | 12,79 | -0,1216 |
| 118,14 | 0,3642 | 12,75 | -0,1653 | 117,99 | 0,3684 | 12,74 | -0,1274 | 117,93 | 0,3879 | 12,82 | -0,1216 |
| 118,11 | 0,3642 | 12,78 | -0,1655 | 117,96 | 0,3683 | 12,77 | -0,1274 | 117,9 | 0,3879 | 12,85 | -0,1217 |
| 118,08 | 0,3643 | 12,81 | -0,1656 | 117,92 | 0,3683 | 12,81 | -0,1275 | 117,87 | 0,3879 | 12,88 | -0,1217 |
| 118,04 | 0,3643 | 12,85 | -0,1656 | 117,89 | 0,3683 | 12,84 | -0,1275 | 117,83 | 0,3878 | 12,92 | -0,1217 |
| 118,01 | 0,3643 | 12,88 | -0,1657 | 117,86 | 0,3683 | 12,87 | -0,1276 | 117,8 | 0,3878 | 12,95 | -0,1217 |
| 117,98 | 0,3643 | 12,91 | -0,1657 | 117,82 | 0,3683 | 12,91 | -0,1276 | 117,76 | 0,3878 | 12,99 | -0,1218 |
| 117,94 | 0,3643 | 12,95 | -0,1658 | 117,79 | 0,3683 | 12,94 | -0,1277 | 117,73 | 0,3878 | 13,02 | -0,1218 |
| 117,91 | 0,3643 | 12,98 | -0,1659 | 117,76 | 0,3682 | 12,97 | -0,1277 | 117,7 | 0,3877 | 13,05 | -0,1218 |
| 117,88 | 0,3644 | 13,01 | -0,166 | 117,73 | 0,3682 | 13,01 | -0,1278 | 117,66 | 0,3877 | 13,09 | -0,1218 |
| 117,84 | 0,3644 | 13,05 | -0,166 | 117,69 | 0,3682 | 13,04 | -0,1278 | 117,63 | 0,3877 | 13,12 | -0,1219 |
| 117,81 | 0,3644 | 13,08 | -0,1661 | 117,66 | 0,3682 | 13,07 | -0,1279 | 117,6 | 0,3877 | 13,15 | -0,1219 |
| 117,78 | 0,3644 | 13,11 | -0,1661 | 117,62 | 0,3682 | 13,11 | -0,128 | 117,56 | 0,3876 | 13,19 | -0,1219 |
| 117,74 | 0,3644 | 13,15 | -0,1662 | 117,59 | 0,3681 | 13,14 | -0,1281 | 117,53 | 0,3876 | 13,22 | -0,1219 |
| 117,71 | 0,3645 | 13,18 | -0,1663 | 117,56 | 0,3681 | 13,17 | -0,1281 | 117,5 | 0,3876 | 13,25 | -0,122 |
| 117,68 | 0,3645 | 13,21 | -0,1663 | 117,52 | 0,3681 | 13,21 | -0,1282 | 117,46 | 0,3876 | 13,29 | -0,122 |
| 117,64 | 0,3645 | 13,25 | -0,1664 | 117,49 | 0,3681 | 13,24 | -0,1282 | 117,43 | 0,3876 | 13,32 | -0,122 |
| 117,61 | 0,3645 | 13,28 | -0,1664 | 117,46 | 0,3681 | 13,27 | -0,1283 | 117,4 | 0,3876 | 13,35 | -0,122 |
| 117,58 | 0,3645 | 13,31 | -0,1665 | 117,43 | 0,3681 | 13,31 | -0,1284 | 117,36 | 0,3875 | 13,39 | -0,1221 |
| 117,54 | 0,3645 | 13,35 | -0,1666 | 117,39 | 0,3681 | 13,34 | -0,1284 | 117,33 | 0,3875 | 13,42 | -0,1221 |
| 117,51 | 0,3645 | 13,38 | -0,1666 | 117,36 | 0,368 | 13,37 | -0,1285 | 117,3 | 0,3875 | 13,45 | -0,1221 |
| 117,48 | 0,3646 | 13,41 | -0,1667 | 117,32 | 0,368 | 13,41 | -0,1285 | 117,26 | 0,3875 | 13,49 | -0,1222 |
| 117,44 | 0,3646 | 13,45 | -0,1667 | 117,29 | 0,368 | 13,44 | -0,1286 | 117,23 | 0,3874 | 13,52 | -0,1222 |
| 117,41 | 0,3646 | 13,48 | -0,1668 | 117,26 | 0,368 | 13,47 | -0,1286 | 117,2 | 0,3874 | 13,55 | -0,1222 |
| 117,38 | 0,3646 | 13,51 | -0,1669 | 117,22 | 0,368 | 13,51 | -0,1287 | 117,16 | 0,3874 | 13,59 | -0,1222 |
| 117,34 | 0,3646 | 13,55 | -0,1669 | 117,19 | 0,3679 | 13,54 | -0,1287 | 117,13 | 0,3874 | 13,62 | -0,1223 |
| 117,31 | 0,3646 | 13,58 | -0,167 | 117,16 | 0,3679 | 13,57 | -0,1288 | 117,1 | 0,3873 | 13,65 | -0,1223 |
| 117,28 | 0,3647 | 13,61 | -0,1671 | 117,12 | 0,3679 | 13,61 | -0,1289 | 117,06 | 0,3873 | 13,69 | -0,1223 |
| 117,24 | 0,3647 | 13,65 | -0,1671 | 117,09 | 0,3679 | 13,64 | -0,1289 | 117,03 | 0,3873 | 13,72 | -0,1223 |
| 117,21 | 0,3647 | 13,68 | -0,1672 | 117,06 | 0,3679 | 13,67 | -0,129 | 117 | 0,3873 | 13,75 | -0,1224 |
| 117,18 | 0,3647 | 13,71 | -0,1673 | 117,02 | 0,3679 | 13,71 | -0,1291 | 116,96 | 0,3872 | 13,79 | -0,1224 |
| 117,14 | 0,3647 | 13,75 | -0,1673 | 116,99 | 0,3678 | 13,74 | -0,1291 | 116,93 | 0,3872 | 13,82 | -0,1224 |
| 117,11 | 0,3647 | 13,78 | -0,1674 | 116,96 | 0,3678 | 13,77 | -0,1292 | 116,9 | 0,3872 | 13,85 | -0,1224 |
| 117,08 | 0,3648 | 13,81 | -0,1674 | 116,92 | 0,3678 | 13,81 | -0,1292 | 116,86 | 0,3872 | 13,89 | -0,1225 |
| 117,04 | 0,3648 | 13,85 | -0,1675 | 116,89 | 0,3678 | 13,84 | -0,1293 | 116,83 | 0,3871 | 13,92 | -0,1225 |
| 117,01 | 0,3648 | 13,88 | -0,1676 | 116,86 | 0,3678 | 13,87 | -0,1294 | 116,8 | 0,3871 | 13,95 | -0,1225 |
| 116,98 | 0,3648 | 13,91 | -0,1676 | 116,83 | 0,3678 | 13,91 | -0,1295 | 116,76 | 0,3871 | 13,99 | -0,1225 |
| 116,94 | 0,3648 | 13,95 | -0,1677 | 116,79 | 0,3677 | 13,94 | -0,1295 | 116,73 | 0,3871 | 14,02 | -0,1226 |
| 116,91 | 0,3648 | 13,98 | -0,1677 | 116,76 | 0,3677 | 13,97 | -0,1296 | 116,7 | 0,387 | 14,05 | -0,1226 |
| 116,88 | 0,3648 | 14,01 | -0,1678 | 116,73 | 0,3677 | 14,01 | -0,1296 | 116,66 | 0,387 | 14,09 | -0,1226 |
| 116,84 | 0,3649 | 14,05 | -0,1678 | 116,69 | 0,3677 | 14,04 | -0,1297 | 116,63 | 0,387 | 14,12 | -0,1226 |
| 116,81 | 0,3649 | 14,08 | -0,1679 | 116,66 | 0,3677 | 14,07 | -0,1297 | 116,6 | 0,387 | 14,15 | -0,1227 |
| 116,78 | 0,3649 | 14,11 | -0,168 | 116,63 | 0,3676 | 14,11 | -0,1298 | 116,56 | 0,387 | 14,19 | -0,1227 |
| 116,74 | 0,3649 | 14,15 | -0,168 | 116,59 | 0,3676 | 14,14 | -0,1299 | 116,53 | 0,3869 | 14,22 | -0,1227 |
| 116,71 | 0,3649 | 14,18 | -0,1681 | 116,56 | 0,3676 | 14,17 | -0,1299 | 116,5 | 0,3869 | 14,25 | -0,1228 |
| 116,68 | 0,3649 | 14,21 | -0,1681 | 116,53 | 0,3676 | 14,21 | -0,13 | 116,46 | 0,3869 | 14,29 | -0,1228 |
| 116,64 | 0,3649 | 14,25 | -0,1682 | 116,49 | 0,3676 | 14,24 | -0,13 | 116,43 | 0,3869 | 14,32 | -0,1228 |
| 116,61 | 0,365 | 14,28 | -0,1683 | 116,46 | 0,3676 | 14,27 | -0,1301 | 116,4 | 0,3868 | 14,35 | -0,1228 |
| 116,58 | 0,365 | 14,31 | -0,1683 | 116,43 | 0,3675 | 14,31 | -0,1302 | 116,36 | 0,3868 | 14,39 | -0,1229 |
| 116,54 | 0,365 | 14,35 | -0,1684 | 116,39 | 0,3675 | 14,34 | -0,1302 | 116,33 | 0,3868 | 14,42 | -0,1229 |
| 116,51 | 0,365 | 14,38 | -0,1685 | 116,36 | 0,3675 | 14,37 | -0,1303 | 116,3 | 0,3868 | 14,45 | -0,1229 |
| 116,48 | 0,365 | 14,41 | -0,1685 | 116,33 | 0,3675 | 14,41 | -0,1303 | 116,26 | 0,3867 | 14,49 | -0,1229 |
| 116,44 | 0,365 | 14,45 | -0,1686 | 116,29 | 0,3675 | 14,44 | -0,1304 | 116,23 | 0,3867 | 14,52 | -0,123 |
| 116,41 | 0,365 | 14,48 | -0,1687 | 116,26 | 0,3674 | 14,47 | -0,1304 | 116,2 | 0,3867 | 14,55 | -0,123 |
| 116,38 | 0,365 | 14,51 | -0,1687 | 116,22 | 0,3674 | 14,51 | -0,1305 | 116,17 | 0,3867 | 14,58 | -0,123 |
| 116,34 | 0,3651 | 14,55 | -0,1688 | 116,19 | 0,3674 | 14,54 | -0,1306 | 116,13 | 0,3866 | 14,62 | -0,123 |
| 116,31 | 0,3651 | 14,58 | -0,1689 | 116,16 | 0,3674 | 14,57 | -0,1307 | 116,1 | 0,3866 | 14,65 | -0,1231 |
| 116,28 | 0,3651 | 14,61 | -0,169 | 116,13 | 0,3674 | 14,61 | -0,1307 | 116,06 | 0,3866 | 14,69 | -0,1231 |
| 116,24 | 0,3651 | 14,65 | -0,1691 | 116,09 | 0,3673 | 14,64 | -0,1308 | 116,03 | 0,3866 | 14,72 | -0,1231 |
| 116,21 | 0,3651 | 14,68 | -0,1692 | 116,06 | 0,3673 | 14,67 | -0,1308 | 116 | 0,3865 | 14,75 | -0,1231 |
| 116,18 | 0,3651 | 14,71 | -0,1692 | 116,03 | 0,3673 | 14,7 | -0,1309 | 115,96 | 0,3865 | 14,79 | -0,1232 |
| 116,15 | 0,3651 | 14,74 | -0,1693 | 115,99 | 0,3673 | 14,74 | -0,131 | 115,93 | 0,3865 | 14,82 | -0,1232 |
| 116,11 | 0,3651 | 14,78 | -0,1693 | 115,96 | 0,3673 | 14,77 | -0,131 | 115,9 | 0,3864 | 14,85 | -0,1232 |
| 116,08 | 0,3652 | 14,81 | -0,1694 | 115,93 | 0,3673 | 14,81 | -0,1311 | 115,86 | 0,3864 | 14,89 | -0,1233 |
| 116,04 | 0,3652 | 14,85 | -0,1695 | 115,89 | 0,3672 | 14,84 | -0,1311 | 115,83 | 0,3864 | 14,92 | -0,1233 |
| 116,01 | 0,3652 | 14,88 | -0,1695 | 115,86 | 0,3672 | 14,87 | -0,1312 | 115,8 | 0,3864 | 14,95 | -0,1233 |
| 115,98 | 0,3652 | 14,91 | -0,1696 | 115,83 | 0,3672 | 14,91 | -0,1313 | 115,77 | 0,3863 | 14,99 | -0,1233 |
| 115,94 | 0,3652 | 14,94 | -0,1696 | 115,79 | 0,3672 | 14,94 | -0,1313 | 115,73 | 0,3863 | 15,02 | -0,1234 |
| 115,91 | 0,3652 | 14,98 | -0,1697 | 115,76 | 0,3672 | 14,97 | -0,1314 | 115,7 | 0,3863 | 15,05 | -0,1234 |
| 115,88 | 0,3652 | 15,01 | -0,1698 | 115,73 | 0,3671 | 15,01 | -0,1314 | 115,67 | 0,3863 | 15,09 | -0,1234 |
| 115,85 | 0,3652 | 15,05 | -0,1699 | 115,69 | 0,3671 | 15,04 | -0,1315 | 115,63 | 0,3863 | 15,12 | -0,1234 |
| 115,81 | 0,3653 | 15,08 | -0,17 | 115,66 | 0,3671 | 15,07 | -0,1315 | 115,6 | 0,3862 | 15,15 | -0,1235 |
| 115,78 | 0,3653 | 15,11 | -0,17 | 115,63 | 0,3671 | 15,11 | -0,1316 | 115,57 | 0,3862 | 15,19 | -0,1235 |
| 115,75 | 0,3653 | 15,15 | -0,1701 | 115,59 | 0,3671 | 15,14 | -0,1317 | 115,53 | 0,3862 | 15,22 | -0,1235 |
| 115,71 | 0,3653 | 15,18 | -0,1701 | 115,56 | 0,367 | 15,17 | -0,1317 | 115,5 | 0,3861 | 15,25 | -0,1235 |
| 115,68 | 0,3653 | 15,21 | -0,1702 | 115,53 | 0,367 | 15,21 | -0,1318 | 115,47 | 0,3861 | 15,29 | -0,1236 |
| 115,64 | 0,3653 | 15,25 | -0,1703 | 115,49 | 0,367 | 15,24 | -0,1319 | 115,43 | 0,3861 | 15,32 | -0,1236 |
| 115,61 | 0,3653 | 15,28 | -0,1704 | 115,46 | 0,367 | 15,27 | -0,1319 | 115,4 | 0,3861 | 15,35 | -0,1236 |
| 115,58 | 0,3653 | 15,31 | -0,1705 | 115,43 | 0,367 | 15,3 | -0,132 | 115,37 | 0,386 | 15,39 | -0,1236 |
| 115,55 | 0,3653 | 15,34 | -0,1706 | 115,39 | 0,3669 | 15,34 | -0,132 | 115,33 | 0,386 | 15,42 | -0,1237 |
| 115,51 | 0,3654 | 15,38 | -0,1707 | 115,36 | 0,3669 | 15,37 | -0,1321 | 115,3 | 0,386 | 15,45 | -0,1237 |
| 115,48 | 0,3654 | 15,41 | -0,1708 | 115,33 | 0,3669 | 15,41 | -0,1322 | 115,27 | 0,386 | 15,49 | -0,1237 |
| 115,45 | 0,3654 | 15,45 | -0,1708 | 115,29 | 0,3669 | 15,44 | -0,1323 | 115,23 | 0,3859 | 15,52 | -0,1237 |
| 115,41 | 0,3654 | 15,48 | -0,1709 | 115,26 | 0,3669 | 15,47 | -0,1323 | 115,2 | 0,3859 | 15,55 | -0,1238 |
| 115,38 | 0,3654 | 15,51 | -0,171 | 115,23 | 0,3668 | 15,5 | -0,1324 | 115,17 | 0,3859 | 15,59 | -0,1238 |
| 115,34 | 0,3654 | 15,54 | -0,171 | 115,19 | 0,3668 | 15,54 | -0,1325 | 115,13 | 0,3859 | 15,62 | -0,1238 |
| 115,31 | 0,3654 | 15,58 | -0,1711 | 115,16 | 0,3668 | 15,57 | -0,1325 | 115,1 | 0,3858 | 15,65 | -0,1238 |
| 115,28 | 0,3654 | 15,61 | -0,1712 | 115,13 | 0,3668 | 15,61 | -0,1326 | 115,07 | 0,3858 | 15,69 | -0,1239 |
| 115,25 | 0,3654 | 15,64 | -0,1712 | 115,09 | 0,3668 | 15,64 | -0,1327 | 115,03 | 0,3858 | 15,72 | -0,1239 |
| 115,21 | 0,3655 | 15,68 | -0,1713 | 115,06 | 0,3668 | 15,67 | -0,1328 | 115 | 0,3858 | 15,75 | -0,1239 |
| 115,18 | 0,3655 | 15,71 | -0,1714 | 115,03 | 0,3667 | 15,7 | -0,1328 | 114,97 | 0,3857 | 15,79 | -0,124 |
| 115,15 | 0,3655 | 15,74 | -0,1714 | 114,99 | 0,3667 | 15,74 | -0,1329 | 114,93 | 0,3857 | 15,82 | -0,124 |
| 115,11 | 0,3655 | 15,78 | -0,1715 | 114,96 | 0,3667 | 15,77 | -0,133 | 114,9 | 0,3857 | 15,85 | -0,124 |
| 115,08 | 0,3655 | 15,81 | -0,1716 | 114,93 | 0,3667 | 15,8 | -0,133 | 114,87 | 0,3856 | 15,88 | -0,124 |
| 115,05 | 0,3655 | 15,84 | -0,1716 | 114,89 | 0,3667 | 15,84 | -0,1331 | 114,83 | 0,3856 | 15,92 | -0,1241 |
| 115,01 | 0,3655 | 15,88 | -0,1717 | 114,86 | 0,3666 | 15,87 | -0,1332 | 114,8 | 0,3856 | 15,95 | -0,1241 |
| 114,98 | 0,3655 | 15,91 | -0,1718 | 114,83 | 0,3666 | 15,9 | -0,1332 | 114,77 | 0,3856 | 15,99 | -0,1241 |
| 114,95 | 0,3655 | 15,94 | -0,1718 | 114,79 | 0,3666 | 15,94 | -0,1333 | 114,73 | 0,3855 | 16,02 | -0,1241 |
| 114,91 | 0,3655 | 15,98 | -0,1719 | 114,76 | 0,3666 | 15,97 | -0,1333 | 114,7 | 0,3855 | 16,05 | -0,1242 |
| 114,88 | 0,3656 | 16,01 | -0,1721 | 114,73 | 0,3665 | 16 | -0,1334 | 114,67 | 0,3855 | 16,09 | -0,1242 |
| 114,85 | 0,3656 | 16,04 | -0,1722 | 114,69 | 0,3665 | 16,04 | -0,1335 | 114,63 | 0,3855 | 16,12 | -0,1242 |
| 114,81 | 0,3656 | 16,08 | -0,1722 | 114,66 | 0,3665 | 16,07 | -0,1335 | 114,6 | 0,3854 | 16,15 | -0,1242 |
| 114,78 | 0,3656 | 16,11 | -0,1723 | 114,63 | 0,3665 | 16,1 | -0,1336 | 114,57 | 0,3854 | 16,19 | -0,1243 |
| 114,75 | 0,3656 | 16,14 | -0,1723 | 114,59 | 0,3665 | 16,14 | -0,1336 | 114,53 | 0,3854 | 16,22 | -0,1243 |
| 114,71 | 0,3656 | 16,18 | -0,1724 | 114,56 | 0,3665 | 16,17 | -0,1337 | 114,5 | 0,3854 | 16,25 | -0,1243 |
| 114,68 | 0,3656 | 16,21 | -0,1725 | 114,53 | 0,3664 | 16,2 | -0,1338 | 114,47 | 0,3853 | 16,28 | -0,1243 |
| 114,64 | 0,3656 | 16,24 | -0,1726 | 114,49 | 0,3664 | 16,24 | -0,1338 | 114,43 | 0,3853 | 16,32 | -0,1244 |
| 114,61 | 0,3656 | 16,28 | -0,1727 | 114,46 | 0,3664 | 16,27 | -0,1339 | 114,4 | 0,3853 | 16,35 | -0,1244 |
| 114,58 | 0,3656 | 16,31 | -0,1728 | 114,43 | 0,3664 | 16,3 | -0,134 | 114,37 | 0,3852 | 16,39 | -0,1244 |
| 114,55 | 0,3656 | 16,34 | -0,1729 | 114,39 | 0,3663 | 16,34 | -0,134 | 114,34 | 0,3852 | 16,42 | -0,1245 |
| 114,51 | 0,3657 | 16,38 | -0,173 | 114,36 | 0,3663 | 16,37 | -0,1341 | 114,3 | 0,3852 | 16,45 | -0,1245 |
| 114,48 | 0,3657 | 16,41 | -0,173 | 114,33 | 0,3663 | 16,4 | -0,1341 | 114,27 | 0,3852 | 16,49 | -0,1245 |
| 114,45 | 0,3657 | 16,44 | -0,1731 | 114,29 | 0,3663 | 16,44 | -0,1342 | 114,23 | 0,3851 | 16,52 | -0,1245 |
| 114,41 | 0,3657 | 16,48 | -0,1732 | 114,26 | 0,3663 | 16,47 | -0,1343 | 114,2 | 0,3851 | 16,55 | -0,1245 |
| 114,38 | 0,3657 | 16,51 | -0,1733 | 114,23 | 0,3663 | 16,5 | -0,1343 | 114,17 | 0,3851 | 16,59 | -0,1246 |
| 114,34 | 0,3657 | 16,54 | -0,1734 | 114,19 | 0,3662 | 16,54 | -0,1344 | 114,14 | 0,3851 | 16,62 | -0,1246 |
| 114,31 | 0,3657 | 16,58 | -0,1735 | 114,16 | 0,3662 | 16,57 | -0,1344 | 114,1 | 0,385 | 16,65 | -0,1246 |
| 114,28 | 0,3657 | 16,61 | -0,1735 | 114,13 | 0,3662 | 16,6 | -0,1345 | 114,07 | 0,385 | 16,69 | -0,1247 |
| 114,25 | 0,3657 | 16,64 | -0,1736 | 114,09 | 0,3662 | 16,64 | -0,1346 | 114,04 | 0,385 | 16,72 | -0,1247 |
| 114,21 | 0,3657 | 16,68 | -0,1737 | 114,06 | 0,3661 | 16,67 | -0,1347 | 114 | 0,385 | 16,75 | -0,1247 |
| 114,18 | 0,3657 | 16,71 | -0,1737 | 114,03 | 0,3661 | 16,7 | -0,1347 | 113,97 | 0,3849 | 16,79 | -0,1247 |
| 114,15 | 0,3657 | 16,74 | -0,1738 | 113,99 | 0,3661 | 16,74 | -0,1348 | 113,94 | 0,3849 | 16,82 | -0,1248 |
| 114,11 | 0,3658 | 16,78 | -0,1739 | 113,96 | 0,3661 | 16,77 | -0,1349 | 113,9 | 0,3849 | 16,85 | -0,1248 |
| 114,08 | 0,3658 | 16,81 | -0,174 | 113,93 | 0,3661 | 16,8 | -0,1349 | 113,87 | 0,3849 | 16,89 | -0,1248 |
| 114,05 | 0,3658 | 16,84 | -0,1741 | 113,89 | 0,366 | 16,84 | -0,135 | 113,84 | 0,3848 | 16,92 | -0,1248 |
| 114,01 | 0,3658 | 16,88 | -0,1742 | 113,86 | 0,366 | 16,87 | -0,135 | 113,8 | 0,3848 | 16,95 | -0,1249 |
| 113,98 | 0,3658 | 16,91 | -0,1742 | 113,83 | 0,366 | 16,9 | -0,1351 | 113,77 | 0,3848 | 16,99 | -0,1249 |
| 113,95 | 0,3658 | 16,94 | -0,1743 | 113,79 | 0,366 | 16,94 | -0,1352 | 113,74 | 0,3847 | 17,02 | -0,1249 |
| 113,91 | 0,3658 | 16,98 | -0,1744 | 113,76 | 0,366 | 16,97 | -0,1352 | 113,7 | 0,3847 | 17,05 | -0,125 |
| 113,88 | 0,3658 | 17,01 | -0,1744 | 113,73 | 0,3659 | 17 | -0,1353 | 113,67 | 0,3847 | 17,09 | -0,125 |
| 113,85 | 0,3658 | 17,04 | -0,1745 | 113,69 | 0,3659 | 17,04 | -0,1353 | 113,64 | 0,3847 | 17,12 | -0,125 |
| 113,81 | 0,3658 | 17,08 | -0,1746 | 113,66 | 0,3659 | 17,07 | -0,1354 | 113,6 | 0,3846 | 17,15 | -0,125 |
| 113,78 | 0,3658 | 17,11 | -0,1746 | 113,63 | 0,3659 | 17,1 | -0,1355 | 113,57 | 0,3846 | 17,19 | -0,125 |
| 113,75 | 0,3658 | 17,14 | -0,1747 | 113,59 | 0,3659 | 17,14 | -0,1356 | 113,54 | 0,3846 | 17,22 | -0,1251 |
| 113,71 | 0,3658 | 17,18 | -0,1748 | 113,56 | 0,3658 | 17,17 | -0,1357 | 113,5 | 0,3846 | 17,25 | -0,1251 |
| 113,68 | 0,3658 | 17,21 | -0,1749 | 113,53 | 0,3658 | 17,2 | -0,1358 | 113,47 | 0,3845 | 17,29 | -0,1251 |
| 113,65 | 0,3658 | 17,24 | -0,175 | 113,49 | 0,3658 | 17,24 | -0,1358 | 113,44 | 0,3845 | 17,32 | -0,1252 |
| 113,61 | 0,3658 | 17,28 | -0,1751 | 113,46 | 0,3658 | 17,27 | -0,1359 | 113,4 | 0,3845 | 17,35 | -0,1252 |
| 113,58 | 0,3659 | 17,31 | -0,1752 | 113,43 | 0,3658 | 17,3 | -0,1359 | 113,37 | 0,3845 | 17,39 | -0,1252 |
| 113,54 | 0,3659 | 17,34 | -0,1753 | 113,39 | 0,3657 | 17,34 | -0,1361 | 113,34 | 0,3844 | 17,42 | -0,1252 |
| 113,51 | 0,3659 | 17,38 | -0,1753 | 113,36 | 0,3657 | 17,37 | -0,1362 | 113,3 | 0,3844 | 17,45 | -0,1253 |
| 113,48 | 0,3659 | 17,41 | -0,1754 | 113,33 | 0,3657 | 17,4 | -0,1363 | 113,27 | 0,3844 | 17,49 | -0,1253 |
| 113,45 | 0,3659 | 17,44 | -0,1755 | 113,29 | 0,3657 | 17,44 | -0,1364 | 113,24 | 0,3844 | 17,52 | -0,1253 |
| 113,41 | 0,3659 | 17,48 | -0,1756 | 113,26 | 0,3657 | 17,47 | -0,1364 | 113,2 | 0,3843 | 17,55 | -0,1253 |
| 113,38 | 0,3659 | 17,51 | -0,1757 | 113,23 | 0,3656 | 17,5 | -0,1365 | 113,17 | 0,3843 | 17,59 | -0,1254 |
| 113,35 | 0,3659 | 17,54 | -0,1759 | 113,19 | 0,3656 | 17,53 | -0,1365 | 113,14 | 0,3843 | 17,62 | -0,1254 |
| 113,31 | 0,3659 | 17,58 | -0,1761 | 113,16 | 0,3656 | 17,57 | -0,1366 | 113,1 | 0,3842 | 17,65 | -0,1254 |
| 113,28 | 0,3659 | 17,61 | -0,1762 | 113,13 | 0,3656 | 17,6 | -0,1367 | 113,07 | 0,3842 | 17,69 | -0,1254 |
| 113,25 | 0,3659 | 17,64 | -0,1763 | 113,1 | 0,3656 | 17,63 | -0,1367 | 113,04 | 0,3842 | 17,72 | -0,1255 |
| 113,21 | 0,3659 | 17,68 | -0,1764 | 113,06 | 0,3655 | 17,67 | -0,1368 | 113 | 0,3842 | 17,75 | -0,1255 |
| 113,18 | 0,3659 | 17,71 | -0,1765 | 113,03 | 0,3655 | 17,7 | -0,1369 | 112,97 | 0,3841 | 17,79 | -0,1255 |
| 113,15 | 0,3659 | 17,74 | -0,1765 | 112,99 | 0,3655 | 17,74 | -0,1369 | 112,94 | 0,3841 | 17,82 | -0,1255 |
| 113,11 | 0,3659 | 17,78 | -0,1766 | 112,96 | 0,3655 | 17,77 | -0,137 | 112,9 | 0,3841 | 17,85 | -0,1256 |
| 113,08 | 0,3659 | 17,81 | -0,1767 | 112,93 | 0,3654 | 17,8 | -0,1371 | 112,87 | 0,3841 | 17,89 | -0,1256 |
| 113,05 | 0,3659 | 17,84 | -0,1768 | 112,89 | 0,3654 | 17,84 | -0,1372 | 112,84 | 0,384 | 17,92 | -0,1256 |
| 113,01 | 0,3659 | 17,88 | -0,1769 | 112,86 | 0,3654 | 17,87 | -0,1372 | 112,8 | 0,384 | 17,95 | -0,1257 |
| 112,98 | 0,366 | 17,91 | -0,1769 | 112,83 | 0,3654 | 17,9 | -0,1373 | 112,77 | 0,384 | 17,99 | -0,1257 |
| 112,95 | 0,366 | 17,94 | -0,177 | 112,79 | 0,3654 | 17,94 | -0,1374 | 112,74 | 0,3839 | 18,02 | -0,1257 |
| 112,91 | 0,366 | 17,97 | -0,1771 | 112,76 | 0,3653 | 17,97 | -0,1374 | 112,7 | 0,3839 | 18,05 | -0,1257 |
| 112,88 | 0,366 | 18,01 | -0,1772 | 112,73 | 0,3653 | 18 | -0,1375 | 112,67 | 0,3839 | 18,09 | -0,1258 |
| 112,85 | 0,366 | 18,04 | -0,1773 | 112,69 | 0,3653 | 18,04 | -0,1375 | 112,64 | 0,3839 | 18,12 | -0,1258 |
| 112,81 | 0,366 | 18,08 | -0,1774 | 112,66 | 0,3653 | 18,07 | -0,1376 | 112,6 | 0,3839 | 18,15 | -0,1258 |
| 112,78 | 0,366 | 18,11 | -0,1774 | 112,63 | 0,3653 | 18,1 | -0,1377 | 112,57 | 0,3838 | 18,19 | -0,1258 |
| 112,75 | 0,366 | 18,14 | -0,1775 | 112,59 | 0,3652 | 18,14 | -0,1378 | 112,54 | 0,3838 | 18,22 | -0,1259 |
| 112,71 | 0,366 | 18,18 | -0,1776 | 112,56 | 0,3652 | 18,17 | -0,1379 | 112,5 | 0,3838 | 18,26 | -0,1259 |
| 112,68 | 0,366 | 18,21 | -0,1777 | 112,53 | 0,3652 | 18,2 | -0,138 | 112,47 | 0,3837 | 18,29 | -0,1259 |
| 112,65 | 0,366 | 18,24 | -0,1778 | 112,49 | 0,3652 | 18,24 | -0,138 | 112,44 | 0,3837 | 18,32 | -0,1259 |
| 112,61 | 0,366 | 18,27 | -0,178 | 112,46 | 0,3652 | 18,27 | -0,1381 | 112,4 | 0,3837 | 18,35 | -0,126 |
| 112,58 | 0,366 | 18,31 | -0,1781 | 112,43 | 0,3651 | 18,3 | -0,1382 | 112,37 | 0,3837 | 18,39 | -0,126 |
| 112,55 | 0,366 | 18,34 | -0,1781 | 112,39 | 0,3651 | 18,34 | -0,1382 | 112,34 | 0,3836 | 18,42 | -0,126 |
| 112,51 | 0,366 | 18,37 | -0,1782 | 112,36 | 0,3651 | 18,37 | -0,1383 | 112,3 | 0,3836 | 18,45 | -0,126 |
| 112,48 | 0,366 | 18,41 | -0,1783 | 112,33 | 0,3651 | 18,4 | -0,1384 | 112,27 | 0,3836 | 18,49 | -0,1261 |
| 112,45 | 0,366 | 18,44 | -0,1784 | 112,29 | 0,3651 | 18,43 | -0,1384 | 112,24 | 0,3835 | 18,52 | -0,1261 |
| 112,41 | 0,366 | 18,48 | -0,1785 | 112,26 | 0,365 | 18,47 | -0,1385 | 112,2 | 0,3835 | 18,56 | -0,1261 |
| 112,38 | 0,366 | 18,51 | -0,1786 | 112,23 | 0,365 | 18,5 | -0,1386 | 112,17 | 0,3835 | 18,59 | -0,1262 |
| 112,35 | 0,366 | 18,54 | -0,1787 | 112,2 | 0,365 | 18,53 | -0,1386 | 112,14 | 0,3835 | 18,62 | -0,1262 |
| 112,31 | 0,366 | 18,57 | -0,1788 | 112,16 | 0,365 | 18,57 | -0,1387 | 112,1 | 0,3834 | 18,65 | -0,1262 |
| 112,28 | 0,366 | 18,61 | -0,1789 | 112,13 | 0,3649 | 18,6 | -0,1388 | 112,07 | 0,3834 | 18,69 | -0,1262 |
| 112,25 | 0,366 | 18,64 | -0,179 | 112,09 | 0,3649 | 18,64 | -0,139 | 112,04 | 0,3834 | 18,72 | -0,1263 |
| 112,21 | 0,3661 | 18,67 | -0,1791 | 112,06 | 0,3649 | 18,67 | -0,139 | 112 | 0,3834 | 18,75 | -0,1263 |
| 112,18 | 0,3661 | 18,71 | -0,1792 | 112,03 | 0,3649 | 18,7 | -0,1391 | 111,97 | 0,3833 | 18,79 | -0,1263 |
| 112,15 | 0,3661 | 18,74 | -0,1793 | 112 | 0,3649 | 18,74 | -0,1392 | 111,94 | 0,3833 | 18,82 | -0,1264 |
| 112,11 | 0,3661 | 18,77 | -0,1794 | 111,96 | 0,3648 | 18,77 | -0,1393 | 111,9 | 0,3833 | 18,85 | -0,1264 |
| 112,08 | 0,3661 | 18,81 | -0,1795 | 111,93 | 0,3648 | 18,8 | -0,1393 | 111,87 | 0,3833 | 18,89 | -0,1264 |
| 112,05 | 0,3661 | 18,84 | -0,1796 | 111,89 | 0,3648 | 18,84 | -0,1394 | 111,84 | 0,3832 | 18,92 | -0,1264 |
| 112,01 | 0,3661 | 18,87 | -0,1796 | 111,86 | 0,3648 | 18,87 | -0,1395 | 111,8 | 0,3832 | 18,95 | -0,1264 |
| 111,98 | 0,3661 | 18,91 | -0,1797 | 111,83 | 0,3648 | 18,9 | -0,1395 | 111,77 | 0,3832 | 18,99 | -0,1265 |
| 111,95 | 0,3661 | 18,94 | -0,1798 | 111,79 | 0,3647 | 18,94 | -0,1396 | 111,74 | 0,3832 | 19,02 | -0,1265 |
| 111,91 | 0,3661 | 18,97 | -0,1799 | 111,76 | 0,3647 | 18,97 | -0,1397 | 111,7 | 0,3831 | 19,05 | -0,1265 |
| 111,88 | 0,3661 | 19,01 | -0,18 | 111,73 | 0,3647 | 19 | -0,1397 | 111,67 | 0,3831 | 19,09 | -0,1266 |
| 111,85 | 0,3661 | 19,04 | -0,1801 | 111,7 | 0,3647 | 19,03 | -0,1398 | 111,64 | 0,3831 | 19,12 | -0,1266 |
| 111,81 | 0,3661 | 19,07 | -0,1803 | 111,66 | 0,3647 | 19,07 | -0,1399 | 111,6 | 0,383 | 19,15 | -0,1266 |
| 111,78 | 0,3661 | 19,11 | -0,1804 | 111,63 | 0,3646 | 19,1 | -0,1399 | 111,57 | 0,383 | 19,19 | -0,1266 |
| 111,75 | 0,3661 | 19,14 | -0,1805 | 111,59 | 0,3646 | 19,13 | -0,14 | 111,54 | 0,383 | 19,22 | -0,1267 |
| 111,71 | 0,3661 | 19,17 | -0,1806 | 111,56 | 0,3646 | 19,17 | -0,1401 | 111,5 | 0,383 | 19,25 | -0,1267 |
| 111,68 | 0,3661 | 19,21 | -0,1808 | 111,53 | 0,3646 | 19,2 | -0,1402 | 111,47 | 0,3829 | 19,29 | -0,1267 |
| 111,65 | 0,3661 | 19,24 | -0,1809 | 111,5 | 0,3645 | 19,23 | -0,1404 | 111,44 | 0,3829 | 19,32 | -0,1267 |
| 111,61 | 0,3661 | 19,27 | -0,181 | 111,46 | 0,3645 | 19,27 | -0,1405 | 111,4 | 0,3829 | 19,36 | -0,1268 |
| 111,58 | 0,3661 | 19,31 | -0,1811 | 111,43 | 0,3645 | 19,3 | -0,1406 | 111,37 | 0,3829 | 19,39 | -0,1268 |
| 111,55 | 0,3661 | 19,34 | -0,1812 | 111,39 | 0,3645 | 19,34 | -0,1407 | 111,34 | 0,3828 | 19,42 | -0,1268 |
| 111,51 | 0,3661 | 19,37 | -0,1813 | 111,36 | 0,3645 | 19,37 | -0,1407 | 111,3 | 0,3828 | 19,45 | -0,1269 |
| 111,48 | 0,3661 | 19,41 | -0,1814 | 111,33 | 0,3644 | 19,4 | -0,1408 | 111,27 | 0,3828 | 19,49 | -0,1269 |
| 111,45 | 0,3661 | 19,44 | -0,1815 | 111,3 | 0,3644 | 19,43 | -0,1409 | 111,24 | 0,3828 | 19,52 | -0,1269 |
| 111,41 | 0,3661 | 19,47 | -0,1816 | 111,26 | 0,3644 | 19,47 | -0,141 | 111,2 | 0,3827 | 19,55 | -0,1269 |
| 111,38 | 0,3661 | 19,51 | -0,1817 | 111,23 | 0,3644 | 19,5 | -0,1411 | 111,17 | 0,3827 | 19,59 | -0,127 |
| 111,35 | 0,3661 | 19,54 | -0,1818 | 111,19 | 0,3644 | 19,53 | -0,1412 | 111,14 | 0,3827 | 19,62 | -0,127 |
| 111,31 | 0,3661 | 19,57 | -0,1818 | 111,16 | 0,3643 | 19,57 | -0,1413 | 111,1 | 0,3827 | 19,65 | -0,127 |
| 111,28 | 0,3661 | 19,61 | -0,182 | 111,13 | 0,3643 | 19,6 | -0,1413 | 111,07 | 0,3826 | 19,69 | -0,127 |
| 111,25 | 0,3661 | 19,64 | -0,1821 | 111,09 | 0,3643 | 19,63 | -0,1414 | 111,04 | 0,3826 | 19,72 | -0,1271 |
| 111,21 | 0,3661 | 19,67 | -0,1823 | 111,06 | 0,3643 | 19,67 | -0,1414 | 111 | 0,3826 | 19,75 | -0,1271 |
| 111,18 | 0,3661 | 19,71 | -0,1824 | 111,03 | 0,3643 | 19,7 | -0,1415 | 110,97 | 0,3825 | 19,79 | -0,1271 |
| 111,15 | 0,3661 | 19,74 | -0,1825 | 110,99 | 0,3642 | 19,74 | -0,1417 | 110,94 | 0,3825 | 19,82 | -0,1271 |
| 111,11 | 0,3661 | 19,77 | -0,1826 | 110,96 | 0,3642 | 19,77 | -0,1418 | 110,9 | 0,3825 | 19,86 | -0,1272 |
| 111,08 | 0,3661 | 19,81 | -0,1827 | 110,93 | 0,3642 | 19,8 | -0,1419 | 110,87 | 0,3825 | 19,89 | -0,1272 |
| 111,05 | 0,3661 | 19,84 | -0,1828 | 110,9 | 0,3642 | 19,83 | -0,142 | 110,84 | 0,3824 | 19,92 | -0,1272 |
| 111,01 | 0,3661 | 19,87 | -0,1829 | 110,86 | 0,3641 | 19,87 | -0,1421 | 110,8 | 0,3824 | 19,95 | -0,1273 |
| 110,98 | 0,3661 | 19,91 | -0,1829 | 110,83 | 0,3641 | 19,9 | -0,1421 | 110,77 | 0,3824 | 19,99 | -0,1273 |
| 110,95 | 0,3661 | 19,94 | -0,1831 | 110,79 | 0,3641 | 19,93 | -0,1422 | 110,74 | 0,3824 | 20,02 | -0,1273 |
| 110,91 | 0,3661 | 19,97 | -0,1832 | 110,76 | 0,3641 | 19,97 | -0,1423 | 110,7 | 0,3823 | 20,05 | -0,1273 |
| 110,88 | 0,3661 | 20 | -0,1834 | 110,73 | 0,3641 | 20 | -0,1423 | 110,67 | 0,3823 | 20,09 | -0,1274 |
| 110,85 | 0,3661 | 20,04 | -0,1835 | 110,7 | 0,364 | 20,03 | -0,1424 | 110,64 | 0,3823 | 20,12 | -0,1274 |
| 110,81 | 0,3661 | 20,07 | -0,1836 | 110,66 | 0,364 | 20,07 | -0,1425 | 110,6 | 0,3823 | 20,15 | -0,1274 |
| 110,78 | 0,3661 | 20,1 | -0,1837 | 110,63 | 0,364 | 20,1 | -0,1427 | 110,57 | 0,3822 | 20,19 | -0,1274 |
| 110,75 | 0,3661 | 20,14 | -0,1839 | 110,6 | 0,364 | 20,13 | -0,1429 | 110,54 | 0,3822 | 20,22 | -0,1275 |
| 110,71 | 0,3661 | 20,17 | -0,184 | 110,56 | 0,364 | 20,17 | -0,143 | 110,5 | 0,3822 | 20,25 | -0,1275 |
| 110,68 | 0,3661 | 20,21 | -0,1842 | 110,53 | 0,3639 | 20,2 | -0,143 | 110,47 | 0,3821 | 20,29 | -0,1275 |
| 110,65 | 0,3661 | 20,24 | -0,1843 | 110,5 | 0,3639 | 20,23 | -0,1431 | 110,44 | 0,3821 | 20,32 | -0,1276 |
| 110,62 | 0,3661 | 20,27 | -0,1844 | 110,46 | 0,3639 | 20,27 | -0,1432 | 110,4 | 0,3821 | 20,36 | -0,1276 |
| 110,58 | 0,3661 | 20,31 | -0,1846 | 110,43 | 0,3639 | 20,3 | -0,1433 | 110,37 | 0,3821 | 20,39 | -0,1276 |
| 110,55 | 0,3661 | 20,34 | -0,1846 | 110,39 | 0,3639 | 20,33 | -0,1434 | 110,34 | 0,382 | 20,42 | -0,1276 |
| 110,52 | 0,3661 | 20,37 | -0,1847 | 110,36 | 0,3638 | 20,37 | -0,1434 | 110,3 | 0,382 | 20,45 | -0,1277 |
| 110,48 | 0,3661 | 20,4 | -0,1848 | 110,33 | 0,3638 | 20,4 | -0,1435 | 110,27 | 0,382 | 20,49 | -0,1277 |
| 110,45 | 0,3661 | 20,44 | -0,1849 | 110,29 | 0,3638 | 20,43 | -0,1436 | 110,24 | 0,382 | 20,52 | -0,1277 |
| 110,41 | 0,3661 | 20,47 | -0,185 | 110,26 | 0,3638 | 20,47 | -0,1437 | 110,2 | 0,3819 | 20,55 | -0,1277 |
| 110,38 | 0,3661 | 20,51 | -0,1851 | 110,23 | 0,3638 | 20,5 | -0,1438 | 110,17 | 0,3819 | 20,59 | -0,1278 |
| 110,35 | 0,3661 | 20,54 | -0,1853 | 110,19 | 0,3637 | 20,53 | -0,1439 | 110,14 | 0,3819 | 20,62 | -0,1278 |
| 110,31 | 0,3661 | 20,57 | -0,1854 | 110,16 | 0,3637 | 20,57 | -0,144 | 110,1 | 0,3819 | 20,65 | -0,1278 |
| 110,28 | 0,3661 | 20,6 | -0,1854 | 110,13 | 0,3637 | 20,6 | -0,144 | 110,07 | 0,3818 | 20,69 | -0,1278 |
| 110,25 | 0,3661 | 20,64 | -0,1855 | 110,09 | 0,3637 | 20,63 | -0,1441 | 110,04 | 0,3818 | 20,72 | -0,1279 |
| 110,21 | 0,3661 | 20,67 | -0,1857 | 110,06 | 0,3636 | 20,67 | -0,1441 | 110 | 0,3818 | 20,75 | -0,1279 |
| 110,18 | 0,3661 | 20,71 | -0,1858 | 110,03 | 0,3636 | 20,7 | -0,1442 | 109,97 | 0,3818 | 20,79 | -0,1279 |
| 110,15 | 0,3661 | 20,74 | -0,1859 | 109,99 | 0,3636 | 20,73 | -0,1444 | 109,94 | 0,3817 | 20,82 | -0,128 |
| 110,12 | 0,3661 | 20,77 | -0,186 | 109,96 | 0,3636 | 20,77 | -0,1445 | 109,9 | 0,3817 | 20,86 | -0,128 |
| 110,08 | 0,3661 | 20,8 | -0,1861 | 109,93 | 0,3636 | 20,8 | -0,1446 | 109,87 | 0,3817 | 20,89 | -0,128 |
| 110,05 | 0,3661 | 20,84 | -0,1862 | 109,89 | 0,3635 | 20,83 | -0,1447 | 109,84 | 0,3816 | 20,92 | -0,128 |
| 110,02 | 0,3661 | 20,87 | -0,1864 | 109,86 | 0,3635 | 20,87 | -0,1448 | 109,8 | 0,3816 | 20,95 | -0,1281 |
| 109,98 | 0,3661 | 20,9 | -0,1865 | 109,83 | 0,3635 | 20,9 | -0,1449 | 109,77 | 0,3816 | 20,99 | -0,1281 |
| 109,95 | 0,3661 | 20,94 | -0,1866 | 109,79 | 0,3635 | 20,93 | -0,1449 | 109,74 | 0,3816 | 21,02 | -0,1281 |
| 109,91 | 0,3661 | 20,97 | -0,1867 | 109,76 | 0,3635 | 20,97 | -0,145 | 109,7 | 0,3816 | 21,05 | -0,1281 |
| 109,88 | 0,3661 | 21 | -0,1868 | 109,73 | 0,3634 | 21 | -0,1451 | 109,67 | 0,3815 | 21,09 | -0,1282 |
| 109,85 | 0,3661 | 21,04 | -0,1869 | 109,69 | 0,3634 | 21,03 | -0,1452 | 109,64 | 0,3815 | 21,12 | -0,1282 |
| 109,82 | 0,3661 | 21,07 | -0,1871 | 109,66 | 0,3634 | 21,07 | -0,1453 | 109,6 | 0,3815 | 21,16 | -0,1282 |
| 109,78 | 0,3661 | 21,1 | -0,1872 | 109,63 | 0,3634 | 21,1 | -0,1455 | 109,57 | 0,3815 | 21,19 | -0,1283 |
| 109,75 | 0,3661 | 21,14 | -0,1873 | 109,59 | 0,3634 | 21,13 | -0,1455 | 109,54 | 0,3814 | 21,22 | -0,1283 |
| 109,71 | 0,3661 | 21,17 | -0,1874 | 109,56 | 0,3633 | 21,17 | -0,1456 | 109,5 | 0,3814 | 21,25 | -0,1283 |
| 109,68 | 0,3661 | 21,2 | -0,1876 | 109,53 | 0,3633 | 21,2 | -0,1457 | 109,47 | 0,3814 | 21,29 | -0,1283 |
| 109,65 | 0,3661 | 21,24 | -0,1877 | 109,49 | 0,3633 | 21,23 | -0,1457 | 109,44 | 0,3814 | 21,32 | -0,1284 |
| 109,61 | 0,3661 | 21,27 | -0,1879 | 109,46 | 0,3633 | 21,27 | -0,1458 | 109,4 | 0,3813 | 21,35 | -0,1284 |
| 109,58 | 0,3661 | 21,3 | -0,188 | 109,43 | 0,3633 | 21,3 | -0,1459 | 109,37 | 0,3813 | 21,39 | -0,1284 |
| 109,55 | 0,3661 | 21,34 | -0,1881 | 109,39 | 0,3632 | 21,33 | -0,146 | 109,34 | 0,3813 | 21,42 | -0,1285 |
| 109,52 | 0,3661 | 21,37 | -0,1883 | 109,36 | 0,3632 | 21,37 | -0,1462 | 109,31 | 0,3813 | 21,46 | -0,1285 |
| 109,48 | 0,3661 | 21,4 | -0,1884 | 109,33 | 0,3632 | 21,4 | -0,1464 | 109,27 | 0,3812 | 21,49 | -0,1285 |
| 109,45 | 0,3661 | 21,44 | -0,1885 | 109,29 | 0,3632 | 21,43 | -0,1465 | 109,24 | 0,3812 | 21,52 | -0,1285 |
| 109,42 | 0,3661 | 21,47 | -0,1886 | 109,26 | 0,3631 | 21,47 | -0,1466 | 109,21 | 0,3812 | 21,56 | -0,1286 |
| 109,38 | 0,3661 | 21,5 | -0,1886 | 109,23 | 0,3631 | 21,5 | -0,1467 | 109,17 | 0,3812 | 21,59 | -0,1286 |
| 109,35 | 0,3661 | 21,54 | -0,1887 | 109,19 | 0,3631 | 21,53 | -0,1467 | 109,14 | 0,3811 | 21,62 | -0,1286 |
| 109,31 | 0,3661 | 21,57 | -0,1889 | 109,16 | 0,3631 | 21,57 | -0,1468 | 109,1 | 0,3811 | 21,65 | -0,1286 |
| 109,28 | 0,3661 | 21,6 | -0,189 | 109,13 | 0,3631 | 21,6 | -0,1469 | 109,07 | 0,3811 | 21,69 | -0,1287 |
| 109,25 | 0,3661 | 21,64 | -0,1891 | 109,09 | 0,363 | 21,63 | -0,147 | 109,04 | 0,3811 | 21,72 | -0,1287 |
| 109,22 | 0,3661 | 21,67 | -0,1893 | 109,06 | 0,363 | 21,66 | -0,1471 | 109 | 0,381 | 21,75 | -0,1287 |
| 109,18 | 0,3661 | 21,7 | -0,1894 | 109,03 | 0,363 | 21,7 | -0,1472 | 108,97 | 0,381 | 21,79 | -0,1287 |
| 109,15 | 0,3661 | 21,74 | -0,1895 | 108,99 | 0,363 | 21,73 | -0,1472 | 108,94 | 0,381 | 21,82 | -0,1288 |
| 109,11 | 0,3661 | 21,77 | -0,1897 | 108,96 | 0,363 | 21,77 | -0,1473 | 108,9 | 0,381 | 21,86 | -0,1288 |
| 109,08 | 0,3661 | 21,8 | -0,1898 | 108,93 | 0,3629 | 21,8 | -0,1474 | 108,87 | 0,3809 | 21,89 | -0,1288 |
| 109,05 | 0,3661 | 21,84 | -0,19 | 108,9 | 0,3629 | 21,83 | -0,1475 | 108,84 | 0,3809 | 21,92 | -0,1289 |
| 109,02 | 0,3661 | 21,87 | -0,1901 | 108,86 | 0,3629 | 21,87 | -0,1476 | 108,81 | 0,3809 | 21,96 | -0,1289 |
| 108,98 | 0,3661 | 21,9 | -0,1902 | 108,83 | 0,3629 | 21,9 | -0,1477 | 108,77 | 0,3809 | 21,99 | -0,1289 |
| 108,95 | 0,3661 | 21,94 | -0,1903 | 108,79 | 0,3629 | 21,93 | -0,1478 | 108,74 | 0,3808 | 22,02 | -0,1289 |
| 108,92 | 0,3661 | 21,97 | -0,1904 | 108,76 | 0,3628 | 21,97 | -0,1479 | 108,7 | 0,3808 | 22,06 | -0,129 |
| 108,88 | 0,3661 | 22 | -0,1907 | 108,73 | 0,3628 | 22 | -0,148 | 108,67 | 0,3808 | 22,09 | -0,129 |
| 108,85 | 0,3661 | 22,04 | -0,1909 | 108,69 | 0,3628 | 22,03 | -0,1481 | 108,64 | 0,3808 | 22,12 | -0,129 |
| 108,82 | 0,3661 | 22,07 | -0,191 | 108,66 | 0,3628 | 22,06 | -0,1482 | 108,61 | 0,3807 | 22,15 | -0,1291 |
| 108,78 | 0,3661 | 22,1 | -0,1911 | 108,63 | 0,3627 | 22,1 | -0,1483 | 108,57 | 0,3807 | 22,19 | -0,1291 |
| 108,75 | 0,3661 | 22,14 | -0,1912 | 108,59 | 0,3627 | 22,13 | -0,1484 | 108,54 | 0,3807 | 22,22 | -0,1291 |
| 108,71 | 0,3661 | 22,17 | -0,1913 | 108,56 | 0,3627 | 22,17 | -0,1486 | 108,51 | 0,3807 | 22,26 | -0,1291 |
| 108,68 | 0,3661 | 22,2 | -0,1914 | 108,53 | 0,3627 | 22,2 | -0,1486 | 108,47 | 0,3806 | 22,29 | -0,1292 |
| 108,65 | 0,3661 | 22,24 | -0,1915 | 108,5 | 0,3627 | 22,23 | -0,1487 | 108,44 | 0,3806 | 22,32 | -0,1292 |
| 108,62 | 0,3661 | 22,27 | -0,1916 | 108,46 | 0,3626 | 22,26 | -0,1488 | 108,4 | 0,3806 | 22,35 | -0,1292 |
| 108,58 | 0,3661 | 22,3 | -0,1917 | 108,43 | 0,3626 | 22,3 | -0,1489 | 108,37 | 0,3806 | 22,39 | -0,1292 |
| 108,55 | 0,3661 | 22,34 | -0,1918 | 108,39 | 0,3626 | 22,33 | -0,1489 | 108,34 | 0,3805 | 22,42 | -0,1293 |
| 108,52 | 0,3661 | 22,37 | -0,1918 | 108,36 | 0,3626 | 22,36 | -0,149 | 108,31 | 0,3805 | 22,46 | -0,1293 |
| 108,48 | 0,3661 | 22,4 | -0,1919 | 108,33 | 0,3626 | 22,4 | -0,1491 | 108,27 | 0,3805 | 22,49 | -0,1293 |
| 108,45 | 0,3661 | 22,44 | -0,1921 | 108,3 | 0,3625 | 22,43 | -0,1492 | 108,24 | 0,3805 | 22,52 | -0,1293 |
| 108,42 | 0,3661 | 22,47 | -0,1923 | 108,26 | 0,3625 | 22,47 | -0,1493 | 108,2 | 0,3804 | 22,56 | -0,1294 |
| 108,38 | 0,3661 | 22,5 | -0,1924 | 108,23 | 0,3625 | 22,5 | -0,1494 | 108,17 | 0,3804 | 22,59 | -0,1294 |
| 108,35 | 0,3661 | 22,53 | -0,1926 | 108,2 | 0,3625 | 22,53 | -0,1495 | 108,14 | 0,3804 | 22,62 | -0,1294 |
| 108,31 | 0,3661 | 22,57 | -0,1928 | 108,16 | 0,3625 | 22,56 | -0,1497 | 108,11 | 0,3804 | 22,65 | -0,1295 |
| 108,28 | 0,3661 | 22,6 | -0,1928 | 108,13 | 0,3624 | 22,6 | -0,1498 | 108,07 | 0,3803 | 22,69 | -0,1295 |
| 108,25 | 0,3661 | 22,63 | -0,1929 | 108,1 | 0,3624 | 22,63 | -0,1499 | 108,04 | 0,3803 | 22,72 | -0,1295 |
| 108,22 | 0,3661 | 22,67 | -0,1931 | 108,06 | 0,3624 | 22,66 | -0,1499 | 108 | 0,3803 | 22,76 | -0,1295 |
| 108,18 | 0,3661 | 22,7 | -0,1932 | 108,03 | 0,3624 | 22,7 | -0,15 | 107,97 | 0,3803 | 22,79 | -0,1296 |
| 108,15 | 0,3661 | 22,73 | -0,1933 | 107,99 | 0,3624 | 22,73 | -0,1501 | 107,94 | 0,3802 | 22,82 | -0,1296 |
| 108,12 | 0,3661 | 22,77 | -0,1934 | 107,96 | 0,3623 | 22,76 | -0,1502 | 107,9 | 0,3802 | 22,85 | -0,1296 |
| 108,08 | 0,3661 | 22,8 | -0,1935 | 107,93 | 0,3623 | 22,8 | -0,1503 | 107,87 | 0,3802 | 22,89 | -0,1297 |
| 108,05 | 0,3661 | 22,83 | -0,1936 | 107,89 | 0,3623 | 22,83 | -0,1504 | 107,84 | 0,3802 | 22,92 | -0,1297 |
| 108,02 | 0,3661 | 22,87 | -0,1937 | 107,86 | 0,3623 | 22,86 | -0,1505 | 107,81 | 0,3801 | 22,96 | -0,1297 |
| 107,98 | 0,3661 | 22,9 | -0,1938 | 107,83 | 0,3622 | 22,9 | -0,1505 | 107,77 | 0,3801 | 22,99 | -0,1297 |
| 107,95 | 0,3661 | 22,93 | -0,1939 | 107,79 | 0,3622 | 22,93 | -0,1506 | 107,74 | 0,3801 | 23,02 | -0,1298 |
| 107,92 | 0,3661 | 22,97 | -0,194 | 107,76 | 0,3622 | 22,96 | -0,1507 | 107,7 | 0,3801 | 23,06 | -0,1298 |
| 107,88 | 0,3661 | 23 | -0,1942 | 107,73 | 0,3622 | 23 | -0,1508 | 107,67 | 0,38 | 23,09 | -0,1298 |
| 107,85 | 0,366 | 23,03 | -0,1944 | 107,69 | 0,3622 | 23,03 | -0,151 | 107,64 | 0,38 | 23,12 | -0,1299 |
| 107,82 | 0,366 | 23,07 | -0,1946 | 107,66 | 0,3621 | 23,06 | -0,1511 | 107,6 | 0,38 | 23,16 | -0,1299 |
| 107,78 | 0,366 | 23,1 | -0,1947 | 107,63 | 0,3621 | 23,1 | -0,1512 | 107,57 | 0,38 | 23,19 | -0,1299 |
| 107,75 | 0,366 | 23,13 | -0,1949 | 107,59 | 0,3621 | 23,13 | -0,1512 | 107,54 | 0,3799 | 23,22 | -0,1299 |
| 107,72 | 0,366 | 23,17 | -0,195 | 107,56 | 0,3621 | 23,16 | -0,1513 | 107,5 | 0,3799 | 23,26 | -0,13 |
| 107,68 | 0,366 | 23,2 | -0,1951 | 107,53 | 0,3621 | 23,2 | -0,1514 | 107,47 | 0,3799 | 23,29 | -0,13 |
| 107,65 | 0,366 | 23,23 | -0,1953 | 107,49 | 0,362 | 23,23 | -0,1516 | 107,44 | 0,3799 | 23,32 | -0,13 |
| 107,62 | 0,366 | 23,27 | -0,1955 | 107,46 | 0,362 | 23,26 | -0,1517 | 107,41 | 0,3798 | 23,36 | -0,1301 |
| 107,58 | 0,366 | 23,3 | -0,1957 | 107,43 | 0,362 | 23,3 | -0,1518 | 107,37 | 0,3798 | 23,39 | -0,1301 |
| 107,55 | 0,366 | 23,33 | -0,1958 | 107,39 | 0,362 | 23,33 | -0,1519 | 107,34 | 0,3798 | 23,42 | -0,1301 |
| 107,52 | 0,366 | 23,37 | -0,1959 | 107,36 | 0,362 | 23,36 | -0,152 | 107,31 | 0,3798 | 23,46 | -0,1301 |
| 107,48 | 0,366 | 23,4 | -0,196 | 107,33 | 0,3619 | 23,4 | -0,1522 | 107,27 | 0,3797 | 23,49 | -0,1302 |
| 107,45 | 0,366 | 23,43 | -0,196 | 107,29 | 0,3619 | 23,43 | -0,1523 | 107,24 | 0,3797 | 23,52 | -0,1302 |
| 107,42 | 0,366 | 23,47 | -0,1961 | 107,26 | 0,3619 | 23,46 | -0,1525 | 107,21 | 0,3797 | 23,56 | -0,1302 |
| 107,38 | 0,366 | 23,5 | -0,1962 | 107,23 | 0,3619 | 23,5 | -0,1526 | 107,17 | 0,3797 | 23,59 | -0,1303 |
| 107,35 | 0,366 | 23,53 | -0,1964 | 107,19 | 0,3618 | 23,53 | -0,1527 | 107,14 | 0,3796 | 23,62 | -0,1303 |
| 107,32 | 0,366 | 23,57 | -0,1966 | 107,16 | 0,3618 | 23,56 | -0,1527 | 107,1 | 0,3796 | 23,66 | -0,1303 |
| 107,28 | 0,366 | 23,6 | -0,1967 | 107,13 | 0,3618 | 23,6 | -0,1528 | 107,07 | 0,3796 | 23,69 | -0,1303 |
| 107,25 | 0,366 | 23,63 | -0,1968 | 107,09 | 0,3618 | 23,63 | -0,1528 | 107,04 | 0,3796 | 23,72 | -0,1304 |
| 107,21 | 0,366 | 23,67 | -0,1969 | 107,06 | 0,3618 | 23,66 | -0,1529 | 107,01 | 0,3795 | 23,76 | -0,1304 |
| 107,18 | 0,366 | 23,7 | -0,197 | 107,03 | 0,3617 | 23,7 | -0,1531 | 106,97 | 0,3795 | 23,79 | -0,1304 |
| 107,15 | 0,366 | 23,73 | -0,197 | 106,99 | 0,3617 | 23,73 | -0,1532 | 106,94 | 0,3795 | 23,82 | -0,1304 |
| 107,12 | 0,366 | 23,77 | -0,1971 | 106,96 | 0,3617 | 23,76 | -0,1534 | 106,91 | 0,3795 | 23,86 | -0,1305 |
| 107,08 | 0,366 | 23,8 | -0,1973 | 106,93 | 0,3617 | 23,8 | -0,1535 | 106,87 | 0,3794 | 23,89 | -0,1305 |
| 107,05 | 0,366 | 23,83 | -0,1975 | 106,89 | 0,3616 | 23,83 | -0,1537 | 106,84 | 0,3794 | 23,92 | -0,1305 |
| 107,02 | 0,366 | 23,87 | -0,1977 | 106,86 | 0,3616 | 23,86 | -0,1539 | 106,8 | 0,3794 | 23,96 | -0,1306 |
| 106,98 | 0,366 | 23,9 | -0,1978 | 106,83 | 0,3616 | 23,9 | -0,154 | 106,77 | 0,3794 | 23,99 | -0,1306 |
| 106,95 | 0,366 | 23,93 | -0,1979 | 106,79 | 0,3616 | 23,93 | -0,1541 | 106,74 | 0,3793 | 24,02 | -0,1306 |
| 106,92 | 0,366 | 23,97 | -0,1979 | 106,76 | 0,3616 | 23,96 | -0,1542 | 106,71 | 0,3793 | 24,05 | -0,1307 |
| 106,88 | 0,366 | 24 | -0,198 | 106,73 | 0,3615 | 24 | -0,1543 | 106,67 | 0,3793 | 24,09 | -0,1307 |
| 106,85 | 0,366 | 24,03 | -0,1981 | 106,69 | 0,3615 | 24,03 | -0,1543 | 106,64 | 0,3792 | 24,12 | -0,1307 |
| 106,82 | 0,366 | 24,07 | -0,1981 | 106,66 | 0,3615 | 24,06 | -0,1544 | 106,61 | 0,3792 | 24,16 | -0,1307 |
| 106,78 | 0,366 | 24,1 | -0,1982 | 106,63 | 0,3615 | 24,1 | -0,1545 | 106,57 | 0,3792 | 24,19 | -0,1308 |
| 106,75 | 0,366 | 24,13 | -0,1984 | 106,59 | 0,3614 | 24,13 | -0,1546 | 106,54 | 0,3792 | 24,22 | -0,1308 |
| 106,72 | 0,366 | 24,17 | -0,1986 | 106,56 | 0,3614 | 24,16 | -0,1547 | 106,5 | 0,3791 | 24,26 | -0,1308 |
| 106,68 | 0,3659 | 24,2 | -0,1989 | 106,53 | 0,3614 | 24,2 | -0,1547 | 106,47 | 0,3791 | 24,29 | -0,1309 |
| 106,65 | 0,3659 | 24,23 | -0,1991 | 106,49 | 0,3614 | 24,23 | -0,1549 | 106,44 | 0,3791 | 24,32 | -0,1309 |
| 106,62 | 0,3659 | 24,27 | -0,1992 | 106,46 | 0,3614 | 24,26 | -0,155 | 106,4 | 0,3791 | 24,36 | -0,1309 |
| 106,58 | 0,3659 | 24,3 | -0,1993 | 106,43 | 0,3613 | 24,29 | -0,1552 | 106,37 | 0,3791 | 24,39 | -0,1309 |
| 106,55 | 0,3659 | 24,33 | -0,1994 | 106,39 | 0,3613 | 24,33 | -0,1554 | 106,34 | 0,379 | 24,42 | -0,131 |
| 106,52 | 0,3659 | 24,37 | -0,1995 | 106,36 | 0,3613 | 24,36 | -0,1555 | 106,3 | 0,379 | 24,46 | -0,131 |
| 106,48 | 0,3659 | 24,4 | -0,1996 | 106,33 | 0,3613 | 24,4 | -0,1556 | 106,27 | 0,379 | 24,49 | -0,131 |
| 106,45 | 0,3659 | 24,43 | -0,1998 | 106,29 | 0,3612 | 24,43 | -0,1557 | 106,24 | 0,3789 | 24,52 | -0,131 |
| 106,42 | 0,3659 | 24,46 | -0,1999 | 106,26 | 0,3612 | 24,46 | -0,1558 | 106,2 | 0,3789 | 24,56 | -0,1311 |
| 106,38 | 0,3659 | 24,5 | -0,2 | 106,23 | 0,3612 | 24,5 | -0,1559 | 106,17 | 0,3789 | 24,59 | -0,1311 |
| 106,35 | 0,3659 | 24,53 | -0,2001 | 106,19 | 0,3612 | 24,53 | -0,156 | 106,14 | 0,3789 | 24,62 | -0,1311 |
| 106,32 | 0,3659 | 24,57 | -0,2002 | 106,16 | 0,3611 | 24,56 | -0,1562 | 106,1 | 0,3788 | 24,66 | -0,1312 |
| 106,28 | 0,3659 | 24,6 | -0,2003 | 106,13 | 0,3611 | 24,6 | -0,1563 | 106,07 | 0,3788 | 24,69 | -0,1312 |
| 106,25 | 0,3659 | 24,63 | -0,2004 | 106,09 | 0,3611 | 24,63 | -0,1564 | 106,04 | 0,3788 | 24,72 | -0,1312 |
| 106,22 | 0,3659 | 24,67 | -0,2005 | 106,06 | 0,3611 | 24,66 | -0,1565 | 106 | 0,3788 | 24,76 | -0,1312 |
| 106,18 | 0,3659 | 24,7 | -0,2007 | 106,03 | 0,3611 | 24,7 | -0,1566 | 105,97 | 0,3787 | 24,79 | -0,1313 |
| 106,15 | 0,3659 | 24,73 | -0,2008 | 105,99 | 0,361 | 24,73 | -0,1567 | 105,94 | 0,3787 | 24,82 | -0,1313 |
| 106,12 | 0,3659 | 24,76 | -0,201 | 105,96 | 0,361 | 24,76 | -0,1568 | 105,9 | 0,3787 | 24,86 | -0,1313 |
| 106,08 | 0,3659 | 24,8 | -0,2011 | 105,93 | 0,361 | 24,8 | -0,1568 | 105,87 | 0,3787 | 24,89 | -0,1314 |
| 106,05 | 0,3659 | 24,83 | -0,2012 | 105,89 | 0,361 | 24,83 | -0,1569 | 105,84 | 0,3786 | 24,92 | -0,1314 |
| 106,02 | 0,3659 | 24,87 | -0,2014 | 105,86 | 0,3609 | 24,86 | -0,1571 | 105,8 | 0,3786 | 24,96 | -0,1314 |
| 105,98 | 0,3659 | 24,9 | -0,2015 | 105,83 | 0,3609 | 24,9 | -0,1573 | 105,77 | 0,3786 | 24,99 | -0,1315 |
| 105,95 | 0,3659 | 24,93 | -0,2016 | 105,79 | 0,3609 | 24,93 | -0,1576 | 105,74 | 0,3785 | 25,02 | -0,1315 |
| 105,92 | 0,3658 | 24,96 | -0,2017 | 105,76 | 0,3609 | 24,96 | -0,1578 | 105,71 | 0,3785 | 25,06 | -0,1315 |
| 105,88 | 0,3658 | 25 | -0,2018 | 105,73 | 0,3609 | 25 | -0,158 | 105,67 | 0,3785 | 25,09 | -0,1315 |
| 105,85 | 0,3658 | 25,03 | -0,2019 | 105,69 | 0,3608 | 25,03 | -0,1581 | 105,64 | 0,3785 | 25,12 | -0,1316 |
| 105,82 | 0,3658 | 25,07 | -0,202 | 105,66 | 0,3608 | 25,06 | -0,1582 | 105,6 | 0,3784 | 25,16 | -0,1316 |
| 105,78 | 0,3658 | 25,1 | -0,2021 | 105,63 | 0,3608 | 25,1 | -0,1582 | 105,57 | 0,3784 | 25,19 | -0,1316 |
| 105,75 | 0,3658 | 25,13 | -0,2022 | 105,59 | 0,3608 | 25,13 | -0,1583 | 105,54 | 0,3784 | 25,22 | -0,1317 |
| 105,72 | 0,3658 | 25,16 | -0,2023 | 105,56 | 0,3607 | 25,16 | -0,1584 | 105,5 | 0,3784 | 25,26 | -0,1317 |
| 105,68 | 0,3658 | 25,2 | -0,2024 | 105,53 | 0,3607 | 25,2 | -0,1585 | 105,47 | 0,3783 | 25,29 | -0,1317 |
| 105,65 | 0,3658 | 25,23 | -0,2025 | 105,49 | 0,3607 | 25,23 | -0,1586 | 105,44 | 0,3783 | 25,32 | -0,1317 |
| 105,61 | 0,3658 | 25,26 | -0,2026 | 105,46 | 0,3607 | 25,26 | -0,1588 | 105,41 | 0,3783 | 25,36 | -0,1318 |
| 105,58 | 0,3658 | 25,3 | -0,2027 | 105,43 | 0,3607 | 25,3 | -0,1591 | 105,37 | 0,3783 | 25,39 | -0,1318 |
| 105,55 | 0,3658 | 25,33 | -0,2027 | 105,39 | 0,3606 | 25,33 | -0,1593 | 105,34 | 0,3782 | 25,42 | -0,1318 |
| 105,52 | 0,3658 | 25,37 | -0,2028 | 105,36 | 0,3606 | 25,36 | -0,1595 | 105,3 | 0,3782 | 25,46 | -0,1318 |
| 105,48 | 0,3658 | 25,4 | -0,2029 | 105,33 | 0,3606 | 25,39 | -0,1595 | 105,27 | 0,3782 | 25,49 | -0,1319 |
| 105,45 | 0,3658 | 25,43 | -0,203 | 105,29 | 0,3606 | 25,43 | -0,1596 | 105,24 | 0,3782 | 25,52 | -0,1319 |
| 105,42 | 0,3658 | 25,46 | -0,2032 | 105,26 | 0,3605 | 25,46 | -0,1596 | 105,2 | 0,3781 | 25,56 | -0,1319 |
| 105,38 | 0,3658 | 25,5 | -0,2033 | 105,23 | 0,3605 | 25,49 | -0,1598 | 105,17 | 0,3781 | 25,59 | -0,132 |
| 105,35 | 0,3658 | 25,53 | -0,2034 | 105,19 | 0,3605 | 25,53 | -0,16 | 105,14 | 0,3781 | 25,62 | -0,132 |
| 105,32 | 0,3658 | 25,56 | -0,2036 | 105,16 | 0,3605 | 25,56 | -0,1601 | 105,1 | 0,3781 | 25,66 | -0,132 |
| 105,28 | 0,3658 | 25,6 | -0,2037 | 105,13 | 0,3605 | 25,59 | -0,1603 | 105,07 | 0,378 | 25,69 | -0,1321 |
| 105,25 | 0,3658 | 25,63 | -0,2038 | 105,09 | 0,3604 | 25,63 | -0,1603 | 105,04 | 0,378 | 25,72 | -0,1321 |
| 105,22 | 0,3657 | 25,66 | -0,2038 | 105,06 | 0,3604 | 25,66 | -0,1604 | 105 | 0,378 | 25,76 | -0,1321 |
| 105,18 | 0,3657 | 25,7 | -0,2039 | 105,03 | 0,3604 | 25,69 | -0,1606 | 104,97 | 0,378 | 25,79 | -0,1322 |
| 105,15 | 0,3657 | 25,73 | -0,204 | 104,99 | 0,3604 | 25,73 | -0,1607 | 104,94 | 0,378 | 25,82 | -0,1322 |
| 105,12 | 0,3657 | 25,76 | -0,2041 | 104,96 | 0,3603 | 25,76 | -0,1609 | 104,9 | 0,3779 | 25,86 | -0,1322 |
| 105,08 | 0,3657 | 25,8 | -0,2043 | 104,93 | 0,3603 | 25,79 | -0,1611 | 104,87 | 0,3779 | 25,89 | -0,1322 |
| 105,05 | 0,3657 | 25,83 | -0,2044 | 104,89 | 0,3603 | 25,83 | -0,1612 | 104,84 | 0,3779 | 25,92 | -0,1323 |
| 105,02 | 0,3657 | 25,86 | -0,2045 | 104,86 | 0,3603 | 25,86 | -0,1614 | 104,81 | 0,3779 | 25,96 | -0,1323 |
| 104,98 | 0,3657 | 25,9 | -0,2047 | 104,83 | 0,3603 | 25,89 | -0,1615 | 104,77 | 0,3778 | 25,99 | -0,1323 |
| 104,95 | 0,3657 | 25,93 | -0,2048 | 104,79 | 0,3602 | 25,93 | -0,1616 | 104,74 | 0,3778 | 26,02 | -0,1324 |
| 104,92 | 0,3657 | 25,96 | -0,205 | 104,76 | 0,3602 | 25,96 | -0,1617 | 104,7 | 0,3778 | 26,06 | -0,1324 |
| 104,88 | 0,3657 | 26 | -0,2052 | 104,73 | 0,3602 | 25,99 | -0,1618 | 104,67 | 0,3778 | 26,09 | -0,1324 |
| 104,85 | 0,3657 | 26,03 | -0,2053 | 104,69 | 0,3602 | 26,03 | -0,162 | 104,64 | 0,3777 | 26,12 | -0,1325 |
| 104,82 | 0,3657 | 26,06 | -0,2054 | 104,66 | 0,3601 | 26,06 | -0,1621 | 104,61 | 0,3777 | 26,16 | -0,1325 |
| 104,78 | 0,3657 | 26,1 | -0,2055 | 104,63 | 0,3601 | 26,09 | -0,1622 | 104,57 | 0,3777 | 26,19 | -0,1325 |
| 104,75 | 0,3657 | 26,13 | -0,2056 | 104,59 | 0,3601 | 26,13 | -0,1625 | 104,54 | 0,3776 | 26,22 | -0,1325 |
| 104,72 | 0,3657 | 26,16 | -0,2057 | 104,56 | 0,3601 | 26,16 | -0,1627 | 104,5 | 0,3776 | 26,26 | -0,1326 |
| 104,68 | 0,3657 | 26,2 | -0,2059 | 104,53 | 0,36 | 26,19 | -0,1629 | 104,47 | 0,3776 | 26,29 | -0,1326 |
| 104,65 | 0,3657 | 26,23 | -0,206 | 104,49 | 0,36 | 26,23 | -0,1631 | 104,44 | 0,3776 | 26,32 | -0,1326 |
| 104,62 | 0,3657 | 26,26 | -0,2061 | 104,46 | 0,36 | 26,26 | -0,1632 | 104,4 | 0,3775 | 26,36 | -0,1326 |
| 104,58 | 0,3657 | 26,3 | -0,2063 | 104,43 | 0,36 | 26,29 | -0,1633 | 104,37 | 0,3775 | 26,39 | -0,1327 |
| 104,55 | 0,3657 | 26,33 | -0,2064 | 104,39 | 0,36 | 26,33 | -0,1634 | 104,34 | 0,3775 | 26,42 | -0,1327 |
| 104,52 | 0,3656 | 26,36 | -0,2065 | 104,36 | 0,3599 | 26,36 | -0,1635 | 104,3 | 0,3775 | 26,46 | -0,1327 |
| 104,48 | 0,3656 | 26,4 | -0,2066 | 104,33 | 0,3599 | 26,39 | -0,1636 | 104,27 | 0,3774 | 26,49 | -0,1328 |
| 104,45 | 0,3656 | 26,43 | -0,2066 | 104,29 | 0,3599 | 26,43 | -0,1637 | 104,24 | 0,3774 | 26,52 | -0,1328 |
| 104,42 | 0,3656 | 26,46 | -0,2068 | 104,26 | 0,3599 | 26,46 | -0,1638 | 104,21 | 0,3774 | 26,56 | -0,1328 |
| 104,38 | 0,3656 | 26,5 | -0,2069 | 104,23 | 0,3599 | 26,49 | -0,1639 | 104,17 | 0,3774 | 26,59 | -0,1329 |
| 104,35 | 0,3656 | 26,53 | -0,207 | 104,2 | 0,3598 | 26,53 | -0,164 | 104,14 | 0,3773 | 26,62 | -0,1329 |
| 104,32 | 0,3656 | 26,56 | -0,2071 | 104,16 | 0,3598 | 26,56 | -0,1642 | 104,11 | 0,3773 | 26,66 | -0,1329 |
| 104,28 | 0,3656 | 26,6 | -0,2072 | 104,13 | 0,3598 | 26,59 | -0,1643 | 104,07 | 0,3773 | 26,69 | -0,133 |
| 104,25 | 0,3656 | 26,63 | -0,2073 | 104,09 | 0,3598 | 26,63 | -0,1645 | 104,04 | 0,3773 | 26,72 | -0,133 |
| 104,22 | 0,3656 | 26,66 | -0,2074 | 104,06 | 0,3597 | 26,66 | -0,1647 | 104 | 0,3773 | 26,76 | -0,133 |
| 104,18 | 0,3656 | 26,7 | -0,2075 | 104,03 | 0,3597 | 26,69 | -0,1648 | 103,97 | 0,3772 | 26,79 | -0,133 |
| 104,15 | 0,3656 | 26,73 | -0,2076 | 103,99 | 0,3597 | 26,73 | -0,165 | 103,94 | 0,3772 | 26,82 | -0,1331 |
| 104,12 | 0,3656 | 26,76 | -0,2077 | 103,96 | 0,3597 | 26,76 | -0,1651 | 103,9 | 0,3772 | 26,86 | -0,1331 |
| 104,08 | 0,3656 | 26,8 | -0,2078 | 103,93 | 0,3597 | 26,79 | -0,1652 | 103,87 | 0,3772 | 26,89 | -0,1331 |
| 104,05 | 0,3656 | 26,83 | -0,2079 | 103,89 | 0,3596 | 26,83 | -0,1653 | 103,84 | 0,3771 | 26,92 | -0,1332 |
| 104,02 | 0,3656 | 26,86 | -0,208 | 103,86 | 0,3596 | 26,86 | -0,1655 | 103,81 | 0,3771 | 26,96 | -0,1332 |
| 103,98 | 0,3656 | 26,9 | -0,208 | 103,83 | 0,3596 | 26,89 | -0,1656 | 103,77 | 0,3771 | 26,99 | -0,1332 |
| 103,95 | 0,3656 | 26,93 | -0,2081 | 103,8 | 0,3596 | 26,93 | -0,1658 | 103,74 | 0,3771 | 27,02 | -0,1333 |
| 103,92 | 0,3656 | 26,96 | -0,2083 | 103,76 | 0,3595 | 26,96 | -0,1659 | 103,7 | 0,377 | 27,06 | -0,1333 |
| 103,88 | 0,3656 | 27 | -0,2084 | 103,73 | 0,3595 | 26,99 | -0,166 | 103,67 | 0,377 | 27,09 | -0,1333 |
| 103,85 | 0,3655 | 27,03 | -0,2086 | 103,7 | 0,3595 | 27,03 | -0,1662 | 103,64 | 0,377 | 27,12 | -0,1333 |
| 103,82 | 0,3655 | 27,06 | -0,2087 | 103,66 | 0,3595 | 27,06 | -0,1664 | 103,61 | 0,377 | 27,16 | -0,1334 |
| 103,78 | 0,3655 | 27,1 | -0,2088 | 103,63 | 0,3595 | 27,09 | -0,1666 | 103,57 | 0,3769 | 27,19 | -0,1334 |
| 103,75 | 0,3655 | 27,13 | -0,2089 | 103,59 | 0,3594 | 27,13 | -0,1667 | 103,54 | 0,3769 | 27,22 | -0,1334 |
| 103,72 | 0,3655 | 27,16 | -0,209 | 103,56 | 0,3594 | 27,16 | -0,1668 | 103,5 | 0,3769 | 27,26 | -0,1335 |
| 103,68 | 0,3655 | 27,2 | -0,2091 | 103,53 | 0,3594 | 27,19 | -0,1669 | 103,47 | 0,3768 | 27,29 | -0,1335 |
| 103,65 | 0,3655 | 27,23 | -0,2092 | 103,5 | 0,3594 | 27,23 | -0,167 | 103,44 | 0,3768 | 27,32 | -0,1335 |
| 103,62 | 0,3655 | 27,26 | -0,2094 | 103,46 | 0,3593 | 27,26 | -0,1671 | 103,4 | 0,3768 | 27,36 | -0,1336 |
| 103,58 | 0,3655 | 27,3 | -0,2095 | 103,43 | 0,3593 | 27,29 | -0,1672 | 103,37 | 0,3768 | 27,39 | -0,1336 |
| 103,55 | 0,3655 | 27,33 | -0,2095 | 103,4 | 0,3593 | 27,32 | -0,1673 | 103,34 | 0,3768 | 27,42 | -0,1336 |
| 103,52 | 0,3655 | 27,36 | -0,2096 | 103,36 | 0,3593 | 27,36 | -0,1675 | 103,3 | 0,3767 | 27,46 | -0,1336 |
| 103,48 | 0,3655 | 27,4 | -0,2097 | 103,33 | 0,3593 | 27,39 | -0,1677 | 103,27 | 0,3767 | 27,49 | -0,1337 |
| 103,45 | 0,3655 | 27,43 | -0,2098 | 103,3 | 0,3592 | 27,42 | -0,168 | 103,24 | 0,3767 | 27,52 | -0,1337 |
| 103,42 | 0,3655 | 27,46 | -0,2099 | 103,26 | 0,3592 | 27,46 | -0,1682 | 103,21 | 0,3766 | 27,56 | -0,1337 |
| 103,38 | 0,3655 | 27,49 | -0,21 | 103,23 | 0,3592 | 27,49 | -0,1683 | 103,17 | 0,3766 | 27,59 | -0,1338 |
| 103,35 | 0,3654 | 27,53 | -0,2101 | 103,2 | 0,3592 | 27,52 | -0,1683 | 103,14 | 0,3766 | 27,62 | -0,1338 |
| 103,32 | 0,3654 | 27,56 | -0,2103 | 103,16 | 0,3591 | 27,56 | -0,1684 | 103,1 | 0,3766 | 27,66 | -0,1338 |
| 103,28 | 0,3654 | 27,59 | -0,2104 | 103,13 | 0,3591 | 27,59 | -0,1685 | 103,07 | 0,3765 | 27,69 | -0,1338 |
| 103,25 | 0,3654 | 27,63 | -0,2105 | 103,1 | 0,3591 | 27,62 | -0,1686 | 103,04 | 0,3765 | 27,72 | -0,1339 |
| 103,21 | 0,3654 | 27,66 | -0,2106 | 103,06 | 0,3591 | 27,66 | -0,1689 | 103,01 | 0,3765 | 27,76 | -0,1339 |
| 103,18 | 0,3654 | 27,7 | -0,2107 | 103,03 | 0,359 | 27,69 | -0,1692 | 102,97 | 0,3765 | 27,79 | -0,1339 |
| 103,15 | 0,3654 | 27,73 | -0,2108 | 103 | 0,359 | 27,72 | -0,1695 | 102,94 | 0,3764 | 27,82 | -0,134 |
| 103,12 | 0,3654 | 27,76 | -0,2109 | 102,96 | 0,359 | 27,76 | -0,1696 | 102,9 | 0,3764 | 27,86 | -0,134 |
| 103,08 | 0,3654 | 27,79 | -0,211 | 102,93 | 0,359 | 27,79 | -0,1697 | 102,87 | 0,3764 | 27,89 | -0,134 |
| 103,05 | 0,3654 | 27,83 | -0,211 | 102,89 | 0,359 | 27,82 | -0,1698 | 102,84 | 0,3764 | 27,92 | -0,1341 |
| 103,01 | 0,3654 | 27,86 | -0,2111 | 102,86 | 0,3589 | 27,86 | -0,1699 | 102,81 | 0,3763 | 27,96 | -0,1341 |
| 102,98 | 0,3654 | 27,89 | -0,2112 | 102,83 | 0,3589 | 27,89 | -0,17 | 102,77 | 0,3763 | 27,99 | -0,1341 |
| 102,95 | 0,3654 | 27,93 | -0,2114 | 102,8 | 0,3589 | 27,92 | -0,1702 | 102,74 | 0,3763 | 28,02 | -0,1342 |
| 102,92 | 0,3654 | 27,96 | -0,2116 | 102,76 | 0,3589 | 27,96 | -0,1704 | 102,7 | 0,3763 | 28,06 | -0,1342 |
| 102,88 | 0,3653 | 27,99 | -0,2118 | 102,73 | 0,3588 | 27,99 | -0,1705 | 102,67 | 0,3762 | 28,09 | -0,1342 |
| 102,85 | 0,3653 | 28,03 | -0,2119 | 102,7 | 0,3588 | 28,02 | -0,1706 | 102,64 | 0,3762 | 28,12 | -0,1343 |
| 102,82 | 0,3653 | 28,06 | -0,212 | 102,66 | 0,3588 | 28,06 | -0,1708 | 102,61 | 0,3762 | 28,16 | -0,1343 |
| 102,78 | 0,3653 | 28,09 | -0,212 | 102,63 | 0,3588 | 28,09 | -0,1709 | 102,57 | 0,3762 | 28,19 | -0,1343 |
| 102,75 | 0,3653 | 28,13 | -0,2121 | 102,6 | 0,3588 | 28,12 | -0,1711 | 102,54 | 0,3761 | 28,22 | -0,1343 |
| 102,71 | 0,3653 | 28,16 | -0,2122 | 102,56 | 0,3587 | 28,16 | -0,1712 | 102,5 | 0,3761 | 28,26 | -0,1344 |
| 102,68 | 0,3653 | 28,19 | -0,2123 | 102,53 | 0,3587 | 28,19 | -0,1714 | 102,47 | 0,3761 | 28,29 | -0,1344 |
| 102,65 | 0,3653 | 28,23 | -0,2124 | 102,49 | 0,3587 | 28,22 | -0,1715 | 102,44 | 0,3761 | 28,32 | -0,1344 |
| 102,62 | 0,3653 | 28,26 | -0,2125 | 102,46 | 0,3587 | 28,26 | -0,1716 | 102,4 | 0,376 | 28,36 | -0,1345 |
| 102,58 | 0,3653 | 28,3 | -0,2125 | 102,43 | 0,3586 | 28,29 | -0,1716 | 102,37 | 0,376 | 28,39 | -0,1345 |
| 102,55 | 0,3653 | 28,33 | -0,2126 | 102,4 | 0,3586 | 28,32 | -0,1717 | 102,34 | 0,376 | 28,42 | -0,1345 |
| 102,52 | 0,3653 | 28,36 | -0,2127 | 102,36 | 0,3586 | 28,36 | -0,1719 | 102,31 | 0,3759 | 28,46 | -0,1346 |
| 102,48 | 0,3653 | 28,39 | -0,2128 | 102,33 | 0,3586 | 28,39 | -0,1721 | 102,27 | 0,3759 | 28,49 | -0,1346 |
| 102,45 | 0,3652 | 28,43 | -0,2129 | 102,3 | 0,3585 | 28,42 | -0,1724 | 102,24 | 0,3759 | 28,52 | -0,1346 |
| 102,42 | 0,3652 | 28,46 | -0,2131 | 102,26 | 0,3585 | 28,46 | -0,1725 | 102,2 | 0,3759 | 28,56 | -0,1346 |
| 102,38 | 0,3652 | 28,5 | -0,2132 | 102,23 | 0,3585 | 28,49 | -0,1727 | 102,17 | 0,3758 | 28,59 | -0,1347 |
| 102,35 | 0,3652 | 28,53 | -0,2134 | 102,2 | 0,3585 | 28,52 | -0,1729 | 102,14 | 0,3758 | 28,62 | -0,1347 |
| 102,32 | 0,3652 | 28,56 | -0,2135 | 102,16 | 0,3585 | 28,56 | -0,1731 | 102,11 | 0,3758 | 28,66 | -0,1347 |
| 102,28 | 0,3652 | 28,59 | -0,2135 | 102,13 | 0,3584 | 28,59 | -0,1732 | 102,07 | 0,3757 | 28,69 | -0,1348 |
| 102,25 | 0,3652 | 28,63 | -0,2136 | 102,1 | 0,3584 | 28,62 | -0,1733 | 102,04 | 0,3757 | 28,73 | -0,1348 |
| 102,22 | 0,3652 | 28,66 | -0,2137 | 102,06 | 0,3584 | 28,66 | -0,1734 | 102 | 0,3757 | 28,76 | -0,1348 |
| 102,18 | 0,3652 | 28,69 | -0,2137 | 102,03 | 0,3584 | 28,69 | -0,1735 | 101,97 | 0,3757 | 28,79 | -0,1349 |
| 102,15 | 0,3652 | 28,73 | -0,2138 | 102 | 0,3583 | 28,72 | -0,1736 | 101,94 | 0,3756 | 28,82 | -0,1349 |
| 102,12 | 0,3652 | 28,76 | -0,2139 | 101,96 | 0,3583 | 28,76 | -0,1738 | 101,91 | 0,3756 | 28,86 | -0,1349 |
| 102,08 | 0,3651 | 28,79 | -0,214 | 101,93 | 0,3583 | 28,79 | -0,174 | 101,87 | 0,3756 | 28,89 | -0,135 |
| 102,05 | 0,3651 | 28,83 | -0,2141 | 101,9 | 0,3583 | 28,82 | -0,1743 | 101,84 | 0,3756 | 28,93 | -0,135 |
| 102,02 | 0,3651 | 28,86 | -0,2142 | 101,86 | 0,3583 | 28,86 | -0,1745 | 101,8 | 0,3755 | 28,96 | -0,135 |
| 101,98 | 0,3651 | 28,89 | -0,2143 | 101,83 | 0,3582 | 28,89 | -0,1747 | 101,77 | 0,3755 | 28,99 | -0,1351 |
| 101,95 | 0,3651 | 28,93 | -0,2144 | 101,8 | 0,3582 | 28,92 | -0,1748 | 101,74 | 0,3755 | 29,03 | -0,1351 |
| 101,92 | 0,3651 | 28,96 | -0,2145 | 101,76 | 0,3582 | 28,96 | -0,175 | 101,71 | 0,3754 | 29,06 | -0,1351 |
| 101,88 | 0,3651 | 28,99 | -0,2145 | 101,73 | 0,3582 | 28,99 | -0,1753 | 101,67 | 0,3754 | 29,09 | -0,1352 |
| 101,85 | 0,3651 | 29,03 | -0,2146 | 101,7 | 0,3581 | 29,02 | -0,1755 | 101,64 | 0,3754 | 29,13 | -0,1352 |
| 101,81 | 0,3651 | 29,06 | -0,2147 | 101,66 | 0,3581 | 29,06 | -0,1757 | 101,6 | 0,3754 | 29,16 | -0,1352 |
| 101,78 | 0,3651 | 29,09 | -0,2149 | 101,63 | 0,3581 | 29,09 | -0,1758 | 101,57 | 0,3753 | 29,19 | -0,1353 |
| 101,75 | 0,3651 | 29,13 | -0,215 | 101,6 | 0,3581 | 29,12 | -0,1759 | 101,54 | 0,3753 | 29,22 | -0,1353 |
| 101,72 | 0,365 | 29,16 | -0,2152 | 101,56 | 0,358 | 29,15 | -0,1759 | 101,5 | 0,3753 | 29,26 | -0,1353 |
| 101,68 | 0,365 | 29,19 | -0,2152 | 101,53 | 0,358 | 29,19 | -0,1761 | 101,47 | 0,3752 | 29,29 | -0,1353 |
| 101,65 | 0,365 | 29,23 | -0,2153 | 101,49 | 0,358 | 29,22 | -0,1762 | 101,44 | 0,3752 | 29,33 | -0,1354 |
| 101,62 | 0,365 | 29,26 | -0,2154 | 101,46 | 0,358 | 29,26 | -0,1764 | 101,4 | 0,3752 | 29,36 | -0,1354 |
| 101,58 | 0,365 | 29,29 | -0,2155 | 101,43 | 0,3579 | 29,29 | -0,1766 | 101,37 | 0,3752 | 29,39 | -0,1354 |
| 101,55 | 0,365 | 29,33 | -0,2156 | 101,39 | 0,3579 | 29,32 | -0,1767 | 101,34 | 0,3751 | 29,43 | -0,1355 |
| 101,51 | 0,365 | 29,36 | -0,2157 | 101,36 | 0,3579 | 29,35 | -0,1768 | 101,3 | 0,3751 | 29,46 | -0,1355 |
| 101,48 | 0,365 | 29,39 | -0,2158 | 101,33 | 0,3579 | 29,39 | -0,1769 | 101,27 | 0,3751 | 29,49 | -0,1355 |
| 101,45 | 0,365 | 29,43 | -0,2159 | 101,29 | 0,3579 | 29,42 | -0,1771 | 101,24 | 0,375 | 29,52 | -0,1356 |
| 101,42 | 0,3649 | 29,46 | -0,2159 | 101,26 | 0,3578 | 29,45 | -0,1772 | 101,21 | 0,375 | 29,56 | -0,1356 |
| 101,38 | 0,3649 | 29,49 | -0,216 | 101,23 | 0,3578 | 29,49 | -0,1774 | 101,17 | 0,375 | 29,59 | -0,1356 |
| 101,35 | 0,3649 | 29,53 | -0,2161 | 101,2 | 0,3578 | 29,52 | -0,1777 | 101,14 | 0,375 | 29,63 | -0,1356 |
| 101,32 | 0,3649 | 29,56 | -0,2161 | 101,16 | 0,3578 | 29,55 | -0,1779 | 101,11 | 0,3749 | 29,66 | -0,1357 |
| 101,28 | 0,3649 | 29,59 | -0,2162 | 101,13 | 0,3577 | 29,59 | -0,1781 | 101,07 | 0,3749 | 29,69 | -0,1357 |
| 101,25 | 0,3649 | 29,63 | -0,2163 | 101,09 | 0,3577 | 29,62 | -0,1782 | 101,04 | 0,3749 | 29,72 | -0,1357 |
| 101,22 | 0,3649 | 29,66 | -0,2164 | 101,06 | 0,3577 | 29,65 | -0,1783 | 101 | 0,3748 | 29,76 | -0,1358 |
| 101,18 | 0,3649 | 29,69 | -0,2165 | 101,03 | 0,3577 | 29,69 | -0,1786 | 100,97 | 0,3748 | 29,79 | -0,1358 |
| 101,15 | 0,3648 | 29,73 | -0,2166 | 101 | 0,3576 | 29,72 | -0,1788 | 100,94 | 0,3748 | 29,82 | -0,1358 |
| 101,12 | 0,3648 | 29,76 | -0,2166 | 100,96 | 0,3576 | 29,75 | -0,1789 | 100,9 | 0,3748 | 29,86 | -0,1359 |
| 101,08 | 0,3648 | 29,79 | -0,2167 | 100,93 | 0,3576 | 29,79 | -0,1791 | 100,87 | 0,3747 | 29,89 | -0,1359 |
| 101,05 | 0,3648 | 29,83 | -0,2168 | 100,9 | 0,3576 | 29,82 | -0,1791 | 100,84 | 0,3747 | 29,93 | -0,1359 |
| 101,02 | 0,3648 | 29,86 | -0,217 | 100,86 | 0,3575 | 29,85 | -0,1792 | 100,8 | 0,3747 | 29,96 | -0,136 |
| 100,98 | 0,3648 | 29,89 | -0,217 | 100,83 | 0,3575 | 29,88 | -0,1793 | 100,77 | 0,3746 | 29,99 | -0,136 |
| 100,95 | 0,3648 | 29,93 | -0,2171 | 100,8 | 0,3575 | 29,92 | -0,1794 | 100,74 | 0,3746 | 30,03 | -0,136 |
| 100,92 | 0,3648 | 29,96 | -0,2172 | 100,76 | 0,3575 | 29,95 | -0,1795 | 100,7 | 0,3746 | 30,06 | -0,1361 |
| 100,88 | 0,3648 | 29,99 | -0,2172 | 100,73 | 0,3574 | 29,99 | -0,1797 | 100,67 | 0,3745 | 30,09 | -0,1361 |
| 100,85 | 0,3647 | 30,03 | -0,2173 | 100,7 | 0,3574 | 30,02 | -0,1798 | 100,64 | 0,3745 | 30,13 | -0,1361 |
| 100,82 | 0,3647 | 30,06 | -0,2173 | 100,66 | 0,3574 | 30,05 | -0,18 | 100,6 | 0,3745 | 30,16 | -0,1362 |
| 100,78 | 0,3647 | 30,09 | -0,2174 | 100,63 | 0,3574 | 30,09 | -0,1801 | 100,57 | 0,3745 | 30,19 | -0,1362 |
| 100,75 | 0,3647 | 30,13 | -0,2175 | 100,59 | 0,3573 | 30,12 | -0,1802 | 100,54 | 0,3744 | 30,23 | -0,1362 |
| 100,72 | 0,3647 | 30,16 | -0,2175 | 100,56 | 0,3573 | 30,16 | -0,1804 | 100,5 | 0,3744 | 30,26 | -0,1363 |
| 100,68 | 0,3647 | 30,19 | -0,2176 | 100,53 | 0,3573 | 30,19 | -0,1806 | 100,47 | 0,3744 | 30,29 | -0,1363 |
| 100,65 | 0,3647 | 30,23 | -0,2177 | 100,5 | 0,3573 | 30,22 | -0,1807 | 100,44 | 0,3743 | 30,33 | -0,1363 |
| 100,62 | 0,3647 | 30,26 | -0,2177 | 100,46 | 0,3572 | 30,25 | -0,1809 | 100,4 | 0,3743 | 30,36 | -0,1364 |
| 100,58 | 0,3646 | 30,29 | -0,2178 | 100,43 | 0,3572 | 30,29 | -0,181 | 100,37 | 0,3743 | 30,39 | -0,1364 |
| 100,55 | 0,3646 | 30,33 | -0,2179 | 100,4 | 0,3572 | 30,32 | -0,1811 | 100,34 | 0,3742 | 30,43 | -0,1364 |
| 100,52 | 0,3646 | 30,36 | -0,2179 | 100,36 | 0,3572 | 30,35 | -0,1813 | 100,3 | 0,3742 | 30,46 | -0,1365 |
| 100,48 | 0,3646 | 30,39 | -0,218 | 100,33 | 0,3571 | 30,39 | -0,1815 | 100,27 | 0,3742 | 30,49 | -0,1365 |
| 100,45 | 0,3646 | 30,43 | -0,218 | 100,29 | 0,3571 | 30,42 | -0,1817 | 100,24 | 0,3741 | 30,53 | -0,1365 |
| 100,41 | 0,3646 | 30,46 | -0,2181 | 100,26 | 0,3571 | 30,45 | -0,1819 | 100,2 | 0,3741 | 30,56 | -0,1365 |
| 100,38 | 0,3646 | 30,49 | -0,2182 | 100,23 | 0,3571 | 30,49 | -0,182 | 100,17 | 0,3741 | 30,59 | -0,1366 |
| 100,35 | 0,3645 | 30,53 | -0,2183 | 100,19 | 0,357 | 30,52 | -0,182 | 100,14 | 0,3741 | 30,63 | -0,1366 |
| 100,32 | 0,3645 | 30,56 | -0,2184 | 100,16 | 0,357 | 30,55 | -0,1821 | 100,1 | 0,374 | 30,66 | -0,1366 |
| 100,28 | 0,3645 | 30,59 | -0,2186 | 100,13 | 0,357 | 30,59 | -0,1823 | 100,07 | 0,374 | 30,69 | -0,1367 |
| 100,25 | 0,3645 | 30,63 | -0,2186 | 100,1 | 0,357 | 30,62 | -0,1825 | 100,04 | 0,374 | 30,73 | -0,1367 |
| 100,22 | 0,3645 | 30,66 | -0,2187 | 100,06 | 0,3569 | 30,65 | -0,1827 | 100 | 0,3739 | 30,76 | -0,1367 |
| 100,18 | 0,3645 | 30,69 | -0,2188 | 100,03 | 0,3569 | 30,68 | -0,1829 | 99,97 | 0,3739 | 30,79 | -0,1368 |
| 100,15 | 0,3645 | 30,73 | -0,2188 | 100 | 0,3569 | 30,72 | -0,1831 | 99,94 | 0,3739 | 30,83 | -0,1368 |
| 100,12 | 0,3644 | 30,76 | -0,2189 | 99,96 | 0,3569 | 30,75 | -0,1833 | 99,91 | 0,3738 | 30,86 | -0,1368 |
| 100,08 | 0,3644 | 30,79 | -0,2189 | 99,93 | 0,3568 | 30,78 | -0,1834 | 99,87 | 0,3738 | 30,89 | -0,1369 |
| 100,05 | 0,3644 | 30,82 | -0,219 | 99,9 | 0,3568 | 30,82 | -0,1836 | 99,84 | 0,3738 | 30,93 | -0,1369 |
| 100,02 | 0,3644 | 30,86 | -0,2191 | 99,86 | 0,3568 | 30,85 | -0,1837 | 99,81 | 0,3737 | 30,96 | -0,1369 |
| 99,98 | 0,3644 | 30,89 | -0,2191 | 99,83 | 0,3568 | 30,88 | -0,1839 | 99,77 | 0,3737 | 30,99 | -0,137 |
| 99,95 | 0,3644 | 30,93 | -0,2192 | 99,8 | 0,3567 | 30,92 | -0,184 | 99,74 | 0,3737 | 31,03 | -0,137 |
| 99,92 | 0,3644 | 30,96 | -0,2192 | 99,76 | 0,3567 | 30,95 | -0,1841 | 99,71 | 0,3736 | 31,06 | -0,137 |
| 99,88 | 0,3643 | 30,99 | -0,2193 | 99,73 | 0,3567 | 30,98 | -0,1842 | 99,67 | 0,3736 | 31,09 | -0,1371 |
| 99,85 | 0,3643 | 31,02 | -0,2195 | 99,7 | 0,3567 | 31,02 | -0,1843 | 99,64 | 0,3736 | 31,13 | -0,1371 |
| 99,82 | 0,3643 | 31,06 | -0,2195 | 99,66 | 0,3566 | 31,05 | -0,1844 | 99,61 | 0,3735 | 31,16 | -0,1371 |
| 99,78 | 0,3643 | 31,09 | -0,2196 | 99,63 | 0,3566 | 31,08 | -0,1846 | 99,57 | 0,3735 | 31,19 | -0,1372 |
| 99,75 | 0,3643 | 31,13 | -0,2197 | 99,6 | 0,3566 | 31,12 | -0,1849 | 99,54 | 0,3735 | 31,23 | -0,1372 |
| 99,72 | 0,3643 | 31,16 | -0,2197 | 99,56 | 0,3566 | 31,15 | -0,1851 | 99,51 | 0,3735 | 31,26 | -0,1372 |
| 99,68 | 0,3642 | 31,19 | -0,2198 | 99,53 | 0,3565 | 31,18 | -0,1852 | 99,47 | 0,3734 | 31,29 | -0,1373 |
| 99,65 | 0,3642 | 31,23 | -0,2199 | 99,5 | 0,3565 | 31,22 | -0,1853 | 99,44 | 0,3734 | 31,33 | -0,1373 |
| 99,62 | 0,3642 | 31,26 | -0,2199 | 99,46 | 0,3565 | 31,25 | -0,1854 | 99,4 | 0,3734 | 31,36 | -0,1373 |
| 99,58 | 0,3642 | 31,29 | -0,22 | 99,43 | 0,3564 | 31,28 | -0,1855 | 99,37 | 0,3733 | 31,39 | -0,1374 |
| 99,55 | 0,3642 | 31,33 | -0,22 | 99,4 | 0,3564 | 31,32 | -0,1856 | 99,34 | 0,3733 | 31,43 | -0,1374 |
| 99,52 | 0,3642 | 31,36 | -0,2201 | 99,36 | 0,3564 | 31,35 | -0,1857 | 99,31 | 0,3733 | 31,46 | -0,1374 |
| 99,48 | 0,3642 | 31,39 | -0,2202 | 99,33 | 0,3564 | 31,38 | -0,1858 | 99,27 | 0,3732 | 31,49 | -0,1374 |
| 99,45 | 0,3641 | 31,43 | -0,2202 | 99,3 | 0,3563 | 31,42 | -0,1859 | 99,24 | 0,3732 | 31,53 | -0,1375 |
| 99,42 | 0,3641 | 31,46 | -0,2203 | 99,26 | 0,3563 | 31,45 | -0,1861 | 99,21 | 0,3732 | 31,56 | -0,1375 |
| 99,38 | 0,3641 | 31,49 | -0,2204 | 99,23 | 0,3563 | 31,48 | -0,1862 | 99,17 | 0,3731 | 31,59 | -0,1375 |
| 99,35 | 0,3641 | 31,52 | -0,2205 | 99,2 | 0,3563 | 31,52 | -0,1863 | 99,14 | 0,3731 | 31,63 | -0,1376 |
| 99,32 | 0,3641 | 31,56 | -0,2205 | 99,16 | 0,3562 | 31,55 | -0,1865 | 99,11 | 0,3731 | 31,66 | -0,1376 |
| 99,28 | 0,364 | 31,59 | -0,2206 | 99,13 | 0,3562 | 31,58 | -0,1866 | 99,07 | 0,373 | 31,69 | -0,1376 |
| 99,25 | 0,364 | 31,63 | -0,2207 | 99,1 | 0,3562 | 31,62 | -0,1868 | 99,04 | 0,373 | 31,73 | -0,1377 |
| 99,22 | 0,364 | 31,66 | -0,2207 | 99,06 | 0,3562 | 31,65 | -0,187 | 99,01 | 0,3729 | 31,76 | -0,1377 |
| 99,18 | 0,364 | 31,69 | -0,2208 | 99,03 | 0,3561 | 31,69 | -0,1871 | 98,97 | 0,3729 | 31,79 | -0,1377 |
| 99,15 | 0,364 | 31,73 | -0,2208 | 99 | 0,3561 | 31,72 | -0,1872 | 98,94 | 0,3729 | 31,83 | -0,1378 |
| 99,12 | 0,364 | 31,76 | -0,2209 | 98,96 | 0,3561 | 31,75 | -0,1874 | 98,9 | 0,3728 | 31,86 | -0,1378 |
| 99,08 | 0,3639 | 31,79 | -0,221 | 98,93 | 0,3561 | 31,78 | -0,1875 | 98,87 | 0,3728 | 31,89 | -0,1378 |
| 99,05 | 0,3639 | 31,83 | -0,2211 | 98,89 | 0,356 | 31,82 | -0,1876 | 98,84 | 0,3728 | 31,93 | -0,1379 |
| 99,02 | 0,3639 | 31,86 | -0,2211 | 98,86 | 0,356 | 31,85 | -0,1878 | 98,81 | 0,3727 | 31,96 | -0,1379 |
| 98,98 | 0,3639 | 31,89 | -0,2212 | 98,83 | 0,356 | 31,88 | -0,1879 | 98,77 | 0,3727 | 31,99 | -0,1379 |
| 98,95 | 0,3639 | 31,93 | -0,2213 | 98,79 | 0,3559 | 31,92 | -0,1881 | 98,74 | 0,3727 | 32,03 | -0,138 |
| 98,92 | 0,3639 | 31,96 | -0,2213 | 98,76 | 0,3559 | 31,95 | -0,1882 | 98,7 | 0,3726 | 32,06 | -0,138 |
| 98,88 | 0,3638 | 31,99 | -0,2214 | 98,73 | 0,3559 | 31,98 | -0,1883 | 98,67 | 0,3726 | 32,09 | -0,138 |
| 98,85 | 0,3638 | 32,03 | -0,2214 | 98,7 | 0,3559 | 32,02 | -0,1885 | 98,64 | 0,3726 | 32,13 | -0,1381 |
| 98,82 | 0,3638 | 32,06 | -0,2215 | 98,66 | 0,3558 | 32,05 | -0,1886 | 98,61 | 0,3725 | 32,16 | -0,1381 |
| 98,78 | 0,3638 | 32,09 | -0,2215 | 98,63 | 0,3558 | 32,08 | -0,1887 | 98,57 | 0,3725 | 32,19 | -0,1381 |
| 98,75 | 0,3638 | 32,13 | -0,2216 | 98,6 | 0,3558 | 32,12 | -0,1888 | 98,54 | 0,3725 | 32,23 | -0,1382 |
| 98,72 | 0,3638 | 32,16 | -0,2217 | 98,56 | 0,3558 | 32,15 | -0,1889 | 98,51 | 0,3724 | 32,26 | -0,1382 |
| 98,68 | 0,3637 | 32,19 | -0,2217 | 98,53 | 0,3557 | 32,18 | -0,189 | 98,47 | 0,3724 | 32,29 | -0,1382 |
| 98,65 | 0,3637 | 32,23 | -0,2218 | 98,5 | 0,3557 | 32,22 | -0,1891 | 98,44 | 0,3724 | 32,33 | -0,1383 |
| 98,62 | 0,3637 | 32,26 | -0,2219 | 98,46 | 0,3557 | 32,25 | -0,1892 | 98,41 | 0,3723 | 32,36 | -0,1383 |
| 98,58 | 0,3637 | 32,29 | -0,2219 | 98,43 | 0,3556 | 32,28 | -0,1893 | 98,37 | 0,3723 | 32,39 | -0,1383 |
| 98,55 | 0,3637 | 32,33 | -0,222 | 98,4 | 0,3556 | 32,32 | -0,1894 | 98,34 | 0,3723 | 32,43 | -0,1384 |
| 98,52 | 0,3636 | 32,36 | -0,222 | 98,36 | 0,3556 | 32,35 | -0,1895 | 98,31 | 0,3722 | 32,46 | -0,1384 |
| 98,48 | 0,3636 | 32,39 | -0,2221 | 98,33 | 0,3556 | 32,38 | -0,1896 | 98,27 | 0,3722 | 32,49 | -0,1384 |
| 98,45 | 0,3636 | 32,42 | -0,2221 | 98,3 | 0,3555 | 32,41 | -0,1897 | 98,24 | 0,3722 | 32,53 | -0,1385 |
| 98,42 | 0,3636 | 32,46 | -0,2222 | 98,26 | 0,3555 | 32,45 | -0,1899 | 98,21 | 0,3721 | 32,56 | -0,1385 |
| 98,38 | 0,3636 | 32,49 | -0,2223 | 98,23 | 0,3555 | 32,48 | -0,1901 | 98,17 | 0,3721 | 32,59 | -0,1385 |
| 98,35 | 0,3635 | 32,53 | -0,2223 | 98,2 | 0,3554 | 32,51 | -0,1902 | 98,14 | 0,3721 | 32,63 | -0,1386 |
| 98,32 | 0,3635 | 32,56 | -0,2224 | 98,16 | 0,3554 | 32,55 | -0,1904 | 98,11 | 0,372 | 32,66 | -0,1386 |
| 98,28 | 0,3635 | 32,59 | -0,2224 | 98,13 | 0,3554 | 32,58 | -0,1905 | 98,07 | 0,372 | 32,69 | -0,1386 |
| 98,25 | 0,3635 | 32,63 | -0,2225 | 98,1 | 0,3554 | 32,62 | -0,1906 | 98,04 | 0,372 | 32,73 | -0,1387 |
| 98,22 | 0,3635 | 32,66 | -0,2225 | 98,06 | 0,3553 | 32,65 | -0,1907 | 98,01 | 0,3719 | 32,76 | -0,1387 |
| 98,18 | 0,3634 | 32,69 | -0,2226 | 98,03 | 0,3553 | 32,68 | -0,1909 | 97,97 | 0,3719 | 32,79 | -0,1387 |
| 98,15 | 0,3634 | 32,73 | -0,2227 | 98 | 0,3553 | 32,71 | -0,1911 | 97,94 | 0,3719 | 32,83 | -0,1388 |
| 98,12 | 0,3634 | 32,76 | -0,2228 | 97,96 | 0,3553 | 32,75 | -0,1913 | 97,91 | 0,3718 | 32,86 | -0,1388 |
| 98,08 | 0,3634 | 32,79 | -0,2228 | 97,93 | 0,3552 | 32,78 | -0,1914 | 97,87 | 0,3718 | 32,89 | -0,1388 |
| 98,05 | 0,3634 | 32,83 | -0,2229 | 97,9 | 0,3552 | 32,82 | -0,1915 | 97,84 | 0,3718 | 32,93 | -0,1389 |
| 98,02 | 0,3633 | 32,86 | -0,2229 | 97,86 | 0,3552 | 32,85 | -0,1916 | 97,81 | 0,3717 | 32,96 | -0,1389 |
| 97,98 | 0,3633 | 32,89 | -0,223 | 97,83 | 0,3551 | 32,88 | -0,1917 | 97,77 | 0,3717 | 32,99 | -0,139 |
| 97,95 | 0,3633 | 32,93 | -0,2231 | 97,8 | 0,3551 | 32,92 | -0,1918 | 97,74 | 0,3716 | 33,03 | -0,139 |
| 97,92 | 0,3633 | 32,96 | -0,2231 | 97,76 | 0,3551 | 32,95 | -0,1919 | 97,71 | 0,3716 | 33,06 | -0,139 |
| 97,88 | 0,3633 | 32,99 | -0,2232 | 97,73 | 0,3551 | 32,98 | -0,192 | 97,67 | 0,3716 | 33,09 | -0,139 |
| 97,85 | 0,3633 | 33,03 | -0,2232 | 97,7 | 0,355 | 33,02 | -0,1921 | 97,64 | 0,3715 | 33,13 | -0,1391 |
| 97,82 | 0,3632 | 33,06 | -0,2233 | 97,66 | 0,355 | 33,05 | -0,1921 | 97,61 | 0,3715 | 33,16 | -0,1391 |
| 97,78 | 0,3632 | 33,09 | -0,2233 | 97,63 | 0,355 | 33,08 | -0,1922 | 97,57 | 0,3715 | 33,19 | -0,1392 |
| 97,75 | 0,3632 | 33,13 | -0,2234 | 97,6 | 0,355 | 33,12 | -0,1924 | 97,54 | 0,3714 | 33,23 | -0,1392 |
| 97,72 | 0,3632 | 33,16 | -0,2234 | 97,56 | 0,3549 | 33,15 | -0,1925 | 97,51 | 0,3714 | 33,26 | -0,1392 |
| 97,68 | 0,3632 | 33,19 | -0,2235 | 97,53 | 0,3549 | 33,18 | -0,1927 | 97,47 | 0,3714 | 33,3 | -0,1393 |
| 97,65 | 0,3631 | 33,23 | -0,2236 | 97,5 | 0,3549 | 33,21 | -0,1928 | 97,44 | 0,3713 | 33,33 | -0,1393 |
| 97,62 | 0,3631 | 33,26 | -0,2236 | 97,46 | 0,3548 | 33,25 | -0,193 | 97,41 | 0,3713 | 33,36 | -0,1393 |
| 97,58 | 0,3631 | 33,29 | -0,2237 | 97,43 | 0,3548 | 33,28 | -0,1931 | 97,37 | 0,3713 | 33,39 | -0,1394 |
| 97,55 | 0,3631 | 33,33 | -0,2237 | 97,4 | 0,3548 | 33,31 | -0,1931 | 97,34 | 0,3712 | 33,43 | -0,1394 |
| 97,52 | 0,3631 | 33,36 | -0,2238 | 97,36 | 0,3548 | 33,35 | -0,1932 | 97,31 | 0,3712 | 33,46 | -0,1394 |
| 97,48 | 0,363 | 33,39 | -0,2238 | 97,33 | 0,3547 | 33,38 | -0,1933 | 97,27 | 0,3712 | 33,49 | -0,1395 |
| 97,45 | 0,363 | 33,43 | -0,2239 | 97,3 | 0,3547 | 33,41 | -0,1934 | 97,24 | 0,3711 | 33,53 | -0,1395 |
| 97,42 | 0,363 | 33,46 | -0,224 | 97,26 | 0,3547 | 33,45 | -0,1936 | 97,21 | 0,3711 | 33,56 | -0,1395 |
| 97,38 | 0,363 | 33,49 | -0,224 | 97,23 | 0,3546 | 33,48 | -0,1937 | 97,17 | 0,3711 | 33,59 | -0,1396 |
| 97,35 | 0,363 | 33,53 | -0,2241 | 97,2 | 0,3546 | 33,51 | -0,1938 | 97,14 | 0,371 | 33,63 | -0,1396 |
| 97,32 | 0,3629 | 33,56 | -0,2241 | 97,16 | 0,3546 | 33,55 | -0,1939 | 97,11 | 0,371 | 33,66 | -0,1396 |
| 97,28 | 0,3629 | 33,59 | -0,2242 | 97,13 | 0,3546 | 33,58 | -0,194 | 97,07 | 0,3709 | 33,69 | -0,1397 |
| 97,25 | 0,3629 | 33,63 | -0,2242 | 97,1 | 0,3545 | 33,61 | -0,1942 | 97,04 | 0,3709 | 33,73 | -0,1397 |
| 97,22 | 0,3629 | 33,66 | -0,2243 | 97,06 | 0,3545 | 33,65 | -0,1943 | 97,01 | 0,3709 | 33,76 | -0,1397 |
| 97,18 | 0,3629 | 33,69 | -0,2243 | 97,03 | 0,3545 | 33,68 | -0,1945 | 96,97 | 0,3708 | 33,79 | -0,1398 |
| 97,15 | 0,3629 | 33,73 | -0,2244 | 97 | 0,3545 | 33,71 | -0,1946 | 96,94 | 0,3708 | 33,83 | -0,1398 |
| 97,12 | 0,3628 | 33,76 | -0,2245 | 96,96 | 0,3544 | 33,75 | -0,1947 | 96,91 | 0,3708 | 33,86 | -0,1399 |
| 97,08 | 0,3628 | 33,79 | -0,2245 | 96,93 | 0,3544 | 33,78 | -0,1947 | 96,87 | 0,3708 | 33,89 | -0,1399 |
| 97,05 | 0,3628 | 33,83 | -0,2246 | 96,9 | 0,3544 | 33,81 | -0,1948 | 96,84 | 0,3707 | 33,93 | -0,1399 |
| 97,02 | 0,3628 | 33,86 | -0,2246 | 96,86 | 0,3543 | 33,85 | -0,1949 | 96,81 | 0,3707 | 33,96 | -0,1399 |
| 96,98 | 0,3628 | 33,89 | -0,2247 | 96,83 | 0,3543 | 33,88 | -0,1949 | 96,77 | 0,3707 | 33,99 | -0,14 |
| 96,95 | 0,3627 | 33,93 | -0,2247 | 96,8 | 0,3543 | 33,91 | -0,195 | 96,74 | 0,3706 | 34,03 | -0,14 |
| 96,92 | 0,3627 | 33,96 | -0,2248 | 96,76 | 0,3543 | 33,95 | -0,1951 | 96,71 | 0,3706 | 34,06 | -0,1401 |
| 96,88 | 0,3627 | 33,99 | -0,2248 | 96,73 | 0,3542 | 33,98 | -0,1952 | 96,67 | 0,3706 | 34,1 | -0,1401 |
| 96,85 | 0,3627 | 34,03 | -0,2249 | 96,7 | 0,3542 | 34,01 | -0,1954 | 96,64 | 0,3705 | 34,13 | -0,1401 |
| 96,82 | 0,3627 | 34,06 | -0,225 | 96,66 | 0,3542 | 34,05 | -0,1956 | 96,61 | 0,3705 | 34,16 | -0,1402 |
| 96,79 | 0,3626 | 34,09 | -0,225 | 96,63 | 0,3542 | 34,08 | -0,1957 | 96,57 | 0,3704 | 34,19 | -0,1402 |
| 96,75 | 0,3626 | 34,13 | -0,2251 | 96,6 | 0,3541 | 34,11 | -0,1958 | 96,54 | 0,3704 | 34,23 | -0,1402 |
| 96,72 | 0,3626 | 34,16 | -0,2251 | 96,56 | 0,3541 | 34,15 | -0,1959 | 96,51 | 0,3704 | 34,26 | -0,1403 |
| 96,68 | 0,3626 | 34,19 | -0,2252 | 96,53 | 0,3541 | 34,18 | -0,1959 | 96,47 | 0,3703 | 34,29 | -0,1403 |
| 96,65 | 0,3625 | 34,23 | -0,2252 | 96,5 | 0,354 | 34,21 | -0,196 | 96,44 | 0,3703 | 34,33 | -0,1403 |
| 96,62 | 0,3625 | 34,26 | -0,2253 | 96,46 | 0,354 | 34,25 | -0,1961 | 96,41 | 0,3703 | 34,36 | -0,1404 |
| 96,58 | 0,3625 | 34,29 | -0,2253 | 96,43 | 0,354 | 34,28 | -0,1961 | 96,37 | 0,3702 | 34,4 | -0,1404 |
| 96,55 | 0,3625 | 34,33 | -0,2254 | 96,39 | 0,354 | 34,31 | -0,1962 | 96,34 | 0,3702 | 34,43 | -0,1405 |
| 96,52 | 0,3625 | 34,36 | -0,2254 | 96,36 | 0,3539 | 34,35 | -0,1963 | 96,31 | 0,3702 | 34,46 | -0,1405 |
| 96,48 | 0,3624 | 34,39 | -0,2255 | 96,33 | 0,3539 | 34,38 | -0,1964 | 96,27 | 0,3701 | 34,5 | -0,1405 |
| 96,45 | 0,3624 | 34,43 | -0,2255 | 96,3 | 0,3539 | 34,41 | -0,1965 | 96,24 | 0,3701 | 34,53 | -0,1406 |
| 96,42 | 0,3624 | 34,46 | -0,2256 | 96,26 | 0,3539 | 34,45 | -0,1967 | 96,21 | 0,3701 | 34,56 | -0,1406 |
| 96,38 | 0,3624 | 34,49 | -0,2256 | 96,23 | 0,3538 | 34,48 | -0,1968 | 96,17 | 0,37 | 34,6 | -0,1406 |
| 96,35 | 0,3624 | 34,53 | -0,2257 | 96,2 | 0,3538 | 34,51 | -0,1969 | 96,14 | 0,37 | 34,63 | -0,1407 |
| 96,32 | 0,3624 | 34,56 | -0,2258 | 96,16 | 0,3538 | 34,55 | -0,1969 | 96,11 | 0,37 | 34,66 | -0,1407 |
| 96,28 | 0,3623 | 34,59 | -0,2258 | 96,13 | 0,3537 | 34,58 | -0,197 | 96,07 | 0,3699 | 34,7 | -0,1407 |
| 96,25 | 0,3623 | 34,63 | -0,2259 | 96,09 | 0,3537 | 34,61 | -0,1972 | 96,04 | 0,3699 | 34,73 | -0,1408 |
| 96,22 | 0,3623 | 34,66 | -0,2259 | 96,06 | 0,3537 | 34,65 | -0,1973 | 96,01 | 0,3699 | 34,76 | -0,1408 |
| 96,18 | 0,3623 | 34,69 | -0,226 | 96,03 | 0,3537 | 34,68 | -0,1973 | 95,97 | 0,3698 | 34,8 | -0,1408 |
| 96,15 | 0,3623 | 34,73 | -0,226 | 96 | 0,3536 | 34,71 | -0,1974 | 95,94 | 0,3698 | 34,83 | -0,1409 |
| 96,12 | 0,3622 | 34,76 | -0,2261 | 95,96 | 0,3536 | 34,75 | -0,1974 | 95,91 | 0,3698 | 34,86 | -0,1409 |
| 96,08 | 0,3622 | 34,79 | -0,2261 | 95,93 | 0,3536 | 34,78 | -0,1975 | 95,87 | 0,3697 | 34,9 | -0,141 |
| 96,05 | 0,3622 | 34,83 | -0,2262 | 95,9 | 0,3535 | 34,81 | -0,1975 | 95,84 | 0,3697 | 34,93 | -0,141 |
| 96,02 | 0,3622 | 34,86 | -0,2262 | 95,86 | 0,3535 | 34,85 | -0,1976 | 95,81 | 0,3697 | 34,96 | -0,141 |
| 95,98 | 0,3621 | 34,89 | -0,2263 | 95,83 | 0,3535 | 34,88 | -0,1977 | 95,77 | 0,3696 | 35 | -0,1411 |
| 95,95 | 0,3621 | 34,93 | -0,2263 | 95,8 | 0,3535 | 34,91 | -0,1977 | 95,74 | 0,3696 | 35,03 | -0,1411 |
| 95,92 | 0,3621 | 34,96 | -0,2264 | 95,76 | 0,3534 | 34,95 | -0,1979 | 95,71 | 0,3696 | 35,06 | -0,1411 |
| 95,88 | 0,3621 | 34,99 | -0,2264 | 95,73 | 0,3534 | 34,98 | -0,198 | 95,67 | 0,3695 | 35,1 | -0,1412 |
| 95,85 | 0,3621 | 35,03 | -0,2265 | 95,7 | 0,3534 | 35,01 | -0,1982 | 95,64 | 0,3695 | 35,13 | -0,1412 |
| 95,82 | 0,362 | 35,06 | -0,2266 | 95,66 | 0,3534 | 35,05 | -0,1983 | 95,61 | 0,3695 | 35,16 | -0,1412 |
| 95,78 | 0,362 | 35,09 | -0,2266 | 95,63 | 0,3533 | 35,08 | -0,1985 | 95,57 | 0,3694 | 35,19 | -0,1413 |
| 95,75 | 0,362 | 35,13 | -0,2267 | 95,59 | 0,3533 | 35,11 | -0,1986 | 95,54 | 0,3694 | 35,23 | -0,1413 |
| 95,72 | 0,362 | 35,16 | -0,2267 | 95,56 | 0,3533 | 35,15 | -0,1986 | 95,51 | 0,3694 | 35,26 | -0,1413 |
| 95,68 | 0,362 | 35,19 | -0,2268 | 95,53 | 0,3533 | 35,18 | -0,1987 | 95,47 | 0,3693 | 35,3 | -0,1414 |
| 95,65 | 0,3619 | 35,23 | -0,2268 | 95,5 | 0,3532 | 35,21 | -0,1988 | 95,44 | 0,3693 | 35,33 | -0,1414 |
| 95,62 | 0,3619 | 35,26 | -0,2269 | 95,46 | 0,3532 | 35,25 | -0,1989 | 95,4 | 0,3693 | 35,36 | -0,1415 |
| 95,58 | 0,3619 | 35,29 | -0,2269 | 95,43 | 0,3532 | 35,28 | -0,199 | 95,37 | 0,3692 | 35,4 | -0,1415 |
| 95,55 | 0,3619 | 35,33 | -0,227 | 95,39 | 0,3531 | 35,31 | -0,199 | 95,34 | 0,3692 | 35,43 | -0,1415 |
| 95,52 | 0,3619 | 35,36 | -0,227 | 95,36 | 0,3531 | 35,35 | -0,1991 | 95,31 | 0,3692 | 35,46 | -0,1416 |
| 95,48 | 0,3619 | 35,39 | -0,2271 | 95,33 | 0,3531 | 35,38 | -0,1992 | 95,27 | 0,3691 | 35,5 | -0,1416 |
| 95,45 | 0,3618 | 35,43 | -0,2271 | 95,3 | 0,3531 | 35,41 | -0,1992 | 95,24 | 0,3691 | 35,53 | -0,1416 |
| 95,42 | 0,3618 | 35,46 | -0,2272 | 95,26 | 0,353 | 35,45 | -0,1993 | 95,21 | 0,3691 | 35,56 | -0,1417 |
| 95,38 | 0,3618 | 35,49 | -0,2272 | 95,23 | 0,353 | 35,48 | -0,1993 | 95,17 | 0,369 | 35,6 | -0,1417 |
| 95,35 | 0,3618 | 35,53 | -0,2273 | 95,19 | 0,353 | 35,51 | -0,1994 | 95,14 | 0,369 | 35,63 | -0,1417 |
| 95,32 | 0,3618 | 35,56 | -0,2273 | 95,16 | 0,3529 | 35,55 | -0,1994 | 95,11 | 0,369 | 35,66 | -0,1418 |
| 95,28 | 0,3617 | 35,59 | -0,2274 | 95,13 | 0,3529 | 35,58 | -0,1995 | 95,07 | 0,3689 | 35,7 | -0,1418 |
| 95,25 | 0,3617 | 35,63 | -0,2274 | 95,1 | 0,3529 | 35,61 | -0,1997 | 95,04 | 0,3689 | 35,73 | -0,1419 |
| 95,22 | 0,3617 | 35,66 | -0,2275 | 95,06 | 0,3529 | 35,65 | -0,1998 | 95,01 | 0,3689 | 35,76 | -0,1419 |
| 95,18 | 0,3617 | 35,69 | -0,2275 | 95,03 | 0,3528 | 35,68 | -0,2 | 94,97 | 0,3688 | 35,8 | -0,1419 |
| 95,15 | 0,3617 | 35,73 | -0,2276 | 95 | 0,3528 | 35,71 | -0,2001 | 94,94 | 0,3688 | 35,83 | -0,142 |
| 95,12 | 0,3616 | 35,76 | -0,2276 | 94,96 | 0,3528 | 35,75 | -0,2002 | 94,91 | 0,3688 | 35,86 | -0,142 |
| 95,08 | 0,3616 | 35,79 | -0,2277 | 94,93 | 0,3528 | 35,78 | -0,2002 | 94,87 | 0,3687 | 35,89 | -0,142 |
| 95,05 | 0,3616 | 35,83 | -0,2277 | 94,9 | 0,3527 | 35,81 | -0,2003 | 94,84 | 0,3687 | 35,93 | -0,1421 |
| 95,02 | 0,3616 | 35,86 | -0,2278 | 94,86 | 0,3527 | 35,85 | -0,2003 | 94,81 | 0,3687 | 35,96 | -0,1421 |
| 94,98 | 0,3615 | 35,89 | -0,2278 | 94,83 | 0,3527 | 35,88 | -0,2004 | 94,77 | 0,3686 | 36 | -0,1422 |
| 94,95 | 0,3615 | 35,93 | -0,2279 | 94,8 | 0,3526 | 35,91 | -0,2005 | 94,74 | 0,3686 | 36,03 | -0,1422 |
| 94,92 | 0,3615 | 35,96 | -0,2279 | 94,76 | 0,3526 | 35,95 | -0,2005 | 94,71 | 0,3686 | 36,06 | -0,1422 |
| 94,88 | 0,3615 | 35,99 | -0,228 | 94,73 | 0,3526 | 35,98 | -0,2006 | 94,67 | 0,3685 | 36,1 | -0,1423 |
| 94,85 | 0,3615 | 36,03 | -0,228 | 94,7 | 0,3526 | 36,01 | -0,2007 | 94,64 | 0,3685 | 36,13 | -0,1423 |
| 94,82 | 0,3614 | 36,06 | -0,2281 | 94,66 | 0,3525 | 36,05 | -0,2008 | 94,61 | 0,3685 | 36,16 | -0,1424 |
| 94,78 | 0,3614 | 36,09 | -0,2281 | 94,63 | 0,3525 | 36,08 | -0,2009 | 94,57 | 0,3684 | 36,2 | -0,1424 |
| 94,75 | 0,3614 | 36,13 | -0,2282 | 94,6 | 0,3525 | 36,11 | -0,201 | 94,54 | 0,3684 | 36,23 | -0,1424 |
| 94,72 | 0,3614 | 36,16 | -0,2282 | 94,56 | 0,3525 | 36,15 | -0,2011 | 94,51 | 0,3684 | 36,26 | -0,1425 |
| 94,68 | 0,3614 | 36,19 | -0,2283 | 94,53 | 0,3524 | 36,18 | -0,2012 | 94,47 | 0,3683 | 36,3 | -0,1425 |
| 94,65 | 0,3613 | 36,23 | -0,2283 | 94,5 | 0,3524 | 36,21 | -0,2012 | 94,44 | 0,3683 | 36,33 | -0,1425 |
| 94,62 | 0,3613 | 36,26 | -0,2284 | 94,46 | 0,3524 | 36,25 | -0,2013 | 94,41 | 0,3683 | 36,36 | -0,1426 |
| 94,58 | 0,3613 | 36,29 | -0,2284 | 94,43 | 0,3523 | 36,28 | -0,2014 | 94,37 | 0,3682 | 36,4 | -0,1426 |
| 94,55 | 0,3613 | 36,33 | -0,2285 | 94,4 | 0,3523 | 36,31 | -0,2014 | 94,34 | 0,3682 | 36,43 | -0,1427 |
| 94,52 | 0,3613 | 36,36 | -0,2285 | 94,36 | 0,3523 | 36,35 | -0,2015 | 94,3 | 0,3682 | 36,46 | -0,1427 |
| 94,48 | 0,3612 | 36,4 | -0,2286 | 94,33 | 0,3523 | 36,38 | -0,2015 | 94,27 | 0,3681 | 36,5 | -0,1427 |
| 94,45 | 0,3612 | 36,43 | -0,2286 | 94,29 | 0,3522 | 36,41 | -0,2016 | 94,24 | 0,3681 | 36,53 | -0,1428 |
| 94,42 | 0,3612 | 36,46 | -0,2287 | 94,26 | 0,3522 | 36,44 | -0,2017 | 94,21 | 0,3681 | 36,56 | -0,1428 |
| 94,38 | 0,3612 | 36,49 | -0,2287 | 94,23 | 0,3522 | 36,48 | -0,2017 | 94,17 | 0,368 | 36,6 | -0,1428 |
| 94,35 | 0,3611 | 36,53 | -0,2288 | 94,2 | 0,3521 | 36,51 | -0,2018 | 94,14 | 0,368 | 36,63 | -0,1429 |
| 94,32 | 0,3611 | 36,56 | -0,2288 | 94,16 | 0,3521 | 36,55 | -0,2018 | 94,11 | 0,368 | 36,66 | -0,1429 |
| 94,28 | 0,3611 | 36,59 | -0,2289 | 94,13 | 0,3521 | 36,58 | -0,2019 | 94,07 | 0,3679 | 36,7 | -0,143 |
| 94,25 | 0,3611 | 36,63 | -0,2289 | 94,1 | 0,3521 | 36,61 | -0,202 | 94,04 | 0,3679 | 36,73 | -0,143 |
| 94,22 | 0,3611 | 36,66 | -0,229 | 94,06 | 0,352 | 36,64 | -0,202 | 94,01 | 0,3678 | 36,76 | -0,143 |
| 94,18 | 0,361 | 36,7 | -0,229 | 94,03 | 0,352 | 36,68 | -0,2021 | 93,97 | 0,3678 | 36,8 | -0,1431 |
| 94,15 | 0,361 | 36,73 | -0,2291 | 94 | 0,352 | 36,71 | -0,2022 | 93,94 | 0,3678 | 36,83 | -0,1431 |
| 94,12 | 0,361 | 36,76 | -0,2291 | 93,96 | 0,3519 | 36,75 | -0,2023 | 93,91 | 0,3677 | 36,86 | -0,1432 |
| 94,08 | 0,361 | 36,8 | -0,2291 | 93,93 | 0,3519 | 36,78 | -0,2024 | 93,87 | 0,3677 | 36,9 | -0,1432 |
| 94,05 | 0,361 | 36,83 | -0,2292 | 93,9 | 0,3519 | 36,81 | -0,2024 | 93,84 | 0,3677 | 36,93 | -0,1432 |
| 94,02 | 0,3609 | 36,86 | -0,2292 | 93,86 | 0,3519 | 36,85 | -0,2025 | 93,81 | 0,3676 | 36,96 | -0,1433 |
| 93,98 | 0,3609 | 36,9 | -0,2293 | 93,83 | 0,3518 | 36,88 | -0,2025 | 93,77 | 0,3676 | 37 | -0,1433 |
| 93,95 | 0,3609 | 36,93 | -0,2293 | 93,8 | 0,3518 | 36,91 | -0,2026 | 93,74 | 0,3676 | 37,03 | -0,1433 |
| 93,92 | 0,3609 | 36,96 | -0,2294 | 93,76 | 0,3518 | 36,94 | -0,2026 | 93,71 | 0,3675 | 37,06 | -0,1434 |
| 93,88 | 0,3608 | 37 | -0,2294 | 93,73 | 0,3517 | 36,98 | -0,2027 | 93,67 | 0,3675 | 37,1 | -0,1434 |
| 93,85 | 0,3608 | 37,03 | -0,2295 | 93,7 | 0,3517 | 37,01 | -0,2027 | 93,64 | 0,3675 | 37,13 | -0,1435 |
| 93,82 | 0,3608 | 37,06 | -0,2295 | 93,66 | 0,3517 | 37,05 | -0,2028 | 93,61 | 0,3674 | 37,16 | -0,1435 |
| 93,78 | 0,3608 | 37,1 | -0,2296 | 93,63 | 0,3517 | 37,08 | -0,2029 | 93,57 | 0,3674 | 37,2 | -0,1435 |
| 93,75 | 0,3608 | 37,13 | -0,2296 | 93,6 | 0,3516 | 37,11 | -0,2029 | 93,54 | 0,3674 | 37,23 | -0,1436 |
| 93,72 | 0,3607 | 37,16 | -0,2297 | 93,56 | 0,3516 | 37,15 | -0,203 | 93,51 | 0,3674 | 37,26 | -0,1436 |
| 93,68 | 0,3607 | 37,19 | -0,2297 | 93,53 | 0,3516 | 37,18 | -0,2031 | 93,47 | 0,3673 | 37,3 | -0,1437 |
| 93,65 | 0,3607 | 37,23 | -0,2297 | 93,5 | 0,3516 | 37,21 | -0,2032 | 93,44 | 0,3673 | 37,33 | -0,1437 |
| 93,62 | 0,3607 | 37,26 | -0,2298 | 93,46 | 0,3515 | 37,25 | -0,2032 | 93,41 | 0,3672 | 37,36 | -0,1437 |
| 93,58 | 0,3606 | 37,29 | -0,2298 | 93,43 | 0,3515 | 37,28 | -0,2033 | 93,37 | 0,3672 | 37,4 | -0,1438 |
| 93,55 | 0,3606 | 37,33 | -0,2299 | 93,39 | 0,3515 | 37,31 | -0,2033 | 93,34 | 0,3672 | 37,43 | -0,1438 |
| 93,52 | 0,3606 | 37,36 | -0,2299 | 93,36 | 0,3514 | 37,35 | -0,2034 | 93,31 | 0,3671 | 37,46 | -0,1438 |
| 93,48 | 0,3606 | 37,4 | -0,23 | 93,33 | 0,3514 | 37,38 | -0,2035 | 93,27 | 0,3671 | 37,5 | -0,1439 |
| 93,45 | 0,3606 | 37,43 | -0,23 | 93,3 | 0,3514 | 37,41 | -0,2035 | 93,24 | 0,3671 | 37,53 | -0,1439 |
| 93,42 | 0,3605 | 37,46 | -0,2301 | 93,26 | 0,3513 | 37,45 | -0,2036 | 93,21 | 0,367 | 37,56 | -0,144 |
| 93,38 | 0,3605 | 37,5 | -0,2301 | 93,23 | 0,3513 | 37,48 | -0,2037 | 93,17 | 0,367 | 37,6 | -0,144 |
| 93,35 | 0,3605 | 37,53 | -0,2302 | 93,19 | 0,3513 | 37,51 | -0,2037 | 93,14 | 0,367 | 37,63 | -0,144 |
| 93,32 | 0,3605 | 37,56 | -0,2302 | 93,16 | 0,3513 | 37,55 | -0,2038 | 93,11 | 0,3669 | 37,66 | -0,1441 |
| 93,28 | 0,3604 | 37,6 | -0,2303 | 93,13 | 0,3512 | 37,58 | -0,2038 | 93,07 | 0,3669 | 37,7 | -0,1441 |
| 93,25 | 0,3604 | 37,63 | -0,2303 | 93,09 | 0,3512 | 37,61 | -0,2039 | 93,04 | 0,3669 | 37,73 | -0,1442 |
| 93,22 | 0,3604 | 37,66 | -0,2303 | 93,06 | 0,3512 | 37,65 | -0,2039 | 93,01 | 0,3668 | 37,76 | -0,1442 |
| 93,18 | 0,3604 | 37,7 | -0,2304 | 93,03 | 0,3511 | 37,68 | -0,204 | 92,97 | 0,3668 | 37,8 | -0,1442 |
| 93,15 | 0,3604 | 37,73 | -0,2304 | 92,99 | 0,3511 | 37,71 | -0,2041 | 92,94 | 0,3668 | 37,83 | -0,1443 |
| 93,12 | 0,3603 | 37,76 | -0,2305 | 92,96 | 0,3511 | 37,75 | -0,2041 | 92,91 | 0,3667 | 37,86 | -0,1443 |
| 93,08 | 0,3603 | 37,8 | -0,2305 | 92,93 | 0,3511 | 37,78 | -0,2042 | 92,87 | 0,3667 | 37,9 | -0,1443 |
| 93,05 | 0,3603 | 37,83 | -0,2306 | 92,9 | 0,351 | 37,81 | -0,2042 | 92,84 | 0,3667 | 37,93 | -0,1444 |
| 93,02 | 0,3603 | 37,86 | -0,2306 | 92,86 | 0,351 | 37,85 | -0,2043 | 92,81 | 0,3666 | 37,96 | -0,1444 |
| 92,98 | 0,3602 | 37,9 | -0,2307 | 92,83 | 0,351 | 37,88 | -0,2043 | 92,77 | 0,3666 | 38 | -0,1445 |
| 92,95 | 0,3602 | 37,93 | -0,2307 | 92,79 | 0,3509 | 37,91 | -0,2044 | 92,74 | 0,3666 | 38,03 | -0,1445 |
| 92,92 | 0,3602 | 37,96 | -0,2307 | 92,76 | 0,3509 | 37,95 | -0,2045 | 92,7 | 0,3665 | 38,06 | -0,1445 |
| 92,88 | 0,3602 | 38 | -0,2308 | 92,73 | 0,3509 | 37,98 | -0,2045 | 92,67 | 0,3665 | 38,1 | -0,1446 |
| 92,85 | 0,3601 | 38,03 | -0,2308 | 92,69 | 0,3509 | 38,01 | -0,2046 | 92,64 | 0,3665 | 38,13 | -0,1446 |
| 92,82 | 0,3601 | 38,06 | -0,2309 | 92,66 | 0,3508 | 38,04 | -0,2046 | 92,61 | 0,3664 | 38,16 | -0,1447 |
| 92,78 | 0,3601 | 38,1 | -0,2309 | 92,63 | 0,3508 | 38,08 | -0,2047 | 92,57 | 0,3664 | 38,2 | -0,1447 |
| 92,75 | 0,3601 | 38,13 | -0,231 | 92,59 | 0,3508 | 38,11 | -0,2047 | 92,54 | 0,3664 | 38,23 | -0,1447 |
| 92,72 | 0,36 | 38,16 | -0,231 | 92,56 | 0,3507 | 38,15 | -0,2048 | 92,51 | 0,3663 | 38,26 | -0,1448 |
| 92,68 | 0,36 | 38,2 | -0,2311 | 92,53 | 0,3507 | 38,18 | -0,2049 | 92,47 | 0,3663 | 38,3 | -0,1448 |
| 92,65 | 0,36 | 38,23 | -0,2311 | 92,49 | 0,3507 | 38,21 | -0,2049 | 92,44 | 0,3663 | 38,33 | -0,1449 |
| 92,62 | 0,36 | 38,26 | -0,2311 | 92,46 | 0,3507 | 38,24 | -0,205 | 92,41 | 0,3662 | 38,36 | -0,1449 |
| 92,58 | 0,36 | 38,3 | -0,2312 | 92,43 | 0,3506 | 38,28 | -0,2051 | 92,37 | 0,3662 | 38,4 | -0,145 |
| 92,55 | 0,3599 | 38,33 | -0,2312 | 92,39 | 0,3506 | 38,31 | -0,2051 | 92,34 | 0,3662 | 38,43 | -0,145 |
| 92,52 | 0,3599 | 38,36 | -0,2313 | 92,36 | 0,3506 | 38,34 | -0,2052 | 92,3 | 0,3661 | 38,46 | -0,145 |
| 92,48 | 0,3599 | 38,4 | -0,2313 | 92,33 | 0,3505 | 38,38 | -0,2053 | 92,27 | 0,3661 | 38,5 | -0,1451 |
| 92,45 | 0,3598 | 38,43 | -0,2314 | 92,29 | 0,3505 | 38,41 | -0,2053 | 92,24 | 0,3661 | 38,53 | -0,1451 |
| 92,42 | 0,3598 | 38,46 | -0,2314 | 92,26 | 0,3505 | 38,44 | -0,2054 | 92,21 | 0,366 | 38,56 | -0,1451 |
| 92,38 | 0,3598 | 38,5 | -0,2314 | 92,23 | 0,3504 | 38,48 | -0,2054 | 92,17 | 0,366 | 38,6 | -0,1452 |
| 92,35 | 0,3598 | 38,53 | -0,2315 | 92,19 | 0,3504 | 38,51 | -0,2055 | 92,14 | 0,366 | 38,63 | -0,1452 |
| 92,32 | 0,3598 | 38,56 | -0,2315 | 92,16 | 0,3504 | 38,55 | -0,2055 | 92,11 | 0,3659 | 38,66 | -0,1453 |
| 92,28 | 0,3597 | 38,6 | -0,2316 | 92,13 | 0,3504 | 38,58 | -0,2056 | 92,07 | 0,3659 | 38,7 | -0,1453 |
| 92,25 | 0,3597 | 38,63 | -0,2316 | 92,1 | 0,3503 | 38,61 | -0,2056 | 92,04 | 0,3658 | 38,73 | -0,1453 |
| 92,22 | 0,3597 | 38,66 | -0,2317 | 92,06 | 0,3503 | 38,65 | -0,2057 | 92,01 | 0,3658 | 38,76 | -0,1454 |
| 92,18 | 0,3597 | 38,7 | -0,2317 | 92,03 | 0,3503 | 38,68 | -0,2058 | 91,97 | 0,3658 | 38,8 | -0,1454 |
| 92,15 | 0,3596 | 38,73 | -0,2317 | 91,99 | 0,3502 | 38,71 | -0,2058 | 91,94 | 0,3657 | 38,83 | -0,1455 |
| 92,12 | 0,3596 | 38,76 | -0,2318 | 91,96 | 0,3502 | 38,75 | -0,2059 | 91,9 | 0,3657 | 38,86 | -0,1455 |
| 92,08 | 0,3596 | 38,8 | -0,2318 | 91,93 | 0,3502 | 38,78 | -0,2059 | 91,87 | 0,3657 | 38,9 | -0,1455 |
| 92,05 | 0,3596 | 38,83 | -0,2319 | 91,9 | 0,3502 | 38,81 | -0,206 | 91,84 | 0,3656 | 38,93 | -0,1456 |
| 92,02 | 0,3595 | 38,86 | -0,2319 | 91,86 | 0,3501 | 38,85 | -0,206 | 91,81 | 0,3656 | 38,96 | -0,1456 |
| 91,98 | 0,3595 | 38,9 | -0,2319 | 91,83 | 0,3501 | 38,88 | -0,2061 | 91,77 | 0,3656 | 39 | -0,1457 |
| 91,95 | 0,3595 | 38,93 | -0,232 | 91,79 | 0,3501 | 38,91 | -0,2061 | 91,74 | 0,3655 | 39,03 | -0,1457 |
| 91,92 | 0,3595 | 38,96 | -0,232 | 91,76 | 0,35 | 38,94 | -0,2062 | 91,71 | 0,3655 | 39,06 | -0,1457 |
| 91,88 | 0,3594 | 39 | -0,2321 | 91,73 | 0,35 | 38,98 | -0,2062 | 91,67 | 0,3655 | 39,1 | -0,1458 |
| 91,85 | 0,3594 | 39,03 | -0,2321 | 91,69 | 0,35 | 39,01 | -0,2063 | 91,64 | 0,3654 | 39,13 | -0,1458 |
| 91,82 | 0,3594 | 39,06 | -0,2322 | 91,66 | 0,3499 | 39,05 | -0,2064 | 91,61 | 0,3654 | 39,16 | -0,1459 |
| 91,78 | 0,3594 | 39,1 | -0,2322 | 91,63 | 0,3499 | 39,08 | -0,2064 | 91,57 | 0,3654 | 39,2 | -0,1459 |
| 91,75 | 0,3593 | 39,13 | -0,2322 | 91,59 | 0,3499 | 39,11 | -0,2065 | 91,54 | 0,3653 | 39,23 | -0,146 |
| 91,72 | 0,3593 | 39,16 | -0,2323 | 91,56 | 0,3499 | 39,15 | -0,2065 | 91,5 | 0,3653 | 39,26 | -0,146 |
| 91,68 | 0,3593 | 39,2 | -0,2323 | 91,53 | 0,3498 | 39,18 | -0,2066 | 91,47 | 0,3652 | 39,3 | -0,146 |
| 91,65 | 0,3593 | 39,23 | -0,2324 | 91,49 | 0,3498 | 39,21 | -0,2066 | 91,44 | 0,3652 | 39,33 | -0,1461 |
| 91,62 | 0,3592 | 39,26 | -0,2324 | 91,46 | 0,3498 | 39,25 | -0,2067 | 91,4 | 0,3652 | 39,36 | -0,1461 |
| 91,58 | 0,3592 | 39,3 | -0,2324 | 91,43 | 0,3497 | 39,28 | -0,2067 | 91,37 | 0,3651 | 39,4 | -0,1462 |
| 91,55 | 0,3592 | 39,33 | -0,2325 | 91,39 | 0,3497 | 39,31 | -0,2068 | 91,34 | 0,3651 | 39,43 | -0,1462 |
| 91,52 | 0,3592 | 39,36 | -0,2325 | 91,36 | 0,3497 | 39,35 | -0,2068 | 91,3 | 0,3651 | 39,46 | -0,1462 |
| 91,48 | 0,3591 | 39,4 | -0,2326 | 91,33 | 0,3496 | 39,38 | -0,2069 | 91,27 | 0,365 | 39,5 | -0,1463 |
| 91,45 | 0,3591 | 39,43 | -0,2326 | 91,29 | 0,3496 | 39,41 | -0,207 | 91,24 | 0,365 | 39,53 | -0,1463 |
| 91,42 | 0,3591 | 39,46 | -0,2326 | 91,26 | 0,3496 | 39,45 | -0,207 | 91,2 | 0,365 | 39,56 | -0,1464 |
| 91,38 | 0,359 | 39,5 | -0,2327 | 91,23 | 0,3495 | 39,48 | -0,2071 | 91,17 | 0,3649 | 39,6 | -0,1464 |
| 91,35 | 0,359 | 39,53 | -0,2327 | 91,19 | 0,3495 | 39,51 | -0,2071 | 91,14 | 0,3649 | 39,63 | -0,1464 |
| 91,32 | 0,359 | 39,56 | -0,2328 | 91,16 | 0,3495 | 39,55 | -0,2072 | 91,1 | 0,3649 | 39,66 | -0,1465 |
| 91,28 | 0,359 | 39,6 | -0,2328 | 91,13 | 0,3494 | 39,58 | -0,2072 | 91,07 | 0,3648 | 39,7 | -0,1465 |
| 91,25 | 0,3589 | 39,63 | -0,2328 | 91,09 | 0,3494 | 39,61 | -0,2073 | 91,04 | 0,3648 | 39,73 | -0,1466 |
| 91,22 | 0,3589 | 39,66 | -0,2329 | 91,06 | 0,3494 | 39,65 | -0,2073 | 91 | 0,3647 | 39,76 | -0,1466 |
| 91,18 | 0,3589 | 39,7 | -0,2329 | 91,03 | 0,3494 | 39,68 | -0,2074 | 90,97 | 0,3647 | 39,8 | -0,1467 |
| 91,15 | 0,3589 | 39,73 | -0,233 | 90,99 | 0,3493 | 39,71 | -0,2074 | 90,94 | 0,3647 | 39,83 | -0,1467 |
| 91,12 | 0,3588 | 39,76 | -0,233 | 90,96 | 0,3493 | 39,75 | -0,2075 | 90,9 | 0,3646 | 39,86 | -0,1467 |
| 91,08 | 0,3588 | 39,8 | -0,233 | 90,93 | 0,3493 | 39,78 | -0,2075 | 90,87 | 0,3646 | 39,9 | -0,1468 |
| 91,05 | 0,3588 | 39,83 | -0,2331 | 90,89 | 0,3492 | 39,81 | -0,2076 | 90,84 | 0,3645 | 39,93 | -0,1468 |
| 91,02 | 0,3588 | 39,86 | -0,2331 | 90,86 | 0,3492 | 39,85 | -0,2077 | 90,8 | 0,3645 | 39,96 | -0,1469 |
| 90,98 | 0,3587 | 39,9 | -0,2332 | 90,83 | 0,3492 | 39,88 | -0,2077 | 90,77 | 0,3645 | 40 | -0,1469 |
| 90,95 | 0,3587 | 39,93 | -0,2332 | 90,79 | 0,3491 | 39,91 | -0,2078 | 90,74 | 0,3644 | 40,03 | -0,1469 |
| 90,92 | 0,3587 | 39,96 | -0,2332 | 90,76 | 0,3491 | 39,95 | -0,2078 | 90,7 | 0,3644 | 40,06 | -0,147 |
| 90,88 | 0,3586 | 40 | -0,2333 | 90,73 | 0,3491 | 39,98 | -0,2079 | 90,67 | 0,3644 | 40,1 | -0,147 |
| 90,85 | 0,3586 | 40,03 | -0,2333 | 90,69 | 0,349 | 40,01 | -0,2079 | 90,64 | 0,3643 | 40,13 | -0,1471 |
| 90,82 | 0,3586 | 40,06 | -0,2334 | 90,66 | 0,349 | 40,05 | -0,208 | 90,6 | 0,3643 | 40,16 | -0,1471 |
| 90,78 | 0,3586 | 40,1 | -0,2334 | 90,63 | 0,349 | 40,08 | -0,208 | 90,57 | 0,3643 | 40,2 | -0,1472 |
| 90,75 | 0,3585 | 40,13 | -0,2334 | 90,59 | 0,3489 | 40,11 | -0,2081 | 90,54 | 0,3642 | 40,23 | -0,1472 |
| 90,72 | 0,3585 | 40,16 | -0,2335 | 90,56 | 0,3489 | 40,15 | -0,2081 | 90,5 | 0,3642 | 40,26 | -0,1472 |
| 90,68 | 0,3585 | 40,2 | -0,2335 | 90,53 | 0,3489 | 40,18 | -0,2082 | 90,47 | 0,3641 | 40,3 | -0,1473 |
| 90,65 | 0,3585 | 40,23 | -0,2335 | 90,49 | 0,3489 | 40,21 | -0,2082 | 90,44 | 0,3641 | 40,33 | -0,1473 |
| 90,62 | 0,3584 | 40,26 | -0,2336 | 90,46 | 0,3488 | 40,25 | -0,2083 | 90,4 | 0,3641 | 40,36 | -0,1474 |
| 90,58 | 0,3584 | 40,3 | -0,2336 | 90,43 | 0,3488 | 40,28 | -0,2083 | 90,37 | 0,364 | 40,4 | -0,1474 |
| 90,55 | 0,3584 | 40,33 | -0,2337 | 90,39 | 0,3488 | 40,31 | -0,2084 | 90,34 | 0,364 | 40,43 | -0,1474 |
| 90,52 | 0,3583 | 40,36 | -0,2337 | 90,36 | 0,3487 | 40,35 | -0,2084 | 90,3 | 0,364 | 40,46 | -0,1475 |
| 90,48 | 0,3583 | 40,4 | -0,2337 | 90,33 | 0,3487 | 40,38 | -0,2085 | 90,27 | 0,3639 | 40,5 | -0,1475 |
| 90,45 | 0,3583 | 40,43 | -0,2338 | 90,29 | 0,3487 | 40,41 | -0,2085 | 90,24 | 0,3639 | 40,53 | -0,1476 |
| 90,42 | 0,3583 | 40,46 | -0,2338 | 90,26 | 0,3486 | 40,45 | -0,2086 | 90,2 | 0,3639 | 40,56 | -0,1476 |
| 90,38 | 0,3582 | 40,5 | -0,2338 | 90,23 | 0,3486 | 40,48 | -0,2086 | 90,17 | 0,3638 | 40,6 | -0,1477 |
| 90,35 | 0,3582 | 40,53 | -0,2339 | 90,19 | 0,3486 | 40,51 | -0,2087 | 90,14 | 0,3638 | 40,63 | -0,1477 |
| 90,32 | 0,3582 | 40,57 | -0,2339 | 90,16 | 0,3485 | 40,55 | -0,2087 | 90,1 | 0,3637 | 40,66 | -0,1477 |
| 90,28 | 0,3582 | 40,6 | -0,234 | 90,13 | 0,3485 | 40,58 | -0,2088 | 90,07 | 0,3637 | 40,7 | -0,1478 |
| 90,25 | 0,3581 | 40,63 | -0,234 | 90,09 | 0,3485 | 40,61 | -0,2088 | 90,04 | 0,3637 | 40,73 | -0,1478 |
| 90,22 | 0,3581 | 40,67 | -0,234 | 90,06 | 0,3484 | 40,65 | -0,2089 | 90 | 0,3636 | 40,76 | -0,1479 |
| 90,18 | 0,3581 | 40,7 | -0,2341 | 90,03 | 0,3484 | 40,68 | -0,2089 | 89,97 | 0,3636 | 40,8 | -0,1479 |
| 90,15 | 0,358 | 40,73 | -0,2341 | 89,99 | 0,3484 | 40,71 | -0,209 | 89,94 | 0,3636 | 40,83 | -0,148 |
| 90,12 | 0,358 | 40,77 | -0,2341 | 89,96 | 0,3483 | 40,75 | -0,2091 | 89,9 | 0,3635 | 40,87 | -0,148 |
| 90,08 | 0,358 | 40,8 | -0,2342 | 89,93 | 0,3483 | 40,78 | -0,2091 | 89,87 | 0,3635 | 40,9 | -0,148 |
| 90,05 | 0,358 | 40,83 | -0,2342 | 89,89 | 0,3483 | 40,81 | -0,2092 | 89,84 | 0,3635 | 40,93 | -0,1481 |
| 90,02 | 0,3579 | 40,87 | -0,2342 | 89,86 | 0,3482 | 40,85 | -0,2092 | 89,8 | 0,3634 | 40,96 | -0,1481 |
| 89,98 | 0,3579 | 40,9 | -0,2343 | 89,83 | 0,3482 | 40,88 | -0,2093 | 89,77 | 0,3634 | 41 | -0,1482 |
| 89,95 | 0,3579 | 40,93 | -0,2343 | 89,79 | 0,3482 | 40,91 | -0,2093 | 89,74 | 0,3633 | 41,03 | -0,1482 |
| 89,92 | 0,3579 | 40,96 | -0,2344 | 89,76 | 0,3481 | 40,95 | -0,2094 | 89,7 | 0,3633 | 41,07 | -0,1483 |
| 89,88 | 0,3578 | 41 | -0,2344 | 89,73 | 0,3481 | 40,98 | -0,2094 | 89,67 | 0,3633 | 41,1 | -0,1483 |
| 89,85 | 0,3578 | 41,03 | -0,2344 | 89,69 | 0,3481 | 41,01 | -0,2095 | 89,64 | 0,3632 | 41,13 | -0,1483 |
| 89,82 | 0,3578 | 41,07 | -0,2345 | 89,66 | 0,3481 | 41,05 | -0,2095 | 89,6 | 0,3632 | 41,17 | -0,1484 |
| 89,78 | 0,3577 | 41,1 | -0,2345 | 89,63 | 0,348 | 41,08 | -0,2096 | 89,57 | 0,3632 | 41,2 | -0,1484 |
| 89,75 | 0,3577 | 41,13 | -0,2345 | 89,59 | 0,348 | 41,11 | -0,2096 | 89,53 | 0,3631 | 41,23 | -0,1485 |
| 89,72 | 0,3577 | 41,17 | -0,2346 | 89,56 | 0,348 | 41,15 | -0,2097 | 89,5 | 0,3631 | 41,27 | -0,1485 |
| 89,68 | 0,3577 | 41,2 | -0,2346 | 89,53 | 0,3479 | 41,18 | -0,2097 | 89,47 | 0,363 | 41,3 | -0,1486 |
| 89,65 | 0,3576 | 41,23 | -0,2346 | 89,49 | 0,3479 | 41,21 | -0,2097 | 89,44 | 0,363 | 41,33 | -0,1486 |
| 89,62 | 0,3576 | 41,26 | -0,2347 | 89,46 | 0,3479 | 41,25 | -0,2098 | 89,4 | 0,363 | 41,36 | -0,1486 |
| 89,58 | 0,3576 | 41,3 | -0,2347 | 89,43 | 0,3478 | 41,28 | -0,2098 | 89,37 | 0,3629 | 41,4 | -0,1487 |
| 89,55 | 0,3575 | 41,33 | -0,2347 | 89,39 | 0,3478 | 41,31 | -0,2099 | 89,34 | 0,3629 | 41,43 | -0,1487 |
| 89,52 | 0,3575 | 41,37 | -0,2348 | 89,36 | 0,3478 | 41,35 | -0,2099 | 89,3 | 0,3629 | 41,46 | -0,1488 |
| 89,48 | 0,3575 | 41,4 | -0,2348 | 89,33 | 0,3477 | 41,38 | -0,21 | 89,27 | 0,3628 | 41,5 | -0,1488 |
| 89,45 | 0,3575 | 41,43 | -0,2348 | 89,29 | 0,3477 | 41,41 | -0,21 | 89,24 | 0,3628 | 41,53 | -0,1489 |
| 89,42 | 0,3574 | 41,46 | -0,2349 | 89,26 | 0,3477 | 41,45 | -0,2101 | 89,2 | 0,3627 | 41,56 | -0,1489 |
| 89,38 | 0,3574 | 41,5 | -0,2349 | 89,23 | 0,3476 | 41,48 | -0,2101 | 89,17 | 0,3627 | 41,6 | -0,1489 |
| 89,35 | 0,3574 | 41,53 | -0,235 | 89,19 | 0,3476 | 41,51 | -0,2102 | 89,13 | 0,3627 | 41,63 | -0,149 |
| 89,32 | 0,3573 | 41,57 | -0,235 | 89,16 | 0,3476 | 41,55 | -0,2102 | 89,1 | 0,3626 | 41,67 | -0,149 |
| 89,28 | 0,3573 | 41,6 | -0,235 | 89,13 | 0,3475 | 41,58 | -0,2103 | 89,07 | 0,3626 | 41,7 | -0,1491 |
| 89,25 | 0,3573 | 41,63 | -0,2351 | 89,09 | 0,3475 | 41,61 | -0,2103 | 89,04 | 0,3625 | 41,73 | -0,1491 |
| 89,22 | 0,3572 | 41,66 | -0,2351 | 89,06 | 0,3475 | 41,65 | -0,2104 | 89 | 0,3625 | 41,76 | -0,1492 |
| 89,18 | 0,3572 | 41,7 | -0,2351 | 89,03 | 0,3474 | 41,68 | -0,2104 | 88,97 | 0,3625 | 41,8 | -0,1492 |
| 89,15 | 0,3572 | 41,73 | -0,2352 | 88,99 | 0,3474 | 41,71 | -0,2105 | 88,94 | 0,3624 | 41,83 | -0,1492 |
| 89,12 | 0,3572 | 41,77 | -0,2352 | 88,96 | 0,3474 | 41,75 | -0,2105 | 88,9 | 0,3624 | 41,87 | -0,1493 |
| 89,08 | 0,3571 | 41,8 | -0,2352 | 88,93 | 0,3473 | 41,78 | -0,2106 | 88,87 | 0,3624 | 41,9 | -0,1493 |
| 89,05 | 0,3571 | 41,83 | -0,2353 | 88,89 | 0,3473 | 41,81 | -0,2106 | 88,84 | 0,3623 | 41,93 | -0,1494 |
| 89,02 | 0,3571 | 41,87 | -0,2353 | 88,86 | 0,3473 | 41,85 | -0,2106 | 88,8 | 0,3623 | 41,97 | -0,1494 |
| 88,98 | 0,357 | 41,9 | -0,2353 | 88,83 | 0,3472 | 41,88 | -0,2107 | 88,77 | 0,3622 | 42 | -0,1495 |
| 88,95 | 0,357 | 41,93 | -0,2354 | 88,79 | 0,3472 | 41,91 | -0,2107 | 88,74 | 0,3622 | 42,03 | -0,1495 |
| 88,92 | 0,357 | 41,96 | -0,2354 | 88,76 | 0,3472 | 41,95 | -0,2108 | 88,7 | 0,3622 | 42,06 | -0,1496 |
| 88,88 | 0,357 | 42 | -0,2354 | 88,73 | 0,3471 | 41,98 | -0,2108 | 88,67 | 0,3621 | 42,1 | -0,1496 |
| 88,85 | 0,3569 | 42,03 | -0,2355 | 88,69 | 0,3471 | 42,01 | -0,2109 | 88,64 | 0,3621 | 42,13 | -0,1496 |
| 88,82 | 0,3569 | 42,07 | -0,2355 | 88,66 | 0,3471 | 42,05 | -0,2109 | 88,6 | 0,3621 | 42,16 | -0,1497 |
| 88,78 | 0,3569 | 42,1 | -0,2355 | 88,63 | 0,347 | 42,08 | -0,211 | 88,57 | 0,362 | 42,2 | -0,1497 |
| 88,75 | 0,3568 | 42,13 | -0,2356 | 88,59 | 0,347 | 42,11 | -0,211 | 88,54 | 0,362 | 42,23 | -0,1498 |
| 88,72 | 0,3568 | 42,17 | -0,2356 | 88,56 | 0,347 | 42,15 | -0,2111 | 88,5 | 0,3619 | 42,27 | -0,1498 |
| 88,68 | 0,3568 | 42,2 | -0,2356 | 88,53 | 0,3469 | 42,18 | -0,2111 | 88,47 | 0,3619 | 42,3 | -0,1499 |
| 88,65 | 0,3568 | 42,23 | -0,2357 | 88,49 | 0,3469 | 42,21 | -0,2111 | 88,44 | 0,3619 | 42,33 | -0,1499 |
| 88,62 | 0,3567 | 42,27 | -0,2357 | 88,46 | 0,3469 | 42,25 | -0,2112 | 88,4 | 0,3618 | 42,36 | -0,1499 |
| 88,58 | 0,3567 | 42,3 | -0,2357 | 88,43 | 0,3468 | 42,28 | -0,2112 | 88,37 | 0,3618 | 42,4 | -0,15 |
| 88,55 | 0,3567 | 42,33 | -0,2358 | 88,39 | 0,3468 | 42,31 | -0,2113 | 88,34 | 0,3617 | 42,43 | -0,1501 |
| 88,52 | 0,3566 | 42,37 | -0,2358 | 88,36 | 0,3468 | 42,35 | -0,2113 | 88,3 | 0,3617 | 42,46 | -0,1502 |
| 88,48 | 0,3566 | 42,4 | -0,2358 | 88,33 | 0,3467 | 42,38 | -0,2114 | 88,27 | 0,3617 | 42,5 | -0,1502 |
| 88,45 | 0,3566 | 42,43 | -0,2359 | 88,29 | 0,3467 | 42,41 | -0,2114 | 88,24 | 0,3616 | 42,53 | -0,1502 |
| 88,42 | 0,3566 | 42,47 | -0,2359 | 88,26 | 0,3467 | 42,45 | -0,2115 | 88,2 | 0,3616 | 42,56 | -0,1503 |
| 88,38 | 0,3565 | 42,5 | -0,2359 | 88,23 | 0,3466 | 42,48 | -0,2115 | 88,17 | 0,3616 | 42,6 | -0,1503 |
| 88,35 | 0,3565 | 42,53 | -0,236 | 88,19 | 0,3466 | 42,51 | -0,2115 | 88,14 | 0,3615 | 42,63 | -0,1504 |
| 88,32 | 0,3565 | 42,57 | -0,236 | 88,16 | 0,3466 | 42,55 | -0,2116 | 88,1 | 0,3615 | 42,67 | -0,1504 |
| 88,28 | 0,3564 | 42,6 | -0,236 | 88,13 | 0,3465 | 42,58 | -0,2116 | 88,07 | 0,3614 | 42,7 | -0,1505 |
| 88,25 | 0,3564 | 42,63 | -0,2361 | 88,09 | 0,3465 | 42,61 | -0,2117 | 88,03 | 0,3614 | 42,73 | -0,1505 |
| 88,22 | 0,3564 | 42,67 | -0,2361 | 88,06 | 0,3465 | 42,65 | -0,2117 | 88 | 0,3614 | 42,77 | -0,1506 |
| 88,18 | 0,3563 | 42,7 | -0,2361 | 88,03 | 0,3464 | 42,68 | -0,2118 | 87,97 | 0,3613 | 42,8 | -0,1506 |
| 88,15 | 0,3563 | 42,73 | -0,2361 | 87,99 | 0,3464 | 42,71 | -0,2118 | 87,94 | 0,3613 | 42,83 | -0,1507 |
| 88,12 | 0,3563 | 42,77 | -0,2362 | 87,96 | 0,3464 | 42,75 | -0,2118 | 87,9 | 0,3613 | 42,87 | -0,1507 |
| 88,08 | 0,3563 | 42,8 | -0,2362 | 87,93 | 0,3463 | 42,78 | -0,2119 | 87,87 | 0,3612 | 42,9 | -0,1507 |
| 88,05 | 0,3562 | 42,83 | -0,2362 | 87,89 | 0,3463 | 42,81 | -0,2119 | 87,84 | 0,3612 | 42,93 | -0,1508 |
| 88,02 | 0,3562 | 42,87 | -0,2363 | 87,86 | 0,3463 | 42,85 | -0,212 | 87,8 | 0,3611 | 42,96 | -0,1508 |
| 87,98 | 0,3562 | 42,9 | -0,2363 | 87,83 | 0,3462 | 42,88 | -0,212 | 87,77 | 0,3611 | 43 | -0,1509 |
| 87,95 | 0,3561 | 42,93 | -0,2363 | 87,79 | 0,3462 | 42,91 | -0,2121 | 87,73 | 0,3611 | 43,03 | -0,1509 |
| 87,92 | 0,3561 | 42,97 | -0,2364 | 87,76 | 0,3462 | 42,95 | -0,2121 | 87,7 | 0,361 | 43,07 | -0,151 |
| 87,88 | 0,3561 | 43 | -0,2364 | 87,73 | 0,3461 | 42,98 | -0,2122 | 87,67 | 0,361 | 43,1 | -0,151 |
| 87,85 | 0,3561 | 43,03 | -0,2364 | 87,69 | 0,3461 | 43,01 | -0,2122 | 87,64 | 0,3609 | 43,13 | -0,1511 |
| 87,82 | 0,356 | 43,07 | -0,2365 | 87,66 | 0,3461 | 43,05 | -0,2122 | 87,6 | 0,3609 | 43,16 | -0,1511 |
| 87,78 | 0,356 | 43,1 | -0,2365 | 87,63 | 0,346 | 43,08 | -0,2123 | 87,57 | 0,3609 | 43,2 | -0,1511 |
| 87,75 | 0,356 | 43,13 | -0,2365 | 87,59 | 0,346 | 43,11 | -0,2123 | 87,54 | 0,3608 | 43,23 | -0,1512 |
| 87,72 | 0,3559 | 43,17 | -0,2366 | 87,56 | 0,346 | 43,15 | -0,2124 | 87,5 | 0,3608 | 43,26 | -0,1512 |
| 87,68 | 0,3559 | 43,2 | -0,2366 | 87,53 | 0,3459 | 43,18 | -0,2124 | 87,47 | 0,3607 | 43,3 | -0,1513 |
| 87,65 | 0,3559 | 43,23 | -0,2366 | 87,49 | 0,3459 | 43,21 | -0,2124 | 87,44 | 0,3607 | 43,33 | -0,1513 |
| 87,62 | 0,3558 | 43,27 | -0,2366 | 87,46 | 0,3459 | 43,25 | -0,2125 | 87,4 | 0,3607 | 43,36 | -0,1514 |
| 87,58 | 0,3558 | 43,3 | -0,2367 | 87,43 | 0,3458 | 43,28 | -0,2125 | 87,37 | 0,3606 | 43,4 | -0,1514 |
| 87,55 | 0,3558 | 43,33 | -0,2367 | 87,39 | 0,3458 | 43,31 | -0,2126 | 87,34 | 0,3606 | 43,43 | -0,1515 |
| 87,52 | 0,3558 | 43,37 | -0,2367 | 87,36 | 0,3458 | 43,35 | -0,2126 | 87,3 | 0,3606 | 43,46 | -0,1515 |
| 87,48 | 0,3557 | 43,4 | -0,2368 | 87,33 | 0,3457 | 43,38 | -0,2126 | 87,27 | 0,3605 | 43,5 | -0,1515 |
| 87,45 | 0,3557 | 43,43 | -0,2368 | 87,29 | 0,3457 | 43,41 | -0,2127 | 87,24 | 0,3605 | 43,53 | -0,1516 |
| 87,42 | 0,3557 | 43,47 | -0,2368 | 87,26 | 0,3457 | 43,45 | -0,2127 | 87,2 | 0,3604 | 43,56 | -0,1516 |
| 87,38 | 0,3556 | 43,5 | -0,2369 | 87,23 | 0,3456 | 43,48 | -0,2128 | 87,17 | 0,3604 | 43,6 | -0,1517 |
| 87,35 | 0,3556 | 43,53 | -0,2369 | 87,19 | 0,3456 | 43,51 | -0,2128 | 87,14 | 0,3604 | 43,63 | -0,1517 |
| 87,32 | 0,3556 | 43,57 | -0,2369 | 87,16 | 0,3456 | 43,55 | -0,2129 | 87,1 | 0,3603 | 43,66 | -0,1518 |
| 87,28 | 0,3556 | 43,6 | -0,237 | 87,13 | 0,3455 | 43,58 | -0,2129 | 87,07 | 0,3603 | 43,7 | -0,1518 |
| 87,25 | 0,3555 | 43,63 | -0,237 | 87,09 | 0,3455 | 43,61 | -0,2129 | 87,04 | 0,3603 | 43,73 | -0,1519 |
| 87,22 | 0,3555 | 43,67 | -0,237 | 87,06 | 0,3455 | 43,65 | -0,213 | 87 | 0,3602 | 43,76 | -0,1519 |
| 87,18 | 0,3555 | 43,7 | -0,237 | 87,03 | 0,3454 | 43,68 | -0,213 | 86,97 | 0,3602 | 43,8 | -0,152 |
| 87,15 | 0,3554 | 43,73 | -0,2371 | 86,99 | 0,3454 | 43,72 | -0,2131 | 86,94 | 0,3601 | 43,83 | -0,152 |
| 87,12 | 0,3554 | 43,77 | -0,2371 | 86,96 | 0,3454 | 43,75 | -0,2131 | 86,9 | 0,3601 | 43,86 | -0,1521 |
| 87,08 | 0,3554 | 43,8 | -0,2371 | 86,93 | 0,3453 | 43,78 | -0,2131 | 86,87 | 0,3601 | 43,9 | -0,1521 |
| 87,05 | 0,3553 | 43,83 | -0,2372 | 86,89 | 0,3453 | 43,81 | -0,2132 | 86,84 | 0,36 | 43,93 | -0,1522 |
| 87,02 | 0,3553 | 43,87 | -0,2372 | 86,86 | 0,3453 | 43,85 | -0,2132 | 86,8 | 0,36 | 43,97 | -0,1522 |
| 86,98 | 0,3553 | 43,9 | -0,2372 | 86,83 | 0,3452 | 43,88 | -0,2133 | 86,77 | 0,3599 | 44 | -0,1523 |
| 86,95 | 0,3553 | 43,94 | -0,2372 | 86,79 | 0,3452 | 43,91 | -0,2133 | 86,74 | 0,3599 | 44,03 | -0,1523 |
| 86,92 | 0,3552 | 43,97 | -0,2373 | 86,76 | 0,3452 | 43,95 | -0,2133 | 86,7 | 0,3599 | 44,06 | -0,1523 |
| 86,88 | 0,3552 | 44 | -0,2373 | 86,73 | 0,3451 | 43,98 | -0,2134 | 86,67 | 0,3598 | 44,1 | -0,1524 |
| 86,85 | 0,3552 | 44,04 | -0,2373 | 86,69 | 0,3451 | 44,02 | -0,2134 | 86,64 | 0,3598 | 44,13 | -0,1524 |
| 86,82 | 0,3551 | 44,07 | -0,2374 | 86,66 | 0,3451 | 44,05 | -0,2135 | 86,6 | 0,3597 | 44,16 | -0,1525 |
| 86,78 | 0,3551 | 44,1 | -0,2374 | 86,63 | 0,345 | 44,08 | -0,2135 | 86,57 | 0,3597 | 44,2 | -0,1525 |
| 86,75 | 0,3551 | 44,14 | -0,2374 | 86,59 | 0,345 | 44,11 | -0,2135 | 86,53 | 0,3597 | 44,23 | -0,1526 |
| 86,72 | 0,355 | 44,17 | -0,2375 | 86,56 | 0,345 | 44,15 | -0,2136 | 86,5 | 0,3596 | 44,26 | -0,1526 |
| 86,68 | 0,355 | 44,2 | -0,2375 | 86,53 | 0,3449 | 44,18 | -0,2136 | 86,47 | 0,3596 | 44,3 | -0,1527 |
| 86,65 | 0,355 | 44,23 | -0,2375 | 86,49 | 0,3449 | 44,21 | -0,2136 | 86,43 | 0,3595 | 44,33 | -0,1527 |
| 86,62 | 0,3549 | 44,27 | -0,2375 | 86,46 | 0,3449 | 44,25 | -0,2137 | 86,4 | 0,3595 | 44,36 | -0,1528 |
| 86,58 | 0,3549 | 44,3 | -0,2376 | 86,43 | 0,3448 | 44,28 | -0,2137 | 86,37 | 0,3595 | 44,4 | -0,1528 |
| 86,55 | 0,3549 | 44,33 | -0,2376 | 86,39 | 0,3448 | 44,32 | -0,2138 | 86,33 | 0,3594 | 44,43 | -0,1529 |
| 86,52 | 0,3549 | 44,37 | -0,2376 | 86,36 | 0,3448 | 44,35 | -0,2138 | 86,3 | 0,3594 | 44,46 | -0,1529 |
| 86,48 | 0,3548 | 44,4 | -0,2377 | 86,33 | 0,3447 | 44,38 | -0,2138 | 86,27 | 0,3593 | 44,5 | -0,153 |
| 86,45 | 0,3548 | 44,43 | -0,2377 | 86,29 | 0,3447 | 44,41 | -0,2139 | 86,23 | 0,3593 | 44,53 | -0,153 |
| 86,42 | 0,3548 | 44,47 | -0,2377 | 86,26 | 0,3447 | 44,45 | -0,2139 | 86,2 | 0,3593 | 44,56 | -0,1531 |
| 86,38 | 0,3547 | 44,5 | -0,2377 | 86,23 | 0,3446 | 44,48 | -0,2139 | 86,17 | 0,3592 | 44,6 | -0,1531 |
| 86,35 | 0,3547 | 44,53 | -0,2378 | 86,19 | 0,3446 | 44,52 | -0,214 | 86,14 | 0,3592 | 44,63 | -0,1532 |
| 86,32 | 0,3547 | 44,57 | -0,2378 | 86,16 | 0,3446 | 44,55 | -0,214 | 86,1 | 0,3592 | 44,67 | -0,1532 |
| 86,28 | 0,3546 | 44,6 | -0,2378 | 86,13 | 0,3445 | 44,58 | -0,2141 | 86,07 | 0,3591 | 44,7 | -0,1533 |
| 86,25 | 0,3546 | 44,64 | -0,2379 | 86,09 | 0,3445 | 44,62 | -0,2141 | 86,03 | 0,3591 | 44,73 | -0,1533 |
| 86,22 | 0,3546 | 44,67 | -0,2379 | 86,06 | 0,3445 | 44,65 | -0,2141 | 86 | 0,359 | 44,77 | -0,1534 |
| 86,18 | 0,3546 | 44,7 | -0,2379 | 86,03 | 0,3444 | 44,68 | -0,2142 | 85,97 | 0,359 | 44,8 | -0,1534 |
| 86,15 | 0,3545 | 44,73 | -0,2379 | 85,99 | 0,3444 | 44,72 | -0,2142 | 85,94 | 0,359 | 44,83 | -0,1535 |
| 86,12 | 0,3545 | 44,77 | -0,238 | 85,96 | 0,3444 | 44,75 | -0,2142 | 85,9 | 0,3589 | 44,87 | -0,1535 |
| 86,08 | 0,3545 | 44,8 | -0,238 | 85,93 | 0,3443 | 44,78 | -0,2143 | 85,87 | 0,3589 | 44,9 | -0,1536 |
| 86,05 | 0,3544 | 44,84 | -0,238 | 85,89 | 0,3443 | 44,82 | -0,2143 | 85,83 | 0,3588 | 44,93 | -0,1536 |
| 86,02 | 0,3544 | 44,87 | -0,2381 | 85,86 | 0,3443 | 44,85 | -0,2143 | 85,8 | 0,3588 | 44,96 | -0,1537 |
| 85,98 | 0,3544 | 44,9 | -0,2381 | 85,83 | 0,3442 | 44,88 | -0,2144 | 85,77 | 0,3588 | 45 | -0,1537 |
| 85,95 | 0,3543 | 44,94 | -0,2381 | 85,79 | 0,3442 | 44,92 | -0,2144 | 85,74 | 0,3587 | 45,03 | -0,1538 |
| 85,92 | 0,3543 | 44,97 | -0,2381 | 85,76 | 0,3442 | 44,95 | -0,2145 | 85,7 | 0,3587 | 45,07 | -0,1538 |
| 85,88 | 0,3543 | 45 | -0,2382 | 85,73 | 0,3441 | 44,98 | -0,2145 | 85,67 | 0,3586 | 45,1 | -0,1538 |
| 85,85 | 0,3542 | 45,04 | -0,2382 | 85,69 | 0,3441 | 45,02 | -0,2145 | 85,63 | 0,3586 | 45,13 | -0,1539 |
| 85,82 | 0,3542 | 45,07 | -0,2382 | 85,66 | 0,3441 | 45,05 | -0,2146 | 85,6 | 0,3586 | 45,17 | -0,1539 |
| 85,78 | 0,3542 | 45,1 | -0,2383 | 85,63 | 0,344 | 45,08 | -0,2146 | 85,57 | 0,3585 | 45,2 | -0,154 |
| 85,75 | 0,3542 | 45,14 | -0,2383 | 85,59 | 0,344 | 45,12 | -0,2146 | 85,53 | 0,3585 | 45,23 | -0,154 |
| 85,72 | 0,3541 | 45,17 | -0,2383 | 85,56 | 0,344 | 45,15 | -0,2147 | 85,5 | 0,3584 | 45,26 | -0,1541 |
| 85,68 | 0,3541 | 45,2 | -0,2383 | 85,53 | 0,3439 | 45,18 | -0,2147 | 85,47 | 0,3584 | 45,3 | -0,1541 |
| 85,65 | 0,3541 | 45,23 | -0,2384 | 85,49 | 0,3439 | 45,22 | -0,2147 | 85,43 | 0,3583 | 45,33 | -0,1542 |
| 85,62 | 0,354 | 45,27 | -0,2384 | 85,46 | 0,3439 | 45,25 | -0,2148 | 85,4 | 0,3583 | 45,37 | -0,1542 |
| 85,58 | 0,354 | 45,3 | -0,2384 | 85,43 | 0,3438 | 45,28 | -0,2148 | 85,37 | 0,3583 | 45,4 | -0,1543 |
| 85,55 | 0,354 | 45,34 | -0,2384 | 85,39 | 0,3438 | 45,32 | -0,2148 | 85,33 | 0,3582 | 45,43 | -0,1543 |
| 85,52 | 0,3539 | 45,37 | -0,2385 | 85,36 | 0,3438 | 45,35 | -0,2149 | 85,3 | 0,3582 | 45,46 | -0,1544 |
| 85,48 | 0,3539 | 45,4 | -0,2385 | 85,33 | 0,3437 | 45,38 | -0,2149 | 85,27 | 0,3582 | 45,5 | -0,1544 |
| 85,45 | 0,3539 | 45,44 | -0,2385 | 85,29 | 0,3437 | 45,42 | -0,2149 | 85,23 | 0,3581 | 45,53 | -0,1545 |
| 85,42 | 0,3538 | 45,47 | -0,2386 | 85,26 | 0,3437 | 45,45 | -0,215 | 85,2 | 0,3581 | 45,57 | -0,1545 |
| 85,38 | 0,3538 | 45,5 | -0,2386 | 85,23 | 0,3436 | 45,48 | -0,215 | 85,17 | 0,358 | 45,6 | -0,1546 |
| 85,35 | 0,3538 | 45,53 | -0,2386 | 85,19 | 0,3436 | 45,52 | -0,2151 | 85,13 | 0,358 | 45,63 | -0,1546 |
| 85,32 | 0,3537 | 45,57 | -0,2386 | 85,16 | 0,3436 | 45,55 | -0,2151 | 85,1 | 0,3579 | 45,67 | -0,1547 |
| 85,28 | 0,3537 | 45,6 | -0,2387 | 85,13 | 0,3435 | 45,58 | -0,2151 | 85,07 | 0,3579 | 45,7 | -0,1547 |
| 85,25 | 0,3537 | 45,63 | -0,2387 | 85,09 | 0,3435 | 45,62 | -0,2152 | 85,03 | 0,3579 | 45,73 | -0,1548 |
| 85,22 | 0,3536 | 45,67 | -0,2387 | 85,06 | 0,3435 | 45,65 | -0,2152 | 85 | 0,3578 | 45,77 | -0,1548 |
| 85,18 | 0,3536 | 45,7 | -0,2387 | 85,03 | 0,3434 | 45,68 | -0,2152 | 84,97 | 0,3578 | 45,8 | -0,1549 |
| 85,15 | 0,3536 | 45,73 | -0,2388 | 84,99 | 0,3434 | 45,72 | -0,2153 | 84,94 | 0,3577 | 45,83 | -0,1549 |
| 85,12 | 0,3536 | 45,77 | -0,2388 | 84,96 | 0,3434 | 45,75 | -0,2153 | 84,9 | 0,3577 | 45,87 | -0,155 |
| 85,08 | 0,3535 | 45,8 | -0,2388 | 84,93 | 0,3433 | 45,78 | -0,2153 | 84,87 | 0,3577 | 45,9 | -0,155 |
| 85,05 | 0,3535 | 45,84 | -0,2389 | 84,89 | 0,3433 | 45,82 | -0,2154 | 84,83 | 0,3576 | 45,93 | -0,1551 |
| 85,02 | 0,3535 | 45,87 | -0,2389 | 84,86 | 0,3433 | 45,85 | -0,2154 | 84,8 | 0,3576 | 45,97 | -0,1551 |
| 84,98 | 0,3534 | 45,9 | -0,2389 | 84,83 | 0,3432 | 45,88 | -0,2154 | 84,77 | 0,3575 | 46 | -0,1552 |
| 84,95 | 0,3534 | 45,94 | -0,2389 | 84,79 | 0,3432 | 45,92 | -0,2155 | 84,73 | 0,3575 | 46,03 | -0,1552 |
| 84,92 | 0,3534 | 45,97 | -0,239 | 84,76 | 0,3432 | 45,95 | -0,2155 | 84,7 | 0,3575 | 46,07 | -0,1553 |
| 84,88 | 0,3533 | 46 | -0,239 | 84,73 | 0,3431 | 45,98 | -0,2155 | 84,67 | 0,3574 | 46,1 | -0,1553 |
| 84,85 | 0,3533 | 46,04 | -0,239 | 84,69 | 0,3431 | 46,02 | -0,2156 | 84,63 | 0,3574 | 46,13 | -0,1554 |
| 84,82 | 0,3533 | 46,07 | -0,239 | 84,66 | 0,3431 | 46,05 | -0,2156 | 84,6 | 0,3573 | 46,17 | -0,1554 |
| 84,78 | 0,3532 | 46,1 | -0,2391 | 84,63 | 0,343 | 46,08 | -0,2156 | 84,57 | 0,3573 | 46,2 | -0,1555 |
| 84,75 | 0,3532 | 46,14 | -0,2391 | 84,59 | 0,343 | 46,12 | -0,2157 | 84,53 | 0,3573 | 46,23 | -0,1556 |
| 84,72 | 0,3532 | 46,17 | -0,2391 | 84,56 | 0,343 | 46,15 | -0,2157 | 84,5 | 0,3572 | 46,27 | -0,1556 |
| 84,68 | 0,3531 | 46,2 | -0,2392 | 84,53 | 0,3429 | 46,18 | -0,2157 | 84,47 | 0,3572 | 46,3 | -0,1557 |
| 84,65 | 0,3531 | 46,24 | -0,2392 | 84,49 | 0,3429 | 46,22 | -0,2158 | 84,43 | 0,3571 | 46,33 | -0,1557 |
| 84,62 | 0,3531 | 46,27 | -0,2392 | 84,46 | 0,3429 | 46,25 | -0,2158 | 84,4 | 0,3571 | 46,37 | -0,1558 |
| 84,58 | 0,353 | 46,3 | -0,2392 | 84,43 | 0,3428 | 46,28 | -0,2158 | 84,37 | 0,357 | 46,4 | -0,1558 |
| 84,55 | 0,353 | 46,34 | -0,2393 | 84,39 | 0,3428 | 46,32 | -0,2159 | 84,33 | 0,357 | 46,43 | -0,1559 |
| 84,52 | 0,353 | 46,37 | -0,2393 | 84,36 | 0,3428 | 46,35 | -0,2159 | 84,3 | 0,357 | 46,47 | -0,1559 |
| 84,48 | 0,3529 | 46,4 | -0,2393 | 84,33 | 0,3427 | 46,38 | -0,2159 | 84,27 | 0,3569 | 46,5 | -0,156 |
| 84,45 | 0,3529 | 46,44 | -0,2393 | 84,29 | 0,3427 | 46,42 | -0,216 | 84,23 | 0,3569 | 46,53 | -0,156 |
| 84,42 | 0,3529 | 46,47 | -0,2394 | 84,26 | 0,3427 | 46,45 | -0,216 | 84,2 | 0,3568 | 46,57 | -0,1561 |
| 84,38 | 0,3528 | 46,5 | -0,2394 | 84,23 | 0,3426 | 46,48 | -0,216 | 84,17 | 0,3568 | 46,6 | -0,1561 |
| 84,35 | 0,3528 | 46,54 | -0,2394 | 84,19 | 0,3426 | 46,52 | -0,216 | 84,13 | 0,3568 | 46,63 | -0,1562 |
| 84,32 | 0,3528 | 46,57 | -0,2394 | 84,16 | 0,3426 | 46,55 | -0,2161 | 84,1 | 0,3567 | 46,67 | -0,1562 |
| 84,28 | 0,3527 | 46,6 | -0,2395 | 84,13 | 0,3425 | 46,58 | -0,2161 | 84,07 | 0,3567 | 46,7 | -0,1563 |
| 84,25 | 0,3527 | 46,64 | -0,2395 | 84,09 | 0,3425 | 46,62 | -0,2161 | 84,04 | 0,3566 | 46,73 | -0,1563 |
| 84,21 | 0,3527 | 46,67 | -0,2395 | 84,06 | 0,3425 | 46,65 | -0,2162 | 84 | 0,3566 | 46,77 | -0,1564 |
| 84,18 | 0,3527 | 46,7 | -0,2395 | 84,03 | 0,3424 | 46,68 | -0,2162 | 83,97 | 0,3566 | 46,8 | -0,1564 |
| 84,15 | 0,3526 | 46,74 | -0,2396 | 83,99 | 0,3424 | 46,72 | -0,2162 | 83,93 | 0,3565 | 46,83 | -0,1565 |
| 84,12 | 0,3526 | 46,77 | -0,2396 | 83,96 | 0,3424 | 46,75 | -0,2163 | 83,9 | 0,3565 | 46,86 | -0,1565 |
| 84,08 | 0,3526 | 46,8 | -0,2396 | 83,93 | 0,3423 | 46,78 | -0,2163 | 83,87 | 0,3564 | 46,9 | -0,1566 |
| 84,05 | 0,3525 | 46,84 | -0,2396 | 83,89 | 0,3423 | 46,82 | -0,2163 | 83,84 | 0,3564 | 46,93 | -0,1566 |
| 84,02 | 0,3525 | 46,87 | -0,2397 | 83,86 | 0,3422 | 46,85 | -0,2164 | 83,8 | 0,3563 | 46,97 | -0,1567 |
| 83,98 | 0,3525 | 46,9 | -0,2397 | 83,83 | 0,3422 | 46,88 | -0,2164 | 83,77 | 0,3563 | 47 | -0,1568 |
| 83,95 | 0,3524 | 46,94 | -0,2397 | 83,79 | 0,3422 | 46,92 | -0,2164 | 83,73 | 0,3562 | 47,03 | -0,1568 |
| 83,92 | 0,3524 | 46,97 | -0,2398 | 83,76 | 0,3421 | 46,95 | -0,2165 | 83,7 | 0,3562 | 47,07 | -0,1569 |
| 83,88 | 0,3524 | 47 | -0,2398 | 83,73 | 0,3421 | 46,98 | -0,2165 | 83,67 | 0,3562 | 47,1 | -0,1569 |
| 83,85 | 0,3523 | 47,04 | -0,2398 | 83,69 | 0,3421 | 47,02 | -0,2165 | 83,64 | 0,3561 | 47,13 | -0,157 |
| 83,82 | 0,3523 | 47,07 | -0,2398 | 83,66 | 0,342 | 47,05 | -0,2165 | 83,6 | 0,3561 | 47,17 | -0,157 |
| 83,78 | 0,3523 | 47,1 | -0,2399 | 83,63 | 0,342 | 47,08 | -0,2166 | 83,57 | 0,356 | 47,2 | -0,1571 |
| 83,75 | 0,3522 | 47,14 | -0,2399 | 83,59 | 0,342 | 47,12 | -0,2166 | 83,54 | 0,356 | 47,23 | -0,1571 |
| 83,71 | 0,3522 | 47,17 | -0,2399 | 83,56 | 0,3419 | 47,15 | -0,2166 | 83,5 | 0,356 | 47,27 | -0,1572 |
| 83,68 | 0,3522 | 47,2 | -0,2399 | 83,53 | 0,3419 | 47,19 | -0,2167 | 83,47 | 0,3559 | 47,3 | -0,1572 |
| 83,65 | 0,3521 | 47,24 | -0,24 | 83,49 | 0,3419 | 47,22 | -0,2167 | 83,43 | 0,3559 | 47,33 | -0,1573 |
| 83,61 | 0,3521 | 47,27 | -0,24 | 83,46 | 0,3418 | 47,25 | -0,2167 | 83,4 | 0,3558 | 47,37 | -0,1574 |
| 83,58 | 0,3521 | 47,3 | -0,24 | 83,43 | 0,3418 | 47,28 | -0,2168 | 83,37 | 0,3558 | 47,4 | -0,1574 |
| 83,55 | 0,352 | 47,34 | -0,2401 | 83,39 | 0,3418 | 47,32 | -0,2168 | 83,33 | 0,3557 | 47,43 | -0,1575 |
| 83,51 | 0,352 | 47,37 | -0,2401 | 83,36 | 0,3417 | 47,35 | -0,2168 | 83,3 | 0,3557 | 47,47 | -0,1575 |
| 83,48 | 0,352 | 47,4 | -0,2401 | 83,33 | 0,3417 | 47,38 | -0,2169 | 83,27 | 0,3557 | 47,5 | -0,1576 |
| 83,45 | 0,3519 | 47,44 | -0,2401 | 83,29 | 0,3417 | 47,42 | -0,2169 | 83,24 | 0,3556 | 47,53 | -0,1577 |
| 83,41 | 0,3519 | 47,47 | -0,2402 | 83,26 | 0,3416 | 47,45 | -0,2169 | 83,2 | 0,3556 | 47,57 | -0,1577 |
| 83,38 | 0,3519 | 47,5 | -0,2402 | 83,23 | 0,3416 | 47,48 | -0,2169 | 83,17 | 0,3555 | 47,6 | -0,1578 |
| 83,35 | 0,3518 | 47,54 | -0,2402 | 83,19 | 0,3416 | 47,52 | -0,217 | 83,13 | 0,3555 | 47,63 | -0,1578 |
| 83,32 | 0,3518 | 47,57 | -0,2402 | 83,16 | 0,3415 | 47,55 | -0,217 | 83,1 | 0,3554 | 47,67 | -0,1579 |
| 83,28 | 0,3518 | 47,6 | -0,2403 | 83,13 | 0,3415 | 47,58 | -0,217 | 83,07 | 0,3554 | 47,7 | -0,1579 |
| 83,25 | 0,3517 | 47,64 | -0,2403 | 83,09 | 0,3414 | 47,62 | -0,2171 | 83,04 | 0,3553 | 47,73 | -0,158 |
| 83,21 | 0,3517 | 47,67 | -0,2403 | 83,06 | 0,3414 | 47,65 | -0,2171 | 83 | 0,3553 | 47,76 | -0,1581 |
| 83,18 | 0,3517 | 47,7 | -0,2403 | 83,03 | 0,3414 | 47,68 | -0,2171 | 82,97 | 0,3553 | 47,8 | -0,1581 |
| 83,15 | 0,3516 | 47,74 | -0,2404 | 82,99 | 0,3413 | 47,72 | -0,2172 | 82,93 | 0,3552 | 47,83 | -0,1582 |
| 83,12 | 0,3516 | 47,77 | -0,2404 | 82,96 | 0,3413 | 47,75 | -0,2172 | 82,9 | 0,3552 | 47,87 | -0,1582 |
| 83,08 | 0,3516 | 47,8 | -0,2404 | 82,93 | 0,3413 | 47,79 | -0,2172 | 82,87 | 0,3551 | 47,9 | -0,1583 |
| 83,05 | 0,3515 | 47,84 | -0,2404 | 82,89 | 0,3412 | 47,82 | -0,2172 | 82,83 | 0,3551 | 47,93 | -0,1583 |
| 83,02 | 0,3515 | 47,87 | -0,2405 | 82,86 | 0,3412 | 47,85 | -0,2173 | 82,8 | 0,355 | 47,97 | -0,1584 |
| 82,98 | 0,3515 | 47,9 | -0,2405 | 82,83 | 0,3412 | 47,88 | -0,2173 | 82,77 | 0,355 | 48 | -0,1585 |
| 82,95 | 0,3514 | 47,94 | -0,2405 | 82,79 | 0,3411 | 47,92 | -0,2173 | 82,73 | 0,3549 | 48,03 | -0,1585 |
| 82,92 | 0,3514 | 47,97 | -0,2406 | 82,76 | 0,3411 | 47,95 | -0,2174 | 82,7 | 0,3549 | 48,07 | -0,1586 |
| 82,88 | 0,3513 | 48 | -0,2406 | 82,73 | 0,3411 | 47,98 | -0,2174 | 82,67 | 0,3548 | 48,1 | -0,1586 |
| 82,85 | 0,3513 | 48,04 | -0,2406 | 82,69 | 0,341 | 48,02 | -0,2174 | 82,63 | 0,3548 | 48,13 | -0,1587 |
| 82,82 | 0,3513 | 48,07 | -0,2406 | 82,66 | 0,341 | 48,05 | -0,2174 | 82,6 | 0,3548 | 48,17 | -0,1587 |
| 82,78 | 0,3512 | 48,1 | -0,2407 | 82,63 | 0,3409 | 48,08 | -0,2175 | 82,57 | 0,3547 | 48,2 | -0,1588 |
| 82,75 | 0,3512 | 48,14 | -0,2407 | 82,59 | 0,3409 | 48,12 | -0,2175 | 82,53 | 0,3547 | 48,23 | -0,1589 |
| 82,71 | 0,3512 | 48,17 | -0,2407 | 82,56 | 0,3409 | 48,15 | -0,2175 | 82,5 | 0,3546 | 48,27 | -0,1589 |
| 82,68 | 0,3511 | 48,2 | -0,2407 | 82,53 | 0,3408 | 48,18 | -0,2176 | 82,47 | 0,3546 | 48,3 | -0,159 |
| 82,65 | 0,3511 | 48,24 | -0,2408 | 82,49 | 0,3408 | 48,22 | -0,2176 | 82,43 | 0,3545 | 48,33 | -0,159 |
| 82,62 | 0,3511 | 48,27 | -0,2408 | 82,46 | 0,3408 | 48,25 | -0,2176 | 82,4 | 0,3545 | 48,36 | -0,1591 |
| 82,58 | 0,351 | 48,3 | -0,2408 | 82,43 | 0,3407 | 48,29 | -0,2177 | 82,37 | 0,3545 | 48,4 | -0,1592 |
| 82,55 | 0,351 | 48,34 | -0,2408 | 82,39 | 0,3407 | 48,32 | -0,2177 | 82,33 | 0,3544 | 48,43 | -0,1592 |
| 82,52 | 0,351 | 48,37 | -0,2409 | 82,36 | 0,3407 | 48,35 | -0,2177 | 82,3 | 0,3544 | 48,46 | -0,1593 |
| 82,48 | 0,3509 | 48,4 | -0,2409 | 82,33 | 0,3406 | 48,38 | -0,2177 | 82,27 | 0,3543 | 48,5 | -0,1594 |
| 82,45 | 0,3509 | 48,44 | -0,2409 | 82,29 | 0,3406 | 48,42 | -0,2178 | 82,23 | 0,3543 | 48,53 | -0,1594 |
| 82,42 | 0,3509 | 48,47 | -0,2409 | 82,26 | 0,3405 | 48,45 | -0,2178 | 82,2 | 0,3542 | 48,57 | -0,1595 |
| 82,38 | 0,3508 | 48,5 | -0,241 | 82,23 | 0,3405 | 48,49 | -0,2178 | 82,17 | 0,3542 | 48,6 | -0,1595 |
| 82,35 | 0,3508 | 48,54 | -0,241 | 82,19 | 0,3405 | 48,52 | -0,2179 | 82,14 | 0,3541 | 48,63 | -0,1596 |
| 82,31 | 0,3507 | 48,57 | -0,241 | 82,16 | 0,3404 | 48,55 | -0,2179 | 82,1 | 0,3541 | 48,66 | -0,1597 |
| 82,28 | 0,3507 | 48,6 | -0,241 | 82,13 | 0,3404 | 48,59 | -0,2179 | 82,07 | 0,354 | 48,7 | -0,1597 |
| 82,25 | 0,3507 | 48,64 | -0,2411 | 82,09 | 0,3404 | 48,62 | -0,2179 | 82,03 | 0,354 | 48,73 | -0,1598 |
| 82,21 | 0,3506 | 48,67 | -0,2411 | 82,06 | 0,3403 | 48,65 | -0,218 | 82 | 0,3539 | 48,76 | -0,1599 |
| 82,18 | 0,3506 | 48,7 | -0,2411 | 82,03 | 0,3403 | 48,68 | -0,218 | 81,97 | 0,3539 | 48,8 | -0,1599 |
| 82,15 | 0,3506 | 48,74 | -0,2412 | 81,99 | 0,3403 | 48,72 | -0,218 | 81,93 | 0,3539 | 48,83 | -0,16 |
| 82,12 | 0,3505 | 48,77 | -0,2412 | 81,96 | 0,3402 | 48,75 | -0,2181 | 81,9 | 0,3538 | 48,86 | -0,16 |
| 82,08 | 0,3505 | 48,8 | -0,2412 | 81,93 | 0,3402 | 48,78 | -0,2181 | 81,87 | 0,3538 | 48,9 | -0,1601 |
| 82,05 | 0,3505 | 48,84 | -0,2412 | 81,89 | 0,3402 | 48,82 | -0,2181 | 81,83 | 0,3537 | 48,93 | -0,1602 |
| 82,01 | 0,3504 | 48,87 | -0,2413 | 81,86 | 0,3401 | 48,85 | -0,2181 | 81,8 | 0,3537 | 48,96 | -0,1602 |
| 81,98 | 0,3504 | 48,9 | -0,2413 | 81,83 | 0,3401 | 48,88 | -0,2182 | 81,77 | 0,3536 | 49 | -0,1603 |
| 81,95 | 0,3504 | 48,94 | -0,2413 | 81,79 | 0,34 | 48,92 | -0,2182 | 81,73 | 0,3536 | 49,03 | -0,1604 |
| 81,91 | 0,3503 | 48,97 | -0,2413 | 81,76 | 0,34 | 48,95 | -0,2182 | 81,7 | 0,3535 | 49,06 | -0,1604 |
| 81,88 | 0,3503 | 49 | -0,2414 | 81,73 | 0,34 | 48,99 | -0,2183 | 81,67 | 0,3535 | 49,1 | -0,1605 |
| 81,85 | 0,3502 | 49,04 | -0,2414 | 81,69 | 0,3399 | 49,02 | -0,2183 | 81,63 | 0,3534 | 49,13 | -0,1606 |
| 81,81 | 0,3502 | 49,07 | -0,2414 | 81,66 | 0,3399 | 49,05 | -0,2183 | 81,6 | 0,3534 | 49,16 | -0,1606 |
| 81,78 | 0,3502 | 49,1 | -0,2415 | 81,63 | 0,3399 | 49,08 | -0,2184 | 81,57 | 0,3533 | 49,2 | -0,1607 |
| 81,75 | 0,3501 | 49,14 | -0,2415 | 81,59 | 0,3398 | 49,12 | -0,2184 | 81,53 | 0,3533 | 49,23 | -0,1608 |
| 81,71 | 0,3501 | 49,17 | -0,2415 | 81,56 | 0,3398 | 49,15 | -0,2184 | 81,5 | 0,3533 | 49,26 | -0,1609 |
| 81,68 | 0,35 | 49,2 | -0,2415 | 81,53 | 0,3397 | 49,18 | -0,2184 | 81,47 | 0,3532 | 49,3 | -0,1609 |
| 81,65 | 0,35 | 49,24 | -0,2416 | 81,49 | 0,3397 | 49,22 | -0,2185 | 81,43 | 0,3532 | 49,33 | -0,161 |
| 81,61 | 0,3499 | 49,27 | -0,2416 | 81,46 | 0,3397 | 49,25 | -0,2185 | 81,4 | 0,3531 | 49,36 | -0,1611 |
| 81,58 | 0,3499 | 49,3 | -0,2416 | 81,43 | 0,3396 | 49,29 | -0,2185 | 81,37 | 0,3531 | 49,4 | -0,1611 |
| 81,55 | 0,3499 | 49,34 | -0,2416 | 81,39 | 0,3396 | 49,32 | -0,2186 | 81,33 | 0,353 | 49,43 | -0,1612 |
| 81,51 | 0,3498 | 49,37 | -0,2417 | 81,36 | 0,3396 | 49,35 | -0,2186 | 81,3 | 0,353 | 49,47 | -0,1613 |
| 81,48 | 0,3498 | 49,4 | -0,2417 | 81,33 | 0,3395 | 49,38 | -0,2186 | 81,27 | 0,3529 | 49,5 | -0,1613 |
| 81,45 | 0,3498 | 49,44 | -0,2417 | 81,29 | 0,3395 | 49,42 | -0,2186 | 81,23 | 0,3528 | 49,53 | -0,1614 |
| 81,41 | 0,3497 | 49,47 | -0,2417 | 81,26 | 0,3395 | 49,45 | -0,2187 | 81,2 | 0,3528 | 49,56 | -0,1615 |
| 81,38 | 0,3497 | 49,5 | -0,2418 | 81,23 | 0,3394 | 49,49 | -0,2187 | 81,17 | 0,3527 | 49,6 | -0,1615 |
| 81,35 | 0,3497 | 49,54 | -0,2418 | 81,19 | 0,3394 | 49,52 | -0,2187 | 81,13 | 0,3527 | 49,63 | -0,1616 |
| 81,31 | 0,3496 | 49,57 | -0,2418 | 81,16 | 0,3394 | 49,55 | -0,2188 | 81,1 | 0,3527 | 49,66 | -0,1616 |
| 81,28 | 0,3496 | 49,6 | -0,2418 | 81,13 | 0,3393 | 49,59 | -0,2188 | 81,07 | 0,3526 | 49,7 | -0,1617 |
| 81,25 | 0,3495 | 49,64 | -0,2419 | 81,09 | 0,3393 | 49,62 | -0,2188 | 81,03 | 0,3526 | 49,73 | -0,1618 |
| 81,21 | 0,3495 | 49,67 | -0,2419 | 81,06 | 0,3392 | 49,65 | -0,2188 | 81 | 0,3525 | 49,76 | -0,1618 |
| 81,18 | 0,3495 | 49,7 | -0,2419 | 81,02 | 0,3392 | 49,69 | -0,2189 | 80,97 | 0,3525 | 49,8 | -0,1619 |
| 81,15 | 0,3494 | 49,74 | -0,242 | 80,99 | 0,3392 | 49,72 | -0,2189 | 80,93 | 0,3524 | 49,83 | -0,162 |
| 81,11 | 0,3494 | 49,77 | -0,242 | 80,96 | 0,3391 | 49,75 | -0,2189 | 80,9 | 0,3524 | 49,86 | -0,1621 |
| 81,08 | 0,3494 | 49,81 | -0,242 | 80,93 | 0,3391 | 49,79 | -0,219 | 80,87 | 0,3523 | 49,9 | -0,1623 |
| 81,05 | 0,3493 | 49,84 | -0,242 | 80,89 | 0,3391 | 49,82 | -0,219 | 80,83 | 0,3523 | 49,93 | -0,1624 |
| 81,01 | 0,3493 | 49,87 | -0,2421 | 80,86 | 0,339 | 49,85 | -0,219 | 80,8 | 0,3522 | 49,96 | -0,1624 |
| 80,98 | 0,3492 | 49,9 | -0,2421 | 80,83 | 0,339 | 49,88 | -0,219 | 80,77 | 0,3522 | 50 | -0,1625 |
| 80,95 | 0,3492 | 49,94 | -0,2421 | 80,79 | 0,3389 | 49,92 | -0,2191 | 80,73 | 0,3521 | 50,03 | -0,1625 |
| 80,91 | 0,3492 | 49,97 | -0,2421 | 80,76 | 0,3389 | 49,95 | -0,2191 | 80,7 | 0,3521 | 50,06 | -0,1626 |
| 80,88 | 0,3491 | 50 | -0,2422 | 80,73 | 0,3389 | 49,99 | -0,2191 | 80,67 | 0,352 | 50,1 | -0,1627 |
| 80,85 | 0,3491 | 50,04 | -0,2422 | 80,69 | 0,3388 | 50,02 | -0,2192 | 80,63 | 0,352 | 50,13 | -0,1628 |
| 80,81 | 0,3491 | 50,07 | -0,2422 | 80,66 | 0,3388 | 50,05 | -0,2192 | 80,6 | 0,3519 | 50,16 | -0,1629 |
| 80,78 | 0,349 | 50,1 | -0,2423 | 80,63 | 0,3388 | 50,09 | -0,2192 | 80,57 | 0,3519 | 50,2 | -0,1629 |
| 80,75 | 0,349 | 50,14 | -0,2423 | 80,59 | 0,3387 | 50,12 | -0,2193 | 80,53 | 0,3519 | 50,23 | -0,163 |
| 80,71 | 0,349 | 50,17 | -0,2423 | 80,56 | 0,3387 | 50,15 | -0,2193 | 80,5 | 0,3518 | 50,26 | -0,1631 |
| 80,68 | 0,3489 | 50,21 | -0,2423 | 80,53 | 0,3386 | 50,19 | -0,2193 | 80,47 | 0,3518 | 50,3 | -0,1632 |
| 80,65 | 0,3489 | 50,24 | -0,2424 | 80,49 | 0,3386 | 50,22 | -0,2193 | 80,43 | 0,3517 | 50,33 | -0,1633 |
| 80,61 | 0,3488 | 50,27 | -0,2424 | 80,46 | 0,3386 | 50,25 | -0,2194 | 80,4 | 0,3516 | 50,36 | -0,1633 |
| 80,58 | 0,3488 | 50,3 | -0,2424 | 80,43 | 0,3385 | 50,29 | -0,2194 | 80,37 | 0,3516 | 50,4 | -0,1634 |
| 80,55 | 0,3488 | 50,34 | -0,2424 | 80,39 | 0,3385 | 50,32 | -0,2194 | 80,33 | 0,3515 | 50,43 | -0,1635 |
| 80,51 | 0,3487 | 50,37 | -0,2425 | 80,36 | 0,3385 | 50,35 | -0,2194 | 80,3 | 0,3515 | 50,46 | -0,1635 |
| 80,48 | 0,3487 | 50,41 | -0,2425 | 80,33 | 0,3384 | 50,39 | -0,2195 | 80,27 | 0,3515 | 50,5 | -0,1636 |
| 80,45 | 0,3487 | 50,44 | -0,2425 | 80,29 | 0,3384 | 50,42 | -0,2195 | 80,23 | 0,3514 | 50,53 | -0,1638 |
| 80,41 | 0,3486 | 50,47 | -0,2426 | 80,26 | 0,3383 | 50,45 | -0,2195 | 80,2 | 0,3514 | 50,56 | -0,1639 |
| 80,38 | 0,3486 | 50,5 | -0,2426 | 80,23 | 0,3383 | 50,49 | -0,2196 | 80,17 | 0,3513 | 50,6 | -0,164 |
| 80,35 | 0,3485 | 50,54 | -0,2426 | 80,19 | 0,3383 | 50,52 | -0,2196 | 80,13 | 0,3513 | 50,63 | -0,164 |
| 80,31 | 0,3485 | 50,57 | -0,2426 | 80,16 | 0,3382 | 50,55 | -0,2196 | 80,1 | 0,3512 | 50,66 | -0,1641 |
| 80,28 | 0,3485 | 50,6 | -0,2427 | 80,13 | 0,3382 | 50,59 | -0,2196 | 80,07 | 0,3512 | 50,7 | -0,1642 |
| 80,25 | 0,3484 | 50,64 | -0,2427 | 80,09 | 0,3382 | 50,62 | -0,2197 | 80,03 | 0,3511 | 50,73 | -0,1643 |
| 80,21 | 0,3484 | 50,67 | -0,2427 | 80,06 | 0,3381 | 50,65 | -0,2197 | 80 | 0,3511 | 50,76 | -0,1644 |
| 80,18 | 0,3484 | 50,7 | -0,2427 | 80,03 | 0,3381 | 50,69 | -0,2197 | 79,97 | 0,351 | 50,8 | -0,1645 |
| 80,15 | 0,3483 | 50,74 | -0,2428 | 79,99 | 0,338 | 50,72 | -0,2198 | 79,93 | 0,351 | 50,83 | -0,1645 |
| 80,11 | 0,3483 | 50,77 | -0,2428 | 79,96 | 0,338 | 50,75 | -0,2198 | 79,9 | 0,3509 | 50,86 | -0,1646 |
| 80,08 | 0,3482 | 50,81 | -0,2428 | 79,93 | 0,338 | 50,79 | -0,2198 | 79,87 | 0,3509 | 50,9 | -0,1647 |
| 80,05 | 0,3482 | 50,84 | -0,2428 | 79,89 | 0,3379 | 50,82 | -0,2199 | 79,83 | 0,3508 | 50,93 | -0,1648 |
| 80,01 | 0,3482 | 50,87 | -0,2429 | 79,86 | 0,3379 | 50,85 | -0,2199 | 79,8 | 0,3508 | 50,96 | -0,1648 |
| 79,98 | 0,3481 | 50,9 | -0,2429 | 79,82 | 0,3378 | 50,89 | -0,2199 | 79,77 | 0,3507 | 51 | -0,1649 |
| 79,95 | 0,3481 | 50,94 | -0,2429 | 79,79 | 0,3378 | 50,92 | -0,2199 | 79,73 | 0,3507 | 51,03 | -0,165 |
| 79,91 | 0,348 | 50,97 | -0,243 | 79,76 | 0,3378 | 50,95 | -0,22 | 79,7 | 0,3506 | 51,06 | -0,165 |
| 79,88 | 0,348 | 51 | -0,243 | 79,72 | 0,3377 | 50,99 | -0,22 | 79,67 | 0,3506 | 51,1 | -0,1651 |
| 79,85 | 0,348 | 51,04 | -0,243 | 79,69 | 0,3377 | 51,02 | -0,22 | 79,63 | 0,3505 | 51,13 | -0,1652 |
| 79,81 | 0,3479 | 51,07 | -0,243 | 79,66 | 0,3377 | 51,05 | -0,2201 | 79,6 | 0,3505 | 51,16 | -0,1653 |
| 79,78 | 0,3479 | 51,1 | -0,2431 | 79,63 | 0,3376 | 51,09 | -0,2201 | 79,57 | 0,3504 | 51,2 | -0,1654 |
| 79,75 | 0,3478 | 51,14 | -0,2431 | 79,59 | 0,3376 | 51,12 | -0,2201 | 79,53 | 0,3504 | 51,23 | -0,1655 |
| 79,71 | 0,3478 | 51,17 | -0,2431 | 79,56 | 0,3376 | 51,15 | -0,2201 | 79,5 | 0,3503 | 51,26 | -0,1656 |
| 79,68 | 0,3478 | 51,2 | -0,2432 | 79,52 | 0,3375 | 51,19 | -0,2202 | 79,47 | 0,3503 | 51,3 | -0,1656 |
| 79,65 | 0,3477 | 51,24 | -0,2432 | 79,49 | 0,3375 | 51,22 | -0,2202 | 79,43 | 0,3502 | 51,33 | -0,1657 |
| 79,62 | 0,3477 | 51,27 | -0,2432 | 79,46 | 0,3374 | 51,25 | -0,2202 | 79,4 | 0,3502 | 51,36 | -0,1658 |
| 79,58 | 0,3477 | 51,3 | -0,2432 | 79,42 | 0,3374 | 51,29 | -0,2203 | 79,37 | 0,3501 | 51,4 | -0,1658 |
| 79,55 | 0,3476 | 51,34 | -0,2433 | 79,39 | 0,3373 | 51,32 | -0,2203 | 79,33 | 0,3501 | 51,43 | -0,1659 |
| 79,51 | 0,3476 | 51,37 | -0,2433 | 79,36 | 0,3373 | 51,35 | -0,2203 | 79,3 | 0,35 | 51,46 | -0,166 |
| 79,48 | 0,3475 | 51,41 | -0,2433 | 79,33 | 0,3373 | 51,39 | -0,2203 | 79,27 | 0,35 | 51,5 | -0,1661 |
| 79,45 | 0,3475 | 51,44 | -0,2433 | 79,29 | 0,3372 | 51,42 | -0,2204 | 79,23 | 0,3499 | 51,53 | -0,1662 |
| 79,41 | 0,3475 | 51,47 | -0,2434 | 79,26 | 0,3372 | 51,45 | -0,2204 | 79,2 | 0,3499 | 51,56 | -0,1664 |
| 79,38 | 0,3474 | 51,5 | -0,2434 | 79,22 | 0,3372 | 51,49 | -0,2204 | 79,17 | 0,3498 | 51,6 | -0,1666 |
| 79,35 | 0,3474 | 51,54 | -0,2434 | 79,19 | 0,3371 | 51,52 | -0,2205 | 79,13 | 0,3498 | 51,63 | -0,1667 |
| 79,31 | 0,3473 | 51,57 | -0,2435 | 79,16 | 0,3371 | 51,55 | -0,2205 | 79,1 | 0,3497 | 51,66 | -0,1668 |
| 79,28 | 0,3473 | 51,6 | -0,2435 | 79,12 | 0,337 | 51,59 | -0,2205 | 79,07 | 0,3497 | 51,7 | -0,1669 |
| 79,25 | 0,3473 | 51,64 | -0,2435 | 79,09 | 0,337 | 51,62 | -0,2205 | 79,03 | 0,3496 | 51,73 | -0,1669 |
| 79,21 | 0,3472 | 51,67 | -0,2435 | 79,06 | 0,337 | 51,65 | -0,2206 | 79 | 0,3496 | 51,76 | -0,167 |
| 79,18 | 0,3472 | 51,7 | -0,2436 | 79,03 | 0,3369 | 51,69 | -0,2206 | 78,97 | 0,3495 | 51,8 | -0,1671 |
| 79,15 | 0,3472 | 51,74 | -0,2436 | 78,99 | 0,3369 | 51,72 | -0,2206 | 78,93 | 0,3495 | 51,83 | -0,1672 |
| 79,11 | 0,3471 | 51,77 | -0,2436 | 78,96 | 0,3368 | 51,75 | -0,2207 | 78,9 | 0,3494 | 51,86 | -0,1673 |
| 79,08 | 0,3471 | 51,8 | -0,2437 | 78,92 | 0,3368 | 51,79 | -0,2207 | 78,87 | 0,3494 | 51,9 | -0,1674 |
| 79,05 | 0,347 | 51,84 | -0,2437 | 78,89 | 0,3368 | 51,82 | -0,2207 | 78,83 | 0,3493 | 51,93 | -0,1674 |
| 79,01 | 0,347 | 51,87 | -0,2437 | 78,86 | 0,3367 | 51,85 | -0,2207 | 78,8 | 0,3493 | 51,96 | -0,1675 |
| 78,98 | 0,347 | 51,9 | -0,2437 | 78,82 | 0,3367 | 51,89 | -0,2208 | 78,76 | 0,3492 | 52 | -0,1676 |
| 78,95 | 0,3469 | 51,94 | -0,2438 | 78,79 | 0,3367 | 51,92 | -0,2208 | 78,73 | 0,3492 | 52,03 | -0,1677 |
| 78,91 | 0,3469 | 51,97 | -0,2438 | 78,76 | 0,3366 | 51,95 | -0,2208 | 78,7 | 0,3491 | 52,06 | -0,1677 |
| 78,88 | 0,3468 | 52,01 | -0,2438 | 78,73 | 0,3366 | 51,99 | -0,2209 | 78,67 | 0,349 | 52,1 | -0,1678 |
| 78,85 | 0,3468 | 52,04 | -0,2439 | 78,69 | 0,3365 | 52,02 | -0,2209 | 78,63 | 0,349 | 52,13 | -0,1679 |
| 78,81 | 0,3468 | 52,07 | -0,2439 | 78,66 | 0,3365 | 52,05 | -0,2209 | 78,6 | 0,3489 | 52,16 | -0,168 |
| 78,78 | 0,3467 | 52,1 | -0,2439 | 78,62 | 0,3365 | 52,09 | -0,2209 | 78,56 | 0,3489 | 52,19 | -0,1681 |
| 78,75 | 0,3467 | 52,14 | -0,2439 | 78,59 | 0,3364 | 52,12 | -0,221 | 78,53 | 0,3488 | 52,23 | -0,1683 |
| 78,71 | 0,3466 | 52,17 | -0,244 | 78,56 | 0,3364 | 52,15 | -0,221 | 78,5 | 0,3488 | 52,26 | -0,1684 |
| 78,68 | 0,3466 | 52,21 | -0,244 | 78,52 | 0,3363 | 52,19 | -0,221 | 78,47 | 0,3487 | 52,29 | -0,1685 |
| 78,65 | 0,3466 | 52,24 | -0,244 | 78,49 | 0,3363 | 52,22 | -0,2211 | 78,43 | 0,3487 | 52,33 | -0,1686 |
| 78,61 | 0,3465 | 52,27 | -0,244 | 78,46 | 0,3363 | 52,25 | -0,2211 | 78,4 | 0,3486 | 52,36 | -0,1687 |
| 78,58 | 0,3465 | 52,3 | -0,2441 | 78,43 | 0,3362 | 52,29 | -0,2211 | 78,37 | 0,3486 | 52,4 | -0,1687 |
| 78,55 | 0,3464 | 52,34 | -0,2441 | 78,39 | 0,3362 | 52,32 | -0,2211 | 78,33 | 0,3485 | 52,43 | -0,1688 |
| 78,51 | 0,3464 | 52,37 | -0,2441 | 78,36 | 0,3361 | 52,35 | -0,2212 | 78,3 | 0,3485 | 52,46 | -0,1689 |
| 78,48 | 0,3464 | 52,4 | -0,2442 | 78,32 | 0,3361 | 52,39 | -0,2212 | 78,27 | 0,3484 | 52,49 | -0,169 |
| 78,45 | 0,3463 | 52,44 | -0,2442 | 78,29 | 0,3361 | 52,42 | -0,2212 | 78,23 | 0,3484 | 52,53 | -0,1691 |
| 78,41 | 0,3463 | 52,47 | -0,2442 | 78,26 | 0,336 | 52,45 | -0,2213 | 78,2 | 0,3483 | 52,56 | -0,1692 |
| 78,38 | 0,3462 | 52,5 | -0,2442 | 78,22 | 0,336 | 52,49 | -0,2213 | 78,17 | 0,3483 | 52,6 | -0,1692 |
| 78,35 | 0,3462 | 52,54 | -0,2443 | 78,19 | 0,336 | 52,52 | -0,2213 | 78,13 | 0,3482 | 52,63 | -0,1693 |
| 78,31 | 0,3462 | 52,57 | -0,2443 | 78,16 | 0,3359 | 52,55 | -0,2214 | 78,1 | 0,3482 | 52,66 | -0,1695 |
| 78,28 | 0,3461 | 52,61 | -0,2443 | 78,12 | 0,3359 | 52,59 | -0,2214 | 78,07 | 0,3481 | 52,69 | -0,1696 |
| 78,25 | 0,3461 | 52,64 | -0,2444 | 78,09 | 0,3358 | 52,62 | -0,2214 | 78,03 | 0,3481 | 52,73 | -0,1697 |
| 78,21 | 0,346 | 52,67 | -0,2444 | 78,06 | 0,3358 | 52,65 | -0,2214 | 78 | 0,348 | 52,76 | -0,1698 |
| 78,18 | 0,346 | 52,71 | -0,2444 | 78,02 | 0,3358 | 52,69 | -0,2215 | 77,97 | 0,348 | 52,79 | -0,1699 |
| 78,15 | 0,346 | 52,74 | -0,2444 | 77,99 | 0,3357 | 52,72 | -0,2215 | 77,93 | 0,3479 | 52,83 | -0,17 |
| 78,11 | 0,3459 | 52,77 | -0,2445 | 77,96 | 0,3357 | 52,75 | -0,2215 | 77,9 | 0,3479 | 52,86 | -0,1702 |
| 78,08 | 0,3459 | 52,8 | -0,2445 | 77,92 | 0,3356 | 52,79 | -0,2216 | 77,86 | 0,3478 | 52,9 | -0,1703 |
| 78,05 | 0,3458 | 52,84 | -0,2445 | 77,89 | 0,3356 | 52,82 | -0,2216 | 77,83 | 0,3478 | 52,93 | -0,1704 |
| 78,01 | 0,3458 | 52,87 | -0,2446 | 77,86 | 0,3356 | 52,85 | -0,2216 | 77,8 | 0,3477 | 52,96 | -0,1704 |
| 77,98 | 0,3458 | 52,91 | -0,2446 | 77,82 | 0,3355 | 52,89 | -0,2217 | 77,77 | 0,3477 | 52,99 | -0,1705 |
| 77,95 | 0,3457 | 52,94 | -0,2446 | 77,79 | 0,3355 | 52,92 | -0,2217 | 77,73 | 0,3476 | 53,03 | -0,1706 |
| 77,91 | 0,3457 | 52,97 | -0,2446 | 77,76 | 0,3354 | 52,96 | -0,2217 | 77,7 | 0,3475 | 53,06 | -0,1707 |
| 77,88 | 0,3456 | 53 | -0,2447 | 77,72 | 0,3354 | 52,99 | -0,2217 | 77,67 | 0,3475 | 53,09 | -0,1708 |
| 77,85 | 0,3456 | 53,04 | -0,2447 | 77,69 | 0,3354 | 53,02 | -0,2218 | 77,63 | 0,3474 | 53,13 | -0,1709 |
| 77,81 | 0,3456 | 53,07 | -0,2447 | 77,66 | 0,3353 | 53,05 | -0,2218 | 77,6 | 0,3474 | 53,16 | -0,1709 |
| 77,78 | 0,3455 | 53,11 | -0,2448 | 77,62 | 0,3353 | 53,09 | -0,2218 | 77,56 | 0,3473 | 53,19 | -0,1711 |
| 77,75 | 0,3455 | 53,14 | -0,2448 | 77,59 | 0,3352 | 53,12 | -0,2219 | 77,53 | 0,3473 | 53,23 | -0,1712 |
| 77,71 | 0,3454 | 53,17 | -0,2448 | 77,56 | 0,3352 | 53,16 | -0,2219 | 77,5 | 0,3472 | 53,26 | -0,1714 |
| 77,68 | 0,3454 | 53,21 | -0,2448 | 77,52 | 0,3352 | 53,19 | -0,2219 | 77,47 | 0,3472 | 53,29 | -0,1715 |
| 77,65 | 0,3453 | 53,24 | -0,2449 | 77,49 | 0,3351 | 53,22 | -0,2219 | 77,43 | 0,3471 | 53,33 | -0,1716 |
| 77,61 | 0,3453 | 53,27 | -0,2449 | 77,46 | 0,3351 | 53,25 | -0,222 | 77,4 | 0,3471 | 53,36 | -0,1717 |
| 77,58 | 0,3453 | 53,31 | -0,2449 | 77,42 | 0,335 | 53,29 | -0,222 | 77,37 | 0,347 | 53,39 | -0,1718 |
| 77,55 | 0,3452 | 53,34 | -0,2449 | 77,39 | 0,335 | 53,32 | -0,222 | 77,33 | 0,347 | 53,43 | -0,172 |
| 77,51 | 0,3452 | 53,37 | -0,245 | 77,36 | 0,335 | 53,36 | -0,2221 | 77,3 | 0,3469 | 53,46 | -0,1721 |
| 77,48 | 0,3451 | 53,41 | -0,245 | 77,32 | 0,3349 | 53,39 | -0,2221 | 77,26 | 0,3469 | 53,49 | -0,1723 |
| 77,45 | 0,3451 | 53,44 | -0,245 | 77,29 | 0,3349 | 53,42 | -0,2221 | 77,23 | 0,3468 | 53,53 | -0,1724 |
| 77,41 | 0,3451 | 53,47 | -0,2451 | 77,26 | 0,3348 | 53,45 | -0,2221 | 77,2 | 0,3468 | 53,56 | -0,1726 |
| 77,38 | 0,345 | 53,5 | -0,2451 | 77,22 | 0,3348 | 53,49 | -0,2222 | 77,17 | 0,3467 | 53,59 | -0,1728 |
| 77,35 | 0,345 | 53,54 | -0,2451 | 77,19 | 0,3348 | 53,52 | -0,2222 | 77,13 | 0,3467 | 53,63 | -0,1729 |
| 77,31 | 0,345 | 53,57 | -0,2451 | 77,16 | 0,3347 | 53,55 | -0,2222 | 77,1 | 0,3466 | 53,66 | -0,173 |
| 77,28 | 0,3449 | 53,61 | -0,2452 | 77,12 | 0,3347 | 53,59 | -0,2223 | 77,06 | 0,3466 | 53,69 | -0,1731 |
| 77,25 | 0,3449 | 53,64 | -0,2452 | 77,09 | 0,3346 | 53,62 | -0,2223 | 77,03 | 0,3465 | 53,73 | -0,1731 |
| 77,21 | 0,3448 | 53,67 | -0,2452 | 77,06 | 0,3346 | 53,66 | -0,2223 | 77 | 0,3465 | 53,76 | -0,1733 |
| 77,18 | 0,3448 | 53,71 | -0,2453 | 77,02 | 0,3346 | 53,69 | -0,2224 | 76,97 | 0,3464 | 53,79 | -0,1734 |
| 77,15 | 0,3448 | 53,74 | -0,2453 | 76,99 | 0,3345 | 53,72 | -0,2224 | 76,93 | 0,3463 | 53,83 | -0,1735 |
| 77,11 | 0,3447 | 53,77 | -0,2453 | 76,96 | 0,3345 | 53,76 | -0,2224 | 76,9 | 0,3463 | 53,86 | -0,1737 |
| 77,08 | 0,3447 | 53,81 | -0,2454 | 76,92 | 0,3344 | 53,79 | -0,2225 | 76,86 | 0,3462 | 53,89 | -0,1738 |
| 77,05 | 0,3446 | 53,84 | -0,2454 | 76,89 | 0,3344 | 53,82 | -0,2225 | 76,83 | 0,3462 | 53,93 | -0,1739 |
| 77,01 | 0,3446 | 53,87 | -0,2454 | 76,86 | 0,3343 | 53,85 | -0,2225 | 76,8 | 0,3461 | 53,96 | -0,1741 |
| 76,98 | 0,3446 | 53,91 | -0,2454 | 76,82 | 0,3343 | 53,89 | -0,2225 | 76,77 | 0,3461 | 53,99 | -0,1742 |
| 76,95 | 0,3445 | 53,94 | -0,2455 | 76,79 | 0,3343 | 53,92 | -0,2226 | 76,73 | 0,346 | 54,03 | -0,1743 |
| 76,91 | 0,3445 | 53,97 | -0,2455 | 76,76 | 0,3342 | 53,95 | -0,2226 | 76,7 | 0,346 | 54,06 | -0,1744 |
| 76,88 | 0,3444 | 54,01 | -0,2455 | 76,72 | 0,3342 | 53,99 | -0,2226 | 76,67 | 0,3459 | 54,09 | -0,1744 |
| 76,85 | 0,3444 | 54,04 | -0,2456 | 76,69 | 0,3341 | 54,02 | -0,2227 | 76,63 | 0,3459 | 54,13 | -0,1745 |
| 76,81 | 0,3444 | 54,07 | -0,2456 | 76,66 | 0,3341 | 54,05 | -0,2227 | 76,6 | 0,3458 | 54,16 | -0,1746 |
| 76,78 | 0,3443 | 54,11 | -0,2456 | 76,62 | 0,3341 | 54,09 | -0,2227 | 76,57 | 0,3458 | 54,19 | -0,1748 |
| 76,75 | 0,3443 | 54,14 | -0,2457 | 76,59 | 0,334 | 54,12 | -0,2227 | 76,53 | 0,3457 | 54,23 | -0,175 |
| 76,71 | 0,3442 | 54,17 | -0,2457 | 76,56 | 0,334 | 54,16 | -0,2228 | 76,5 | 0,3457 | 54,26 | -0,1753 |
| 76,68 | 0,3442 | 54,21 | -0,2457 | 76,52 | 0,3339 | 54,19 | -0,2228 | 76,46 | 0,3456 | 54,29 | -0,1754 |
| 76,65 | 0,3441 | 54,24 | -0,2457 | 76,49 | 0,3339 | 54,22 | -0,2228 | 76,43 | 0,3456 | 54,33 | -0,1755 |
| 76,61 | 0,3441 | 54,27 | -0,2458 | 76,46 | 0,3339 | 54,25 | -0,2229 | 76,4 | 0,3455 | 54,36 | -0,1756 |
| 76,58 | 0,3441 | 54,3 | -0,2458 | 76,42 | 0,3338 | 54,29 | -0,2229 | 76,36 | 0,3454 | 54,39 | -0,1757 |
| 76,55 | 0,344 | 54,34 | -0,2458 | 76,39 | 0,3338 | 54,32 | -0,2229 | 76,33 | 0,3454 | 54,43 | -0,1759 |
| 76,51 | 0,344 | 54,37 | -0,2459 | 76,36 | 0,3337 | 54,35 | -0,223 | 76,3 | 0,3453 | 54,46 | -0,1761 |
| 76,48 | 0,3439 | 54,41 | -0,2459 | 76,32 | 0,3337 | 54,39 | -0,223 | 76,26 | 0,3453 | 54,49 | -0,1762 |
| 76,45 | 0,3439 | 54,44 | -0,2459 | 76,29 | 0,3337 | 54,42 | -0,223 | 76,23 | 0,3452 | 54,53 | -0,1763 |
| 76,41 | 0,3438 | 54,47 | -0,246 | 76,26 | 0,3336 | 54,46 | -0,2231 | 76,2 | 0,3452 | 54,56 | -0,1764 |
| 76,38 | 0,3438 | 54,51 | -0,246 | 76,22 | 0,3336 | 54,49 | -0,2231 | 76,17 | 0,3451 | 54,59 | -0,1765 |
| 76,35 | 0,3437 | 54,54 | -0,246 | 76,19 | 0,3335 | 54,52 | -0,2231 | 76,13 | 0,3451 | 54,63 | -0,1767 |
| 76,31 | 0,3437 | 54,57 | -0,246 | 76,16 | 0,3335 | 54,56 | -0,2231 | 76,1 | 0,345 | 54,66 | -0,1769 |
| 76,28 | 0,3437 | 54,61 | -0,2461 | 76,12 | 0,3335 | 54,59 | -0,2232 | 76,07 | 0,345 | 54,69 | -0,177 |
| 76,25 | 0,3436 | 54,64 | -0,2461 | 76,09 | 0,3334 | 54,62 | -0,2232 | 76,03 | 0,3449 | 54,72 | -0,1771 |
| 76,21 | 0,3436 | 54,67 | -0,2461 | 76,06 | 0,3334 | 54,65 | -0,2232 | 76 | 0,3449 | 54,76 | -0,1772 |
| 76,18 | 0,3435 | 54,71 | -0,2462 | 76,02 | 0,3333 | 54,69 | -0,2233 | 75,96 | 0,3448 | 54,79 | -0,1773 |
| 76,15 | 0,3435 | 54,74 | -0,2462 | 75,99 | 0,3333 | 54,72 | -0,2233 | 75,93 | 0,3448 | 54,83 | -0,1774 |
| 76,11 | 0,3435 | 54,77 | -0,2462 | 75,96 | 0,3333 | 54,76 | -0,2233 | 75,9 | 0,3447 | 54,86 | -0,1776 |
| 76,08 | 0,3434 | 54,81 | -0,2463 | 75,92 | 0,3332 | 54,79 | -0,2234 | 75,86 | 0,3447 | 54,89 | -0,1777 |
| 76,04 | 0,3434 | 54,84 | -0,2463 | 75,89 | 0,3332 | 54,82 | -0,2234 | 75,83 | 0,3446 | 54,92 | -0,1779 |
| 76,01 | 0,3433 | 54,87 | -0,2463 | 75,86 | 0,3331 | 54,85 | -0,2234 | 75,8 | 0,3445 | 54,96 | -0,178 |
| 75,98 | 0,3433 | 54,91 | -0,2464 | 75,82 | 0,3331 | 54,89 | -0,2235 | 75,77 | 0,3445 | 54,99 | -0,1781 |
| 75,95 | 0,3432 | 54,94 | -0,2464 | 75,79 | 0,3331 | 54,92 | -0,2235 | 75,73 | 0,3444 | 55,02 | -0,1782 |
| 75,91 | 0,3432 | 54,97 | -0,2464 | 75,76 | 0,333 | 54,96 | -0,2235 | 75,7 | 0,3444 | 55,06 | -0,1784 |
| 75,88 | 0,3432 | 55,01 | -0,2464 | 75,72 | 0,333 | 54,99 | -0,2236 | 75,67 | 0,3443 | 55,09 | -0,1786 |
| 75,84 | 0,3431 | 55,04 | -0,2465 | 75,69 | 0,3329 | 55,02 | -0,2236 | 75,63 | 0,3443 | 55,12 | -0,1787 |
| 75,81 | 0,3431 | 55,07 | -0,2465 | 75,66 | 0,3329 | 55,06 | -0,2236 | 75,6 | 0,3442 | 55,16 | -0,1789 |
| 75,78 | 0,343 | 55,11 | -0,2465 | 75,62 | 0,3329 | 55,09 | -0,2236 | 75,57 | 0,3442 | 55,19 | -0,179 |
| 75,75 | 0,343 | 55,14 | -0,2466 | 75,59 | 0,3328 | 55,12 | -0,2237 | 75,53 | 0,3441 | 55,22 | -0,1791 |
| 75,71 | 0,343 | 55,17 | -0,2466 | 75,56 | 0,3328 | 55,16 | -0,2237 | 75,5 | 0,3441 | 55,26 | -0,1792 |
| 75,68 | 0,3429 | 55,21 | -0,2466 | 75,52 | 0,3327 | 55,19 | -0,2237 | 75,47 | 0,344 | 55,29 | -0,1793 |
| 75,65 | 0,3429 | 55,24 | -0,2467 | 75,49 | 0,3327 | 55,22 | -0,2238 | 75,43 | 0,344 | 55,32 | -0,1795 |
| 75,61 | 0,3428 | 55,27 | -0,2467 | 75,46 | 0,3327 | 55,25 | -0,2238 | 75,4 | 0,3439 | 55,36 | -0,1797 |
| 75,58 | 0,3428 | 55,31 | -0,2467 | 75,42 | 0,3326 | 55,29 | -0,2238 | 75,36 | 0,3439 | 55,39 | -0,1799 |
| 75,55 | 0,3428 | 55,34 | -0,2467 | 75,39 | 0,3326 | 55,32 | -0,2239 | 75,33 | 0,3438 | 55,42 | -0,1801 |
| 75,51 | 0,3427 | 55,37 | -0,2468 | 75,36 | 0,3325 | 55,36 | -0,2239 | 75,3 | 0,3438 | 55,46 | -0,1803 |
| 75,48 | 0,3427 | 55,41 | -0,2468 | 75,32 | 0,3325 | 55,39 | -0,2239 | 75,26 | 0,3437 | 55,49 | -0,1805 |
| 75,44 | 0,3426 | 55,44 | -0,2468 | 75,29 | 0,3325 | 55,42 | -0,224 | 75,23 | 0,3436 | 55,52 | -0,1806 |
| 75,41 | 0,3426 | 55,47 | -0,2469 | 75,26 | 0,3324 | 55,45 | -0,224 | 75,2 | 0,3436 | 55,56 | -0,1807 |
| 75,38 | 0,3426 | 55,51 | -0,2469 | 75,22 | 0,3324 | 55,49 | -0,224 | 75,16 | 0,3435 | 55,59 | -0,1808 |
| 75,34 | 0,3425 | 55,54 | -0,2469 | 75,19 | 0,3323 | 55,52 | -0,2241 | 75,13 | 0,3435 | 55,62 | -0,181 |
| 75,31 | 0,3425 | 55,57 | -0,247 | 75,16 | 0,3323 | 55,56 | -0,2241 | 75,1 | 0,3434 | 55,66 | -0,1812 |
| 75,28 | 0,3424 | 55,61 | -0,247 | 75,12 | 0,3323 | 55,59 | -0,2241 | 75,06 | 0,3434 | 55,69 | -0,1813 |
| 75,24 | 0,3424 | 55,64 | -0,247 | 75,09 | 0,3322 | 55,62 | -0,2241 | 75,03 | 0,3433 | 55,72 | -0,1814 |
| 75,21 | 0,3424 | 55,67 | -0,2471 | 75,06 | 0,3322 | 55,65 | -0,2242 | 75 | 0,3433 | 55,76 | -0,1815 |
| 75,18 | 0,3423 | 55,71 | -0,2471 | 75,02 | 0,3321 | 55,69 | -0,2242 | 74,96 | 0,3432 | 55,79 | -0,1816 |
| 75,14 | 0,3423 | 55,74 | -0,2471 | 74,99 | 0,3321 | 55,72 | -0,2242 | 74,93 | 0,3432 | 55,82 | -0,1818 |
| 75,11 | 0,3422 | 55,77 | -0,2472 | 74,96 | 0,3321 | 55,76 | -0,2243 | 74,9 | 0,3431 | 55,86 | -0,182 |
| 75,08 | 0,3422 | 55,81 | -0,2472 | 74,92 | 0,332 | 55,79 | -0,2243 | 74,87 | 0,3431 | 55,89 | -0,1821 |
| 75,04 | 0,3421 | 55,84 | -0,2472 | 74,89 | 0,332 | 55,82 | -0,2243 | 74,83 | 0,343 | 55,92 | -0,1822 |
| 75,01 | 0,3421 | 55,87 | -0,2473 | 74,86 | 0,3319 | 55,86 | -0,2244 | 74,8 | 0,3429 | 55,96 | -0,1823 |
| 74,98 | 0,3421 | 55,91 | -0,2473 | 74,82 | 0,3319 | 55,89 | -0,2244 | 74,77 | 0,3429 | 55,99 | -0,1824 |
| 74,95 | 0,342 | 55,94 | -0,2473 | 74,79 | 0,3319 | 55,92 | -0,2244 | 74,73 | 0,3428 | 56,02 | -0,1826 |
| 74,91 | 0,342 | 55,97 | -0,2474 | 74,76 | 0,3318 | 55,95 | -0,2245 | 74,7 | 0,3428 | 56,06 | -0,1827 |
| 74,88 | 0,3419 | 56,01 | -0,2474 | 74,72 | 0,3318 | 55,99 | -0,2245 | 74,67 | 0,3427 | 56,09 | -0,1828 |
| 74,84 | 0,3419 | 56,04 | -0,2474 | 74,69 | 0,3317 | 56,02 | -0,2245 | 74,63 | 0,3427 | 56,12 | -0,183 |
| 74,81 | 0,3419 | 56,07 | -0,2474 | 74,66 | 0,3317 | 56,05 | -0,2246 | 74,6 | 0,3426 | 56,16 | -0,1832 |
| 74,78 | 0,3418 | 56,11 | -0,2475 | 74,62 | 0,3317 | 56,09 | -0,2246 | 74,56 | 0,3426 | 56,19 | -0,1835 |
| 74,75 | 0,3418 | 56,14 | -0,2475 | 74,59 | 0,3316 | 56,12 | -0,2246 | 74,53 | 0,3425 | 56,22 | -0,1838 |
| 74,71 | 0,3417 | 56,17 | -0,2475 | 74,56 | 0,3316 | 56,15 | -0,2247 | 74,5 | 0,3425 | 56,26 | -0,184 |
| 74,68 | 0,3417 | 56,21 | -0,2476 | 74,52 | 0,3315 | 56,19 | -0,2247 | 74,47 | 0,3424 | 56,29 | -0,1841 |
| 74,64 | 0,3417 | 56,24 | -0,2476 | 74,49 | 0,3315 | 56,22 | -0,2247 | 74,43 | 0,3424 | 56,32 | -0,1842 |
| 74,61 | 0,3416 | 56,27 | -0,2476 | 74,46 | 0,3315 | 56,25 | -0,2248 | 74,4 | 0,3423 | 56,35 | -0,1843 |
| 74,58 | 0,3416 | 56,31 | -0,2477 | 74,42 | 0,3314 | 56,29 | -0,2248 | 74,36 | 0,3423 | 56,39 | -0,1845 |
| 74,54 | 0,3415 | 56,34 | -0,2477 | 74,39 | 0,3314 | 56,32 | -0,2248 | 74,33 | 0,3422 | 56,42 | -0,1848 |
| 74,51 | 0,3415 | 56,37 | -0,2477 | 74,36 | 0,3313 | 56,35 | -0,2249 | 74,3 | 0,3422 | 56,46 | -0,185 |
| 74,48 | 0,3415 | 56,41 | -0,2478 | 74,32 | 0,3313 | 56,39 | -0,2249 | 74,26 | 0,3421 | 56,49 | -0,1851 |
| 74,45 | 0,3414 | 56,44 | -0,2478 | 74,29 | 0,3313 | 56,42 | -0,2249 | 74,23 | 0,342 | 56,52 | -0,1852 |
| 74,41 | 0,3414 | 56,47 | -0,2478 | 74,26 | 0,3312 | 56,45 | -0,225 | 74,2 | 0,342 | 56,56 | -0,1853 |
| 74,38 | 0,3413 | 56,51 | -0,2479 | 74,22 | 0,3312 | 56,49 | -0,225 | 74,17 | 0,3419 | 56,59 | -0,1854 |
| 74,34 | 0,3413 | 56,54 | -0,2479 | 74,19 | 0,3311 | 56,52 | -0,225 | 74,13 | 0,3419 | 56,62 | -0,1855 |
| 74,31 | 0,3413 | 56,57 | -0,2479 | 74,16 | 0,3311 | 56,55 | -0,2251 | 74,1 | 0,3418 | 56,65 | -0,1858 |
| 74,28 | 0,3412 | 56,61 | -0,248 | 74,12 | 0,331 | 56,59 | -0,2251 | 74,06 | 0,3418 | 56,69 | -0,1861 |
| 74,25 | 0,3412 | 56,64 | -0,248 | 74,09 | 0,331 | 56,62 | -0,2251 | 74,03 | 0,3417 | 56,72 | -0,1863 |
| 74,21 | 0,3411 | 56,67 | -0,248 | 74,06 | 0,331 | 56,65 | -0,2252 | 74 | 0,3417 | 56,75 | -0,1865 |
| 74,18 | 0,3411 | 56,71 | -0,2481 | 74,02 | 0,3309 | 56,69 | -0,2252 | 73,96 | 0,3416 | 56,79 | -0,1867 |
| 74,15 | 0,3411 | 56,74 | -0,2481 | 73,99 | 0,3309 | 56,72 | -0,2253 | 73,93 | 0,3416 | 56,82 | -0,1868 |
| 74,11 | 0,341 | 56,77 | -0,2481 | 73,96 | 0,3308 | 56,76 | -0,2253 | 73,9 | 0,3415 | 56,86 | -0,1869 |
| 74,08 | 0,341 | 56,81 | -0,2482 | 73,92 | 0,3308 | 56,79 | -0,2253 | 73,86 | 0,3415 | 56,89 | -0,187 |
| 74,05 | 0,3409 | 56,84 | -0,2482 | 73,89 | 0,3308 | 56,82 | -0,2254 | 73,83 | 0,3414 | 56,92 | -0,1871 |
| 74,01 | 0,3409 | 56,87 | -0,2482 | 73,86 | 0,3307 | 56,85 | -0,2254 | 73,8 | 0,3413 | 56,95 | -0,1872 |
| 73,98 | 0,3408 | 56,91 | -0,2483 | 73,82 | 0,3307 | 56,89 | -0,2254 | 73,76 | 0,3413 | 56,99 | -0,1874 |
| 73,94 | 0,3408 | 56,94 | -0,2483 | 73,79 | 0,3306 | 56,92 | -0,2255 | 73,73 | 0,3412 | 57,02 | -0,1876 |
| 73,91 | 0,3408 | 56,97 | -0,2483 | 73,76 | 0,3306 | 56,95 | -0,2255 | 73,7 | 0,3412 | 57,05 | -0,1878 |
| 73,88 | 0,3407 | 57,01 | -0,2484 | 73,72 | 0,3306 | 56,99 | -0,2255 | 73,66 | 0,3411 | 57,09 | -0,1879 |
| 73,84 | 0,3407 | 57,04 | -0,2484 | 73,69 | 0,3305 | 57,02 | -0,2255 | 73,63 | 0,3411 | 57,12 | -0,1881 |
| 73,81 | 0,3406 | 57,07 | -0,2485 | 73,66 | 0,3305 | 57,06 | -0,2256 | 73,6 | 0,341 | 57,15 | -0,1883 |
| 73,78 | 0,3406 | 57,11 | -0,2485 | 73,62 | 0,3304 | 57,09 | -0,2256 | 73,56 | 0,341 | 57,19 | -0,1884 |
| 73,74 | 0,3406 | 57,14 | -0,2485 | 73,59 | 0,3304 | 57,12 | -0,2256 | 73,53 | 0,3409 | 57,22 | -0,1886 |
| 73,71 | 0,3405 | 57,17 | -0,2486 | 73,55 | 0,3304 | 57,15 | -0,2257 | 73,5 | 0,3409 | 57,25 | -0,1887 |
| 73,68 | 0,3405 | 57,21 | -0,2486 | 73,52 | 0,3303 | 57,19 | -0,2257 | 73,46 | 0,3408 | 57,29 | -0,1888 |
| 73,64 | 0,3404 | 57,24 | -0,2486 | 73,49 | 0,3303 | 57,22 | -0,2258 | 73,43 | 0,3408 | 57,32 | -0,189 |
| 73,61 | 0,3404 | 57,27 | -0,2487 | 73,46 | 0,3302 | 57,25 | -0,2258 | 73,4 | 0,3407 | 57,35 | -0,1891 |
| 73,58 | 0,3404 | 57,31 | -0,2487 | 73,42 | 0,3302 | 57,29 | -0,2258 | 73,36 | 0,3407 | 57,39 | -0,1892 |
| 73,54 | 0,3403 | 57,34 | -0,2487 | 73,39 | 0,3302 | 57,32 | -0,2259 | 73,33 | 0,3406 | 57,42 | -0,1894 |
| 73,51 | 0,3403 | 57,37 | -0,2488 | 73,36 | 0,3301 | 57,35 | -0,2259 | 73,3 | 0,3406 | 57,45 | -0,1896 |
| 73,48 | 0,3402 | 57,41 | -0,2488 | 73,32 | 0,3301 | 57,39 | -0,2259 | 73,26 | 0,3405 | 57,49 | -0,1898 |
| 73,44 | 0,3402 | 57,44 | -0,2488 | 73,29 | 0,33 | 57,42 | -0,226 | 73,23 | 0,3404 | 57,52 | -0,19 |
| 73,41 | 0,3401 | 57,47 | -0,2489 | 73,26 | 0,33 | 57,45 | -0,226 | 73,2 | 0,3404 | 57,55 | -0,1902 |
| 73,38 | 0,3401 | 57,51 | -0,2489 | 73,22 | 0,33 | 57,49 | -0,226 | 73,16 | 0,3403 | 57,59 | -0,1903 |
| 73,34 | 0,3401 | 57,54 | -0,2489 | 73,19 | 0,3299 | 57,52 | -0,2261 | 73,13 | 0,3403 | 57,62 | -0,1904 |
| 73,31 | 0,34 | 57,57 | -0,249 | 73,16 | 0,3299 | 57,55 | -0,2261 | 73,1 | 0,3402 | 57,65 | -0,1905 |
| 73,28 | 0,34 | 57,61 | -0,249 | 73,12 | 0,3298 | 57,59 | -0,2261 | 73,06 | 0,3402 | 57,69 | -0,1906 |
| 73,24 | 0,3399 | 57,64 | -0,249 | 73,09 | 0,3298 | 57,62 | -0,2262 | 73,03 | 0,3401 | 57,72 | -0,1907 |
| 73,21 | 0,3399 | 57,67 | -0,2491 | 73,06 | 0,3298 | 57,65 | -0,2262 | 73 | 0,3401 | 57,75 | -0,1908 |
| 73,18 | 0,3399 | 57,71 | -0,2491 | 73,02 | 0,3297 | 57,69 | -0,2262 | 72,96 | 0,34 | 57,79 | -0,191 |
| 73,14 | 0,3398 | 57,74 | -0,2491 | 72,99 | 0,3297 | 57,72 | -0,2263 | 72,93 | 0,34 | 57,82 | -0,1912 |
| 73,11 | 0,3398 | 57,77 | -0,2492 | 72,95 | 0,3297 | 57,75 | -0,2263 | 72,9 | 0,3399 | 57,85 | -0,1913 |
| 73,08 | 0,3397 | 57,81 | -0,2492 | 72,92 | 0,3296 | 57,79 | -0,2263 | 72,86 | 0,3399 | 57,89 | -0,1914 |
| 73,04 | 0,3397 | 57,84 | -0,2492 | 72,89 | 0,3296 | 57,82 | -0,2264 | 72,83 | 0,3398 | 57,92 | -0,1915 |
| 73,01 | 0,3397 | 57,87 | -0,2493 | 72,86 | 0,3295 | 57,85 | -0,2264 | 72,8 | 0,3398 | 57,95 | -0,1916 |
| 72,98 | 0,3396 | 57,91 | -0,2493 | 72,82 | 0,3295 | 57,89 | -0,2265 | 72,76 | 0,3397 | 57,99 | -0,1917 |
| 72,94 | 0,3396 | 57,94 | -0,2493 | 72,79 | 0,3295 | 57,92 | -0,2265 | 72,73 | 0,3397 | 58,02 | -0,1918 |
| 72,91 | 0,3395 | 57,97 | -0,2494 | 72,75 | 0,3294 | 57,95 | -0,2265 | 72,7 | 0,3396 | 58,05 | -0,1919 |
| 72,88 | 0,3395 | 58,01 | -0,2494 | 72,72 | 0,3294 | 57,99 | -0,2266 | 72,66 | 0,3396 | 58,09 | -0,192 |
| 72,84 | 0,3395 | 58,04 | -0,2495 | 72,69 | 0,3293 | 58,02 | -0,2266 | 72,63 | 0,3395 | 58,12 | -0,1922 |
| 72,81 | 0,3394 | 58,07 | -0,2495 | 72,65 | 0,3293 | 58,05 | -0,2266 | 72,6 | 0,3395 | 58,15 | -0,1926 |
| 72,78 | 0,3394 | 58,11 | -0,2495 | 72,62 | 0,3293 | 58,09 | -0,2267 | 72,56 | 0,3394 | 58,19 | -0,1929 |
| 72,74 | 0,3393 | 58,14 | -0,2496 | 72,59 | 0,3292 | 58,12 | -0,2267 | 72,53 | 0,3394 | 58,22 | -0,1931 |
| 72,71 | 0,3393 | 58,17 | -0,2496 | 72,55 | 0,3292 | 58,15 | -0,2267 | 72,5 | 0,3393 | 58,25 | -0,1932 |
| 72,68 | 0,3393 | 58,21 | -0,2496 | 72,52 | 0,3292 | 58,19 | -0,2268 | 72,46 | 0,3393 | 58,29 | -0,1933 |
| 72,64 | 0,3392 | 58,24 | -0,2497 | 72,49 | 0,3291 | 58,22 | -0,2268 | 72,43 | 0,3392 | 58,32 | -0,1935 |
| 72,61 | 0,3392 | 58,27 | -0,2497 | 72,46 | 0,3291 | 58,25 | -0,2268 | 72,4 | 0,3392 | 58,35 | -0,1936 |
| 72,58 | 0,3391 | 58,31 | -0,2497 | 72,42 | 0,329 | 58,29 | -0,2269 | 72,36 | 0,3391 | 58,39 | -0,1938 |
| 72,54 | 0,3391 | 58,34 | -0,2498 | 72,39 | 0,329 | 58,32 | -0,2269 | 72,33 | 0,3391 | 58,42 | -0,194 |
| 72,51 | 0,3391 | 58,37 | -0,2498 | 72,36 | 0,329 | 58,35 | -0,2269 | 72,3 | 0,339 | 58,45 | -0,1942 |
| 72,48 | 0,339 | 58,41 | -0,2498 | 72,32 | 0,3289 | 58,39 | -0,227 | 72,26 | 0,339 | 58,49 | -0,1945 |
| 72,44 | 0,339 | 58,44 | -0,2499 | 72,29 | 0,3289 | 58,42 | -0,227 | 72,23 | 0,3389 | 58,52 | -0,1946 |
| 72,41 | 0,3389 | 58,47 | -0,2499 | 72,25 | 0,3288 | 58,45 | -0,2271 | 72,2 | 0,3389 | 58,55 | -0,1947 |
| 72,38 | 0,3389 | 58,51 | -0,2499 | 72,22 | 0,3288 | 58,49 | -0,2271 | 72,16 | 0,3388 | 58,59 | -0,1948 |
| 72,34 | 0,3389 | 58,54 | -0,25 | 72,19 | 0,3288 | 58,52 | -0,2271 | 72,13 | 0,3388 | 58,62 | -0,1949 |
| 72,31 | 0,3388 | 58,57 | -0,25 | 72,16 | 0,3287 | 58,55 | -0,2272 | 72,1 | 0,3387 | 58,65 | -0,195 |
| 72,28 | 0,3388 | 58,61 | -0,2501 | 72,12 | 0,3287 | 58,59 | -0,2272 | 72,06 | 0,3387 | 58,69 | -0,1951 |
| 72,24 | 0,3387 | 58,64 | -0,2501 | 72,09 | 0,3287 | 58,62 | -0,2272 | 72,03 | 0,3386 | 58,72 | -0,1952 |
| 72,21 | 0,3387 | 58,67 | -0,2501 | 72,05 | 0,3286 | 58,65 | -0,2273 | 72 | 0,3386 | 58,75 | -0,1953 |
| 72,18 | 0,3387 | 58,71 | -0,2502 | 72,02 | 0,3286 | 58,69 | -0,2273 | 71,96 | 0,3385 | 58,78 | -0,1954 |
| 72,14 | 0,3386 | 58,74 | -0,2502 | 71,99 | 0,3285 | 58,72 | -0,2273 | 71,93 | 0,3385 | 58,82 | -0,1955 |
| 72,11 | 0,3386 | 58,77 | -0,2502 | 71,96 | 0,3285 | 58,75 | -0,2274 | 71,9 | 0,3384 | 58,85 | -0,1956 |
| 72,08 | 0,3385 | 58,81 | -0,2503 | 71,92 | 0,3285 | 58,79 | -0,2274 | 71,86 | 0,3383 | 58,88 | -0,1957 |
| 72,04 | 0,3385 | 58,84 | -0,2503 | 71,89 | 0,3284 | 58,82 | -0,2274 | 71,83 | 0,3383 | 58,92 | -0,1959 |
| 72,01 | 0,3385 | 58,87 | -0,2504 | 71,86 | 0,3284 | 58,85 | -0,2275 | 71,8 | 0,3382 | 58,95 | -0,1961 |
| 71,98 | 0,3384 | 58,91 | -0,2504 | 71,82 | 0,3284 | 58,89 | -0,2275 | 71,76 | 0,3382 | 58,98 | -0,1962 |
| 71,94 | 0,3384 | 58,94 | -0,2504 | 71,79 | 0,3283 | 58,92 | -0,2276 | 71,73 | 0,3381 | 59,02 | -0,1964 |
| 71,91 | 0,3383 | 58,97 | -0,2505 | 71,76 | 0,3283 | 58,95 | -0,2276 | 71,7 | 0,3381 | 59,05 | -0,1965 |
| 71,88 | 0,3383 | 59,01 | -0,2505 | 71,72 | 0,3282 | 58,99 | -0,2276 | 71,66 | 0,338 | 59,08 | -0,1966 |
| 71,84 | 0,3383 | 59,04 | -0,2505 | 71,69 | 0,3282 | 59,02 | -0,2277 | 71,63 | 0,338 | 59,12 | -0,1968 |
| 71,81 | 0,3382 | 59,07 | -0,2506 | 71,65 | 0,3282 | 59,05 | -0,2277 | 71,6 | 0,3379 | 59,15 | -0,1969 |
| 71,78 | 0,3382 | 59,11 | -0,2506 | 71,62 | 0,3281 | 59,09 | -0,2277 | 71,56 | 0,3379 | 59,18 | -0,1971 |
| 71,74 | 0,3382 | 59,14 | -0,2506 | 71,59 | 0,3281 | 59,12 | -0,2278 | 71,53 | 0,3378 | 59,22 | -0,1972 |
| 71,71 | 0,3381 | 59,17 | -0,2507 | 71,55 | 0,3281 | 59,15 | -0,2278 | 71,5 | 0,3378 | 59,25 | -0,1973 |
| 71,68 | 0,3381 | 59,21 | -0,2507 | 71,52 | 0,328 | 59,19 | -0,2279 | 71,46 | 0,3377 | 59,28 | -0,1974 |
| 71,64 | 0,338 | 59,24 | -0,2508 | 71,49 | 0,328 | 59,22 | -0,2279 | 71,43 | 0,3377 | 59,32 | -0,1975 |
| 71,61 | 0,338 | 59,27 | -0,2508 | 71,45 | 0,3279 | 59,25 | -0,2279 | 71,4 | 0,3376 | 59,35 | -0,1977 |
| 71,58 | 0,338 | 59,31 | -0,2508 | 71,42 | 0,3279 | 59,29 | -0,228 | 71,36 | 0,3376 | 59,38 | -0,198 |
| 71,54 | 0,3379 | 59,34 | -0,2509 | 71,39 | 0,3279 | 59,32 | -0,228 | 71,33 | 0,3375 | 59,42 | -0,1982 |
| 71,51 | 0,3379 | 59,37 | -0,2509 | 71,36 | 0,3278 | 59,36 | -0,228 | 71,3 | 0,3375 | 59,45 | -0,1984 |
| 71,48 | 0,3378 | 59,41 | -0,2509 | 71,32 | 0,3278 | 59,39 | -0,2281 | 71,26 | 0,3374 | 59,48 | -0,1985 |
| 71,44 | 0,3378 | 59,44 | -0,251 | 71,29 | 0,3277 | 59,42 | -0,2281 | 71,23 | 0,3374 | 59,52 | -0,1986 |
| 71,41 | 0,3377 | 59,47 | -0,251 | 71,25 | 0,3277 | 59,45 | -0,2282 | 71,2 | 0,3373 | 59,55 | -0,1986 |
| 71,38 | 0,3377 | 59,5 | -0,251 | 71,22 | 0,3277 | 59,49 | -0,2282 | 71,16 | 0,3373 | 59,58 | -0,1987 |
| 71,34 | 0,3377 | 59,54 | -0,2511 | 71,19 | 0,3276 | 59,52 | -0,2282 | 71,13 | 0,3372 | 59,62 | -0,1988 |
| 71,31 | 0,3376 | 59,57 | -0,2511 | 71,15 | 0,3276 | 59,55 | -0,2283 | 71,1 | 0,3372 | 59,65 | -0,199 |
| 71,28 | 0,3376 | 59,61 | -0,2512 | 71,12 | 0,3276 | 59,59 | -0,2283 | 71,06 | 0,3371 | 59,68 | -0,199 |
| 71,24 | 0,3375 | 59,64 | -0,2512 | 71,09 | 0,3275 | 59,62 | -0,2283 | 71,03 | 0,3371 | 59,72 | -0,1991 |
| 71,21 | 0,3375 | 59,67 | -0,2512 | 71,05 | 0,3275 | 59,66 | -0,2284 | 71 | 0,337 | 59,75 | -0,1992 |
| 71,18 | 0,3375 | 59,7 | -0,2513 | 71,02 | 0,3274 | 59,69 | -0,2284 | 70,96 | 0,337 | 59,78 | -0,1993 |
| 71,14 | 0,3374 | 59,74 | -0,2513 | 70,99 | 0,3274 | 59,72 | -0,2284 | 70,93 | 0,3369 | 59,82 | -0,1995 |
| 71,11 | 0,3374 | 59,77 | -0,2513 | 70,95 | 0,3274 | 59,75 | -0,2285 | 70,9 | 0,3369 | 59,85 | -0,1996 |
| 71,08 | 0,3373 | 59,81 | -0,2514 | 70,92 | 0,3273 | 59,79 | -0,2285 | 70,86 | 0,3368 | 59,88 | -0,1997 |
| 71,04 | 0,3373 | 59,84 | -0,2514 | 70,89 | 0,3273 | 59,82 | -0,2285 | 70,83 | 0,3368 | 59,92 | -0,1998 |
| 71,01 | 0,3373 | 59,87 | -0,2514 | 70,86 | 0,3272 | 59,85 | -0,2286 | 70,8 | 0,3367 | 59,95 | -0,1999 |
| 70,98 | 0,3372 | 59,91 | -0,2515 | 70,82 | 0,3272 | 59,89 | -0,2286 | 70,76 | 0,3367 | 59,98 | -0,2 |
| 70,94 | 0,3372 | 59,94 | -0,2515 | 70,79 | 0,3272 | 59,92 | -0,2287 | 70,73 | 0,3366 | 60,02 | -0,2001 |
| 70,91 | 0,3371 | 59,97 | -0,2516 | 70,75 | 0,3271 | 59,95 | -0,2287 | 70,7 | 0,3366 | 60,05 | -0,2002 |
| 70,88 | 0,3371 | 60,01 | -0,2516 | 70,72 | 0,3271 | 59,99 | -0,2287 | 70,66 | 0,3365 | 60,08 | -0,2003 |
| 70,84 | 0,337 | 60,04 | -0,2516 | 70,69 | 0,327 | 60,02 | -0,2288 | 70,63 | 0,3364 | 60,12 | -0,2005 |
| 70,81 | 0,337 | 60,07 | -0,2517 | 70,65 | 0,327 | 60,05 | -0,2288 | 70,6 | 0,3364 | 60,15 | -0,2006 |
| 70,78 | 0,337 | 60,11 | -0,2517 | 70,62 | 0,327 | 60,09 | -0,2288 | 70,56 | 0,3363 | 60,18 | -0,2007 |
| 70,74 | 0,3369 | 60,14 | -0,2517 | 70,59 | 0,3269 | 60,12 | -0,2289 | 70,53 | 0,3363 | 60,22 | -0,2008 |
| 70,71 | 0,3369 | 60,17 | -0,2518 | 70,55 | 0,3269 | 60,15 | -0,2289 | 70,5 | 0,3362 | 60,25 | -0,2009 |
| 70,68 | 0,3368 | 60,2 | -0,2518 | 70,52 | 0,3268 | 60,19 | -0,2289 | 70,46 | 0,3362 | 60,28 | -0,201 |
| 70,64 | 0,3368 | 60,24 | -0,2519 | 70,49 | 0,3268 | 60,22 | -0,229 | 70,43 | 0,3361 | 60,32 | -0,2011 |
| 70,61 | 0,3367 | 60,27 | -0,2519 | 70,45 | 0,3268 | 60,25 | -0,229 | 70,4 | 0,3361 | 60,35 | -0,2013 |
| 70,58 | 0,3367 | 60,31 | -0,2519 | 70,42 | 0,3267 | 60,29 | -0,229 | 70,36 | 0,336 | 60,38 | -0,2016 |
| 70,54 | 0,3366 | 60,34 | -0,252 | 70,39 | 0,3267 | 60,32 | -0,2291 | 70,33 | 0,336 | 60,42 | -0,2018 |
| 70,51 | 0,3366 | 60,37 | -0,252 | 70,35 | 0,3266 | 60,35 | -0,2291 | 70,3 | 0,3359 | 60,45 | -0,2019 |
| 70,48 | 0,3366 | 60,41 | -0,252 | 70,32 | 0,3266 | 60,39 | -0,2292 | 70,26 | 0,3359 | 60,48 | -0,202 |
| 70,44 | 0,3365 | 60,44 | -0,2521 | 70,29 | 0,3266 | 60,42 | -0,2292 | 70,23 | 0,3358 | 60,52 | -0,2021 |
| 70,41 | 0,3365 | 60,47 | -0,2521 | 70,25 | 0,3265 | 60,45 | -0,2292 | 70,2 | 0,3358 | 60,55 | -0,2022 |
| 70,38 | 0,3365 | 60,51 | -0,2522 | 70,22 | 0,3265 | 60,49 | -0,2293 | 70,16 | 0,3357 | 60,58 | -0,2023 |
| 70,34 | 0,3364 | 60,54 | -0,2522 | 70,19 | 0,3264 | 60,52 | -0,2293 | 70,13 | 0,3356 | 60,62 | -0,2024 |
| 70,31 | 0,3364 | 60,57 | -0,2522 | 70,16 | 0,3264 | 60,55 | -0,2293 | 70,1 | 0,3356 | 60,65 | -0,2025 |
| 70,28 | 0,3363 | 60,61 | -0,2523 | 70,12 | 0,3264 | 60,59 | -0,2294 | 70,06 | 0,3355 | 60,68 | -0,2025 |
| 70,24 | 0,3363 | 60,64 | -0,2523 | 70,09 | 0,3263 | 60,62 | -0,2294 | 70,03 | 0,3355 | 60,72 | -0,2026 |
| 70,21 | 0,3362 | 60,67 | -0,2523 | 70,05 | 0,3263 | 60,65 | -0,2294 | 70 | 0,3354 | 60,75 | -0,2027 |
| 70,18 | 0,3362 | 60,71 | -0,2524 | 70,02 | 0,3262 | 60,69 | -0,2295 | 69,96 | 0,3354 | 60,78 | -0,2028 |
| 70,14 | 0,3362 | 60,74 | -0,2524 | 69,99 | 0,3262 | 60,72 | -0,2295 | 69,93 | 0,3353 | 60,82 | -0,2029 |
| 70,11 | 0,3361 | 60,77 | -0,2525 | 69,95 | 0,3262 | 60,75 | -0,2296 | 69,9 | 0,3353 | 60,85 | -0,2031 |
| 70,08 | 0,3361 | 60,81 | -0,2525 | 69,92 | 0,3261 | 60,79 | -0,2296 | 69,86 | 0,3352 | 60,88 | -0,2032 |
| 70,04 | 0,336 | 60,84 | -0,2525 | 69,89 | 0,3261 | 60,82 | -0,2296 | 69,83 | 0,3351 | 60,92 | -0,2032 |
| 70,01 | 0,336 | 60,87 | -0,2526 | 69,85 | 0,326 | 60,85 | -0,2297 | 69,8 | 0,3351 | 60,95 | -0,2033 |
| 69,98 | 0,336 | 60,91 | -0,2526 | 69,82 | 0,326 | 60,89 | -0,2297 | 69,76 | 0,335 | 60,98 | -0,2034 |
| 69,94 | 0,3359 | 60,94 | -0,2526 | 69,79 | 0,326 | 60,92 | -0,2297 | 69,73 | 0,335 | 61,02 | -0,2035 |
| 69,91 | 0,3359 | 60,97 | -0,2527 | 69,75 | 0,3259 | 60,95 | -0,2298 | 69,7 | 0,3349 | 61,05 | -0,2036 |
| 69,88 | 0,3358 | 61,01 | -0,2527 | 69,72 | 0,3259 | 60,99 | -0,2298 | 69,66 | 0,3349 | 61,08 | -0,2037 |
| 69,84 | 0,3358 | 61,04 | -0,2528 | 69,69 | 0,3258 | 61,02 | -0,2298 | 69,63 | 0,3348 | 61,11 | -0,2038 |
| 69,81 | 0,3357 | 61,07 | -0,2528 | 69,65 | 0,3258 | 61,06 | -0,2299 | 69,6 | 0,3348 | 61,15 | -0,2039 |
| 69,78 | 0,3357 | 61,1 | -0,2528 | 69,62 | 0,3258 | 61,09 | -0,2299 | 69,56 | 0,3347 | 61,18 | -0,204 |
| 69,74 | 0,3357 | 61,14 | -0,2529 | 69,59 | 0,3257 | 61,12 | -0,2299 | 69,53 | 0,3347 | 61,22 | -0,2041 |
| 69,71 | 0,3356 | 61,17 | -0,2529 | 69,55 | 0,3257 | 61,15 | -0,23 | 69,5 | 0,3346 | 61,25 | -0,2042 |
| 69,67 | 0,3356 | 61,2 | -0,2529 | 69,52 | 0,3256 | 61,19 | -0,23 | 69,46 | 0,3346 | 61,28 | -0,2043 |
| 69,64 | 0,3355 | 61,24 | -0,253 | 69,49 | 0,3256 | 61,22 | -0,2301 | 69,43 | 0,3345 | 61,32 | -0,2044 |
| 69,61 | 0,3355 | 61,27 | -0,253 | 69,45 | 0,3256 | 61,26 | -0,2301 | 69,4 | 0,3344 | 61,35 | -0,2044 |
| 69,58 | 0,3354 | 61,3 | -0,2531 | 69,42 | 0,3255 | 61,29 | -0,2301 | 69,36 | 0,3344 | 61,38 | -0,2046 |
| 69,54 | 0,3354 | 61,34 | -0,2531 | 69,39 | 0,3255 | 61,32 | -0,2302 | 69,33 | 0,3343 | 61,42 | -0,2047 |
| 69,51 | 0,3354 | 61,37 | -0,2531 | 69,35 | 0,3254 | 61,35 | -0,2302 | 69,3 | 0,3343 | 61,45 | -0,2048 |
| 69,48 | 0,3353 | 61,41 | -0,2532 | 69,32 | 0,3254 | 61,39 | -0,2302 | 69,26 | 0,3342 | 61,48 | -0,2049 |
| 69,44 | 0,3353 | 61,44 | -0,2532 | 69,29 | 0,3254 | 61,42 | -0,2303 | 69,23 | 0,3342 | 61,51 | -0,205 |
| 69,41 | 0,3352 | 61,47 | -0,2533 | 69,25 | 0,3253 | 61,46 | -0,2303 | 69,2 | 0,3341 | 61,55 | -0,2051 |
| 69,38 | 0,3352 | 61,5 | -0,2533 | 69,22 | 0,3253 | 61,49 | -0,2303 | 69,16 | 0,3341 | 61,58 | -0,2052 |
| 69,34 | 0,3352 | 61,54 | -0,2533 | 69,19 | 0,3252 | 61,52 | -0,2304 | 69,13 | 0,334 | 61,61 | -0,2053 |
| 69,31 | 0,3351 | 61,57 | -0,2534 | 69,15 | 0,3252 | 61,56 | -0,2304 | 69,1 | 0,3339 | 61,65 | -0,2054 |
| 69,27 | 0,3351 | 61,61 | -0,2534 | 69,12 | 0,3252 | 61,59 | -0,2305 | 69,06 | 0,3339 | 61,68 | -0,2054 |
| 69,24 | 0,335 | 61,64 | -0,2535 | 69,09 | 0,3251 | 61,62 | -0,2305 | 69,03 | 0,3338 | 61,71 | -0,2055 |
| 69,21 | 0,335 | 61,67 | -0,2535 | 69,05 | 0,3251 | 61,66 | -0,2305 | 69 | 0,3338 | 61,75 | -0,2056 |
| 69,17 | 0,3349 | 61,7 | -0,2536 | 69,02 | 0,325 | 61,69 | -0,2306 | 68,96 | 0,3337 | 61,78 | -0,2057 |
| 69,14 | 0,3349 | 61,74 | -0,2536 | 68,99 | 0,325 | 61,72 | -0,2306 | 68,93 | 0,3337 | 61,81 | -0,2058 |
| 69,11 | 0,3349 | 61,77 | -0,2536 | 68,95 | 0,325 | 61,76 | -0,2306 | 68,9 | 0,3336 | 61,85 | -0,2059 |
| 69,08 | 0,3348 | 61,81 | -0,2537 | 68,92 | 0,3249 | 61,79 | -0,2307 | 68,86 | 0,3336 | 61,88 | -0,206 |
| 69,04 | 0,3348 | 61,84 | -0,2537 | 68,89 | 0,3249 | 61,82 | -0,2307 | 68,83 | 0,3335 | 61,91 | -0,2061 |
| 69,01 | 0,3347 | 61,87 | -0,2537 | 68,85 | 0,3248 | 61,85 | -0,2307 | 68,8 | 0,3335 | 61,95 | -0,2061 |
| 68,98 | 0,3347 | 61,9 | -0,2538 | 68,82 | 0,3248 | 61,89 | -0,2308 | 68,76 | 0,3334 | 61,98 | -0,2062 |
| 68,94 | 0,3346 | 61,94 | -0,2538 | 68,79 | 0,3248 | 61,92 | -0,2308 | 68,73 | 0,3334 | 62,01 | -0,2063 |
| 68,91 | 0,3346 | 61,97 | -0,2539 | 68,75 | 0,3247 | 61,95 | -0,2308 | 68,7 | 0,3333 | 62,05 | -0,2064 |
| 68,87 | 0,3346 | 62 | -0,2539 | 68,72 | 0,3247 | 61,99 | -0,2309 | 68,66 | 0,3332 | 62,08 | -0,2065 |
| 68,84 | 0,3345 | 62,04 | -0,2539 | 68,69 | 0,3246 | 62,02 | -0,2309 | 68,63 | 0,3332 | 62,11 | -0,2066 |
| 68,81 | 0,3345 | 62,07 | -0,254 | 68,65 | 0,3246 | 62,05 | -0,2309 | 68,6 | 0,3331 | 62,15 | -0,2067 |
| 68,78 | 0,3344 | 62,1 | -0,254 | 68,62 | 0,3246 | 62,09 | -0,231 | 68,56 | 0,3331 | 62,18 | -0,2068 |
| 68,74 | 0,3344 | 62,14 | -0,254 | 68,59 | 0,3245 | 62,12 | -0,231 | 68,53 | 0,333 | 62,21 | -0,2069 |
| 68,71 | 0,3344 | 62,17 | -0,2541 | 68,55 | 0,3245 | 62,15 | -0,2311 | 68,49 | 0,333 | 62,25 | -0,2069 |
| 68,68 | 0,3343 | 62,2 | -0,2541 | 68,52 | 0,3245 | 62,19 | -0,2311 | 68,46 | 0,3329 | 62,28 | -0,207 |
| 68,64 | 0,3343 | 62,24 | -0,2542 | 68,49 | 0,3244 | 62,22 | -0,2311 | 68,43 | 0,3329 | 62,31 | -0,2071 |
| 68,61 | 0,3342 | 62,27 | -0,2542 | 68,45 | 0,3244 | 62,25 | -0,2312 | 68,4 | 0,3328 | 62,35 | -0,2072 |
| 68,57 | 0,3342 | 62,3 | -0,2542 | 68,42 | 0,3243 | 62,29 | -0,2312 | 68,36 | 0,3328 | 62,38 | -0,2073 |
| 68,54 | 0,3341 | 62,34 | -0,2543 | 68,39 | 0,3243 | 62,32 | -0,2312 | 68,33 | 0,3327 | 62,41 | -0,2074 |
| 68,51 | 0,3341 | 62,37 | -0,2543 | 68,35 | 0,3243 | 62,35 | -0,2313 | 68,29 | 0,3327 | 62,45 | -0,2075 |
| 68,47 | 0,3341 | 62,4 | -0,2544 | 68,32 | 0,3242 | 62,39 | -0,2313 | 68,26 | 0,3326 | 62,48 | -0,2075 |
| 68,44 | 0,334 | 62,44 | -0,2544 | 68,29 | 0,3242 | 62,42 | -0,2313 | 68,23 | 0,3326 | 62,51 | -0,2076 |
| 68,41 | 0,334 | 62,47 | -0,2544 | 68,25 | 0,3241 | 62,45 | -0,2314 | 68,2 | 0,3325 | 62,55 | -0,2077 |
| 68,38 | 0,3339 | 62,5 | -0,2545 | 68,22 | 0,3241 | 62,49 | -0,2314 | 68,16 | 0,3325 | 62,58 | -0,2078 |
| 68,34 | 0,3339 | 62,54 | -0,2545 | 68,19 | 0,3241 | 62,52 | -0,2314 | 68,13 | 0,3324 | 62,61 | -0,2079 |
| 68,31 | 0,3339 | 62,57 | -0,2546 | 68,15 | 0,324 | 62,55 | -0,2315 | 68,1 | 0,3324 | 62,65 | -0,208 |
| 68,27 | 0,3338 | 62,6 | -0,2546 | 68,12 | 0,324 | 62,59 | -0,2315 | 68,06 | 0,3323 | 62,68 | -0,2081 |
| 68,24 | 0,3338 | 62,64 | -0,2546 | 68,09 | 0,3239 | 62,62 | -0,2315 | 68,03 | 0,3322 | 62,71 | -0,2081 |
| 68,21 | 0,3337 | 62,67 | -0,2547 | 68,05 | 0,3239 | 62,65 | -0,2316 | 68 | 0,3322 | 62,75 | -0,2082 |
| 68,17 | 0,3337 | 62,7 | -0,2547 | 68,02 | 0,3239 | 62,69 | -0,2316 | 67,96 | 0,3321 | 62,78 | -0,2083 |
| 68,14 | 0,3336 | 62,74 | -0,2548 | 67,99 | 0,3238 | 62,72 | -0,2317 | 67,93 | 0,3321 | 62,81 | -0,2084 |
| 68,11 | 0,3336 | 62,77 | -0,2548 | 67,95 | 0,3238 | 62,75 | -0,2317 | 67,9 | 0,332 | 62,85 | -0,2085 |
| 68,07 | 0,3336 | 62,8 | -0,2548 | 67,92 | 0,3237 | 62,79 | -0,2317 | 67,86 | 0,332 | 62,88 | -0,2086 |
| 68,04 | 0,3335 | 62,84 | -0,2549 | 67,89 | 0,3237 | 62,82 | -0,2318 | 67,83 | 0,3319 | 62,91 | -0,2087 |
| 68,01 | 0,3335 | 62,87 | -0,2549 | 67,85 | 0,3237 | 62,85 | -0,2318 | 67,8 | 0,3319 | 62,95 | -0,2087 |
| 67,97 | 0,3334 | 62,91 | -0,2549 | 67,82 | 0,3236 | 62,89 | -0,2318 | 67,76 | 0,3318 | 62,98 | -0,2088 |
| 67,94 | 0,3334 | 62,94 | -0,255 | 67,79 | 0,3236 | 62,92 | -0,2319 | 67,73 | 0,3318 | 63,01 | -0,2089 |
| 67,91 | 0,3334 | 62,97 | -0,255 | 67,75 | 0,3236 | 62,95 | -0,2319 | 67,7 | 0,3317 | 63,05 | -0,209 |
| 67,87 | 0,3333 | 63,01 | -0,2551 | 67,72 | 0,3235 | 62,99 | -0,2319 | 67,66 | 0,3317 | 63,08 | -0,2091 |
| 67,84 | 0,3333 | 63,04 | -0,2551 | 67,69 | 0,3235 | 63,02 | -0,232 | 67,63 | 0,3316 | 63,11 | -0,2092 |
| 67,81 | 0,3332 | 63,07 | -0,2551 | 67,65 | 0,3234 | 63,06 | -0,232 | 67,59 | 0,3316 | 63,15 | -0,2093 |
| 67,77 | 0,3332 | 63,11 | -0,2552 | 67,62 | 0,3234 | 63,09 | -0,232 | 67,56 | 0,3315 | 63,18 | -0,2093 |
| 67,74 | 0,3332 | 63,14 | -0,2552 | 67,59 | 0,3234 | 63,12 | -0,2321 | 67,53 | 0,3315 | 63,21 | -0,2094 |
| 67,71 | 0,3331 | 63,17 | -0,2553 | 67,55 | 0,3233 | 63,15 | -0,2321 | 67,49 | 0,3314 | 63,25 | -0,2095 |
| 67,67 | 0,3331 | 63,2 | -0,2553 | 67,52 | 0,3233 | 63,19 | -0,2322 | 67,46 | 0,3314 | 63,28 | -0,2096 |
| 67,64 | 0,333 | 63,24 | -0,2553 | 67,49 | 0,3233 | 63,22 | -0,2322 | 67,43 | 0,3313 | 63,31 | -0,2097 |
| 67,61 | 0,333 | 63,27 | -0,2554 | 67,45 | 0,3232 | 63,25 | -0,2322 | 67,4 | 0,3313 | 63,35 | -0,2098 |
| 67,57 | 0,333 | 63,3 | -0,2554 | 67,42 | 0,3232 | 63,29 | -0,2323 | 67,36 | 0,3312 | 63,38 | -0,2099 |
| 67,54 | 0,3329 | 63,34 | -0,2555 | 67,39 | 0,3231 | 63,32 | -0,2323 | 67,33 | 0,3312 | 63,41 | -0,21 |
| 67,51 | 0,3329 | 63,37 | -0,2555 | 67,35 | 0,3231 | 63,35 | -0,2323 | 67,29 | 0,3311 | 63,45 | -0,2101 |
| 67,47 | 0,3328 | 63,41 | -0,2555 | 67,32 | 0,3231 | 63,39 | -0,2324 | 67,26 | 0,3311 | 63,48 | -0,2102 |
| 67,44 | 0,3328 | 63,44 | -0,2556 | 67,29 | 0,323 | 63,42 | -0,2324 | 67,23 | 0,331 | 63,51 | -0,2103 |
| 67,41 | 0,3328 | 63,47 | -0,2556 | 67,25 | 0,323 | 63,45 | -0,2324 | 67,19 | 0,331 | 63,55 | -0,2104 |
| 67,37 | 0,3327 | 63,51 | -0,2557 | 67,22 | 0,323 | 63,49 | -0,2325 | 67,16 | 0,3309 | 63,58 | -0,2104 |
| 67,34 | 0,3327 | 63,54 | -0,2557 | 67,19 | 0,3229 | 63,52 | -0,2325 | 67,13 | 0,3309 | 63,61 | -0,2105 |
| 67,31 | 0,3326 | 63,57 | -0,2557 | 67,15 | 0,3229 | 63,55 | -0,2325 | 67,09 | 0,3308 | 63,65 | -0,2106 |
| 67,27 | 0,3326 | 63,61 | -0,2558 | 67,12 | 0,3228 | 63,59 | -0,2326 | 67,06 | 0,3308 | 63,68 | -0,2107 |
| 67,24 | 0,3326 | 63,64 | -0,2558 | 67,09 | 0,3228 | 63,62 | -0,2326 | 67,03 | 0,3307 | 63,71 | -0,2108 |
| 67,21 | 0,3325 | 63,67 | -0,2558 | 67,05 | 0,3228 | 63,65 | -0,2326 | 66,99 | 0,3307 | 63,75 | -0,2109 |
| 67,17 | 0,3325 | 63,7 | -0,2559 | 67,02 | 0,3227 | 63,69 | -0,2327 | 66,96 | 0,3306 | 63,78 | -0,211 |
| 67,14 | 0,3324 | 63,74 | -0,2559 | 66,99 | 0,3227 | 63,72 | -0,2327 | 66,93 | 0,3306 | 63,81 | -0,2111 |
| 67,11 | 0,3324 | 63,77 | -0,256 | 66,95 | 0,3227 | 63,75 | -0,2328 | 66,89 | 0,3305 | 63,85 | -0,2111 |
| 67,07 | 0,3324 | 63,81 | -0,256 | 66,92 | 0,3226 | 63,79 | -0,2328 | 66,86 | 0,3305 | 63,88 | -0,2112 |
| 67,04 | 0,3323 | 63,84 | -0,256 | 66,89 | 0,3226 | 63,82 | -0,2328 | 66,83 | 0,3304 | 63,91 | -0,2113 |
| 67,01 | 0,3323 | 63,87 | -0,2561 | 66,85 | 0,3225 | 63,85 | -0,2329 | 66,79 | 0,3304 | 63,95 | -0,2114 |
| 66,97 | 0,3322 | 63,91 | -0,2561 | 66,82 | 0,3225 | 63,89 | -0,2329 | 66,76 | 0,3303 | 63,98 | -0,2115 |
| 66,94 | 0,3322 | 63,94 | -0,2562 | 66,79 | 0,3225 | 63,92 | -0,2329 | 66,73 | 0,3303 | 64,01 | -0,2115 |
| 66,91 | 0,3322 | 63,97 | -0,2562 | 66,75 | 0,3224 | 63,95 | -0,233 | 66,7 | 0,3302 | 64,05 | -0,2116 |
| 66,87 | 0,3321 | 64,01 | -0,2562 | 66,72 | 0,3224 | 63,99 | -0,233 | 66,66 | 0,3302 | 64,08 | -0,2117 |
| 66,84 | 0,3321 | 64,04 | -0,2563 | 66,69 | 0,3224 | 64,02 | -0,233 | 66,63 | 0,3301 | 64,11 | -0,2118 |
| 66,81 | 0,332 | 64,07 | -0,2563 | 66,65 | 0,3223 | 64,06 | -0,2331 | 66,59 | 0,3301 | 64,15 | -0,2119 |
| 66,77 | 0,332 | 64,1 | -0,2564 | 66,62 | 0,3223 | 64,09 | -0,2331 | 66,56 | 0,33 | 64,18 | -0,212 |
| 66,74 | 0,332 | 64,14 | -0,2564 | 66,59 | 0,3222 | 64,12 | -0,2331 | 66,53 | 0,33 | 64,21 | -0,212 |
| 66,71 | 0,3319 | 64,17 | -0,2564 | 66,55 | 0,3222 | 64,15 | -0,2332 | 66,49 | 0,3299 | 64,25 | -0,2121 |
| 66,67 | 0,3319 | 64,21 | -0,2565 | 66,52 | 0,3222 | 64,19 | -0,2332 | 66,46 | 0,3299 | 64,28 | -0,2122 |
| 66,64 | 0,3318 | 64,24 | -0,2565 | 66,49 | 0,3221 | 64,22 | -0,2332 | 66,43 | 0,3298 | 64,31 | -0,2123 |
| 66,61 | 0,3318 | 64,27 | -0,2566 | 66,45 | 0,3221 | 64,25 | -0,2333 | 66,39 | 0,3298 | 64,35 | -0,2124 |
| 66,57 | 0,3318 | 64,31 | -0,2566 | 66,42 | 0,3221 | 64,29 | -0,2333 | 66,36 | 0,3297 | 64,38 | -0,2124 |
| 66,54 | 0,3317 | 64,34 | -0,2566 | 66,39 | 0,322 | 64,32 | -0,2333 | 66,33 | 0,3297 | 64,41 | -0,2125 |
| 66,51 | 0,3317 | 64,37 | -0,2567 | 66,35 | 0,322 | 64,35 | -0,2334 | 66,29 | 0,3296 | 64,45 | -0,2126 |
| 66,47 | 0,3316 | 64,41 | -0,2567 | 66,32 | 0,3219 | 64,39 | -0,2334 | 66,26 | 0,3296 | 64,48 | -0,2127 |
| 66,44 | 0,3316 | 64,44 | -0,2567 | 66,29 | 0,3219 | 64,42 | -0,2335 | 66,23 | 0,3295 | 64,51 | -0,2128 |
| 66,41 | 0,3316 | 64,47 | -0,2568 | 66,25 | 0,3219 | 64,45 | -0,2335 | 66,19 | 0,3295 | 64,55 | -0,2129 |
| 66,37 | 0,3315 | 64,51 | -0,2568 | 66,22 | 0,3218 | 64,49 | -0,2335 | 66,16 | 0,3294 | 64,58 | -0,2129 |
| 66,34 | 0,3315 | 64,54 | -0,2569 | 66,19 | 0,3218 | 64,52 | -0,2336 | 66,13 | 0,3294 | 64,61 | -0,213 |
| 66,31 | 0,3314 | 64,57 | -0,2569 | 66,15 | 0,3218 | 64,56 | -0,2336 | 66,09 | 0,3293 | 64,65 | -0,2131 |
| 66,27 | 0,3314 | 64,61 | -0,2569 | 66,12 | 0,3217 | 64,59 | -0,2336 | 66,06 | 0,3293 | 64,68 | -0,2132 |
| 66,24 | 0,3314 | 64,64 | -0,257 | 66,09 | 0,3217 | 64,62 | -0,2337 | 66,03 | 0,3292 | 64,71 | -0,2133 |
| 66,21 | 0,3313 | 64,67 | -0,257 | 66,05 | 0,3217 | 64,65 | -0,2337 | 65,99 | 0,3292 | 64,75 | -0,2133 |
| 66,17 | 0,3313 | 64,71 | -0,2571 | 66,02 | 0,3216 | 64,69 | -0,2337 | 65,96 | 0,3291 | 64,78 | -0,2134 |
| 66,14 | 0,3312 | 64,74 | -0,2571 | 65,99 | 0,3216 | 64,72 | -0,2338 | 65,93 | 0,3291 | 64,81 | -0,2135 |
| 66,11 | 0,3312 | 64,77 | -0,2571 | 65,95 | 0,3215 | 64,75 | -0,2338 | 65,89 | 0,329 | 64,85 | -0,2136 |
| 66,07 | 0,3312 | 64,8 | -0,2572 | 65,92 | 0,3215 | 64,79 | -0,2338 | 65,86 | 0,329 | 64,88 | -0,2137 |
| 66,04 | 0,3311 | 64,84 | -0,2572 | 65,89 | 0,3215 | 64,82 | -0,2339 | 65,83 | 0,3289 | 64,91 | -0,2137 |
| 66,01 | 0,3311 | 64,87 | -0,2573 | 65,85 | 0,3214 | 64,86 | -0,2339 | 65,79 | 0,3289 | 64,95 | -0,2138 |
| 65,97 | 0,331 | 64,91 | -0,2573 | 65,82 | 0,3214 | 64,89 | -0,234 | 65,76 | 0,3288 | 64,98 | -0,2139 |
| 65,94 | 0,331 | 64,94 | -0,2573 | 65,79 | 0,3214 | 64,92 | -0,234 | 65,73 | 0,3287 | 65,01 | -0,214 |
| 65,91 | 0,331 | 64,97 | -0,2574 | 65,75 | 0,3213 | 64,95 | -0,234 | 65,69 | 0,3287 | 65,05 | -0,2141 |
| 65,87 | 0,3309 | 65,01 | -0,2574 | 65,72 | 0,3213 | 64,99 | -0,2341 | 65,66 | 0,3286 | 65,08 | -0,2141 |
| 65,84 | 0,3309 | 65,04 | -0,2575 | 65,69 | 0,3212 | 65,02 | -0,2341 | 65,63 | 0,3286 | 65,11 | -0,2142 |
| 65,81 | 0,3308 | 65,07 | -0,2575 | 65,65 | 0,3212 | 65,05 | -0,2341 | 65,59 | 0,3285 | 65,15 | -0,2143 |
| 65,77 | 0,3308 | 65,1 | -0,2575 | 65,62 | 0,3212 | 65,09 | -0,2342 | 65,56 | 0,3285 | 65,18 | -0,2144 |
| 65,74 | 0,3308 | 65,14 | -0,2576 | 65,59 | 0,3211 | 65,12 | -0,2342 | 65,53 | 0,3284 | 65,21 | -0,2145 |
| 65,71 | 0,3307 | 65,17 | -0,2576 | 65,55 | 0,3211 | 65,15 | -0,2342 | 65,49 | 0,3284 | 65,25 | -0,2145 |
| 65,67 | 0,3307 | 65,21 | -0,2577 | 65,52 | 0,3211 | 65,19 | -0,2343 | 65,46 | 0,3283 | 65,28 | -0,2146 |
| 65,64 | 0,3306 | 65,24 | -0,2577 | 65,49 | 0,321 | 65,22 | -0,2343 | 65,43 | 0,3283 | 65,31 | -0,2147 |
| 65,61 | 0,3306 | 65,27 | -0,2577 | 65,45 | 0,321 | 65,25 | -0,2343 | 65,39 | 0,3282 | 65,35 | -0,2148 |
| 65,57 | 0,3306 | 65,31 | -0,2578 | 65,42 | 0,3209 | 65,29 | -0,2344 | 65,36 | 0,3282 | 65,38 | -0,2149 |
| 65,54 | 0,3305 | 65,34 | -0,2578 | 65,39 | 0,3209 | 65,32 | -0,2344 | 65,33 | 0,3281 | 65,41 | -0,2149 |
| 65,51 | 0,3305 | 65,37 | -0,2579 | 65,35 | 0,3209 | 65,36 | -0,2344 | 65,29 | 0,3281 | 65,45 | -0,215 |
| 65,47 | 0,3304 | 65,4 | -0,2579 | 65,32 | 0,3208 | 65,39 | -0,2345 | 65,26 | 0,328 | 65,48 | -0,2151 |
| 65,44 | 0,3304 | 65,44 | -0,2579 | 65,29 | 0,3208 | 65,42 | -0,2345 | 65,23 | 0,328 | 65,51 | -0,2152 |
| 65,41 | 0,3304 | 65,47 | -0,258 | 65,25 | 0,3208 | 65,45 | -0,2346 | 65,19 | 0,3279 | 65,55 | -0,2153 |
| 65,37 | 0,3303 | 65,51 | -0,258 | 65,22 | 0,3207 | 65,49 | -0,2346 | 65,16 | 0,3279 | 65,58 | -0,2153 |
| 65,34 | 0,3303 | 65,54 | -0,2581 | 65,19 | 0,3207 | 65,52 | -0,2346 | 65,13 | 0,3278 | 65,61 | -0,2154 |
| 65,31 | 0,3302 | 65,57 | -0,2581 | 65,15 | 0,3207 | 65,55 | -0,2347 | 65,09 | 0,3278 | 65,65 | -0,2155 |
| 65,27 | 0,3302 | 65,61 | -0,2581 | 65,12 | 0,3206 | 65,59 | -0,2347 | 65,06 | 0,3277 | 65,68 | -0,2156 |
| 65,24 | 0,3302 | 65,64 | -0,2582 | 65,09 | 0,3206 | 65,62 | -0,2347 | 65,03 | 0,3277 | 65,71 | -0,2156 |
| 65,21 | 0,3301 | 65,67 | -0,2582 | 65,05 | 0,3205 | 65,66 | -0,2348 | 64,99 | 0,3276 | 65,75 | -0,2157 |
| 65,17 | 0,3301 | 65,71 | -0,2583 | 65,02 | 0,3205 | 65,69 | -0,2348 | 64,96 | 0,3276 | 65,78 | -0,2158 |
| 65,14 | 0,33 | 65,74 | -0,2583 | 64,99 | 0,3205 | 65,72 | -0,2349 | 64,93 | 0,3275 | 65,81 | -0,2159 |
| 65,11 | 0,33 | 65,77 | -0,2583 | 64,95 | 0,3204 | 65,75 | -0,2349 | 64,89 | 0,3275 | 65,85 | -0,216 |
| 65,07 | 0,3299 | 65,8 | -0,2584 | 64,92 | 0,3204 | 65,79 | -0,2349 | 64,86 | 0,3274 | 65,88 | -0,216 |
| 65,04 | 0,3299 | 65,84 | -0,2584 | 64,89 | 0,3204 | 65,82 | -0,235 | 64,83 | 0,3274 | 65,91 | -0,2161 |
| 65,01 | 0,3299 | 65,87 | -0,2584 | 64,85 | 0,3203 | 65,86 | -0,235 | 64,79 | 0,3273 | 65,95 | -0,2162 |
| 64,97 | 0,3298 | 65,91 | -0,2585 | 64,82 | 0,3203 | 65,89 | -0,235 | 64,76 | 0,3273 | 65,98 | -0,2162 |
| 64,94 | 0,3298 | 65,94 | -0,2585 | 64,79 | 0,3202 | 65,92 | -0,2351 | 64,73 | 0,3272 | 66,01 | -0,2163 |
| 64,91 | 0,3297 | 65,97 | -0,2586 | 64,75 | 0,3202 | 65,96 | -0,2351 | 64,69 | 0,3272 | 66,05 | -0,2164 |
| 64,87 | 0,3297 | 66,01 | -0,2586 | 64,72 | 0,3202 | 65,99 | -0,2351 | 64,66 | 0,3271 | 66,08 | -0,2165 |
| 64,84 | 0,3296 | 66,04 | -0,2586 | 64,69 | 0,3201 | 66,02 | -0,2352 | 64,63 | 0,327 | 66,11 | -0,2166 |
| 64,81 | 0,3296 | 66,07 | -0,2587 | 64,65 | 0,3201 | 66,05 | -0,2352 | 64,59 | 0,327 | 66,15 | -0,2166 |
| 64,77 | 0,3296 | 66,11 | -0,2587 | 64,62 | 0,32 | 66,09 | -0,2353 | 64,56 | 0,3269 | 66,18 | -0,2167 |
| 64,74 | 0,3295 | 66,14 | -0,2588 | 64,59 | 0,32 | 66,12 | -0,2353 | 64,53 | 0,3269 | 66,21 | -0,2168 |
| 64,71 | 0,3295 | 66,17 | -0,2588 | 64,55 | 0,32 | 66,15 | -0,2353 | 64,49 | 0,3268 | 66,25 | -0,2169 |
| 64,67 | 0,3294 | 66,21 | -0,2588 | 64,52 | 0,3199 | 66,19 | -0,2354 | 64,46 | 0,3268 | 66,28 | -0,2169 |
| 64,64 | 0,3294 | 66,24 | -0,2589 | 64,48 | 0,3199 | 66,22 | -0,2354 | 64,43 | 0,3267 | 66,31 | -0,217 |
| 64,6 | 0,3294 | 66,27 | -0,2589 | 64,45 | 0,3199 | 66,26 | -0,2354 | 64,39 | 0,3267 | 66,35 | -0,2171 |
| 64,57 | 0,3293 | 66,31 | -0,259 | 64,42 | 0,3198 | 66,29 | -0,2355 | 64,36 | 0,3266 | 66,38 | -0,2172 |
| 64,54 | 0,3293 | 66,34 | -0,259 | 64,38 | 0,3198 | 66,32 | -0,2355 | 64,33 | 0,3266 | 66,41 | -0,2172 |
| 64,5 | 0,3292 | 66,37 | -0,259 | 64,35 | 0,3197 | 66,35 | -0,2355 | 64,29 | 0,3265 | 66,45 | -0,2173 |
| 64,47 | 0,3292 | 66,41 | -0,2591 | 64,32 | 0,3197 | 66,39 | -0,2356 | 64,26 | 0,3265 | 66,48 | -0,2174 |
| 64,44 | 0,3292 | 66,44 | -0,2591 | 64,28 | 0,3197 | 66,42 | -0,2356 | 64,23 | 0,3264 | 66,51 | -0,2175 |
| 64,41 | 0,3291 | 66,47 | -0,2591 | 64,25 | 0,3196 | 66,46 | -0,2356 | 64,19 | 0,3263 | 66,55 | -0,2175 |
| 64,37 | 0,3291 | 66,51 | -0,2592 | 64,22 | 0,3196 | 66,49 | -0,2357 | 64,16 | 0,3263 | 66,58 | -0,2176 |
| 64,34 | 0,329 | 66,54 | -0,2592 | 64,18 | 0,3196 | 66,52 | -0,2357 | 64,13 | 0,3262 | 66,61 | -0,2177 |
| 64,31 | 0,329 | 66,57 | -0,2593 | 64,15 | 0,3195 | 66,55 | -0,2357 | 64,09 | 0,3262 | 66,65 | -0,2177 |
| 64,27 | 0,3289 | 66,61 | -0,2593 | 64,12 | 0,3195 | 66,59 | -0,2358 | 64,06 | 0,3262 | 66,68 | -0,2178 |
| 64,24 | 0,3289 | 66,64 | -0,2594 | 64,08 | 0,3195 | 66,62 | -0,2358 | 64,03 | 0,3261 | 66,71 | -0,2179 |
| 64,2 | 0,3289 | 66,67 | -0,2594 | 64,05 | 0,3194 | 66,66 | -0,2359 | 63,99 | 0,326 | 66,75 | -0,218 |
| 64,17 | 0,3288 | 66,71 | -0,2594 | 64,02 | 0,3194 | 66,69 | -0,2359 | 63,96 | 0,326 | 66,78 | -0,218 |
| 64,14 | 0,3288 | 66,74 | -0,2595 | 63,98 | 0,3193 | 66,72 | -0,2359 | 63,92 | 0,3259 | 66,81 | -0,2181 |
| 64,11 | 0,3287 | 66,77 | -0,2595 | 63,95 | 0,3193 | 66,76 | -0,236 | 63,89 | 0,3259 | 66,85 | -0,2182 |
| 64,07 | 0,3287 | 66,81 | -0,2595 | 63,92 | 0,3193 | 66,79 | -0,236 | 63,86 | 0,3258 | 66,88 | -0,2183 |
| 64,04 | 0,3287 | 66,84 | -0,2596 | 63,88 | 0,3192 | 66,82 | -0,236 | 63,83 | 0,3258 | 66,92 | -0,2183 |
| 64 | 0,3286 | 66,87 | -0,2596 | 63,85 | 0,3192 | 66,86 | -0,2361 | 63,79 | 0,3257 | 66,95 | -0,2184 |
| 63,97 | 0,3285 | 66,91 | -0,2597 | 63,82 | 0,3192 | 66,89 | -0,2361 | 63,76 | 0,3257 | 66,98 | -0,2185 |
| 63,94 | 0,3285 | 66,94 | -0,2597 | 63,78 | 0,3191 | 66,92 | -0,2362 | 63,73 | 0,3256 | 67,01 | -0,2185 |
| 63,91 | 0,3285 | 66,97 | -0,2597 | 63,75 | 0,3191 | 66,95 | -0,2362 | 63,69 | 0,3256 | 67,05 | -0,2186 |
| 63,87 | 0,3284 | 67,01 | -0,2598 | 63,72 | 0,319 | 66,99 | -0,2362 | 63,66 | 0,3255 | 67,08 | -0,2187 |
| 63,84 | 0,3284 | 67,04 | -0,2598 | 63,68 | 0,319 | 67,02 | -0,2363 | 63,62 | 0,3255 | 67,11 | -0,2188 |
| 63,81 | 0,3283 | 67,07 | -0,2599 | 63,65 | 0,319 | 67,06 | -0,2363 | 63,59 | 0,3254 | 67,15 | -0,2188 |
| 63,77 | 0,3283 | 67,11 | -0,2599 | 63,62 | 0,3189 | 67,09 | -0,2363 | 63,56 | 0,3254 | 67,18 | -0,2189 |
| 63,74 | 0,3283 | 67,14 | -0,2599 | 63,58 | 0,3189 | 67,12 | -0,2364 | 63,53 | 0,3253 | 67,22 | -0,219 |
| 63,71 | 0,3282 | 67,17 | -0,26 | 63,55 | 0,3189 | 67,16 | -0,2364 | 63,49 | 0,3253 | 67,25 | -0,219 |
| 63,67 | 0,3282 | 67,21 | -0,26 | 63,52 | 0,3188 | 67,19 | -0,2364 | 63,46 | 0,3252 | 67,28 | -0,2191 |
| 63,64 | 0,3281 | 67,24 | -0,2601 | 63,48 | 0,3188 | 67,22 | -0,2365 | 63,43 | 0,3251 | 67,32 | -0,2192 |
| 63,6 | 0,3281 | 67,27 | -0,2601 | 63,45 | 0,3187 | 67,26 | -0,2365 | 63,39 | 0,3251 | 67,35 | -0,2192 |
| 63,57 | 0,3281 | 67,31 | -0,2601 | 63,42 | 0,3187 | 67,29 | -0,2365 | 63,36 | 0,325 | 67,38 | -0,2193 |
| 63,54 | 0,328 | 67,34 | -0,2602 | 63,38 | 0,3187 | 67,32 | -0,2366 | 63,32 | 0,325 | 67,41 | -0,2194 |
| 63,5 | 0,328 | 67,37 | -0,2602 | 63,35 | 0,3186 | 67,36 | -0,2366 | 63,29 | 0,3249 | 67,45 | -0,2194 |
| 63,47 | 0,3279 | 67,41 | -0,2603 | 63,32 | 0,3186 | 67,39 | -0,2367 | 63,26 | 0,3249 | 67,48 | -0,2195 |
| 63,44 | 0,3279 | 67,44 | -0,2603 | 63,28 | 0,3186 | 67,42 | -0,2367 | 63,22 | 0,3248 | 67,52 | -0,2196 |
| 63,4 | 0,3279 | 67,47 | -0,2603 | 63,25 | 0,3185 | 67,46 | -0,2367 | 63,19 | 0,3248 | 67,55 | -0,2197 |
| 63,37 | 0,3278 | 67,51 | -0,2604 | 63,22 | 0,3185 | 67,49 | -0,2368 | 63,16 | 0,3247 | 67,58 | -0,2197 |
| 63,34 | 0,3278 | 67,54 | -0,2604 | 63,18 | 0,3184 | 67,52 | -0,2368 | 63,12 | 0,3247 | 67,62 | -0,2198 |
| 63,3 | 0,3277 | 67,57 | -0,2605 | 63,15 | 0,3184 | 67,55 | -0,2368 | 63,09 | 0,3246 | 67,65 | -0,2199 |
| 63,27 | 0,3277 | 67,61 | -0,2605 | 63,12 | 0,3184 | 67,59 | -0,2369 | 63,06 | 0,3246 | 67,68 | -0,2199 |
| 63,24 | 0,3276 | 67,64 | -0,2605 | 63,08 | 0,3183 | 67,62 | -0,2369 | 63,02 | 0,3245 | 67,72 | -0,22 |
| 63,2 | 0,3276 | 67,67 | -0,2606 | 63,05 | 0,3183 | 67,66 | -0,2369 | 62,99 | 0,3245 | 67,75 | -0,2201 |
| 63,17 | 0,3276 | 67,71 | -0,2606 | 63,02 | 0,3183 | 67,69 | -0,237 | 62,96 | 0,3244 | 67,78 | -0,2201 |
| 63,14 | 0,3275 | 67,74 | -0,2606 | 62,98 | 0,3182 | 67,72 | -0,237 | 62,92 | 0,3243 | 67,82 | -0,2202 |
| 63,1 | 0,3275 | 67,77 | -0,2607 | 62,95 | 0,3182 | 67,76 | -0,237 | 62,89 | 0,3243 | 67,85 | -0,2203 |
| 63,07 | 0,3274 | 67,81 | -0,2607 | 62,92 | 0,3181 | 67,79 | -0,2371 | 62,86 | 0,3242 | 67,88 | -0,2203 |
| 63,04 | 0,3274 | 67,84 | -0,2608 | 62,88 | 0,3181 | 67,82 | -0,2371 | 62,82 | 0,3242 | 67,91 | -0,2204 |
| 63 | 0,3273 | 67,87 | -0,2608 | 62,85 | 0,3181 | 67,86 | -0,2371 | 62,79 | 0,3241 | 67,95 | -0,2205 |
| 62,97 | 0,3273 | 67,91 | -0,2608 | 62,82 | 0,318 | 67,89 | -0,2372 | 62,76 | 0,3241 | 67,98 | -0,2205 |
| 62,94 | 0,3273 | 67,94 | -0,2609 | 62,78 | 0,318 | 67,92 | -0,2372 | 62,72 | 0,324 | 68,02 | -0,2206 |
| 62,9 | 0,3272 | 67,97 | -0,2609 | 62,75 | 0,318 | 67,96 | -0,2373 | 62,69 | 0,324 | 68,05 | -0,2207 |
| 62,87 | 0,3272 | 68,01 | -0,261 | 62,72 | 0,3179 | 67,99 | -0,2373 | 62,66 | 0,3239 | 68,08 | -0,2207 |
| 62,84 | 0,3271 | 68,04 | -0,261 | 62,68 | 0,3179 | 68,02 | -0,2373 | 62,62 | 0,3239 | 68,12 | -0,2208 |
| 62,8 | 0,3271 | 68,07 | -0,261 | 62,65 | 0,3178 | 68,06 | -0,2374 | 62,59 | 0,3238 | 68,15 | -0,2209 |
| 62,77 | 0,3271 | 68,11 | -0,2611 | 62,62 | 0,3178 | 68,09 | -0,2374 | 62,56 | 0,3238 | 68,18 | -0,2209 |
| 62,74 | 0,327 | 68,14 | -0,2611 | 62,58 | 0,3178 | 68,12 | -0,2374 | 62,52 | 0,3237 | 68,22 | -0,221 |
| 62,7 | 0,327 | 68,17 | -0,2611 | 62,55 | 0,3177 | 68,16 | -0,2375 | 62,49 | 0,3237 | 68,25 | -0,2211 |
| 62,67 | 0,3269 | 68,21 | -0,2612 | 62,52 | 0,3177 | 68,19 | -0,2375 | 62,46 | 0,3236 | 68,28 | -0,2211 |
| 62,64 | 0,3269 | 68,24 | -0,2612 | 62,48 | 0,3177 | 68,22 | -0,2375 | 62,42 | 0,3235 | 68,32 | -0,2212 |
| 62,6 | 0,3269 | 68,27 | -0,2613 | 62,45 | 0,3176 | 68,26 | -0,2376 | 62,39 | 0,3235 | 68,35 | -0,2213 |
| 62,57 | 0,3268 | 68,31 | -0,2613 | 62,42 | 0,3176 | 68,29 | -0,2376 | 62,36 | 0,3234 | 68,38 | -0,2213 |
| 62,54 | 0,3268 | 68,34 | -0,2613 | 62,38 | 0,3175 | 68,32 | -0,2376 | 62,32 | 0,3234 | 68,42 | -0,2214 |
| 62,5 | 0,3267 | 68,37 | -0,2614 | 62,35 | 0,3175 | 68,36 | -0,2377 | 62,29 | 0,3233 | 68,45 | -0,2215 |
| 62,47 | 0,3267 | 68,41 | -0,2614 | 62,32 | 0,3175 | 68,39 | -0,2377 | 62,26 | 0,3233 | 68,48 | -0,2215 |
| 62,44 | 0,3266 | 68,44 | -0,2615 | 62,28 | 0,3174 | 68,42 | -0,2378 | 62,22 | 0,3232 | 68,51 | -0,2216 |
| 62,4 | 0,3266 | 68,47 | -0,2615 | 62,25 | 0,3174 | 68,46 | -0,2378 | 62,19 | 0,3232 | 68,55 | -0,2217 |
| 62,37 | 0,3266 | 68,51 | -0,2616 | 62,22 | 0,3174 | 68,49 | -0,2378 | 62,16 | 0,3231 | 68,58 | -0,2217 |
| 62,34 | 0,3265 | 68,54 | -0,2616 | 62,18 | 0,3173 | 68,52 | -0,2379 | 62,12 | 0,3231 | 68,62 | -0,2218 |
| 62,3 | 0,3265 | 68,57 | -0,2616 | 62,15 | 0,3173 | 68,56 | -0,2379 | 62,09 | 0,323 | 68,65 | -0,2219 |
| 62,27 | 0,3264 | 68,61 | -0,2617 | 62,12 | 0,3172 | 68,59 | -0,2379 | 62,06 | 0,323 | 68,68 | -0,2219 |
| 62,24 | 0,3264 | 68,64 | -0,2617 | 62,08 | 0,3172 | 68,62 | -0,238 | 62,02 | 0,3229 | 68,72 | -0,222 |
| 62,2 | 0,3264 | 68,67 | -0,2618 | 62,05 | 0,3172 | 68,66 | -0,238 | 61,99 | 0,3228 | 68,75 | -0,2221 |
| 62,17 | 0,3263 | 68,71 | -0,2618 | 62,02 | 0,3171 | 68,69 | -0,238 | 61,96 | 0,3228 | 68,78 | -0,2221 |
| 62,14 | 0,3263 | 68,74 | -0,2618 | 61,98 | 0,3171 | 68,72 | -0,2381 | 61,92 | 0,3227 | 68,81 | -0,2222 |
| 62,1 | 0,3262 | 68,77 | -0,2619 | 61,95 | 0,3171 | 68,76 | -0,2381 | 61,89 | 0,3227 | 68,85 | -0,2223 |
| 62,07 | 0,3262 | 68,81 | -0,2619 | 61,92 | 0,317 | 68,79 | -0,2382 | 61,86 | 0,3226 | 68,88 | -0,2223 |
| 62,04 | 0,3262 | 68,84 | -0,2619 | 61,88 | 0,317 | 68,82 | -0,2382 | 61,82 | 0,3226 | 68,92 | -0,2224 |
| 62 | 0,3261 | 68,87 | -0,262 | 61,85 | 0,3169 | 68,86 | -0,2382 | 61,79 | 0,3225 | 68,95 | -0,2225 |
| 61,97 | 0,3261 | 68,91 | -0,262 | 61,82 | 0,3169 | 68,89 | -0,2383 | 61,76 | 0,3225 | 68,98 | -0,2225 |
| 61,94 | 0,326 | 68,94 | -0,2621 | 61,78 | 0,3169 | 68,92 | -0,2383 | 61,72 | 0,3224 | 69,02 | -0,2226 |
| 61,9 | 0,326 | 68,97 | -0,2621 | 61,75 | 0,3168 | 68,96 | -0,2383 | 61,69 | 0,3224 | 69,05 | -0,2227 |
| 61,87 | 0,3259 | 69,01 | -0,2622 | 61,72 | 0,3168 | 68,99 | -0,2384 | 61,66 | 0,3223 | 69,08 | -0,2227 |
| 61,84 | 0,3259 | 69,04 | -0,2622 | 61,68 | 0,3168 | 69,02 | -0,2384 | 61,62 | 0,3222 | 69,12 | -0,2228 |
| 61,8 | 0,3259 | 69,07 | -0,2622 | 61,65 | 0,3167 | 69,06 | -0,2384 | 61,59 | 0,3222 | 69,15 | -0,2229 |
| 61,77 | 0,3258 | 69,11 | -0,2623 | 61,62 | 0,3167 | 69,09 | -0,2385 | 61,56 | 0,3221 | 69,18 | -0,2229 |
| 61,74 | 0,3258 | 69,14 | -0,2623 | 61,58 | 0,3167 | 69,12 | -0,2385 | 61,52 | 0,3221 | 69,22 | -0,223 |
| 61,7 | 0,3257 | 69,17 | -0,2624 | 61,55 | 0,3166 | 69,16 | -0,2386 | 61,49 | 0,322 | 69,25 | -0,2231 |
| 61,67 | 0,3257 | 69,21 | -0,2624 | 61,52 | 0,3166 | 69,19 | -0,2386 | 61,46 | 0,322 | 69,28 | -0,2231 |
| 61,64 | 0,3257 | 69,24 | -0,2624 | 61,48 | 0,3165 | 69,22 | -0,2386 | 61,42 | 0,3219 | 69,32 | -0,2232 |
| 61,6 | 0,3256 | 69,27 | -0,2625 | 61,45 | 0,3165 | 69,26 | -0,2387 | 61,39 | 0,3219 | 69,35 | -0,2233 |
| 61,57 | 0,3256 | 69,31 | -0,2625 | 61,42 | 0,3165 | 69,29 | -0,2387 | 61,36 | 0,3218 | 69,38 | -0,2233 |
| 61,54 | 0,3255 | 69,34 | -0,2626 | 61,38 | 0,3164 | 69,32 | -0,2387 | 61,32 | 0,3218 | 69,42 | -0,2234 |
| 61,5 | 0,3255 | 69,37 | -0,2626 | 61,35 | 0,3164 | 69,36 | -0,2388 | 61,29 | 0,3217 | 69,45 | -0,2234 |
| 61,47 | 0,3255 | 69,41 | -0,2626 | 61,32 | 0,3164 | 69,39 | -0,2388 | 61,26 | 0,3217 | 69,48 | -0,2235 |
| 61,44 | 0,3254 | 69,44 | -0,2627 | 61,28 | 0,3163 | 69,42 | -0,2388 | 61,22 | 0,3216 | 69,52 | -0,2236 |
| 61,4 | 0,3254 | 69,47 | -0,2627 | 61,25 | 0,3163 | 69,46 | -0,2389 | 61,19 | 0,3215 | 69,55 | -0,2236 |
| 61,37 | 0,3253 | 69,51 | -0,2628 | 61,22 | 0,3162 | 69,49 | -0,2389 | 61,16 | 0,3215 | 69,58 | -0,2237 |
| 61,34 | 0,3253 | 69,54 | -0,2628 | 61,18 | 0,3162 | 69,52 | -0,239 | 61,12 | 0,3214 | 69,62 | -0,2238 |
| 61,3 | 0,3252 | 69,57 | -0,2629 | 61,15 | 0,3162 | 69,56 | -0,239 | 61,09 | 0,3214 | 69,65 | -0,2238 |
| 61,27 | 0,3252 | 69,61 | -0,2629 | 61,12 | 0,3161 | 69,59 | -0,239 | 61,06 | 0,3213 | 69,68 | -0,2239 |
| 61,24 | 0,3252 | 69,64 | -0,2629 | 61,08 | 0,3161 | 69,62 | -0,2391 | 61,02 | 0,3213 | 69,72 | -0,224 |
| 61,2 | 0,3251 | 69,67 | -0,263 | 61,05 | 0,3161 | 69,65 | -0,2391 | 60,99 | 0,3212 | 69,75 | -0,224 |
| 61,17 | 0,3251 | 69,71 | -0,263 | 61,02 | 0,316 | 69,69 | -0,2392 | 60,96 | 0,3212 | 69,78 | -0,2241 |
| 61,14 | 0,325 | 69,74 | -0,2631 | 60,98 | 0,316 | 69,72 | -0,2392 | 60,92 | 0,3211 | 69,82 | -0,2242 |
| 61,1 | 0,325 | 69,77 | -0,2631 | 60,95 | 0,316 | 69,76 | -0,2392 | 60,89 | 0,3211 | 69,85 | -0,2242 |
| 61,07 | 0,325 | 69,81 | -0,2631 | 60,92 | 0,3159 | 69,79 | -0,2393 | 60,86 | 0,321 | 69,88 | -0,2243 |
| 61,04 | 0,3249 | 69,84 | -0,2632 | 60,88 | 0,3159 | 69,82 | -0,2393 | 60,82 | 0,3209 | 69,92 | -0,2244 |
| 61 | 0,3249 | 69,87 | -0,2632 | 60,85 | 0,3158 | 69,86 | -0,2393 | 60,79 | 0,3209 | 69,95 | -0,2244 |
| 60,97 | 0,3248 | 69,91 | -0,2633 | 60,82 | 0,3158 | 69,89 | -0,2394 | 60,76 | 0,3208 | 69,98 | -0,2245 |
| 60,94 | 0,3248 | 69,94 | -0,2633 | 60,78 | 0,3158 | 69,92 | -0,2394 | 60,72 | 0,3208 | 70,02 | -0,2245 |
| 60,9 | 0,3248 | 69,97 | -0,2634 | 60,75 | 0,3157 | 69,95 | -0,2395 | 60,69 | 0,3207 | 70,05 | -0,2246 |
| 60,87 | 0,3247 | 70,01 | -0,2634 | 60,71 | 0,3157 | 69,99 | -0,2395 | 60,66 | 0,3207 | 70,08 | -0,2247 |
| 60,84 | 0,3247 | 70,04 | -0,2634 | 60,68 | 0,3157 | 70,02 | -0,2395 | 60,62 | 0,3206 | 70,12 | -0,2247 |
| 60,8 | 0,3246 | 70,07 | -0,2635 | 60,65 | 0,3156 | 70,06 | -0,2396 | 60,59 | 0,3206 | 70,15 | -0,2248 |
| 60,77 | 0,3246 | 70,11 | -0,2635 | 60,62 | 0,3156 | 70,09 | -0,2396 | 60,56 | 0,3205 | 70,18 | -0,2249 |
| 60,74 | 0,3246 | 70,14 | -0,2636 | 60,58 | 0,3155 | 70,12 | -0,2396 | 60,52 | 0,3205 | 70,22 | -0,2249 |
| 60,7 | 0,3245 | 70,17 | -0,2636 | 60,55 | 0,3155 | 70,15 | -0,2397 | 60,49 | 0,3204 | 70,25 | -0,225 |
| 60,67 | 0,3245 | 70,21 | -0,2637 | 60,52 | 0,3155 | 70,19 | -0,2397 | 60,46 | 0,3203 | 70,28 | -0,2251 |
| 60,64 | 0,3244 | 70,24 | -0,2637 | 60,48 | 0,3154 | 70,22 | -0,2398 | 60,42 | 0,3203 | 70,32 | -0,2251 |
| 60,6 | 0,3244 | 70,27 | -0,2637 | 60,45 | 0,3154 | 70,26 | -0,2398 | 60,39 | 0,3202 | 70,35 | -0,2252 |
| 60,57 | 0,3244 | 70,31 | -0,2638 | 60,42 | 0,3154 | 70,29 | -0,2398 | 60,36 | 0,3202 | 70,38 | -0,2253 |
| 60,54 | 0,3243 | 70,34 | -0,2638 | 60,38 | 0,3153 | 70,32 | -0,2399 | 60,32 | 0,3201 | 70,42 | -0,2253 |
| 60,5 | 0,3243 | 70,37 | -0,2639 | 60,35 | 0,3153 | 70,36 | -0,2399 | 60,29 | 0,3201 | 70,45 | -0,2254 |
| 60,47 | 0,3242 | 70,41 | -0,2639 | 60,32 | 0,3152 | 70,39 | -0,2399 | 60,26 | 0,32 | 70,48 | -0,2255 |
| 60,44 | 0,3242 | 70,44 | -0,2639 | 60,28 | 0,3152 | 70,42 | -0,24 | 60,22 | 0,3199 | 70,52 | -0,2255 |
| 60,4 | 0,3241 | 70,47 | -0,264 | 60,25 | 0,3152 | 70,45 | -0,24 | 60,19 | 0,3199 | 70,55 | -0,2256 |
| 60,37 | 0,3241 | 70,51 | -0,264 | 60,22 | 0,3151 | 70,49 | -0,2401 | 60,16 | 0,3198 | 70,58 | -0,2256 |
| 60,34 | 0,3241 | 70,54 | -0,2641 | 60,18 | 0,3151 | 70,52 | -0,2401 | 60,12 | 0,3198 | 70,62 | -0,2257 |
| 60,3 | 0,324 | 70,57 | -0,2641 | 60,15 | 0,3151 | 70,56 | -0,2401 | 60,09 | 0,3197 | 70,65 | -0,2258 |
| 60,27 | 0,324 | 70,61 | -0,2642 | 60,12 | 0,315 | 70,59 | -0,2402 | 60,06 | 0,3197 | 70,68 | -0,2258 |
| 60,24 | 0,3239 | 70,64 | -0,2642 | 60,08 | 0,315 | 70,62 | -0,2402 | 60,02 | 0,3196 | 70,72 | -0,2259 |
| 60,2 | 0,3239 | 70,67 | -0,2642 | 60,05 | 0,315 | 70,65 | -0,2403 | 59,99 | 0,3196 | 70,75 | -0,226 |
| 60,17 | 0,3239 | 70,71 | -0,2643 | 60,02 | 0,3149 | 70,69 | -0,2403 | 59,96 | 0,3195 | 70,78 | -0,226 |
| 60,13 | 0,3238 | 70,74 | -0,2643 | 59,98 | 0,3149 | 70,72 | -0,2403 | 59,92 | 0,3194 | 70,82 | -0,2261 |
| 60,1 | 0,3238 | 70,77 | -0,2644 | 59,95 | 0,3148 | 70,76 | -0,2404 | 59,89 | 0,3194 | 70,85 | -0,2261 |
| 60,07 | 0,3237 | 70,81 | -0,2645 | 59,92 | 0,3148 | 70,79 | -0,2404 | 59,86 | 0,3193 | 70,88 | -0,2262 |
| 60,03 | 0,3237 | 70,84 | -0,2646 | 59,88 | 0,3148 | 70,82 | -0,2405 | 59,82 | 0,3193 | 70,92 | -0,2263 |
| 60 | 0,3237 | 70,87 | -0,2646 | 59,85 | 0,3147 | 70,86 | -0,2405 | 59,79 | 0,3192 | 70,95 | -0,2263 |
| 59,97 | 0,3236 | 70,91 | -0,2646 | 59,82 | 0,3147 | 70,89 | -0,2405 | 59,76 | 0,3192 | 70,98 | -0,2264 |
| 59,93 | 0,3236 | 70,94 | -0,2647 | 59,78 | 0,3147 | 70,92 | -0,2406 | 59,72 | 0,3191 | 71,02 | -0,2265 |
| 59,9 | 0,3235 | 70,97 | -0,2647 | 59,75 | 0,3146 | 70,96 | -0,2406 | 59,69 | 0,3191 | 71,05 | -0,2265 |
| 59,87 | 0,3235 | 71,01 | -0,2648 | 59,71 | 0,3146 | 70,99 | -0,2407 | 59,66 | 0,319 | 71,08 | -0,2266 |
| 59,83 | 0,3235 | 71,04 | -0,2648 | 59,68 | 0,3145 | 71,02 | -0,2407 | 59,62 | 0,3189 | 71,12 | -0,2267 |
| 59,8 | 0,3234 | 71,07 | -0,2649 | 59,65 | 0,3145 | 71,06 | -0,2407 | 59,59 | 0,3189 | 71,15 | -0,2267 |
| 59,77 | 0,3234 | 71,11 | -0,2649 | 59,62 | 0,3145 | 71,09 | -0,2408 | 59,55 | 0,3188 | 71,18 | -0,2268 |
| 59,73 | 0,3233 | 71,14 | -0,265 | 59,58 | 0,3144 | 71,12 | -0,2408 | 59,52 | 0,3188 | 71,22 | -0,2269 |
| 59,7 | 0,3233 | 71,17 | -0,265 | 59,55 | 0,3144 | 71,16 | -0,2409 | 59,49 | 0,3187 | 71,25 | -0,2269 |
| 59,67 | 0,3233 | 71,21 | -0,265 | 59,51 | 0,3144 | 71,19 | -0,2409 | 59,46 | 0,3187 | 71,28 | -0,227 |
| 59,63 | 0,3232 | 71,24 | -0,2651 | 59,48 | 0,3143 | 71,22 | -0,2409 | 59,42 | 0,3186 | 71,32 | -0,227 |
| 59,6 | 0,3232 | 71,27 | -0,2651 | 59,45 | 0,3143 | 71,26 | -0,241 | 59,39 | 0,3185 | 71,35 | -0,2271 |
| 59,57 | 0,3231 | 71,31 | -0,2652 | 59,41 | 0,3143 | 71,29 | -0,241 | 59,36 | 0,3185 | 71,38 | -0,2272 |
| 59,53 | 0,3231 | 71,34 | -0,2652 | 59,38 | 0,3142 | 71,32 | -0,241 | 59,32 | 0,3184 | 71,42 | -0,2272 |
| 59,5 | 0,3231 | 71,37 | -0,2653 | 59,35 | 0,3142 | 71,35 | -0,2411 | 59,29 | 0,3184 | 71,45 | -0,2273 |
| 59,47 | 0,323 | 71,41 | -0,2653 | 59,32 | 0,3141 | 71,39 | -0,2411 | 59,25 | 0,3183 | 71,48 | -0,2274 |
| 59,43 | 0,323 | 71,44 | -0,2654 | 59,28 | 0,3141 | 71,42 | -0,2412 | 59,22 | 0,3183 | 71,52 | -0,2274 |
| 59,4 | 0,3229 | 71,47 | -0,2654 | 59,25 | 0,3141 | 71,46 | -0,2412 | 59,19 | 0,3182 | 71,55 | -0,2275 |
| 59,37 | 0,3229 | 71,51 | -0,2654 | 59,21 | 0,314 | 71,49 | -0,2412 | 59,15 | 0,3182 | 71,58 | -0,2276 |
| 59,33 | 0,3228 | 71,54 | -0,2655 | 59,18 | 0,314 | 71,52 | -0,2413 | 59,12 | 0,3181 | 71,62 | -0,2276 |
| 59,3 | 0,3228 | 71,57 | -0,2655 | 59,15 | 0,314 | 71,56 | -0,2413 | 59,09 | 0,318 | 71,65 | -0,2277 |
| 59,27 | 0,3228 | 71,61 | -0,2656 | 59,11 | 0,3139 | 71,59 | -0,2414 | 59,05 | 0,318 | 71,68 | -0,2278 |
| 59,23 | 0,3227 | 71,64 | -0,2656 | 59,08 | 0,3139 | 71,62 | -0,2414 | 59,02 | 0,3179 | 71,72 | -0,2278 |
| 59,2 | 0,3227 | 71,67 | -0,2657 | 59,05 | 0,3139 | 71,66 | -0,2415 | 58,99 | 0,3179 | 71,75 | -0,2279 |
| 59,17 | 0,3227 | 71,71 | -0,2657 | 59,01 | 0,3138 | 71,69 | -0,2415 | 58,95 | 0,3178 | 71,78 | -0,228 |
| 59,13 | 0,3226 | 71,74 | -0,2658 | 58,98 | 0,3138 | 71,72 | -0,2415 | 58,92 | 0,3178 | 71,82 | -0,2281 |
| 59,1 | 0,3226 | 71,77 | -0,2658 | 58,95 | 0,3137 | 71,75 | -0,2416 | 58,89 | 0,3177 | 71,85 | -0,2281 |
| 59,07 | 0,3225 | 71,81 | -0,2658 | 58,91 | 0,3137 | 71,79 | -0,2416 | 58,85 | 0,3176 | 71,88 | -0,2282 |
| 59,03 | 0,3225 | 71,84 | -0,2659 | 58,88 | 0,3137 | 71,82 | -0,2417 | 58,82 | 0,3176 | 71,92 | -0,2282 |
| 59 | 0,3224 | 71,87 | -0,2659 | 58,85 | 0,3136 | 71,86 | -0,2417 | 58,79 | 0,3175 | 71,95 | -0,2283 |
| 58,97 | 0,3224 | 71,91 | -0,266 | 58,81 | 0,3136 | 71,89 | -0,2418 | 58,75 | 0,3175 | 71,98 | -0,2284 |
| 58,93 | 0,3224 | 71,94 | -0,266 | 58,78 | 0,3136 | 71,92 | -0,2418 | 58,72 | 0,3174 | 72,02 | -0,2284 |
| 58,9 | 0,3223 | 71,97 | -0,2661 | 58,75 | 0,3135 | 71,96 | -0,2418 | 58,69 | 0,3173 | 72,05 | -0,2285 |
| 58,87 | 0,3223 | 72,01 | -0,2661 | 58,71 | 0,3135 | 71,99 | -0,2419 | 58,65 | 0,3173 | 72,08 | -0,2286 |
| 58,83 | 0,3223 | 72,04 | -0,2662 | 58,68 | 0,3134 | 72,02 | -0,2419 | 58,62 | 0,3172 | 72,12 | -0,2286 |
| 58,8 | 0,3222 | 72,07 | -0,2662 | 58,65 | 0,3134 | 72,06 | -0,242 | 58,59 | 0,3172 | 72,15 | -0,2287 |
| 58,77 | 0,3222 | 72,11 | -0,2663 | 58,61 | 0,3134 | 72,09 | -0,242 | 58,55 | 0,3171 | 72,18 | -0,2288 |
| 58,73 | 0,3221 | 72,14 | -0,2663 | 58,58 | 0,3133 | 72,12 | -0,242 | 58,52 | 0,3171 | 72,22 | -0,2288 |
| 58,7 | 0,3221 | 72,17 | -0,2663 | 58,55 | 0,3133 | 72,16 | -0,2421 | 58,49 | 0,317 | 72,25 | -0,2289 |
| 58,67 | 0,322 | 72,21 | -0,2664 | 58,51 | 0,3133 | 72,19 | -0,2421 | 58,45 | 0,3169 | 72,28 | -0,229 |
| 58,63 | 0,322 | 72,24 | -0,2664 | 58,48 | 0,3132 | 72,22 | -0,2422 | 58,42 | 0,3169 | 72,32 | -0,229 |
| 58,6 | 0,322 | 72,27 | -0,2665 | 58,45 | 0,3132 | 72,25 | -0,2422 | 58,39 | 0,3168 | 72,35 | -0,2291 |
| 58,57 | 0,3219 | 72,31 | -0,2665 | 58,41 | 0,3132 | 72,29 | -0,2423 | 58,35 | 0,3168 | 72,38 | -0,2292 |
| 58,53 | 0,3219 | 72,34 | -0,2666 | 58,38 | 0,3131 | 72,32 | -0,2423 | 58,32 | 0,3167 | 72,42 | -0,2292 |
| 58,5 | 0,3218 | 72,37 | -0,2666 | 58,35 | 0,3131 | 72,36 | -0,2423 | 58,29 | 0,3166 | 72,45 | -0,2293 |
| 58,47 | 0,3218 | 72,41 | -0,2667 | 58,31 | 0,313 | 72,39 | -0,2424 | 58,25 | 0,3166 | 72,48 | -0,2294 |
| 58,43 | 0,3218 | 72,44 | -0,2668 | 58,28 | 0,313 | 72,42 | -0,2424 | 58,22 | 0,3165 | 72,52 | -0,2294 |
| 58,4 | 0,3217 | 72,47 | -0,2669 | 58,25 | 0,313 | 72,46 | -0,2425 | 58,18 | 0,3165 | 72,55 | -0,2295 |
| 58,37 | 0,3217 | 72,51 | -0,267 | 58,21 | 0,3129 | 72,49 | -0,2425 | 58,15 | 0,3164 | 72,58 | -0,2296 |
| 58,33 | 0,3217 | 72,54 | -0,267 | 58,18 | 0,3129 | 72,52 | -0,2426 | 58,12 | 0,3163 | 72,62 | -0,2296 |
| 58,3 | 0,3216 | 72,57 | -0,2671 | 58,15 | 0,3129 | 72,56 | -0,2426 | 58,09 | 0,3163 | 72,65 | -0,2297 |
| 58,27 | 0,3216 | 72,61 | -0,2671 | 58,11 | 0,3128 | 72,59 | -0,2426 | 58,05 | 0,3162 | 72,68 | -0,2297 |
| 58,23 | 0,3215 | 72,64 | -0,2672 | 58,08 | 0,3128 | 72,62 | -0,2427 | 58,02 | 0,3162 | 72,72 | -0,2298 |
| 58,2 | 0,3215 | 72,67 | -0,2672 | 58,05 | 0,3127 | 72,66 | -0,2427 | 57,99 | 0,3161 | 72,75 | -0,2299 |
| 58,17 | 0,3214 | 72,71 | -0,2673 | 58,01 | 0,3127 | 72,69 | -0,2428 | 57,95 | 0,316 | 72,78 | -0,2299 |
| 58,13 | 0,3214 | 72,74 | -0,2673 | 57,98 | 0,3127 | 72,72 | -0,2428 | 57,92 | 0,316 | 72,82 | -0,23 |
| 58,1 | 0,3214 | 72,77 | -0,2674 | 57,95 | 0,3126 | 72,76 | -0,2429 | 57,89 | 0,3159 | 72,85 | -0,2301 |
| 58,07 | 0,3213 | 72,81 | -0,2674 | 57,91 | 0,3126 | 72,79 | -0,2429 | 57,85 | 0,3158 | 72,88 | -0,2301 |
| 58,03 | 0,3213 | 72,84 | -0,2674 | 57,88 | 0,3126 | 72,82 | -0,243 | 57,82 | 0,3158 | 72,92 | -0,2302 |
| 58 | 0,3212 | 72,87 | -0,2675 | 57,85 | 0,3125 | 72,86 | -0,243 | 57,79 | 0,3157 | 72,95 | -0,2303 |
| 57,97 | 0,3212 | 72,91 | -0,2675 | 57,81 | 0,3125 | 72,89 | -0,243 | 57,75 | 0,3157 | 72,98 | -0,2303 |
| 57,93 | 0,3212 | 72,94 | -0,2676 | 57,78 | 0,3124 | 72,92 | -0,2431 | 57,72 | 0,3156 | 73,02 | -0,2304 |
| 57,9 | 0,3211 | 72,97 | -0,2676 | 57,75 | 0,3124 | 72,96 | -0,2431 | 57,69 | 0,3155 | 73,05 | -0,2305 |
| 57,87 | 0,3211 | 73,01 | -0,2677 | 57,71 | 0,3124 | 72,99 | -0,2432 | 57,65 | 0,3155 | 73,08 | -0,2305 |
| 57,83 | 0,3211 | 73,04 | -0,2677 | 57,68 | 0,3123 | 73,02 | -0,2432 | 57,62 | 0,3154 | 73,12 | -0,2306 |
| 57,8 | 0,321 | 73,07 | -0,2678 | 57,65 | 0,3123 | 73,06 | -0,2433 | 57,59 | 0,3154 | 73,15 | -0,2307 |
| 57,77 | 0,321 | 73,11 | -0,2678 | 57,61 | 0,3123 | 73,09 | -0,2433 | 57,55 | 0,3153 | 73,18 | -0,2307 |
| 57,73 | 0,3209 | 73,14 | -0,2679 | 57,58 | 0,3122 | 73,12 | -0,2434 | 57,52 | 0,3152 | 73,22 | -0,2308 |
| 57,7 | 0,3209 | 73,17 | -0,2679 | 57,55 | 0,3122 | 73,15 | -0,2434 | 57,48 | 0,3152 | 73,25 | -0,2309 |
| 57,67 | 0,3208 | 73,21 | -0,268 | 57,51 | 0,3122 | 73,19 | -0,2434 | 57,45 | 0,3151 | 73,28 | -0,2309 |
| 57,63 | 0,3208 | 73,24 | -0,268 | 57,48 | 0,3121 | 73,22 | -0,2435 | 57,42 | 0,3151 | 73,32 | -0,231 |
| 57,6 | 0,3208 | 73,27 | -0,268 | 57,45 | 0,3121 | 73,26 | -0,2435 | 57,38 | 0,315 | 73,35 | -0,2311 |
| 57,57 | 0,3207 | 73,31 | -0,2681 | 57,41 | 0,312 | 73,29 | -0,2436 | 57,35 | 0,3149 | 73,38 | -0,2311 |
| 57,53 | 0,3207 | 73,34 | -0,2681 | 57,38 | 0,312 | 73,32 | -0,2436 | 57,32 | 0,3149 | 73,42 | -0,2312 |
| 57,5 | 0,3206 | 73,37 | -0,2682 | 57,35 | 0,312 | 73,36 | -0,2437 | 57,28 | 0,3148 | 73,45 | -0,2313 |
| 57,47 | 0,3206 | 73,41 | -0,2682 | 57,31 | 0,3119 | 73,39 | -0,2437 | 57,25 | 0,3148 | 73,48 | -0,2313 |
| 57,43 | 0,3206 | 73,44 | -0,2683 | 57,28 | 0,3119 | 73,42 | -0,2437 | 57,22 | 0,3147 | 73,52 | -0,2314 |
| 57,4 | 0,3205 | 73,47 | -0,2683 | 57,25 | 0,3119 | 73,45 | -0,2438 | 57,18 | 0,3146 | 73,55 | -0,2315 |
| 57,37 | 0,3205 | 73,51 | -0,2684 | 57,21 | 0,3118 | 73,49 | -0,2438 | 57,15 | 0,3146 | 73,58 | -0,2315 |
| 57,33 | 0,3205 | 73,54 | -0,2684 | 57,18 | 0,3118 | 73,52 | -0,2439 | 57,12 | 0,3145 | 73,62 | -0,2316 |
| 57,3 | 0,3204 | 73,57 | -0,2685 | 57,15 | 0,3117 | 73,55 | -0,2439 | 57,08 | 0,3144 | 73,65 | -0,2316 |
| 57,27 | 0,3204 | 73,61 | -0,2685 | 57,11 | 0,3117 | 73,59 | -0,244 | 57,05 | 0,3144 | 73,68 | -0,2317 |
| 57,23 | 0,3203 | 73,64 | -0,2686 | 57,08 | 0,3117 | 73,62 | -0,244 | 57,02 | 0,3143 | 73,72 | -0,2318 |
| 57,2 | 0,3203 | 73,67 | -0,2686 | 57,05 | 0,3116 | 73,65 | -0,2441 | 56,98 | 0,3142 | 73,75 | -0,2319 |
| 57,17 | 0,3202 | 73,7 | -0,2687 | 57,01 | 0,3116 | 73,69 | -0,2441 | 56,95 | 0,3142 | 73,78 | -0,232 |
| 57,13 | 0,3202 | 73,74 | -0,2687 | 56,98 | 0,3116 | 73,72 | -0,2441 | 56,92 | 0,3141 | 73,82 | -0,232 |
| 57,1 | 0,3202 | 73,77 | -0,2688 | 56,95 | 0,3115 | 73,76 | -0,2442 | 56,88 | 0,314 | 73,85 | -0,2321 |
| 57,07 | 0,3201 | 73,81 | -0,2688 | 56,91 | 0,3115 | 73,79 | -0,2442 | 56,85 | 0,3139 | 73,88 | -0,2322 |
| 57,03 | 0,3201 | 73,84 | -0,2689 | 56,88 | 0,3114 | 73,82 | -0,2443 | 56,82 | 0,3138 | 73,92 | -0,2322 |
| 57 | 0,3201 | 73,87 | -0,2689 | 56,85 | 0,3114 | 73,86 | -0,2444 | 56,78 | 0,3137 | 73,95 | -0,2323 |
| 56,97 | 0,32 | 73,91 | -0,269 | 56,81 | 0,3114 | 73,89 | -0,2444 | 56,75 | 0,3136 | 73,98 | -0,2324 |
| 56,93 | 0,32 | 73,94 | -0,2691 | 56,78 | 0,3113 | 73,92 | -0,2444 | 56,72 | 0,3135 | 74,02 | -0,2324 |
| 56,9 | 0,3199 | 73,97 | -0,2691 | 56,75 | 0,3113 | 73,96 | -0,2445 | 56,68 | 0,3135 | 74,05 | -0,2325 |
| 56,87 | 0,3199 | 74,01 | -0,2692 | 56,71 | 0,3113 | 73,99 | -0,2445 | 56,65 | 0,3134 | 74,08 | -0,2326 |
| 56,83 | 0,3199 | 74,04 | -0,2692 | 56,68 | 0,3112 | 74,02 | -0,2446 | 56,62 | 0,3133 | 74,11 | -0,2326 |
| 56,8 | 0,3198 | 74,07 | -0,2693 | 56,65 | 0,3112 | 74,05 | -0,2446 | 56,58 | 0,3133 | 74,15 | -0,2327 |
| 56,77 | 0,3198 | 74,11 | -0,2693 | 56,61 | 0,3111 | 74,09 | -0,2447 | 56,55 | 0,3132 | 74,18 | -0,2328 |
| 56,73 | 0,3197 | 74,14 | -0,2694 | 56,58 | 0,3111 | 74,12 | -0,2447 | 56,52 | 0,3132 | 74,22 | -0,2328 |
| 56,7 | 0,3197 | 74,17 | -0,2694 | 56,55 | 0,3111 | 74,15 | -0,2448 | 56,48 | 0,3131 | 74,25 | -0,2329 |
| 56,67 | 0,3197 | 74,21 | -0,2694 | 56,51 | 0,311 | 74,19 | -0,2448 | 56,45 | 0,313 | 74,28 | -0,233 |
| 56,63 | 0,3196 | 74,24 | -0,2695 | 56,48 | 0,311 | 74,22 | -0,2448 | 56,42 | 0,313 | 74,32 | -0,233 |
| 56,6 | 0,3196 | 74,27 | -0,2695 | 56,45 | 0,311 | 74,26 | -0,2449 | 56,38 | 0,3129 | 74,35 | -0,2331 |
| 56,57 | 0,3195 | 74,31 | -0,2696 | 56,41 | 0,3109 | 74,29 | -0,2449 | 56,35 | 0,3128 | 74,38 | -0,2332 |
| 56,53 | 0,3195 | 74,34 | -0,2697 | 56,38 | 0,3109 | 74,32 | -0,245 | 56,32 | 0,3127 | 74,42 | -0,2332 |
| 56,5 | 0,3194 | 74,37 | -0,2697 | 56,35 | 0,3108 | 74,36 | -0,245 | 56,28 | 0,3126 | 74,45 | -0,2333 |
| 56,47 | 0,3194 | 74,41 | -0,2698 | 56,31 | 0,3108 | 74,39 | -0,2451 | 56,25 | 0,3126 | 74,48 | -0,2334 |
| 56,43 | 0,3194 | 74,44 | -0,2698 | 56,28 | 0,3108 | 74,42 | -0,2451 | 56,22 | 0,3125 | 74,52 | -0,2335 |
| 56,4 | 0,3193 | 74,47 | -0,2699 | 56,25 | 0,3107 | 74,46 | -0,2452 | 56,18 | 0,3125 | 74,55 | -0,2336 |
| 56,37 | 0,3193 | 74,51 | -0,2699 | 56,21 | 0,3107 | 74,49 | -0,2452 | 56,15 | 0,3124 | 74,58 | -0,2336 |
| 56,33 | 0,3192 | 74,54 | -0,27 | 56,18 | 0,3106 | 74,52 | -0,2453 | 56,12 | 0,3123 | 74,62 | -0,2337 |
| 56,3 | 0,3192 | 74,57 | -0,27 | 56,15 | 0,3106 | 74,56 | -0,2453 | 56,08 | 0,3123 | 74,65 | -0,2338 |
| 56,27 | 0,3192 | 74,61 | -0,2701 | 56,11 | 0,3106 | 74,59 | -0,2454 | 56,05 | 0,3122 | 74,68 | -0,2338 |
| 56,23 | 0,3191 | 74,64 | -0,2701 | 56,08 | 0,3105 | 74,62 | -0,2454 | 56,02 | 0,3121 | 74,72 | -0,2339 |
| 56,2 | 0,3191 | 74,67 | -0,2702 | 56,05 | 0,3105 | 74,66 | -0,2455 | 55,98 | 0,3121 | 74,75 | -0,234 |
| 56,17 | 0,319 | 74,71 | -0,2702 | 56,01 | 0,3105 | 74,69 | -0,2455 | 55,95 | 0,312 | 74,78 | -0,234 |
| 56,13 | 0,319 | 74,74 | -0,2703 | 55,98 | 0,3104 | 74,72 | -0,2455 | 55,92 | 0,3119 | 74,82 | -0,2341 |
| 56,1 | 0,319 | 74,77 | -0,2703 | 55,95 | 0,3104 | 74,76 | -0,2456 | 55,88 | 0,3118 | 74,85 | -0,2342 |
| 56,07 | 0,3189 | 74,81 | -0,2704 | 55,91 | 0,3104 | 74,79 | -0,2456 | 55,85 | 0,3117 | 74,88 | -0,2342 |
| 56,03 | 0,3189 | 74,84 | -0,2704 | 55,88 | 0,3103 | 74,82 | -0,2457 | 55,82 | 0,3116 | 74,92 | -0,2343 |
| 56 | 0,3188 | 74,87 | -0,2705 | 55,85 | 0,3103 | 74,86 | -0,2457 | 55,78 | 0,3116 | 74,95 | -0,2344 |
| 55,97 | 0,3188 | 74,91 | -0,2705 | 55,81 | 0,3102 | 74,89 | -0,2458 | 55,75 | 0,3115 | 74,98 | -0,2344 |
| 55,93 | 0,3188 | 74,94 | -0,2706 | 55,78 | 0,3102 | 74,92 | -0,2458 | 55,72 | 0,3114 | 75,02 | -0,2345 |
| 55,9 | 0,3187 | 74,97 | -0,2706 | 55,75 | 0,3102 | 74,96 | -0,2458 | 55,68 | 0,3114 | 75,05 | -0,2346 |
| 55,87 | 0,3187 | 75,01 | -0,2707 | 55,71 | 0,3101 | 74,99 | -0,2459 | 55,65 | 0,3113 | 75,08 | -0,2346 |
| 55,83 | 0,3186 | 75,04 | -0,2707 | 55,68 | 0,3101 | 75,02 | -0,2459 | 55,62 | 0,3112 | 75,12 | -0,2347 |
| 55,8 | 0,3186 | 75,07 | -0,2708 | 55,65 | 0,31 | 75,06 | -0,246 | 55,58 | 0,3111 | 75,15 | -0,2348 |
| 55,77 | 0,3186 | 75,11 | -0,2708 | 55,61 | 0,31 | 75,09 | -0,246 | 55,55 | 0,3111 | 75,18 | -0,2349 |
| 55,73 | 0,3185 | 75,14 | -0,2709 | 55,58 | 0,31 | 75,12 | -0,2461 | 55,52 | 0,311 | 75,22 | -0,2349 |
| 55,7 | 0,3185 | 75,17 | -0,2709 | 55,55 | 0,3099 | 75,16 | -0,2461 | 55,48 | 0,3109 | 75,25 | -0,235 |
| 55,67 | 0,3184 | 75,21 | -0,271 | 55,51 | 0,3099 | 75,19 | -0,2462 | 55,45 | 0,3109 | 75,28 | -0,2351 |
| 55,63 | 0,3184 | 75,24 | -0,271 | 55,48 | 0,3098 | 75,22 | -0,2462 | 55,42 | 0,3108 | 75,32 | -0,2351 |
| 55,6 | 0,3184 | 75,27 | -0,2711 | 55,45 | 0,3098 | 75,26 | -0,2463 | 55,38 | 0,3107 | 75,35 | -0,2352 |
| 55,57 | 0,3183 | 75,3 | -0,2711 | 55,41 | 0,3098 | 75,29 | -0,2463 | 55,35 | 0,3106 | 75,38 | -0,2353 |
| 55,53 | 0,3183 | 75,34 | -0,2712 | 55,38 | 0,3097 | 75,32 | -0,2463 | 55,32 | 0,3106 | 75,42 | -0,2353 |
| 55,5 | 0,3182 | 75,37 | -0,2712 | 55,34 | 0,3097 | 75,36 | -0,2464 | 55,28 | 0,3105 | 75,45 | -0,2354 |
| 55,47 | 0,3182 | 75,4 | -0,2713 | 55,31 | 0,3097 | 75,39 | -0,2464 | 55,25 | 0,3104 | 75,48 | -0,2355 |
| 55,43 | 0,3182 | 75,44 | -0,2713 | 55,28 | 0,3096 | 75,42 | -0,2465 | 55,22 | 0,3104 | 75,52 | -0,2355 |
| 55,4 | 0,3181 | 75,47 | -0,2714 | 55,25 | 0,3096 | 75,46 | -0,2465 | 55,18 | 0,3103 | 75,55 | -0,2356 |
| 55,37 | 0,3181 | 75,51 | -0,2714 | 55,21 | 0,3095 | 75,49 | -0,2466 | 55,15 | 0,3102 | 75,58 | -0,2357 |
| 55,33 | 0,318 | 75,54 | -0,2715 | 55,18 | 0,3095 | 75,52 | -0,2466 | 55,12 | 0,3102 | 75,62 | -0,2357 |
| 55,3 | 0,318 | 75,57 | -0,2715 | 55,15 | 0,3095 | 75,55 | -0,2466 | 55,08 | 0,3101 | 75,65 | -0,2358 |
| 55,27 | 0,318 | 75,6 | -0,2716 | 55,11 | 0,3094 | 75,59 | -0,2467 | 55,05 | 0,31 | 75,68 | -0,2359 |
| 55,23 | 0,3179 | 75,64 | -0,2716 | 55,08 | 0,3094 | 75,62 | -0,2467 | 55,02 | 0,3099 | 75,72 | -0,2359 |
| 55,2 | 0,3179 | 75,67 | -0,2717 | 55,04 | 0,3094 | 75,66 | -0,2468 | 54,98 | 0,3098 | 75,75 | -0,236 |
| 55,17 | 0,3178 | 75,71 | -0,2717 | 55,01 | 0,3093 | 75,69 | -0,2468 | 54,95 | 0,3097 | 75,78 | -0,2361 |
| 55,13 | 0,3178 | 75,74 | -0,2718 | 54,98 | 0,3093 | 75,72 | -0,2469 | 54,92 | 0,3097 | 75,82 | -0,2361 |
| 55,1 | 0,3178 | 75,77 | -0,2718 | 54,95 | 0,3092 | 75,76 | -0,2469 | 54,88 | 0,3096 | 75,85 | -0,2362 |
| 55,07 | 0,3177 | 75,8 | -0,2719 | 54,91 | 0,3092 | 75,79 | -0,247 | 54,85 | 0,3095 | 75,88 | -0,2363 |
| 55,03 | 0,3177 | 75,84 | -0,2719 | 54,88 | 0,3092 | 75,82 | -0,247 | 54,81 | 0,3094 | 75,92 | -0,2363 |
| 55 | 0,3176 | 75,87 | -0,272 | 54,85 | 0,3091 | 75,86 | -0,2471 | 54,78 | 0,3093 | 75,95 | -0,2364 |
| 54,97 | 0,3176 | 75,9 | -0,272 | 54,81 | 0,3091 | 75,89 | -0,2471 | 54,75 | 0,3093 | 75,98 | -0,2365 |
| 54,93 | 0,3176 | 75,94 | -0,2721 | 54,78 | 0,309 | 75,92 | -0,2472 | 54,72 | 0,3092 | 76,02 | -0,2366 |
| 54,9 | 0,3175 | 75,97 | -0,2721 | 54,75 | 0,309 | 75,95 | -0,2472 | 54,68 | 0,3091 | 76,05 | -0,2366 |
| 54,87 | 0,3175 | 76 | -0,2722 | 54,71 | 0,309 | 75,99 | -0,2472 | 54,65 | 0,3091 | 76,08 | -0,2367 |
| 54,83 | 0,3174 | 76,04 | -0,2722 | 54,68 | 0,3089 | 76,02 | -0,2473 | 54,62 | 0,309 | 76,12 | -0,2368 |
| 54,8 | 0,3174 | 76,07 | -0,2723 | 54,65 | 0,3089 | 76,06 | -0,2473 | 54,58 | 0,3089 | 76,15 | -0,2368 |
| 54,77 | 0,3174 | 76,1 | -0,2723 | 54,61 | 0,3089 | 76,09 | -0,2474 | 54,55 | 0,3088 | 76,18 | -0,2369 |
| 54,73 | 0,3173 | 76,14 | -0,2724 | 54,58 | 0,3088 | 76,12 | -0,2474 | 54,52 | 0,3088 | 76,22 | -0,237 |
| 54,7 | 0,3173 | 76,17 | -0,2724 | 54,55 | 0,3088 | 76,16 | -0,2475 | 54,48 | 0,3087 | 76,25 | -0,237 |
| 54,67 | 0,3172 | 76,2 | -0,2725 | 54,51 | 0,3087 | 76,19 | -0,2475 | 54,45 | 0,3086 | 76,28 | -0,2371 |
| 54,63 | 0,3172 | 76,24 | -0,2725 | 54,48 | 0,3087 | 76,22 | -0,2475 | 54,42 | 0,3086 | 76,32 | -0,2372 |
| 54,6 | 0,3172 | 76,27 | -0,2726 | 54,45 | 0,3087 | 76,26 | -0,2476 | 54,38 | 0,3085 | 76,35 | -0,2372 |
| 54,57 | 0,3171 | 76,3 | -0,2727 | 54,41 | 0,3086 | 76,29 | -0,2476 | 54,35 | 0,3084 | 76,38 | -0,2373 |
| 54,53 | 0,3171 | 76,34 | -0,2727 | 54,38 | 0,3086 | 76,32 | -0,2477 | 54,32 | 0,3084 | 76,42 | -0,2374 |
| 54,5 | 0,317 | 76,37 | -0,2728 | 54,35 | 0,3086 | 76,36 | -0,2477 | 54,28 | 0,3083 | 76,45 | -0,2374 |
| 54,47 | 0,317 | 76,41 | -0,2728 | 54,31 | 0,3085 | 76,39 | -0,2478 | 54,25 | 0,3082 | 76,48 | -0,2375 |
| 54,43 | 0,317 | 76,44 | -0,2729 | 54,28 | 0,3085 | 76,42 | -0,2478 | 54,22 | 0,3081 | 76,52 | -0,2376 |
| 54,4 | 0,3169 | 76,47 | -0,2729 | 54,25 | 0,3084 | 76,46 | -0,2479 | 54,18 | 0,3081 | 76,55 | -0,2377 |
| 54,37 | 0,3169 | 76,51 | -0,273 | 54,21 | 0,3084 | 76,49 | -0,2479 | 54,15 | 0,308 | 76,58 | -0,2377 |
| 54,33 | 0,3168 | 76,54 | -0,273 | 54,18 | 0,3084 | 76,52 | -0,248 | 54,12 | 0,3079 | 76,62 | -0,2378 |
| 54,3 | 0,3168 | 76,57 | -0,2731 | 54,15 | 0,3083 | 76,56 | -0,248 | 54,08 | 0,3078 | 76,65 | -0,2379 |
| 54,27 | 0,3168 | 76,6 | -0,2731 | 54,11 | 0,3083 | 76,59 | -0,248 | 54,05 | 0,3078 | 76,68 | -0,2379 |
| 54,23 | 0,3167 | 76,64 | -0,2732 | 54,08 | 0,3083 | 76,62 | -0,2481 | 54,01 | 0,3077 | 76,72 | -0,238 |
| 54,2 | 0,3167 | 76,67 | -0,2732 | 54,05 | 0,3082 | 76,66 | -0,2481 | 53,98 | 0,3076 | 76,75 | -0,2381 |
| 54,17 | 0,3166 | 76,71 | -0,2733 | 54,01 | 0,3082 | 76,69 | -0,2482 | 53,95 | 0,3075 | 76,78 | -0,2381 |
| 54,13 | 0,3166 | 76,74 | -0,2733 | 53,98 | 0,3081 | 76,72 | -0,2482 | 53,92 | 0,3075 | 76,82 | -0,2382 |
| 54,1 | 0,3166 | 76,77 | -0,2734 | 53,95 | 0,3081 | 76,76 | -0,2483 | 53,88 | 0,3074 | 76,85 | -0,2383 |
| 54,07 | 0,3165 | 76,8 | -0,2734 | 53,91 | 0,3081 | 76,79 | -0,2483 | 53,85 | 0,3073 | 76,88 | -0,2383 |
| 54,03 | 0,3165 | 76,84 | -0,2735 | 53,88 | 0,308 | 76,82 | -0,2484 | 53,82 | 0,3072 | 76,92 | -0,2384 |
| 54 | 0,3165 | 76,87 | -0,2735 | 53,84 | 0,308 | 76,85 | -0,2484 | 53,78 | 0,3071 | 76,95 | -0,2385 |
| 53,97 | 0,3164 | 76,91 | -0,2736 | 53,81 | 0,3079 | 76,89 | -0,2484 | 53,75 | 0,3071 | 76,98 | -0,2385 |
| 53,93 | 0,3164 | 76,94 | -0,2736 | 53,78 | 0,3079 | 76,92 | -0,2485 | 53,72 | 0,307 | 77,02 | -0,2386 |
| 53,9 | 0,3163 | 76,97 | -0,2737 | 53,75 | 0,3079 | 76,95 | -0,2485 | 53,68 | 0,3069 | 77,05 | -0,2387 |
| 53,87 | 0,3163 | 77 | -0,2737 | 53,71 | 0,3078 | 76,99 | -0,2486 | 53,65 | 0,3068 | 77,08 | -0,2387 |
| 53,83 | 0,3163 | 77,04 | -0,2738 | 53,68 | 0,3078 | 77,02 | -0,2486 | 53,62 | 0,3068 | 77,12 | -0,2388 |
| 53,8 | 0,3162 | 77,07 | -0,2738 | 53,64 | 0,3078 | 77,05 | -0,2487 | 53,58 | 0,3066 | 77,15 | -0,2389 |
| 53,77 | 0,3162 | 77,1 | -0,2739 | 53,61 | 0,3077 | 77,09 | -0,2487 | 53,55 | 0,3066 | 77,18 | -0,239 |
| 53,73 | 0,3161 | 77,14 | -0,2739 | 53,58 | 0,3077 | 77,12 | -0,2488 | 53,51 | 0,3065 | 77,22 | -0,239 |
| 53,7 | 0,3161 | 77,17 | -0,274 | 53,54 | 0,3077 | 77,16 | -0,2488 | 53,48 | 0,3064 | 77,25 | -0,2391 |
| 53,67 | 0,3161 | 77,2 | -0,274 | 53,51 | 0,3076 | 77,19 | -0,2488 | 53,45 | 0,3063 | 77,28 | -0,2392 |
| 53,63 | 0,316 | 77,24 | -0,2741 | 53,48 | 0,3076 | 77,22 | -0,2489 | 53,41 | 0,3063 | 77,32 | -0,2392 |
| 53,6 | 0,316 | 77,27 | -0,2741 | 53,44 | 0,3075 | 77,25 | -0,2489 | 53,38 | 0,3062 | 77,35 | -0,2393 |
| 53,57 | 0,3159 | 77,3 | -0,2742 | 53,41 | 0,3075 | 77,29 | -0,249 | 53,35 | 0,3061 | 77,38 | -0,2394 |
| 53,53 | 0,3159 | 77,34 | -0,2742 | 53,38 | 0,3075 | 77,32 | -0,249 | 53,31 | 0,306 | 77,42 | -0,2394 |
| 53,5 | 0,3159 | 77,37 | -0,2743 | 53,35 | 0,3074 | 77,36 | -0,2491 | 53,28 | 0,306 | 77,45 | -0,2395 |
| 53,47 | 0,3158 | 77,4 | -0,2743 | 53,31 | 0,3074 | 77,39 | -0,2491 | 53,25 | 0,3059 | 77,48 | -0,2396 |
| 53,43 | 0,3158 | 77,44 | -0,2744 | 53,28 | 0,3074 | 77,42 | -0,2492 | 53,21 | 0,3058 | 77,52 | -0,2396 |
| 53,4 | 0,3157 | 77,47 | -0,2744 | 53,25 | 0,3073 | 77,46 | -0,2492 | 53,18 | 0,3057 | 77,55 | -0,2397 |
| 53,37 | 0,3157 | 77,5 | -0,2745 | 53,21 | 0,3073 | 77,49 | -0,2492 | 53,15 | 0,3057 | 77,58 | -0,2398 |
| 53,33 | 0,3157 | 77,54 | -0,2745 | 53,18 | 0,3072 | 77,52 | -0,2493 | 53,11 | 0,3056 | 77,62 | -0,2398 |
| 53,3 | 0,3156 | 77,57 | -0,2746 | 53,14 | 0,3072 | 77,56 | -0,2493 | 53,08 | 0,3055 | 77,65 | -0,2399 |
| 53,27 | 0,3156 | 77,6 | -0,2746 | 53,11 | 0,3072 | 77,59 | -0,2494 | 53,05 | 0,3054 | 77,68 | -0,24 |
| 53,23 | 0,3156 | 77,64 | -0,2747 | 53,08 | 0,3071 | 77,62 | -0,2494 | 53,01 | 0,3053 | 77,72 | -0,24 |
| 53,2 | 0,3155 | 77,67 | -0,2747 | 53,05 | 0,3071 | 77,66 | -0,2495 | 52,98 | 0,3052 | 77,75 | -0,2401 |
| 53,16 | 0,3155 | 77,7 | -0,2748 | 53,01 | 0,3071 | 77,69 | -0,2495 | 52,95 | 0,3051 | 77,78 | -0,2402 |
| 53,13 | 0,3154 | 77,74 | -0,2748 | 52,98 | 0,307 | 77,72 | -0,2496 | 52,91 | 0,305 | 77,82 | -0,2402 |
| 53,1 | 0,3154 | 77,77 | -0,2749 | 52,94 | 0,307 | 77,75 | -0,2496 | 52,88 | 0,3049 | 77,85 | -0,2403 |
| 53,06 | 0,3154 | 77,81 | -0,2749 | 52,91 | 0,307 | 77,79 | -0,2496 | 52,85 | 0,3049 | 77,88 | -0,2404 |
| 53,03 | 0,3153 | 77,84 | -0,275 | 52,88 | 0,3069 | 77,82 | -0,2497 | 52,81 | 0,3048 | 77,92 | -0,2404 |
| 53 | 0,3153 | 77,87 | -0,275 | 52,85 | 0,3069 | 77,85 | -0,2497 | 52,78 | 0,3047 | 77,95 | -0,2405 |
| 52,97 | 0,3152 | 77,91 | -0,2751 | 52,81 | 0,3068 | 77,89 | -0,2498 | 52,75 | 0,3046 | 77,98 | -0,2406 |
| 52,93 | 0,3152 | 77,94 | -0,2751 | 52,78 | 0,3068 | 77,92 | -0,2498 | 52,71 | 0,3045 | 78,02 | -0,2406 |
| 52,9 | 0,3152 | 77,97 | -0,2752 | 52,74 | 0,3068 | 77,96 | -0,2499 | 52,68 | 0,3045 | 78,05 | -0,2407 |
| 52,87 | 0,3151 | 78,01 | -0,2752 | 52,71 | 0,3067 | 77,99 | -0,2499 | 52,65 | 0,3044 | 78,08 | -0,2408 |
| 52,83 | 0,3151 | 78,04 | -0,2753 | 52,68 | 0,3067 | 78,02 | -0,25 | 52,61 | 0,3043 | 78,12 | -0,2408 |
| 52,8 | 0,315 | 78,07 | -0,2754 | 52,65 | 0,3067 | 78,05 | -0,25 | 52,58 | 0,3042 | 78,15 | -0,2409 |
| 52,76 | 0,315 | 78,1 | -0,2754 | 52,61 | 0,3066 | 78,09 | -0,25 | 52,55 | 0,3042 | 78,18 | -0,241 |
| 52,73 | 0,315 | 78,14 | -0,2755 | 52,58 | 0,3066 | 78,12 | -0,2501 | 52,51 | 0,3041 | 78,22 | -0,241 |
| 52,7 | 0,3149 | 78,17 | -0,2755 | 52,54 | 0,3065 | 78,16 | -0,2501 | 52,48 | 0,304 | 78,25 | -0,2411 |
| 52,66 | 0,3149 | 78,21 | -0,2755 | 52,51 | 0,3065 | 78,19 | -0,2502 | 52,45 | 0,3039 | 78,28 | -0,2412 |
| 52,63 | 0,3149 | 78,24 | -0,2756 | 52,48 | 0,3065 | 78,22 | -0,2502 | 52,41 | 0,3038 | 78,32 | -0,2412 |
| 52,6 | 0,3148 | 78,27 | -0,2756 | 52,45 | 0,3064 | 78,26 | -0,2503 | 52,38 | 0,3037 | 78,35 | -0,2413 |
| 52,56 | 0,3148 | 78,31 | -0,2757 | 52,41 | 0,3064 | 78,29 | -0,2503 | 52,35 | 0,3037 | 78,38 | -0,2414 |
| 52,53 | 0,3147 | 78,34 | -0,2758 | 52,38 | 0,3064 | 78,32 | -0,2504 | 52,31 | 0,3036 | 78,42 | -0,2414 |
| 52,5 | 0,3147 | 78,37 | -0,2758 | 52,34 | 0,3063 | 78,36 | -0,2504 | 52,28 | 0,3035 | 78,45 | -0,2415 |
| 52,46 | 0,3147 | 78,4 | -0,2758 | 52,31 | 0,3063 | 78,39 | -0,2504 | 52,25 | 0,3034 | 78,48 | -0,2416 |
| 52,43 | 0,3146 | 78,44 | -0,2759 | 52,28 | 0,3063 | 78,42 | -0,2505 | 52,21 | 0,3033 | 78,52 | -0,2416 |
| 52,4 | 0,3146 | 78,47 | -0,276 | 52,24 | 0,3062 | 78,46 | -0,2505 | 52,18 | 0,3032 | 78,55 | -0,2417 |
| 52,37 | 0,3146 | 78,51 | -0,276 | 52,21 | 0,3062 | 78,49 | -0,2506 | 52,15 | 0,3031 | 78,58 | -0,2418 |
| 52,33 | 0,3145 | 78,54 | -0,2761 | 52,18 | 0,3061 | 78,52 | -0,2506 | 52,11 | 0,303 | 78,62 | -0,2418 |
| 52,3 | 0,3145 | 78,57 | -0,2761 | 52,14 | 0,3061 | 78,56 | -0,2507 | 52,08 | 0,3029 | 78,65 | -0,2419 |
| 52,26 | 0,3144 | 78,61 | -0,2762 | 52,11 | 0,3061 | 78,59 | -0,2507 | 52,05 | 0,3028 | 78,68 | -0,242 |
| 52,23 | 0,3144 | 78,64 | -0,2762 | 52,08 | 0,306 | 78,62 | -0,2508 | 52,01 | 0,3027 | 78,72 | -0,242 |
| 52,2 | 0,3144 | 78,67 | -0,2763 | 52,04 | 0,306 | 78,66 | -0,2508 | 51,98 | 0,3026 | 78,75 | -0,2421 |
| 52,16 | 0,3143 | 78,71 | -0,2763 | 52,01 | 0,306 | 78,69 | -0,2508 | 51,95 | 0,3025 | 78,78 | -0,2422 |
| 52,13 | 0,3143 | 78,74 | -0,2764 | 51,98 | 0,3059 | 78,72 | -0,2509 | 51,91 | 0,3024 | 78,82 | -0,2422 |
| 52,1 | 0,3142 | 78,77 | -0,2764 | 51,94 | 0,3059 | 78,76 | -0,2509 | 51,88 | 0,3023 | 78,85 | -0,2423 |
| 52,06 | 0,3142 | 78,8 | -0,2765 | 51,91 | 0,3059 | 78,79 | -0,251 | 51,85 | 0,3022 | 78,88 | -0,2424 |
| 52,03 | 0,3142 | 78,84 | -0,2765 | 51,88 | 0,3058 | 78,82 | -0,251 | 51,81 | 0,3022 | 78,92 | -0,2424 |
| 52 | 0,3141 | 78,87 | -0,2766 | 51,84 | 0,3058 | 78,86 | -0,2511 | 51,78 | 0,3021 | 78,95 | -0,2425 |
| 51,96 | 0,3141 | 78,91 | -0,2766 | 51,81 | 0,3057 | 78,89 | -0,2511 | 51,74 | 0,302 | 78,98 | -0,2426 |
| 51,93 | 0,3141 | 78,94 | -0,2767 | 51,78 | 0,3057 | 78,92 | -0,2512 | 51,71 | 0,3019 | 79,02 | -0,2426 |
| 51,9 | 0,314 | 78,97 | -0,2767 | 51,75 | 0,3057 | 78,96 | -0,2512 | 51,68 | 0,3018 | 79,05 | -0,2427 |
| 51,86 | 0,314 | 79,01 | -0,2768 | 51,71 | 0,3056 | 78,99 | -0,2512 | 51,64 | 0,3017 | 79,08 | -0,2428 |
| 51,83 | 0,314 | 79,04 | -0,2768 | 51,68 | 0,3056 | 79,02 | -0,2513 | 51,61 | 0,3017 | 79,12 | -0,2428 |
| 51,8 | 0,3139 | 79,07 | -0,2768 | 51,64 | 0,3056 | 79,06 | -0,2513 | 51,58 | 0,3016 | 79,15 | -0,2429 |
| 51,76 | 0,3139 | 79,11 | -0,2769 | 51,61 | 0,3055 | 79,09 | -0,2514 | 51,54 | 0,3015 | 79,18 | -0,243 |
| 51,73 | 0,3138 | 79,14 | -0,277 | 51,58 | 0,3055 | 79,12 | -0,2514 | 51,51 | 0,3014 | 79,22 | -0,243 |
| 51,7 | 0,3138 | 79,17 | -0,2771 | 51,54 | 0,3054 | 79,16 | -0,2515 | 51,48 | 0,3013 | 79,25 | -0,2431 |
| 51,66 | 0,3138 | 79,2 | -0,2771 | 51,51 | 0,3054 | 79,19 | -0,2515 | 51,45 | 0,3013 | 79,28 | -0,2432 |
| 51,63 | 0,3137 | 79,24 | -0,2772 | 51,48 | 0,3054 | 79,22 | -0,2515 | 51,41 | 0,3012 | 79,32 | -0,2432 |
| 51,6 | 0,3137 | 79,27 | -0,2772 | 51,44 | 0,3053 | 79,26 | -0,2516 | 51,38 | 0,3011 | 79,35 | -0,2433 |
| 51,56 | 0,3136 | 79,31 | -0,2773 | 51,41 | 0,3053 | 79,29 | -0,2516 | 51,34 | 0,301 | 79,38 | -0,2434 |
| 51,53 | 0,3136 | 79,34 | -0,2773 | 51,38 | 0,3053 | 79,32 | -0,2517 | 51,31 | 0,3009 | 79,42 | -0,2434 |
| 51,5 | 0,3136 | 79,37 | -0,2774 | 51,34 | 0,3052 | 79,36 | -0,2517 | 51,28 | 0,3008 | 79,45 | -0,2435 |
| 51,46 | 0,3135 | 79,41 | -0,2774 | 51,31 | 0,3052 | 79,39 | -0,2518 | 51,24 | 0,3007 | 79,48 | -0,2436 |
| 51,43 | 0,3135 | 79,44 | -0,2775 | 51,28 | 0,3052 | 79,42 | -0,2518 | 51,21 | 0,3006 | 79,52 | -0,2436 |
| 51,4 | 0,3135 | 79,47 | -0,2775 | 51,24 | 0,3051 | 79,46 | -0,2519 | 51,18 | 0,3006 | 79,55 | -0,2437 |
| 51,36 | 0,3134 | 79,51 | -0,2776 | 51,21 | 0,3051 | 79,49 | -0,2519 | 51,14 | 0,3005 | 79,58 | -0,2438 |
| 51,33 | 0,3134 | 79,54 | -0,2776 | 51,18 | 0,3051 | 79,52 | -0,2519 | 51,11 | 0,3004 | 79,62 | -0,2438 |
| 51,3 | 0,3133 | 79,57 | -0,2777 | 51,14 | 0,305 | 79,56 | -0,252 | 51,08 | 0,3003 | 79,65 | -0,2439 |
| 51,26 | 0,3133 | 79,61 | -0,2777 | 51,11 | 0,305 | 79,59 | -0,252 | 51,04 | 0,3002 | 79,68 | -0,244 |
| 51,23 | 0,3133 | 79,64 | -0,2778 | 51,08 | 0,3049 | 79,62 | -0,2521 | 51,01 | 0,3001 | 79,72 | -0,244 |
| 51,2 | 0,3132 | 79,67 | -0,2778 | 51,04 | 0,3049 | 79,66 | -0,2521 | 50,98 | 0,3 | 79,75 | -0,2441 |
| 51,16 | 0,3132 | 79,71 | -0,2779 | 51,01 | 0,3049 | 79,69 | -0,2522 | 50,94 | 0,2999 | 79,78 | -0,2442 |
| 51,13 | 0,3132 | 79,74 | -0,2779 | 50,98 | 0,3048 | 79,72 | -0,2522 | 50,91 | 0,2998 | 79,82 | -0,2442 |
| 51,1 | 0,3131 | 79,77 | -0,278 | 50,94 | 0,3048 | 79,76 | -0,2522 | 50,88 | 0,2997 | 79,85 | -0,2443 |
| 51,06 | 0,3131 | 79,8 | -0,278 | 50,91 | 0,3048 | 79,79 | -0,2523 | 50,85 | 0,2997 | 79,88 | -0,2443 |
| 51,03 | 0,313 | 79,84 | -0,2781 | 50,88 | 0,3047 | 79,82 | -0,2523 | 50,81 | 0,2996 | 79,92 | -0,2444 |
| 51 | 0,313 | 79,87 | -0,2781 | 50,84 | 0,3047 | 79,86 | -0,2524 | 50,78 | 0,2995 | 79,95 | -0,2445 |
| 50,96 | 0,313 | 79,91 | -0,2782 | 50,81 | 0,3047 | 79,89 | -0,2524 | 50,74 | 0,2994 | 79,98 | -0,2446 |
| 50,93 | 0,3129 | 79,94 | -0,2782 | 50,78 | 0,3046 | 79,92 | -0,2525 | 50,71 | 0,2993 | 80,02 | -0,2446 |
| 50,9 | 0,3129 | 79,97 | -0,2783 | 50,74 | 0,3046 | 79,96 | -0,2525 | 50,68 | 0,2992 | 80,05 | -0,2447 |
| 50,86 | 0,3129 | 80,01 | -0,2783 | 50,71 | 0,3045 | 79,99 | -0,2526 | 50,64 | 0,2992 | 80,08 | -0,2448 |
| 50,83 | 0,3128 | 80,04 | -0,2784 | 50,68 | 0,3045 | 80,02 | -0,2526 | 50,61 | 0,2991 | 80,12 | -0,2448 |
| 50,8 | 0,3128 | 80,07 | -0,2784 | 50,64 | 0,3045 | 80,06 | -0,2526 | 50,58 | 0,299 | 80,15 | -0,2449 |
| 50,76 | 0,3127 | 80,11 | -0,2785 | 50,61 | 0,3044 | 80,09 | -0,2527 | 50,54 | 0,2989 | 80,18 | -0,245 |
| 50,73 | 0,3127 | 80,14 | -0,2785 | 50,58 | 0,3044 | 80,12 | -0,2527 | 50,51 | 0,2988 | 80,22 | -0,245 |
| 50,7 | 0,3127 | 80,17 | -0,2786 | 50,54 | 0,3044 | 80,16 | -0,2528 | 50,48 | 0,2987 | 80,25 | -0,2451 |
| 50,66 | 0,3126 | 80,2 | -0,2786 | 50,51 | 0,3043 | 80,19 | -0,2528 | 50,44 | 0,2986 | 80,28 | -0,2451 |
| 50,63 | 0,3126 | 80,24 | -0,2787 | 50,48 | 0,3043 | 80,22 | -0,2529 | 50,41 | 0,2985 | 80,32 | -0,2452 |
| 50,6 | 0,3126 | 80,27 | -0,2787 | 50,44 | 0,3043 | 80,26 | -0,2529 | 50,38 | 0,2984 | 80,35 | -0,2453 |
| 50,56 | 0,3125 | 80,31 | -0,2788 | 50,41 | 0,3042 | 80,29 | -0,2529 | 50,34 | 0,2983 | 80,38 | -0,2453 |
| 50,53 | 0,3125 | 80,34 | -0,2788 | 50,38 | 0,3042 | 80,32 | -0,253 | 50,31 | 0,2983 | 80,42 | -0,2454 |
| 50,5 | 0,3124 | 80,37 | -0,2789 | 50,34 | 0,3041 | 80,36 | -0,253 | 50,28 | 0,2982 | 80,45 | -0,2455 |
| 50,46 | 0,3124 | 80,41 | -0,2789 | 50,31 | 0,3041 | 80,39 | -0,2531 | 50,24 | 0,2981 | 80,48 | -0,2455 |
| 50,43 | 0,3124 | 80,44 | -0,279 | 50,28 | 0,3041 | 80,42 | -0,2531 | 50,21 | 0,298 | 80,52 | -0,2456 |
| 50,4 | 0,3123 | 80,47 | -0,279 | 50,24 | 0,304 | 80,46 | -0,2532 | 50,18 | 0,2979 | 80,55 | -0,2457 |
| 50,36 | 0,3123 | 80,5 | -0,2791 | 50,21 | 0,304 | 80,49 | -0,2532 | 50,14 | 0,2978 | 80,58 | -0,2457 |
| 50,33 | 0,3123 | 80,54 | -0,2791 | 50,18 | 0,304 | 80,52 | -0,2533 | 50,11 | 0,2977 | 80,62 | -0,2458 |
| 50,3 | 0,3122 | 80,57 | -0,2792 | 50,14 | 0,3039 | 80,56 | -0,2533 | 50,08 | 0,2976 | 80,65 | -0,2459 |
| 50,26 | 0,3122 | 80,6 | -0,2792 | 50,11 | 0,3039 | 80,59 | -0,2533 | 50,04 | 0,2976 | 80,68 | -0,2459 |
| 50,23 | 0,3121 | 80,64 | -0,2793 | 50,08 | 0,3039 | 80,62 | -0,2534 | 50,01 | 0,2975 | 80,72 | -0,246 |
| 50,2 | 0,3121 | 80,67 | -0,2793 | 50,04 | 0,3038 | 80,66 | -0,2534 | 49,98 | 0,2973 | 80,75 | -0,2461 |
| 50,16 | 0,3121 | 80,71 | -0,2794 | 50,01 | 0,3038 | 80,69 | -0,2535 | 49,94 | 0,2971 | 80,78 | -0,2461 |
| 50,13 | 0,312 | 80,74 | -0,2794 | 49,98 | 0,3037 | 80,72 | -0,2535 | 49,91 | 0,297 | 80,82 | -0,2462 |
| 50,1 | 0,312 | 80,77 | -0,2795 | 49,94 | 0,3037 | 80,76 | -0,2536 | 49,88 | 0,2969 | 80,85 | -0,2463 |
| 50,06 | 0,312 | 80,81 | -0,2795 | 49,91 | 0,3037 | 80,79 | -0,2536 | 49,84 | 0,2968 | 80,88 | -0,2464 |
| 50,03 | 0,3119 | 80,84 | -0,2796 | 49,88 | 0,3036 | 80,82 | -0,2536 | 49,81 | 0,2967 | 80,92 | -0,2464 |
| 50 | 0,3119 | 80,87 | -0,2796 | 49,84 | 0,3036 | 80,86 | -0,2537 | 49,78 | 0,2967 | 80,95 | -0,2465 |
| 49,96 | 0,3118 | 80,91 | -0,2797 | 49,81 | 0,3036 | 80,89 | -0,2537 | 49,74 | 0,2966 | 80,98 | -0,2466 |
| 49,93 | 0,3118 | 80,94 | -0,2797 | 49,78 | 0,3035 | 80,92 | -0,2538 | 49,71 | 0,2965 | 81,02 | -0,2466 |
| 49,9 | 0,3118 | 80,97 | -0,2798 | 49,74 | 0,3035 | 80,96 | -0,2538 | 49,68 | 0,2964 | 81,05 | -0,2467 |
| 49,86 | 0,3117 | 81,01 | -0,2798 | 49,71 | 0,3035 | 80,99 | -0,2538 | 49,64 | 0,2963 | 81,08 | -0,2468 |
| 49,83 | 0,3117 | 81,04 | -0,2799 | 49,68 | 0,3034 | 81,02 | -0,2539 | 49,61 | 0,2962 | 81,11 | -0,2468 |
| 49,8 | 0,3117 | 81,07 | -0,2799 | 49,64 | 0,3034 | 81,06 | -0,2539 | 49,58 | 0,2961 | 81,15 | -0,2469 |
| 49,76 | 0,3116 | 81,11 | -0,28 | 49,61 | 0,3033 | 81,09 | -0,254 | 49,54 | 0,296 | 81,18 | -0,2469 |
| 49,73 | 0,3116 | 81,14 | -0,28 | 49,58 | 0,3033 | 81,12 | -0,254 | 49,51 | 0,2959 | 81,22 | -0,247 |
| 49,7 | 0,3115 | 81,17 | -0,2801 | 49,54 | 0,3033 | 81,16 | -0,2541 | 49,48 | 0,2958 | 81,25 | -0,2471 |
| 49,66 | 0,3115 | 81,21 | -0,2801 | 49,51 | 0,3032 | 81,19 | -0,2541 | 49,44 | 0,2957 | 81,28 | -0,2471 |
| 49,63 | 0,3115 | 81,24 | -0,2802 | 49,48 | 0,3032 | 81,22 | -0,2542 | 49,41 | 0,2956 | 81,32 | -0,2472 |
| 49,6 | 0,3114 | 81,27 | -0,2802 | 49,44 | 0,3032 | 81,26 | -0,2542 | 49,37 | 0,2955 | 81,35 | -0,2473 |
| 49,56 | 0,3114 | 81,3 | -0,2803 | 49,41 | 0,3031 | 81,29 | -0,2542 | 49,34 | 0,2953 | 81,38 | -0,2473 |
| 49,53 | 0,3114 | 81,34 | -0,2803 | 49,38 | 0,3031 | 81,32 | -0,2543 | 49,31 | 0,2952 | 81,42 | -0,2474 |
| 49,5 | 0,3113 | 81,37 | -0,2804 | 49,34 | 0,3031 | 81,36 | -0,2543 | 49,28 | 0,2951 | 81,45 | -0,2475 |
| 49,46 | 0,3113 | 81,4 | -0,2804 | 49,31 | 0,303 | 81,39 | -0,2544 | 49,24 | 0,295 | 81,48 | -0,2475 |
| 49,43 | 0,3112 | 81,44 | -0,2805 | 49,28 | 0,303 | 81,42 | -0,2544 | 49,21 | 0,2949 | 81,52 | -0,2476 |
| 49,4 | 0,3112 | 81,47 | -0,2805 | 49,24 | 0,3029 | 81,46 | -0,2544 | 49,17 | 0,2949 | 81,55 | -0,2477 |
| 49,36 | 0,3112 | 81,51 | -0,2806 | 49,21 | 0,3029 | 81,49 | -0,2545 | 49,14 | 0,2948 | 81,58 | -0,2477 |
| 49,33 | 0,3111 | 81,54 | -0,2806 | 49,18 | 0,3029 | 81,52 | -0,2545 | 49,11 | 0,2947 | 81,62 | -0,2478 |
| 49,29 | 0,3111 | 81,57 | -0,2807 | 49,14 | 0,3028 | 81,56 | -0,2546 | 49,08 | 0,2946 | 81,65 | -0,2479 |
| 49,26 | 0,311 | 81,61 | -0,2807 | 49,11 | 0,3028 | 81,59 | -0,2546 | 49,04 | 0,2945 | 81,68 | -0,2479 |
| 49,23 | 0,311 | 81,64 | -0,2808 | 49,08 | 0,3028 | 81,62 | -0,2547 | 49,01 | 0,2944 | 81,72 | -0,248 |
| 49,2 | 0,311 | 81,67 | -0,2808 | 49,04 | 0,3027 | 81,66 | -0,2547 | 48,97 | 0,2943 | 81,75 | -0,248 |
| 49,16 | 0,3109 | 81,71 | -0,2809 | 49,01 | 0,3027 | 81,69 | -0,2547 | 48,94 | 0,2942 | 81,78 | -0,2481 |
| 49,13 | 0,3109 | 81,74 | -0,2809 | 48,98 | 0,3026 | 81,72 | -0,2548 | 48,91 | 0,2941 | 81,82 | -0,2482 |
| 49,1 | 0,3109 | 81,77 | -0,281 | 48,94 | 0,3026 | 81,76 | -0,2548 | 48,87 | 0,294 | 81,85 | -0,2482 |
| 49,06 | 0,3108 | 81,81 | -0,281 | 48,91 | 0,3026 | 81,79 | -0,2549 | 48,84 | 0,2939 | 81,88 | -0,2483 |
| 49,03 | 0,3108 | 81,84 | -0,2811 | 48,88 | 0,3025 | 81,82 | -0,2549 | 48,81 | 0,2938 | 81,92 | -0,2483 |
| 49 | 0,3107 | 81,87 | -0,2811 | 48,84 | 0,3025 | 81,86 | -0,255 | 48,77 | 0,2938 | 81,95 | -0,2484 |
| 48,96 | 0,3107 | 81,91 | -0,2812 | 48,81 | 0,3025 | 81,89 | -0,255 | 48,74 | 0,2937 | 81,98 | -0,2485 |
| 48,93 | 0,3107 | 81,94 | -0,2812 | 48,78 | 0,3024 | 81,92 | -0,255 | 48,71 | 0,2935 | 82,02 | -0,2485 |
| 48,89 | 0,3106 | 81,97 | -0,2813 | 48,74 | 0,3024 | 81,96 | -0,2551 | 48,67 | 0,2934 | 82,05 | -0,2486 |
| 48,86 | 0,3106 | 82,01 | -0,2813 | 48,71 | 0,3023 | 81,99 | -0,2551 | 48,64 | 0,2932 | 82,08 | -0,2487 |
| 48,83 | 0,3106 | 82,04 | -0,2814 | 48,68 | 0,3023 | 82,03 | -0,2552 | 48,61 | 0,2931 | 82,12 | -0,2487 |
| 48,8 | 0,3105 | 82,07 | -0,2814 | 48,64 | 0,3023 | 82,06 | -0,2552 | 48,57 | 0,293 | 82,15 | -0,2488 |
| 48,76 | 0,3105 | 82,11 | -0,2815 | 48,61 | 0,3022 | 82,09 | -0,2553 | 48,54 | 0,293 | 82,18 | -0,2489 |
| 48,73 | 0,3104 | 82,14 | -0,2815 | 48,58 | 0,3022 | 82,12 | -0,2553 | 48,51 | 0,2929 | 82,22 | -0,2489 |
| 48,7 | 0,3104 | 82,17 | -0,2816 | 48,54 | 0,3022 | 82,16 | -0,2553 | 48,47 | 0,2928 | 82,25 | -0,249 |
| 48,66 | 0,3104 | 82,21 | -0,2816 | 48,51 | 0,3021 | 82,19 | -0,2554 | 48,44 | 0,2926 | 82,28 | -0,249 |
| 48,63 | 0,3103 | 82,24 | -0,2817 | 48,48 | 0,3021 | 82,22 | -0,2554 | 48,41 | 0,2925 | 82,32 | -0,2491 |
| 48,59 | 0,3103 | 82,27 | -0,2817 | 48,44 | 0,3021 | 82,26 | -0,2555 | 48,37 | 0,2924 | 82,35 | -0,2492 |
| 48,56 | 0,3102 | 82,31 | -0,2817 | 48,41 | 0,302 | 82,29 | -0,2555 | 48,34 | 0,2923 | 82,38 | -0,2492 |
| 48,53 | 0,3102 | 82,34 | -0,2818 | 48,38 | 0,302 | 82,32 | -0,2556 | 48,31 | 0,2922 | 82,42 | -0,2493 |
| 48,5 | 0,3102 | 82,37 | -0,2818 | 48,34 | 0,3019 | 82,36 | -0,2556 | 48,27 | 0,2921 | 82,45 | -0,2494 |
| 48,46 | 0,3101 | 82,4 | -0,2819 | 48,31 | 0,3019 | 82,39 | -0,2556 | 48,24 | 0,292 | 82,48 | -0,2494 |
| 48,43 | 0,3101 | 82,44 | -0,2819 | 48,28 | 0,3019 | 82,43 | -0,2557 | 48,21 | 0,2918 | 82,52 | -0,2495 |
| 48,4 | 0,3101 | 82,47 | -0,282 | 48,24 | 0,3018 | 82,46 | -0,2557 | 48,17 | 0,2917 | 82,55 | -0,2496 |
| 48,36 | 0,31 | 82,51 | -0,282 | 48,21 | 0,3018 | 82,49 | -0,2558 | 48,14 | 0,2915 | 82,58 | -0,2496 |
| 48,33 | 0,31 | 82,54 | -0,2821 | 48,18 | 0,3017 | 82,52 | -0,2558 | 48,1 | 0,2913 | 82,62 | -0,2497 |
| 48,3 | 0,3099 | 82,57 | -0,2821 | 48,14 | 0,3017 | 82,56 | -0,2559 | 48,07 | 0,2912 | 82,65 | -0,2497 |
| 48,26 | 0,3099 | 82,6 | -0,2822 | 48,11 | 0,3017 | 82,59 | -0,2559 | 48,04 | 0,2911 | 82,68 | -0,2498 |
| 48,23 | 0,3099 | 82,64 | -0,2822 | 48,08 | 0,3016 | 82,63 | -0,2559 | 48,01 | 0,291 | 82,72 | -0,2499 |
| 48,2 | 0,3098 | 82,67 | -0,2823 | 48,04 | 0,3016 | 82,66 | -0,256 | 47,97 | 0,2909 | 82,75 | -0,2499 |
| 48,16 | 0,3098 | 82,71 | -0,2823 | 48,01 | 0,3016 | 82,69 | -0,256 | 47,94 | 0,2909 | 82,78 | -0,25 |
| 48,13 | 0,3098 | 82,74 | -0,2824 | 47,98 | 0,3015 | 82,73 | -0,2561 | 47,91 | 0,2908 | 82,82 | -0,25 |
| 48,1 | 0,3097 | 82,77 | -0,2824 | 47,94 | 0,3015 | 82,76 | -0,2561 | 47,87 | 0,2906 | 82,85 | -0,2501 |
| 48,06 | 0,3097 | 82,81 | -0,2825 | 47,91 | 0,3015 | 82,79 | -0,2562 | 47,84 | 0,2905 | 82,88 | -0,2502 |
| 48,03 | 0,3096 | 82,84 | -0,2826 | 47,88 | 0,3014 | 82,83 | -0,2562 | 47,81 | 0,2903 | 82,92 | -0,2502 |
| 48 | 0,3096 | 82,87 | -0,2826 | 47,84 | 0,3014 | 82,86 | -0,2562 | 47,77 | 0,2902 | 82,95 | -0,2503 |
| 47,96 | 0,3096 | 82,91 | -0,2827 | 47,81 | 0,3013 | 82,89 | -0,2563 | 47,74 | 0,2901 | 82,98 | -0,2504 |
| 47,93 | 0,3095 | 82,94 | -0,2827 | 47,78 | 0,3013 | 82,93 | -0,2563 | 47,7 | 0,29 | 83,02 | -0,2504 |
| 47,89 | 0,3095 | 82,97 | -0,2827 | 47,74 | 0,3013 | 82,96 | -0,2564 | 47,67 | 0,29 | 83,05 | -0,2505 |
| 47,86 | 0,3095 | 83 | -0,2828 | 47,71 | 0,3012 | 82,99 | -0,2564 | 47,64 | 0,2899 | 83,08 | -0,2506 |
| 47,83 | 0,3094 | 83,04 | -0,2829 | 47,68 | 0,3012 | 83,03 | -0,2564 | 47,6 | 0,2898 | 83,12 | -0,2506 |
| 47,8 | 0,3094 | 83,07 | -0,2829 | 47,64 | 0,3012 | 83,06 | -0,2565 | 47,57 | 0,2897 | 83,15 | -0,2507 |
| 47,76 | 0,3093 | 83,11 | -0,283 | 47,61 | 0,3011 | 83,09 | -0,2565 | 47,54 | 0,2896 | 83,18 | -0,2507 |
| 47,73 | 0,3093 | 83,14 | -0,283 | 47,58 | 0,3011 | 83,13 | -0,2566 | 47,5 | 0,2895 | 83,22 | -0,2508 |
| 47,7 | 0,3093 | 83,17 | -0,2831 | 47,54 | 0,301 | 83,16 | -0,2566 | 47,47 | 0,2893 | 83,25 | -0,2508 |
| 47,66 | 0,3092 | 83,21 | -0,2831 | 47,51 | 0,301 | 83,19 | -0,2567 | 47,44 | 0,2892 | 83,28 | -0,2509 |
| 47,63 | 0,3092 | 83,24 | -0,2831 | 47,48 | 0,301 | 83,23 | -0,2567 | 47,4 | 0,289 | 83,32 | -0,251 |
| 47,6 | 0,3092 | 83,27 | -0,2832 | 47,44 | 0,3009 | 83,26 | -0,2567 | 47,37 | 0,2889 | 83,35 | -0,251 |
| 47,56 | 0,3091 | 83,31 | -0,2832 | 47,41 | 0,3009 | 83,29 | -0,2568 | 47,34 | 0,2889 | 83,38 | -0,2511 |
| 47,53 | 0,3091 | 83,34 | -0,2833 | 47,37 | 0,3009 | 83,33 | -0,2568 | 47,3 | 0,2888 | 83,42 | -0,2512 |
| 47,5 | 0,309 | 83,37 | -0,2833 | 47,34 | 0,3008 | 83,36 | -0,2569 | 47,27 | 0,2887 | 83,45 | -0,2512 |
| 47,46 | 0,309 | 83,41 | -0,2834 | 47,31 | 0,3008 | 83,39 | -0,2569 | 47,24 | 0,2886 | 83,48 | -0,2513 |
| 47,43 | 0,309 | 83,44 | -0,2834 | 47,27 | 0,3008 | 83,42 | -0,2569 | 47,2 | 0,2885 | 83,52 | -0,2514 |
| 47,4 | 0,3089 | 83,47 | -0,2835 | 47,24 | 0,3007 | 83,46 | -0,257 | 47,17 | 0,2884 | 83,55 | -0,2514 |
| 47,36 | 0,3089 | 83,51 | -0,2835 | 47,21 | 0,3007 | 83,49 | -0,257 | 47,14 | 0,2883 | 83,58 | -0,2515 |
| 47,33 | 0,3089 | 83,54 | -0,2836 | 47,18 | 0,3006 | 83,53 | -0,2571 | 47,1 | 0,2882 | 83,62 | -0,2515 |
| 47,29 | 0,3088 | 83,57 | -0,2836 | 47,14 | 0,3006 | 83,56 | -0,2571 | 47,07 | 0,2881 | 83,65 | -0,2516 |
| 47,26 | 0,3088 | 83,61 | -0,2837 | 47,11 | 0,3006 | 83,59 | -0,2572 | 47,04 | 0,288 | 83,68 | -0,2517 |
| 47,23 | 0,3087 | 83,64 | -0,2837 | 47,08 | 0,3005 | 83,63 | -0,2572 | 47 | 0,2879 | 83,72 | -0,2517 |
| 47,19 | 0,3087 | 83,67 | -0,2838 | 47,04 | 0,3005 | 83,66 | -0,2572 | 46,97 | 0,2878 | 83,75 | -0,2518 |
| 47,16 | 0,3087 | 83,71 | -0,2838 | 47,01 | 0,3005 | 83,69 | -0,2573 | 46,94 | 0,2877 | 83,78 | -0,2518 |
| 47,13 | 0,3086 | 83,74 | -0,2839 | 46,97 | 0,3004 | 83,72 | -0,2573 | 46,9 | 0,2875 | 83,82 | -0,2519 |
| 47,09 | 0,3086 | 83,77 | -0,2839 | 46,94 | 0,3004 | 83,76 | -0,2574 | 46,87 | 0,2874 | 83,85 | -0,252 |
| 47,06 | 0,3086 | 83,81 | -0,284 | 46,91 | 0,3004 | 83,79 | -0,2574 | 46,84 | 0,2873 | 83,88 | -0,252 |
| 47,03 | 0,3085 | 83,84 | -0,2841 | 46,87 | 0,3003 | 83,83 | -0,2574 | 46,8 | 0,2872 | 83,92 | -0,2521 |
| 46,99 | 0,3085 | 83,87 | -0,2841 | 46,84 | 0,3003 | 83,86 | -0,2575 | 46,77 | 0,2872 | 83,95 | -0,2521 |
| 46,96 | 0,3084 | 83,91 | -0,2842 | 46,81 | 0,3003 | 83,89 | -0,2575 | 46,74 | 0,2871 | 83,98 | -0,2522 |
| 46,93 | 0,3084 | 83,94 | -0,2842 | 46,78 | 0,3002 | 83,93 | -0,2576 | 46,7 | 0,287 | 84,02 | -0,2523 |
| 46,9 | 0,3084 | 83,97 | -0,2843 | 46,74 | 0,3002 | 83,96 | -0,2576 | 46,67 | 0,2869 | 84,05 | -0,2523 |
| 46,86 | 0,3083 | 84,01 | -0,2843 | 46,71 | 0,3001 | 83,99 | -0,2577 | 46,64 | 0,2868 | 84,08 | -0,2524 |
| 46,83 | 0,3083 | 84,04 | -0,2844 | 46,67 | 0,3001 | 84,03 | -0,2577 | 46,6 | 0,2867 | 84,12 | -0,2525 |
| 46,8 | 0,3083 | 84,07 | -0,2844 | 46,64 | 0,3001 | 84,06 | -0,2577 | 46,57 | 0,2866 | 84,15 | -0,2525 |
| 46,76 | 0,3082 | 84,11 | -0,2845 | 46,61 | 0,3 | 84,09 | -0,2578 | 46,54 | 0,2865 | 84,18 | -0,2526 |
| 46,73 | 0,3082 | 84,14 | -0,2845 | 46,58 | 0,3 | 84,13 | -0,2578 | 46,5 | 0,2864 | 84,22 | -0,2526 |
| 46,69 | 0,3081 | 84,17 | -0,2846 | 46,54 | 0,3 | 84,16 | -0,2579 | 46,47 | 0,2863 | 84,25 | -0,2527 |
| 46,66 | 0,3081 | 84,21 | -0,2846 | 46,51 | 0,2999 | 84,19 | -0,2579 | 46,44 | 0,2862 | 84,28 | -0,2528 |
| 46,63 | 0,3081 | 84,24 | -0,2846 | 46,47 | 0,2999 | 84,23 | -0,2579 | 46,4 | 0,2862 | 84,32 | -0,2528 |
| 46,59 | 0,308 | 84,27 | -0,2847 | 46,44 | 0,2999 | 84,26 | -0,258 | 46,37 | 0,2861 | 84,35 | -0,2529 |
| 46,56 | 0,308 | 84,31 | -0,2847 | 46,41 | 0,2998 | 84,29 | -0,258 | 46,34 | 0,286 | 84,38 | -0,2529 |
| 46,53 | 0,3079 | 84,34 | -0,2848 | 46,37 | 0,2998 | 84,33 | -0,2581 | 46,3 | 0,2859 | 84,42 | -0,253 |
| 46,49 | 0,3079 | 84,37 | -0,2848 | 46,34 | 0,2997 | 84,36 | -0,2581 | 46,27 | 0,2858 | 84,45 | -0,2531 |
| 46,46 | 0,3079 | 84,41 | -0,2849 | 46,31 | 0,2997 | 84,39 | -0,2581 | 46,24 | 0,2857 | 84,48 | -0,2531 |
| 46,43 | 0,3078 | 84,44 | -0,2849 | 46,27 | 0,2997 | 84,43 | -0,2582 | 46,2 | 0,2856 | 84,52 | -0,2532 |
| 46,39 | 0,3078 | 84,47 | -0,285 | 46,24 | 0,2996 | 84,46 | -0,2582 | 46,17 | 0,2855 | 84,55 | -0,2532 |
| 46,36 | 0,3078 | 84,51 | -0,285 | 46,21 | 0,2996 | 84,49 | -0,2583 | 46,14 | 0,2853 | 84,58 | -0,2533 |
| 46,33 | 0,3077 | 84,54 | -0,2851 | 46,17 | 0,2996 | 84,53 | -0,2583 | 46,1 | 0,2851 | 84,62 | -0,2533 |
| 46,29 | 0,3077 | 84,57 | -0,2851 | 46,14 | 0,2995 | 84,56 | -0,2583 | 46,07 | 0,285 | 84,65 | -0,2534 |
| 46,26 | 0,3077 | 84,61 | -0,2852 | 46,11 | 0,2995 | 84,59 | -0,2584 | 46,04 | 0,2849 | 84,68 | -0,2535 |
| 46,23 | 0,3076 | 84,64 | -0,2852 | 46,08 | 0,2995 | 84,63 | -0,2584 | 46 | 0,2849 | 84,72 | -0,2535 |
| 46,19 | 0,3076 | 84,67 | -0,2853 | 46,04 | 0,2994 | 84,66 | -0,2585 | 45,97 | 0,2848 | 84,75 | -0,2536 |
| 46,16 | 0,3075 | 84,71 | -0,2853 | 46,01 | 0,2994 | 84,69 | -0,2585 | 45,94 | 0,2847 | 84,78 | -0,2536 |
| 46,13 | 0,3075 | 84,74 | -0,2854 | 45,98 | 0,2993 | 84,73 | -0,2585 | 45,9 | 0,2846 | 84,82 | -0,2537 |
| 46,09 | 0,3075 | 84,77 | -0,2854 | 45,94 | 0,2993 | 84,76 | -0,2586 | 45,87 | 0,2845 | 84,85 | -0,2538 |
| 46,06 | 0,3074 | 84,81 | -0,2854 | 45,91 | 0,2993 | 84,79 | -0,2586 | 45,84 | 0,2844 | 84,88 | -0,2538 |
| 46,03 | 0,3074 | 84,84 | -0,2855 | 45,87 | 0,2992 | 84,83 | -0,2587 | 45,8 | 0,2843 | 84,92 | -0,2539 |
| 45,99 | 0,3074 | 84,87 | -0,2855 | 45,84 | 0,2992 | 84,86 | -0,2587 | 45,77 | 0,2842 | 84,95 | -0,2539 |
| 45,96 | 0,3073 | 84,91 | -0,2856 | 45,81 | 0,2992 | 84,89 | -0,2587 | 45,74 | 0,2841 | 84,98 | -0,254 |
| 45,93 | 0,3073 | 84,94 | -0,2856 | 45,78 | 0,2991 | 84,93 | -0,2588 | 45,7 | 0,2841 | 85,02 | -0,254 |
| 45,89 | 0,3073 | 84,97 | -0,2857 | 45,74 | 0,2991 | 84,96 | -0,2588 | 45,67 | 0,284 | 85,05 | -0,2541 |
| 45,86 | 0,3072 | 85,01 | -0,2857 | 45,71 | 0,2991 | 84,99 | -0,2589 | 45,64 | 0,2839 | 85,08 | -0,2542 |
| 45,83 | 0,3072 | 85,04 | -0,2858 | 45,68 | 0,299 | 85,03 | -0,2589 | 45,6 | 0,2838 | 85,12 | -0,2542 |
| 45,79 | 0,3071 | 85,07 | -0,2858 | 45,64 | 0,299 | 85,06 | -0,2589 | 45,57 | 0,2836 | 85,15 | -0,2543 |
| 45,76 | 0,3071 | 85,11 | -0,2859 | 45,61 | 0,299 | 85,09 | -0,259 | 45,53 | 0,2835 | 85,18 | -0,2543 |
| 45,73 | 0,3071 | 85,14 | -0,2859 | 45,57 | 0,2989 | 85,13 | -0,259 | 45,5 | 0,2834 | 85,22 | -0,2544 |
| 45,69 | 0,307 | 85,17 | -0,286 | 45,54 | 0,2989 | 85,16 | -0,2591 | 45,47 | 0,2833 | 85,25 | -0,2545 |
| 45,66 | 0,307 | 85,21 | -0,286 | 45,51 | 0,2988 | 85,19 | -0,2591 | 45,43 | 0,2832 | 85,29 | -0,2545 |
| 45,63 | 0,307 | 85,24 | -0,2861 | 45,48 | 0,2988 | 85,23 | -0,2591 | 45,4 | 0,2831 | 85,32 | -0,2546 |
| 45,59 | 0,3069 | 85,27 | -0,2861 | 45,44 | 0,2988 | 85,26 | -0,2592 | 45,37 | 0,283 | 85,35 | -0,2546 |
| 45,56 | 0,3069 | 85,31 | -0,2861 | 45,41 | 0,2987 | 85,29 | -0,2592 | 45,33 | 0,2829 | 85,38 | -0,2547 |
| 45,53 | 0,3069 | 85,34 | -0,2862 | 45,38 | 0,2987 | 85,33 | -0,2593 | 45,3 | 0,2828 | 85,42 | -0,2548 |
| 45,49 | 0,3068 | 85,37 | -0,2862 | 45,34 | 0,2987 | 85,36 | -0,2593 | 45,27 | 0,2827 | 85,45 | -0,2548 |
| 45,46 | 0,3068 | 85,41 | -0,2863 | 45,31 | 0,2986 | 85,39 | -0,2593 | 45,24 | 0,2826 | 85,48 | -0,2549 |
| 45,43 | 0,3067 | 85,44 | -0,2863 | 45,28 | 0,2986 | 85,43 | -0,2594 | 45,2 | 0,2825 | 85,52 | -0,2549 |
| 45,39 | 0,3067 | 85,47 | -0,2864 | 45,24 | 0,2986 | 85,46 | -0,2594 | 45,17 | 0,2824 | 85,55 | -0,255 |
| 45,36 | 0,3067 | 85,51 | -0,2864 | 45,21 | 0,2985 | 85,49 | -0,2594 | 45,13 | 0,2822 | 85,58 | -0,255 |
| 45,33 | 0,3066 | 85,54 | -0,2865 | 45,18 | 0,2985 | 85,53 | -0,2595 | 45,1 | 0,282 | 85,62 | -0,2551 |
| 45,29 | 0,3066 | 85,58 | -0,2865 | 45,14 | 0,2985 | 85,56 | -0,2595 | 45,07 | 0,282 | 85,65 | -0,2552 |
| 45,26 | 0,3066 | 85,61 | -0,2866 | 45,11 | 0,2984 | 85,59 | -0,2596 | 45,03 | 0,2819 | 85,68 | -0,2552 |
| 45,23 | 0,3065 | 85,64 | -0,2866 | 45,07 | 0,2984 | 85,63 | -0,2596 | 45 | 0,2818 | 85,72 | -0,2553 |
| 45,19 | 0,3065 | 85,67 | -0,2866 | 45,04 | 0,2983 | 85,66 | -0,2596 | 44,97 | 0,2817 | 85,75 | -0,2553 |
| 45,16 | 0,3065 | 85,71 | -0,2867 | 45,01 | 0,2983 | 85,69 | -0,2597 | 44,94 | 0,2816 | 85,78 | -0,2554 |
| 45,13 | 0,3064 | 85,74 | -0,2867 | 44,98 | 0,2983 | 85,73 | -0,2597 | 44,9 | 0,2815 | 85,82 | -0,2554 |
| 45,09 | 0,3064 | 85,77 | -0,2868 | 44,94 | 0,2982 | 85,76 | -0,2598 | 44,87 | 0,2814 | 85,85 | -0,2555 |
| 45,06 | 0,3064 | 85,81 | -0,2868 | 44,91 | 0,2982 | 85,79 | -0,2598 | 44,83 | 0,2813 | 85,89 | -0,2556 |
| 45,03 | 0,3063 | 85,84 | -0,2869 | 44,87 | 0,2982 | 85,83 | -0,2598 | 44,8 | 0,2812 | 85,92 | -0,2556 |
| 44,99 | 0,3063 | 85,87 | -0,2869 | 44,84 | 0,2982 | 85,86 | -0,2599 | 44,77 | 0,2812 | 85,95 | -0,2557 |
| 44,96 | 0,3062 | 85,91 | -0,287 | 44,81 | 0,2981 | 85,89 | -0,2599 | 44,74 | 0,2811 | 85,98 | -0,2557 |
| 44,93 | 0,3062 | 85,94 | -0,287 | 44,77 | 0,2981 | 85,93 | -0,26 | 44,7 | 0,281 | 86,02 | -0,2558 |
| 44,89 | 0,3062 | 85,97 | -0,2871 | 44,74 | 0,2981 | 85,96 | -0,26 | 44,67 | 0,2809 | 86,05 | -0,2558 |
| 44,86 | 0,3061 | 86,01 | -0,2871 | 44,71 | 0,298 | 85,99 | -0,26 | 44,63 | 0,2808 | 86,09 | -0,2559 |
| 44,83 | 0,3061 | 86,04 | -0,2871 | 44,68 | 0,298 | 86,03 | -0,2601 | 44,6 | 0,2807 | 86,12 | -0,256 |
| 44,79 | 0,3061 | 86,07 | -0,2872 | 44,64 | 0,2979 | 86,06 | -0,2601 | 44,57 | 0,2806 | 86,15 | -0,256 |
| 44,76 | 0,306 | 86,11 | -0,2872 | 44,61 | 0,2979 | 86,1 | -0,2601 | 44,53 | 0,2805 | 86,19 | -0,2561 |
| 44,73 | 0,306 | 86,14 | -0,2873 | 44,57 | 0,2979 | 86,13 | -0,2602 | 44,5 | 0,2804 | 86,22 | -0,2561 |
| 44,69 | 0,306 | 86,17 | -0,2873 | 44,54 | 0,2978 | 86,16 | -0,2602 | 44,47 | 0,2803 | 86,25 | -0,2562 |
| 44,66 | 0,3059 | 86,21 | -0,2874 | 44,51 | 0,2978 | 86,19 | -0,2603 | 44,43 | 0,2803 | 86,28 | -0,2562 |
| 44,63 | 0,3059 | 86,24 | -0,2874 | 44,47 | 0,2978 | 86,23 | -0,2603 | 44,4 | 0,2802 | 86,32 | -0,2563 |
| 44,59 | 0,3058 | 86,28 | -0,2875 | 44,44 | 0,2977 | 86,26 | -0,2603 | 44,37 | 0,2801 | 86,35 | -0,2563 |
| 44,56 | 0,3058 | 86,31 | -0,2875 | 44,41 | 0,2977 | 86,29 | -0,2604 | 44,33 | 0,28 | 86,39 | -0,2564 |
| 44,53 | 0,3058 | 86,34 | -0,2876 | 44,37 | 0,2977 | 86,33 | -0,2604 | 44,3 | 0,2799 | 86,42 | -0,2565 |
| 44,49 | 0,3058 | 86,37 | -0,2876 | 44,34 | 0,2976 | 86,36 | -0,2605 | 44,27 | 0,2798 | 86,45 | -0,2565 |
| 44,46 | 0,3057 | 86,41 | -0,2876 | 44,31 | 0,2976 | 86,39 | -0,2605 | 44,23 | 0,2797 | 86,49 | -0,2566 |
| 44,43 | 0,3057 | 86,44 | -0,2877 | 44,27 | 0,2976 | 86,43 | -0,2605 | 44,2 | 0,2796 | 86,52 | -0,2566 |
| 44,39 | 0,3056 | 86,47 | -0,2877 | 44,24 | 0,2975 | 86,46 | -0,2606 | 44,17 | 0,2795 | 86,55 | -0,2567 |
| 44,36 | 0,3056 | 86,51 | -0,2878 | 44,21 | 0,2975 | 86,5 | -0,2606 | 44,13 | 0,2794 | 86,58 | -0,2567 |
| 44,33 | 0,3056 | 86,54 | -0,2878 | 44,17 | 0,2975 | 86,53 | -0,2606 | 44,1 | 0,2793 | 86,62 | -0,2568 |
| 44,29 | 0,3055 | 86,58 | -0,2879 | 44,14 | 0,2974 | 86,56 | -0,2607 | 44,07 | 0,2792 | 86,65 | -0,2568 |
| 44,26 | 0,3055 | 86,61 | -0,2879 | 44,11 | 0,2974 | 86,6 | -0,2607 | 44,03 | 0,2791 | 86,69 | -0,2569 |
| 44,23 | 0,3055 | 86,64 | -0,288 | 44,07 | 0,2974 | 86,63 | -0,2608 | 44 | 0,279 | 86,72 | -0,257 |
| 44,2 | 0,3054 | 86,67 | -0,288 | 44,04 | 0,2973 | 86,66 | -0,2608 | 43,97 | 0,2789 | 86,75 | -0,257 |
| 44,16 | 0,3054 | 86,71 | -0,288 | 44,01 | 0,2973 | 86,69 | -0,2608 | 43,93 | 0,2788 | 86,79 | -0,2571 |
| 44,13 | 0,3054 | 86,74 | -0,2881 | 43,97 | 0,2973 | 86,73 | -0,2609 | 43,9 | 0,2787 | 86,82 | -0,2571 |
| 44,09 | 0,3053 | 86,78 | -0,2881 | 43,94 | 0,2972 | 86,76 | -0,2609 | 43,87 | 0,2787 | 86,85 | -0,2572 |
| 44,06 | 0,3053 | 86,81 | -0,2882 | 43,91 | 0,2972 | 86,79 | -0,2609 | 43,83 | 0,2786 | 86,89 | -0,2572 |
| 44,03 | 0,3052 | 86,84 | -0,2882 | 43,87 | 0,2972 | 86,83 | -0,261 | 43,8 | 0,2785 | 86,92 | -0,2573 |
| 43,99 | 0,3052 | 86,87 | -0,2883 | 43,84 | 0,2971 | 86,86 | -0,261 | 43,77 | 0,2784 | 86,95 | -0,2573 |
| 43,96 | 0,3052 | 86,91 | -0,2883 | 43,81 | 0,2971 | 86,9 | -0,2611 | 43,73 | 0,2782 | 86,99 | -0,2574 |
| 43,93 | 0,3051 | 86,94 | -0,2884 | 43,77 | 0,2971 | 86,93 | -0,2611 | 43,7 | 0,2782 | 87,02 | -0,2575 |
| 43,89 | 0,3051 | 86,98 | -0,2884 | 43,74 | 0,297 | 86,96 | -0,2611 | 43,67 | 0,2781 | 87,05 | -0,2575 |
| 43,86 | 0,3051 | 87,01 | -0,2885 | 43,71 | 0,297 | 86,99 | -0,2612 | 43,63 | 0,278 | 87,09 | -0,2576 |
| 43,83 | 0,305 | 87,04 | -0,2885 | 43,67 | 0,297 | 87,03 | -0,2612 | 43,6 | 0,2779 | 87,12 | -0,2576 |
| 43,8 | 0,305 | 87,07 | -0,2885 | 43,64 | 0,2969 | 87,06 | -0,2612 | 43,57 | 0,2778 | 87,15 | -0,2577 |
| 43,76 | 0,305 | 87,11 | -0,2886 | 43,61 | 0,2969 | 87,1 | -0,2613 | 43,53 | 0,2777 | 87,19 | -0,2577 |
| 43,73 | 0,3049 | 87,14 | -0,2886 | 43,57 | 0,2969 | 87,13 | -0,2613 | 43,5 | 0,2776 | 87,22 | -0,2578 |
| 43,69 | 0,3049 | 87,17 | -0,2887 | 43,54 | 0,2968 | 87,16 | -0,2613 | 43,47 | 0,2775 | 87,25 | -0,2578 |
| 43,66 | 0,3049 | 87,21 | -0,2887 | 43,51 | 0,2968 | 87,2 | -0,2614 | 43,43 | 0,2774 | 87,29 | -0,2579 |
| 43,63 | 0,3048 | 87,24 | -0,2888 | 43,47 | 0,2968 | 87,23 | -0,2614 | 43,4 | 0,2773 | 87,32 | -0,258 |
| 43,59 | 0,3048 | 87,27 | -0,2888 | 43,44 | 0,2967 | 87,26 | -0,2615 | 43,37 | 0,2772 | 87,35 | -0,258 |
| 43,56 | 0,3048 | 87,31 | -0,2889 | 43,41 | 0,2967 | 87,3 | -0,2615 | 43,33 | 0,2771 | 87,39 | -0,2581 |
| 43,53 | 0,3047 | 87,34 | -0,2889 | 43,37 | 0,2967 | 87,33 | -0,2615 | 43,3 | 0,277 | 87,42 | -0,2581 |
| 43,49 | 0,3047 | 87,37 | -0,289 | 43,34 | 0,2966 | 87,36 | -0,2616 | 43,27 | 0,277 | 87,45 | -0,2582 |
| 43,46 | 0,3047 | 87,41 | -0,289 | 43,31 | 0,2966 | 87,39 | -0,2616 | 43,23 | 0,2769 | 87,49 | -0,2582 |
| 43,43 | 0,3046 | 87,44 | -0,289 | 43,27 | 0,2966 | 87,43 | -0,2616 | 43,2 | 0,2768 | 87,52 | -0,2583 |
| 43,39 | 0,3046 | 87,47 | -0,2891 | 43,24 | 0,2965 | 87,46 | -0,2617 | 43,17 | 0,2767 | 87,55 | -0,2583 |
| 43,36 | 0,3045 | 87,51 | -0,2891 | 43,2 | 0,2965 | 87,5 | -0,2617 | 43,13 | 0,2766 | 87,59 | -0,2584 |
| 43,33 | 0,3045 | 87,54 | -0,2892 | 43,17 | 0,2965 | 87,53 | -0,2617 | 43,1 | 0,2765 | 87,62 | -0,2584 |
| 43,29 | 0,3045 | 87,58 | -0,2892 | 43,14 | 0,2964 | 87,56 | -0,2618 | 43,07 | 0,2764 | 87,65 | -0,2585 |
| 43,26 | 0,3044 | 87,61 | -0,2893 | 43,11 | 0,2964 | 87,6 | -0,2618 | 43,03 | 0,2763 | 87,69 | -0,2586 |
| 43,23 | 0,3044 | 87,64 | -0,2893 | 43,07 | 0,2964 | 87,63 | -0,2619 | 43 | 0,2762 | 87,72 | -0,2586 |
| 43,19 | 0,3044 | 87,67 | -0,2893 | 43,04 | 0,2963 | 87,66 | -0,2619 | 42,97 | 0,2761 | 87,75 | -0,2587 |
| 43,16 | 0,3043 | 87,71 | -0,2894 | 43,01 | 0,2963 | 87,7 | -0,2619 | 42,93 | 0,276 | 87,79 | -0,2587 |
| 43,13 | 0,3043 | 87,74 | -0,2894 | 42,97 | 0,2963 | 87,73 | -0,262 | 42,9 | 0,2759 | 87,82 | -0,2588 |
| 43,09 | 0,3043 | 87,77 | -0,2895 | 42,94 | 0,2962 | 87,76 | -0,262 | 42,86 | 0,2758 | 87,85 | -0,2588 |
| 43,06 | 0,3042 | 87,81 | -0,2895 | 42,91 | 0,2962 | 87,8 | -0,262 | 42,83 | 0,2757 | 87,89 | -0,2589 |
| 43,03 | 0,3042 | 87,84 | -0,2896 | 42,87 | 0,2962 | 87,83 | -0,2621 | 42,8 | 0,2757 | 87,92 | -0,2589 |
| 42,99 | 0,3042 | 87,87 | -0,2896 | 42,84 | 0,2961 | 87,86 | -0,2621 | 42,77 | 0,2756 | 87,95 | -0,259 |
| 42,96 | 0,3041 | 87,91 | -0,2897 | 42,81 | 0,2961 | 87,9 | -0,2621 | 42,73 | 0,2755 | 87,99 | -0,259 |
| 42,93 | 0,3041 | 87,94 | -0,2897 | 42,77 | 0,2961 | 87,93 | -0,2622 | 42,7 | 0,2754 | 88,02 | -0,2591 |
| 42,89 | 0,3041 | 87,97 | -0,2897 | 42,74 | 0,296 | 87,96 | -0,2622 | 42,67 | 0,2753 | 88,05 | -0,2591 |
| 42,86 | 0,304 | 88,01 | -0,2898 | 42,7 | 0,296 | 88 | -0,2622 | 42,63 | 0,2752 | 88,09 | -0,2592 |
| 42,83 | 0,304 | 88,04 | -0,2898 | 42,67 | 0,2959 | 88,03 | -0,2623 | 42,6 | 0,2751 | 88,12 | -0,2592 |
| 42,79 | 0,304 | 88,07 | -0,2899 | 42,64 | 0,2959 | 88,06 | -0,2623 | 42,57 | 0,275 | 88,15 | -0,2593 |
| 42,76 | 0,3039 | 88,11 | -0,2899 | 42,61 | 0,2959 | 88,1 | -0,2623 | 42,53 | 0,2749 | 88,19 | -0,2593 |
| 42,73 | 0,3039 | 88,14 | -0,29 | 42,57 | 0,2959 | 88,13 | -0,2624 | 42,5 | 0,2748 | 88,22 | -0,2594 |
| 42,69 | 0,3039 | 88,17 | -0,29 | 42,54 | 0,2958 | 88,16 | -0,2624 | 42,47 | 0,2747 | 88,25 | -0,2595 |
| 42,66 | 0,3038 | 88,21 | -0,29 | 42,51 | 0,2958 | 88,2 | -0,2624 | 42,43 | 0,2746 | 88,29 | -0,2595 |
| 42,63 | 0,3038 | 88,24 | -0,2901 | 42,47 | 0,2958 | 88,23 | -0,2625 | 42,4 | 0,2746 | 88,32 | -0,2596 |
| 42,59 | 0,3038 | 88,27 | -0,2901 | 42,44 | 0,2957 | 88,26 | -0,2625 | 42,36 | 0,2745 | 88,35 | -0,2596 |
| 42,56 | 0,3037 | 88,31 | -0,2902 | 42,41 | 0,2957 | 88,3 | -0,2626 | 42,33 | 0,2744 | 88,39 | -0,2597 |
| 42,53 | 0,3037 | 88,34 | -0,2902 | 42,37 | 0,2957 | 88,33 | -0,2626 | 42,3 | 0,2743 | 88,42 | -0,2597 |
| 42,49 | 0,3037 | 88,37 | -0,2903 | 42,34 | 0,2956 | 88,36 | -0,2626 | 42,26 | 0,2742 | 88,45 | -0,2598 |
| 42,46 | 0,3036 | 88,41 | -0,2903 | 42,31 | 0,2956 | 88,4 | -0,2627 | 42,23 | 0,2741 | 88,49 | -0,2598 |
| 42,43 | 0,3036 | 88,44 | -0,2904 | 42,27 | 0,2956 | 88,43 | -0,2627 | 42,2 | 0,274 | 88,52 | -0,2599 |
| 42,39 | 0,3036 | 88,47 | -0,2904 | 42,24 | 0,2955 | 88,46 | -0,2627 | 42,16 | 0,2739 | 88,55 | -0,2599 |
| 42,36 | 0,3035 | 88,51 | -0,2904 | 42,2 | 0,2955 | 88,5 | -0,2628 | 42,13 | 0,2738 | 88,59 | -0,26 |
| 42,33 | 0,3035 | 88,54 | -0,2905 | 42,17 | 0,2955 | 88,53 | -0,2628 | 42,1 | 0,2738 | 88,62 | -0,26 |
| 42,29 | 0,3035 | 88,58 | -0,2905 | 42,14 | 0,2954 | 88,56 | -0,2628 | 42,06 | 0,2737 | 88,65 | -0,2601 |
| 42,26 | 0,3034 | 88,61 | -0,2906 | 42,11 | 0,2954 | 88,6 | -0,2629 | 42,03 | 0,2736 | 88,69 | -0,2601 |
| 42,23 | 0,3034 | 88,64 | -0,2906 | 42,07 | 0,2953 | 88,63 | -0,2629 | 42 | 0,2735 | 88,72 | -0,2602 |
| 42,19 | 0,3034 | 88,68 | -0,2906 | 42,04 | 0,2953 | 88,66 | -0,2629 | 41,96 | 0,2734 | 88,75 | -0,2602 |
| 42,16 | 0,3033 | 88,71 | -0,2907 | 42,01 | 0,2953 | 88,7 | -0,263 | 41,93 | 0,2733 | 88,79 | -0,2603 |
| 42,13 | 0,3033 | 88,74 | -0,2907 | 41,97 | 0,2953 | 88,73 | -0,263 | 41,9 | 0,2732 | 88,82 | -0,2603 |
| 42,09 | 0,3032 | 88,78 | -0,2908 | 41,94 | 0,2952 | 88,76 | -0,263 | 41,86 | 0,2731 | 88,85 | -0,2604 |
| 42,06 | 0,3032 | 88,81 | -0,2908 | 41,9 | 0,2952 | 88,8 | -0,2631 | 41,83 | 0,273 | 88,89 | -0,2604 |
| 42,03 | 0,3032 | 88,84 | -0,2909 | 41,87 | 0,2951 | 88,83 | -0,2631 | 41,8 | 0,2729 | 88,92 | -0,2605 |
| 41,99 | 0,3031 | 88,88 | -0,2909 | 41,84 | 0,2951 | 88,86 | -0,2631 | 41,76 | 0,2728 | 88,95 | -0,2605 |
| 41,96 | 0,3031 | 88,91 | -0,2909 | 41,81 | 0,2951 | 88,9 | -0,2632 | 41,73 | 0,2727 | 88,99 | -0,2606 |
| 41,93 | 0,3031 | 88,94 | -0,291 | 41,77 | 0,295 | 88,93 | -0,2632 | 41,7 | 0,2726 | 89,02 | -0,2606 |
| 41,89 | 0,303 | 88,98 | -0,291 | 41,74 | 0,295 | 88,96 | -0,2632 | 41,66 | 0,2725 | 89,05 | -0,2607 |
| 41,86 | 0,303 | 89,01 | -0,2911 | 41,71 | 0,295 | 89 | -0,2633 | 41,63 | 0,2724 | 89,09 | -0,2607 |
| 41,83 | 0,303 | 89,04 | -0,2911 | 41,67 | 0,295 | 89,03 | -0,2633 | 41,6 | 0,2723 | 89,12 | -0,2608 |
| 41,79 | 0,3029 | 89,07 | -0,2912 | 41,64 | 0,2949 | 89,06 | -0,2633 | 41,56 | 0,2723 | 89,15 | -0,2608 |
| 41,76 | 0,3029 | 89,11 | -0,2912 | 41,61 | 0,2949 | 89,1 | -0,2634 | 41,53 | 0,2722 | 89,19 | -0,2609 |
| 41,72 | 0,3029 | 89,14 | -0,2913 | 41,57 | 0,2948 | 89,13 | -0,2634 | 41,5 | 0,2721 | 89,22 | -0,2609 |
| 41,69 | 0,3028 | 89,18 | -0,2913 | 41,54 | 0,2948 | 89,16 | -0,2634 | 41,46 | 0,272 | 89,25 | -0,261 |
| 41,66 | 0,3028 | 89,21 | -0,2913 | 41,51 | 0,2948 | 89,2 | -0,2635 | 41,43 | 0,2719 | 89,29 | -0,261 |
| 41,63 | 0,3028 | 89,24 | -0,2914 | 41,47 | 0,2948 | 89,23 | -0,2635 | 41,4 | 0,2718 | 89,32 | -0,2611 |
| 41,59 | 0,3027 | 89,28 | -0,2914 | 41,44 | 0,2947 | 89,26 | -0,2635 | 41,36 | 0,2717 | 89,35 | -0,2611 |
| 41,56 | 0,3027 | 89,31 | -0,2915 | 41,41 | 0,2947 | 89,3 | -0,2636 | 41,33 | 0,2716 | 89,39 | -0,2612 |
| 41,53 | 0,3027 | 89,34 | -0,2915 | 41,37 | 0,2947 | 89,33 | -0,2636 | 41,3 | 0,2715 | 89,42 | -0,2612 |
| 41,49 | 0,3026 | 89,38 | -0,2915 | 41,34 | 0,2946 | 89,36 | -0,2636 | 41,26 | 0,2714 | 89,45 | -0,2613 |
| 41,46 | 0,3026 | 89,41 | -0,2916 | 41,31 | 0,2946 | 89,4 | -0,2637 | 41,23 | 0,2713 | 89,49 | -0,2613 |
| 41,43 | 0,3026 | 89,44 | -0,2916 | 41,27 | 0,2946 | 89,43 | -0,2637 | 41,2 | 0,2712 | 89,52 | -0,2614 |
| 41,39 | 0,3025 | 89,48 | -0,2917 | 41,24 | 0,2945 | 89,46 | -0,2637 | 41,16 | 0,2712 | 89,56 | -0,2614 |
| 41,36 | 0,3025 | 89,51 | -0,2917 | 41,21 | 0,2945 | 89,5 | -0,2638 | 41,13 | 0,2711 | 89,59 | -0,2615 |
| 41,33 | 0,3025 | 89,54 | -0,2918 | 41,17 | 0,2945 | 89,53 | -0,2638 | 41,1 | 0,271 | 89,62 | -0,2616 |
| 41,29 | 0,3024 | 89,58 | -0,2918 | 41,14 | 0,2944 | 89,56 | -0,2638 | 41,06 | 0,2709 | 89,65 | -0,2616 |
| 41,26 | 0,3024 | 89,61 | -0,2919 | 41,1 | 0,2944 | 89,6 | -0,2639 | 41,03 | 0,2708 | 89,69 | -0,2617 |
| 41,23 | 0,3024 | 89,64 | -0,2919 | 41,07 | 0,2943 | 89,63 | -0,2639 | 41 | 0,2707 | 89,72 | -0,2617 |
| 41,19 | 0,3023 | 89,68 | -0,2919 | 41,04 | 0,2943 | 89,66 | -0,2639 | 40,96 | 0,2706 | 89,75 | -0,2618 |
| 41,16 | 0,3023 | 89,71 | -0,292 | 41,01 | 0,2943 | 89,7 | -0,264 | 40,93 | 0,2705 | 89,79 | -0,2618 |
| 41,13 | 0,3023 | 89,74 | -0,292 | 40,97 | 0,2942 | 89,73 | -0,264 | 40,9 | 0,2704 | 89,82 | -0,2619 |
| 41,09 | 0,3022 | 89,78 | -0,2921 | 40,94 | 0,2942 | 89,76 | -0,264 | 40,86 | 0,2704 | 89,85 | -0,2619 |
| 41,06 | 0,3022 | 89,81 | -0,2921 | 40,91 | 0,2942 | 89,8 | -0,2641 | 40,83 | 0,2703 | 89,89 | -0,262 |
| 41,03 | 0,3022 | 89,84 | -0,2922 | 40,87 | 0,2941 | 89,83 | -0,2641 | 40,79 | 0,2702 | 89,92 | -0,262 |
| 40,99 | 0,3021 | 89,88 | -0,2922 | 40,84 | 0,2941 | 89,86 | -0,2641 | 40,76 | 0,2701 | 89,95 | -0,2621 |
| 40,96 | 0,3021 | 89,91 | -0,2922 | 40,81 | 0,2941 | 89,9 | -0,2642 | 40,73 | 0,27 | 89,99 | -0,2621 |
| 40,92 | 0,3021 | 89,94 | -0,2923 | 40,77 | 0,294 | 89,93 | -0,2642 | 40,7 | 0,2699 | 90,02 | -0,2622 |
| 40,89 | 0,302 | 89,98 | -0,2923 | 40,74 | 0,294 | 89,96 | -0,2642 | 40,66 | 0,2698 | 90,06 | -0,2622 |
| 40,86 | 0,302 | 90,01 | -0,2924 | 40,71 | 0,294 | 90 | -0,2643 | 40,63 | 0,2697 | 90,09 | -0,2623 |
| 40,83 | 0,302 | 90,04 | -0,2924 | 40,67 | 0,2939 | 90,03 | -0,2643 | 40,6 | 0,2696 | 90,12 | -0,2623 |
| 40,79 | 0,3019 | 90,08 | -0,2924 | 40,64 | 0,2939 | 90,06 | -0,2643 | 40,56 | 0,2695 | 90,15 | -0,2624 |
| 40,76 | 0,3019 | 90,11 | -0,2925 | 40,61 | 0,2939 | 90,1 | -0,2644 | 40,53 | 0,2694 | 90,19 | -0,2624 |
| 40,72 | 0,3019 | 90,14 | -0,2925 | 40,57 | 0,2938 | 90,13 | -0,2644 | 40,5 | 0,2693 | 90,22 | -0,2625 |
| 40,69 | 0,3018 | 90,18 | -0,2926 | 40,54 | 0,2938 | 90,16 | -0,2644 | 40,46 | 0,2692 | 90,25 | -0,2625 |
| 40,66 | 0,3018 | 90,21 | -0,2926 | 40,51 | 0,2938 | 90,2 | -0,2645 | 40,43 | 0,2692 | 90,29 | -0,2626 |
| 40,62 | 0,3018 | 90,24 | -0,2927 | 40,47 | 0,2937 | 90,23 | -0,2645 | 40,4 | 0,2691 | 90,32 | -0,2626 |
| 40,59 | 0,3017 | 90,28 | -0,2927 | 40,44 | 0,2937 | 90,26 | -0,2645 | 40,36 | 0,269 | 90,36 | -0,2627 |
| 40,56 | 0,3017 | 90,31 | -0,2927 | 40,4 | 0,2937 | 90,3 | -0,2646 | 40,33 | 0,2689 | 90,39 | -0,2627 |
| 40,53 | 0,3017 | 90,34 | -0,2928 | 40,37 | 0,2936 | 90,33 | -0,2646 | 40,29 | 0,2688 | 90,42 | -0,2628 |
| 40,49 | 0,3016 | 90,38 | -0,2928 | 40,34 | 0,2936 | 90,36 | -0,2646 | 40,26 | 0,2687 | 90,45 | -0,2628 |
| 40,46 | 0,3016 | 90,41 | -0,2929 | 40,31 | 0,2935 | 90,4 | -0,2647 | 40,23 | 0,2686 | 90,49 | -0,2629 |
| 40,43 | 0,3016 | 90,44 | -0,2929 | 40,27 | 0,2935 | 90,43 | -0,2647 | 40,19 | 0,2685 | 90,52 | -0,2629 |
| 40,39 | 0,3015 | 90,48 | -0,293 | 40,24 | 0,2935 | 90,46 | -0,2647 | 40,16 | 0,2684 | 90,55 | -0,263 |
| 40,36 | 0,3015 | 90,51 | -0,293 | 40,21 | 0,2934 | 90,5 | -0,2648 | 40,13 | 0,2683 | 90,59 | -0,263 |
| 40,33 | 0,3015 | 90,54 | -0,293 | 40,17 | 0,2934 | 90,53 | -0,2648 | 40,1 | 0,2683 | 90,62 | -0,2631 |
| 40,29 | 0,3014 | 90,58 | -0,2931 | 40,14 | 0,2934 | 90,56 | -0,2648 | 40,06 | 0,2682 | 90,65 | -0,2631 |
| 40,26 | 0,3014 | 90,61 | -0,2931 | 40,1 | 0,2933 | 90,6 | -0,2649 | 40,03 | 0,2681 | 90,69 | -0,2632 |
| 40,22 | 0,3014 | 90,64 | -0,2932 | 40,07 | 0,2933 | 90,63 | -0,2649 | 39,99 | 0,268 | 90,72 | -0,2632 |
| 40,19 | 0,3013 | 90,68 | -0,2932 | 40,04 | 0,2933 | 90,66 | -0,2649 | 39,96 | 0,2679 | 90,75 | -0,2633 |
| 40,16 | 0,3013 | 90,71 | -0,2933 | 40,01 | 0,2932 | 90,7 | -0,265 | 39,93 | 0,2678 | 90,79 | -0,2633 |
| 40,13 | 0,3013 | 90,74 | -0,2933 | 39,97 | 0,2932 | 90,73 | -0,265 | 39,89 | 0,2677 | 90,82 | -0,2634 |
| 40,09 | 0,3012 | 90,78 | -0,2933 | 39,94 | 0,2932 | 90,76 | -0,265 | 39,86 | 0,2676 | 90,85 | -0,2634 |
| 40,06 | 0,3012 | 90,81 | -0,2934 | 39,91 | 0,2931 | 90,8 | -0,2651 | 39,83 | 0,2675 | 90,89 | -0,2635 |
| 40,03 | 0,3012 | 90,84 | -0,2934 | 39,87 | 0,2931 | 90,83 | -0,2651 | 39,79 | 0,2674 | 90,92 | -0,2635 |
| 39,99 | 0,3011 | 90,88 | -0,2935 | 39,84 | 0,2931 | 90,86 | -0,2651 | 39,76 | 0,2674 | 90,95 | -0,2636 |
| 39,96 | 0,3011 | 90,91 | -0,2935 | 39,81 | 0,293 | 90,9 | -0,2652 | 39,73 | 0,2673 | 90,99 | -0,2636 |
| 39,93 | 0,3011 | 90,94 | -0,2936 | 39,77 | 0,293 | 90,93 | -0,2652 | 39,69 | 0,2672 | 91,02 | -0,2637 |
| 39,89 | 0,301 | 90,98 | -0,2936 | 39,74 | 0,293 | 90,96 | -0,2652 | 39,66 | 0,2671 | 91,06 | -0,2637 |
| 39,86 | 0,301 | 91,01 | -0,2936 | 39,71 | 0,2929 | 91 | -0,2653 | 39,63 | 0,267 | 91,09 | -0,2638 |
| 39,83 | 0,301 | 91,04 | -0,2937 | 39,67 | 0,2929 | 91,03 | -0,2653 | 39,59 | 0,2669 | 91,12 | -0,2638 |
| 39,79 | 0,3009 | 91,08 | -0,2937 | 39,64 | 0,2929 | 91,06 | -0,2653 | 39,56 | 0,2668 | 91,15 | -0,2639 |
| 39,76 | 0,3009 | 91,11 | -0,2938 | 39,61 | 0,2928 | 91,1 | -0,2654 | 39,53 | 0,2667 | 91,19 | -0,2639 |
| 39,72 | 0,3009 | 91,14 | -0,2938 | 39,57 | 0,2928 | 91,13 | -0,2654 | 39,49 | 0,2667 | 91,22 | -0,264 |
| 39,69 | 0,3008 | 91,18 | -0,2938 | 39,54 | 0,2928 | 91,16 | -0,2654 | 39,46 | 0,2665 | 91,25 | -0,264 |
| 39,66 | 0,3008 | 91,21 | -0,2939 | 39,51 | 0,2927 | 91,2 | -0,2655 | 39,43 | 0,2664 | 91,29 | -0,2641 |
| 39,63 | 0,3008 | 91,24 | -0,2939 | 39,47 | 0,2927 | 91,23 | -0,2655 | 39,39 | 0,2664 | 91,32 | -0,2641 |
| 39,59 | 0,3007 | 91,28 | -0,294 | 39,44 | 0,2926 | 91,26 | -0,2655 | 39,36 | 0,2663 | 91,35 | -0,2642 |
| 39,56 | 0,3007 | 91,31 | -0,294 | 39,4 | 0,2926 | 91,3 | -0,2656 | 39,33 | 0,2662 | 91,39 | -0,2642 |
| 39,53 | 0,3007 | 91,34 | -0,2941 | 39,37 | 0,2926 | 91,33 | -0,2656 | 39,29 | 0,2661 | 91,42 | -0,2643 |
| 39,49 | 0,3006 | 91,38 | -0,2941 | 39,34 | 0,2925 | 91,36 | -0,2656 | 39,26 | 0,266 | 91,45 | -0,2643 |
| 39,46 | 0,3006 | 91,41 | -0,2942 | 39,31 | 0,2925 | 91,4 | -0,2657 | 39,23 | 0,2659 | 91,49 | -0,2644 |
| 39,43 | 0,3006 | 91,44 | -0,2942 | 39,27 | 0,2925 | 91,43 | -0,2657 | 39,19 | 0,2658 | 91,52 | -0,2644 |
| 39,39 | 0,3005 | 91,48 | -0,2942 | 39,24 | 0,2924 | 91,46 | -0,2657 | 39,16 | 0,2656 | 91,55 | -0,2645 |
| 39,36 | 0,3005 | 91,51 | -0,2943 | 39,21 | 0,2924 | 91,5 | -0,2658 | 39,13 | 0,2655 | 91,59 | -0,2645 |
| 39,33 | 0,3005 | 91,54 | -0,2943 | 39,17 | 0,2924 | 91,53 | -0,2658 | 39,09 | 0,2654 | 91,62 | -0,2646 |
| 39,29 | 0,3004 | 91,58 | -0,2944 | 39,14 | 0,2923 | 91,56 | -0,2658 | 39,06 | 0,2653 | 91,65 | -0,2646 |
| 39,26 | 0,3004 | 91,61 | -0,2944 | 39,11 | 0,2923 | 91,6 | -0,2659 | 39,03 | 0,2652 | 91,69 | -0,2647 |
| 39,23 | 0,3004 | 91,64 | -0,2944 | 39,07 | 0,2923 | 91,63 | -0,2659 | 38,99 | 0,2651 | 91,72 | -0,2647 |
| 39,19 | 0,3003 | 91,68 | -0,2945 | 39,04 | 0,2922 | 91,66 | -0,2659 | 38,96 | 0,265 | 91,75 | -0,2648 |
| 39,16 | 0,3003 | 91,71 | -0,2945 | 39,01 | 0,2922 | 91,7 | -0,266 | 38,93 | 0,2649 | 91,79 | -0,2648 |
| 39,13 | 0,3003 | 91,74 | -0,2946 | 38,97 | 0,2922 | 91,73 | -0,266 | 38,89 | 0,2648 | 91,82 | -0,2649 |
| 39,09 | 0,3002 | 91,78 | -0,2946 | 38,94 | 0,2921 | 91,76 | -0,266 | 38,86 | 0,2647 | 91,85 | -0,2649 |
| 39,06 | 0,3002 | 91,81 | -0,2946 | 38,91 | 0,2921 | 91,8 | -0,266 | 38,83 | 0,2646 | 91,89 | -0,265 |
| 39,03 | 0,3002 | 91,84 | -0,2947 | 38,87 | 0,2921 | 91,83 | -0,2661 | 38,79 | 0,2645 | 91,92 | -0,265 |
| 38,99 | 0,3002 | 91,88 | -0,2947 | 38,84 | 0,292 | 91,87 | -0,2661 | 38,76 | 0,2644 | 91,95 | -0,2651 |
| 38,96 | 0,3001 | 91,91 | -0,2948 | 38,81 | 0,292 | 91,9 | -0,2661 | 38,73 | 0,2644 | 91,99 | -0,2651 |
| 38,93 | 0,3001 | 91,94 | -0,2948 | 38,77 | 0,2919 | 91,93 | -0,2662 | 38,69 | 0,2643 | 92,02 | -0,2652 |
| 38,89 | 0,3 | 91,98 | -0,2949 | 38,74 | 0,2919 | 91,96 | -0,2662 | 38,66 | 0,2642 | 92,06 | -0,2652 |
| 38,86 | 0,3 | 92,01 | -0,2949 | 38,7 | 0,2919 | 92 | -0,2663 | 38,63 | 0,2641 | 92,09 | -0,2653 |
| 38,83 | 0,3 | 92,04 | -0,2949 | 38,67 | 0,2918 | 92,03 | -0,2663 | 38,59 | 0,264 | 92,12 | -0,2653 |
| 38,79 | 0,2999 | 92,08 | -0,295 | 38,64 | 0,2918 | 92,06 | -0,2663 | 38,56 | 0,2639 | 92,15 | -0,2654 |
| 38,76 | 0,2999 | 92,11 | -0,295 | 38,6 | 0,2918 | 92,1 | -0,2664 | 38,53 | 0,2638 | 92,19 | -0,2654 |
| 38,72 | 0,2999 | 92,14 | -0,2951 | 38,57 | 0,2917 | 92,13 | -0,2664 | 38,49 | 0,2637 | 92,22 | -0,2655 |
| 38,69 | 0,2998 | 92,18 | -0,2951 | 38,54 | 0,2917 | 92,16 | -0,2664 | 38,46 | 0,2636 | 92,26 | -0,2655 |
| 38,66 | 0,2998 | 92,21 | -0,2951 | 38,51 | 0,2917 | 92,2 | -0,2664 | 38,43 | 0,2635 | 92,29 | -0,2656 |
| 38,63 | 0,2998 | 92,24 | -0,2952 | 38,47 | 0,2916 | 92,23 | -0,2665 | 38,39 | 0,2634 | 92,32 | -0,2656 |
| 38,59 | 0,2997 | 92,28 | -0,2952 | 38,44 | 0,2916 | 92,26 | -0,2665 | 38,36 | 0,2633 | 92,35 | -0,2657 |
| 38,56 | 0,2997 | 92,31 | -0,2953 | 38,41 | 0,2916 | 92,3 | -0,2665 | 38,33 | 0,2632 | 92,39 | -0,2657 |
| 38,52 | 0,2997 | 92,34 | -0,2953 | 38,37 | 0,2915 | 92,33 | -0,2666 | 38,29 | 0,2632 | 92,42 | -0,2658 |
| 38,49 | 0,2996 | 92,38 | -0,2954 | 38,34 | 0,2915 | 92,36 | -0,2666 | 38,26 | 0,2631 | 92,45 | -0,2658 |
| 38,46 | 0,2996 | 92,41 | -0,2954 | 38,3 | 0,2914 | 92,4 | -0,2666 | 38,22 | 0,263 | 92,49 | -0,2658 |
| 38,43 | 0,2996 | 92,44 | -0,2955 | 38,27 | 0,2914 | 92,43 | -0,2667 | 38,19 | 0,2629 | 92,52 | -0,2659 |
| 38,39 | 0,2995 | 92,48 | -0,2955 | 38,24 | 0,2914 | 92,46 | -0,2667 | 38,16 | 0,2628 | 92,55 | -0,2659 |
| 38,36 | 0,2995 | 92,51 | -0,2955 | 38,2 | 0,2913 | 92,5 | -0,2667 | 38,12 | 0,2627 | 92,59 | -0,266 |
| 38,33 | 0,2995 | 92,54 | -0,2956 | 38,17 | 0,2913 | 92,53 | -0,2668 | 38,09 | 0,2626 | 92,62 | -0,266 |
| 38,29 | 0,2994 | 92,58 | -0,2956 | 38,14 | 0,2913 | 92,56 | -0,2668 | 38,06 | 0,2625 | 92,66 | -0,2661 |
| 38,26 | 0,2994 | 92,61 | -0,2957 | 38,1 | 0,2912 | 92,6 | -0,2668 | 38,03 | 0,2625 | 92,69 | -0,2661 |
| 38,22 | 0,2994 | 92,64 | -0,2957 | 38,07 | 0,2912 | 92,63 | -0,2669 | 37,99 | 0,2624 | 92,72 | -0,2662 |
| 38,19 | 0,2993 | 92,68 | -0,2957 | 38,04 | 0,2912 | 92,66 | -0,2669 | 37,96 | 0,2623 | 92,76 | -0,2662 |
| 38,16 | 0,2993 | 92,71 | -0,2958 | 38 | 0,2911 | 92,7 | -0,2669 | 37,93 | 0,2622 | 92,79 | -0,2663 |
| 38,12 | 0,2993 | 92,74 | -0,2958 | 37,97 | 0,2911 | 92,73 | -0,267 | 37,89 | 0,2621 | 92,82 | -0,2663 |
| 38,09 | 0,2993 | 92,78 | -0,2959 | 37,94 | 0,2911 | 92,76 | -0,267 | 37,86 | 0,2619 | 92,86 | -0,2664 |
| 38,06 | 0,2992 | 92,81 | -0,2959 | 37,91 | 0,291 | 92,8 | -0,267 | 37,83 | 0,2618 | 92,89 | -0,2664 |
[truncated: 191,836 more chars]
